# Multi‐omic profiling defines three distinct molecular subtypes of urothelial carcinoma with implications for precision therapy

**DOI:** 10.1002/ctm2.70638

**Published:** 2026-03-10

**Authors:** Nils C. H. van Creij, Piotr Tymoszuk, Florian Handle, Andreas Seeber, Teresa Sellemond, Agnieszka Martowicz, Eva Comperat, Hamed Wafa, Steffen Ormanns, Michael Günther, Walther Parson, Maxim Noeparast, Frédéric R. Santer, José Daniel Subiela, Petros Grivas, Roger Li, Zoran Culig, Renate Pichler

**Affiliations:** ^1^ Department of Urology, Division of Experimental Urology Medical University of Innsbruck Innsbruck Austria; ^2^ Data Analytics As a Service Tirol Wörgl Austria; ^3^ XPseq Analytics GmbH Innsbruck Austria; ^4^ Department of Internal Medicine V (Hematology and Oncology), Comprehensive Cancer Center Innsbruck Medical University of Innsbruck Innsbruck Austria; ^5^ Department of Oncology, Hematology and Palliative Care General Hospital of Oberwart Oberwart Austria; ^6^ Tyrolean Cancer Research Institute (TKFI) Innsbruck Austria; ^7^ Department of Pathology Medical University of Vienna Vienna Austria; ^8^ INNPATH GmbH, Tirol Kliniken Innsbruck Innsbruck Austria; ^9^ Institute of General Pathology, Medical University of Innsbruck Innsbruck Austria; ^10^ Institute of Legal Medicine, Medical University of Innsbruck Innsbruck Austria; ^11^ Translational Oncology, II. Med Clinics Hematology and Oncology University of Augsburg Augsburg Germany; ^12^ Department of Gynecology and Obstetrics Medical University of Innsbruck Innsbruck Austria; ^13^ Department of Urology, Instituto Ramón y Cajal de Investigación Sanitaria Hospital Universitario Ramón y Cajal, Universidad de Alcalá Madrid Spain; ^14^ Fred Hutchinson Cancer Center University of Washington Seattle Washington USA; ^15^ Department of GU Oncology H. Lee Moffitt Cancer Center and Research Institute Tampa Florida USA; ^16^ Department of Urology, Comprehensive Cancer Center Innsbruck Medical University of Innsbruck Innsbruck Austria

**Keywords:** bladder cancer, drug response, molecular classification, proteomics, transcriptomics

## Abstract

**Background:**

Urothelial carcinoma (UC) is a biologically heterogeneous disease, and current molecular classifications have limited integration into clinical decision‐making. To further pursue precision oncology efforts in UC, we developed a molecular classification framework applicable to transcriptomic and proteomic data from non–muscle‐invasive bladder cancer (NMIBC), muscle‐invasive bladder cancer (MIBC) and urothelial cancer cell lines.

**Methods:**

Using a whole‐transcriptome self‐organised map and regularised semi‐supervised clustering of 4439 bulk NMIBC and MIBC transcriptomes and proteomes, and 33 UC cell lines, we identified three molecular UC clusters. Making use of both in silico and in vitro approaches, we selected promising treatment approaches for each cluster.

**Results:**

The three developed clusters displayed distinct signatures of mRNA, proteins, biological processes, metabolism and essential driver genes. They also differed in prognosis and machine learning‐predicted treatment vulnerabilities and resistance. High‐risk, stroma‐rich Cluster #1 cancers were predicted to respond to selected cytotoxic drugs, ferroptosis inducers and PARP inhibitors. For the aggressive, fast‐proliferating, immune‐infiltrated Cluster #2 tumours with basal/squamous differentiation, cytotoxic agents and EGFR/ERBB‐ and MEK/ERK‐targeting therapies were proposed. Cluster #3 cancers of predominantly luminal papillary phenotype with scarce stroma and immune infiltration were enriched with NMIBC and low‐risk malignancies. For patients with Cluster #3 tumours, selected epigenetic drugs or EGFR/FGFR inhibitors may represent attractive treatment options.

**Conclusions:**

Our novel molecular taxonomy holds promise as a practical framework for patient risk stratification and clinical trials in UC. Our molecular classification scheme may facilitate personalised transcriptome‐ and proteome‐based risk assessment and clinical trial design for the development of various therapeutics.

**key points:**

- We developed three UC clusters, applicable for MIBC and NMIBC, which were validated using transcriptomic- and proteomic datasets.
- Publically available UC cell lines were assigned to the clusters, to have in vitro models representing each cluster.
- The clusters differ in molecular and biological signatures, with distinct prognostic and therapeutic characteristics.

## Graphical abstract

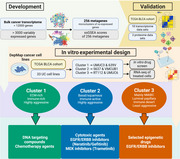

## 1 BACKGROUND

Urothelial carcinoma (UC) ranks as the ninth most common cancer worldwide, with an estimated 150 000 newly diagnosed patients each year.^1^, ^2^ At primary diagnosis, approximately 70% of patients are diagnosed with non–muscle‐invasive bladder cancer (NMIBC), 25% of the patients have muscle‐invasive bladder cancer (MIBC) and about 5% present with de novo metastatic disease. NMIBC has a high recurrence rate, and around 30% of cases progress to MIBC.^3^ Advanced UC is still associated with poor prognosis and high mortality rates. After decades of relying almost exclusively on chemotherapy, novel treatment strategies have emerged, including immune checkpoint inhibitors (ICIs), antibody–drug conjugates (ADC) and targeted therapies, such as fibroblast growth factor receptor3 (FGFR3) inhibitors.^4^ Recently, two phase 3 trials with drug combinations have demonstrated remarkable survival benefits compared to the conventional standard of care in metastatic UC (mUC): those new therapy combinations include enfortumab vedotin (EV; ADC) plus pembrolizumab (programmed cell death protein 1 [PD‐1] inhibitor), which is the preferred frontline regimen^5^ as well as gemcitabine/cisplatin plus nivolumab, which targets the PD‐1 receptor.^6^ Furthermore, the pan‐FGFR inhibitor erdafitinib has been approved for mUC harbouring *FGFR3* susceptible alterations after progression on prior anti‐PD‐1/L1 treatment.^7^ These breakthroughs underline the strength of novel targeted therapies and have led to new guideline recommendations.^8^ However, the significant molecular heterogeneity and phenotypic variability of UC, resulting in variable response rates ranging 20%–70%, pose a major challenge for further development of novel therapeutic agents, predictive biomarkers and a personalised treatment approach.

Whole‐transcriptome profiling has provided the first insights into the molecular heterogeneity of UC. By transcriptome‐based clustering, several molecular classification systems have been developed for NMIBC,^9^, ^10^, ^11^ MIBC,^12^, ^13^, ^14^ or UC in general.^15^, ^16^, ^17^, ^18^, ^19^, ^20^, ^21^ The most used classification scheme of NMIBC proposed by the UROMOL study identifies subtypes 1, 2a, 2b and3.^9^, ^11^ For MIBC, Kamoun et al. introduced six consensus molecular classes: stroma‐rich, luminal unstable, luminal–non‐specified, basal/squamous, luminal–papillary and neuroendocrine‐like,^22^ which substantially advanced our understanding of MIBC biology.^12^, ^17^, ^18^, ^22^ Yet, unclear prognostic, predictive and therapeutic relevance of the existing molecular classification schemes hinders their implementation into guidelines and clinical practice. Furthermore, most molecular classification schemes were developed separately either for NMIBC or MIBC alone and rely exclusively on bulk cancer transcriptome data.^9^ Their validity for pan‐UC, UC cell lines and compatibility with other ‐omics data, such as proteomics or single cell transcriptomics has not been investigated so far, but is urgently needed for clinical practice and understanding molecular heterogeneity. Herein, we aimed to establish a molecular classification scheme applicable across bulk tumour data, NMIBC and MIBC, transcriptomics, proteomics and UC cell line transcriptomes. By a combined approach involving a self‐organised map (SOM) and regularised semi‐supervised clustering of transcriptomes and proteomes, we identified three biologically and clinically distinct UC clusters in a large analysis of 20 independent cohorts comprising over 4000 UC samples and 33 UC cell lines. Representative models could furthermore predict cluster‐specific treatment approaches that might pave the way towards a more personalised medicine approach.

## 2 METHODS

An overview of the whole‐transcriptome UC cohorts and analysis strategy is shown in the graphical abstract. Details on data sources, data processing and management, statistics, and bioinformatics are provided in the Supporting Information, and Tables S1 and S2.

### 2.1 Software and data sources

Data management, statistical and bioinformatic analyses were done with R version ≥ 4.2.3 (R Foundation for Statistical Computing). The cBioportal, Gene Expression Omnibus (GEO) and Array Express repositories were screened for transcriptomic and proteomic studies on primary, recurrent, progressing and metastatic urothelial cancers. Herein, 19 bulk cancer transcriptome cohorts with at least 80 samples and 18 000 quantified genes (TCGA BLCA,^12^, ^23^ IMvigor,^24^, ^25^ GSE13507,^26^ GSE32548,^27^ GSE48075,^28^ GSE48276,^28^ GSE83586,^29^ GSE86411,^30^ GSE87304,^31^ GSE120736,^32^ GSE124305,^33^ GSE128192,^34^ GSE128701,^35^ GSE128959,^36^ GSE198269,^37^ GSE203149,^38^ E‐MTAB‐4321,^9^ Groeneveld 2024,^39^ BCAN^40^) and three bulk cancer proteomes with 1600–9265 quantified proteins (Groeneveld 2024,^39^ Dressler 2024,^41^ Stroggilos 2020^42^) were analysed (Tables 1 and 2, and S3 and S4). Additionally, gene expression and CRISPR‐Cas9 knockout (KO) screening data for 33 urothelial cancer cell lines with reliable assignment to UC clusters were imported from the DepMap portal.^43^

**TABLE 1:** Demographic and clinical characteristic of urothelial cancer patients analysed in the current study.

Variable^a^	All patients^b^	NMIBC^c^	MIBC^d^	Test type^e^	Significance^e^	Effect size^e^
Patients, *N*	4298	1380	2817			
Sex	Female: 11% (468)	Female: 11% (148)	Female: 11% (320)	*χ* ^2^	*p* =.009	*V* =.062
	Male: 39% (1670)	Male: 47% (649)	Male: 36% (1021)			
	Unknown: 50% (2160)	Unknown: 42% (583)	Unknown: 52% (1476)			
Age, years	67 [IQR: 59–75]	68 [IQR: 61–76]	67 [IQR: 59–74]	Mann–Whitney	*p* =.023	*r* =.055
	Range: 22–96	Range: 24–96	Range: 22–94			
	Complete: *n* = 1914	Complete: *n* = 616	Complete: *n* = 1298			
Race	Asian: 1.3% (55)	Not provided	Asian: 2% (55)			
	Black or African American: 1.1% (47)		Black or African American: 1.7% (47)			
	White: 17% (724)		White: 26% (724)			
	Other:.72% (31)		Other: 1.1% (31)			
	Unknown: 80% (3441)		Unknown: 70% (1960)			
Body mass index, kg/m^2^	26 [IQR: 23–30]	Not provided	26 [IQR: 23–30]			
	Range: 15–68		Range: 15–68			
	Complete: n = 357		Complete: *n* = 357			
Body mass class	Normal: 3.4% (148)	Not provided	Normal: 5.3% (148)			
	Overweight: 2.9% (124)		Overweight: 4.4% (124)			
	Obesity: 2% (85)		Obesity: 3% (85)			
	Unknown: 92% (3941)		Unknown: 87% (2460)			
Smoking history	Never: 5.4% (230)	Not provided	Never: 8.2% (230)			
	Current or previous: 13% (557)		Current or previous: 20% (557)			
	Unknown: 82% (3511)		Unknown: 72% (2030)			
History of BCG treatment	No: 19% (813)	No: 27% (372)	No: 16% (441)	*χ* ^2^	ns (*p* =.56)	*V* =.024
	Yes: 4.2% (180)	Yes: 6.4% (88)	Yes: 3.3% (92)			
	Unknown: 77% (3305)	Unknown: 67% (920)	Unknown: 81% (2284)			
Neoadjuvant systemic chemotherapy	No: 21% (903)	Not provided	No: 32% (903)			
	Yes: 7.8% (335)		Yes: 12% (335)			
	Unknown: 71% (3060)		Unknown: 56% (1579)			
Cystectomy	No: 14% (621)	No: 37% (510)	No: 3.9% (111)	χ^2^	*p* <.001	*V* =.68
	Yes: 7.7% (329)	Yes: 2.7% (37)	Yes: 10% (292)			
	Unknown: 78% (3348)	Unknown: 60% (833)	Unknown: 86% (2414)			
Adjuvant systemic chemotherapy	No: 11% (477)	No: 4.9% (68)	No: 15% (409)	*χ* ^2^	*p* <.001	*V* =.31
	Yes: 19% (827)	Yes: 0% (0)	Yes: 29% (827)			
	Unknown: 70% (2994)	Unknown: 95% (1312)	Unknown: 56% (1581)			
Death during follow‐up	No: 18% (770)	No: 17% (229)	No: 19% (541)	*χ* ^2^	*p* <.001	*V* =.19
	Yes: 16% (685)	Yes: 6.8% (94)	Yes: 21% (591)			
	Unknown: 66% (2843)	Unknown: 77% (1057)	Unknown: 60% (1685)			
Follow‐up time, days	660 [IQR: 330–1600]	1800 [IQR: 800–1800]	570 [IQR: 270–1100]	Peto–Peto	*p* <.001	
	Range: 0–6400	Range: 0–4600	Range: 0–6400			
	Complete: *n* = 1443	Complete: *n* = 323	Complete: *n* = 1120			
Relapse during follow‐up	No: 8.7% (375)	No: 7% (96)	No: 9.9% (279)	*χ* ^2^	ns (*p* =.54)	*V* =.036
	Yes: 5.6% (241)	Yes: 3.9% (54)	Yes: 6.6% (187)			
	Unknown: 86% (3682)	Unknown: 89% (1230)	Unknown: 83% (2351)			
Relapse‐free survival, days	470 [IQR: 210–730]	590 [IQR: 200–730]	460 [IQR: 220–730]	Peto–Peto	ns (*p* =.86)	
	Range: 0–5000	Range: 4.1–730	Range: 0–5000			
	Complete: *n* = 622	Complete: *n* = 154	Complete: *n* = 468			
MIBC progression during follow‐up	No: 13% (561)	No: 41% (561)	Not provided			
	Yes: 1.2% (53)	Yes: 3.8% (53)				
	Unknown: 86% (3684)	Unknown: 56% (766)				
MIBC progression‐free survival, days	820 [IQR: 560–1200]	820 [IQR: 560–1200]	Not provided			
	Range: 0–2300	Range: 0–2300				
	Complete: *n* = 614	Complete: *n* = 614				

Qualitative variables are presented as percentages and counts of the categories within the complete observation set. Qualitative variables are shown as medians with interquartile ranges and ranges. Statistical significance of differences between non‐muscle invasive (NMIBC) and muscle invasive cancers (MIBC) was assessed by *χ* ^2^ test with Cramer's *V* effect size statistic (qualitative variables), Mann–Whitney test with *r* effect size statistic (quantitative variables) or Peto–Peto test (survival). *p* values were corrected for multiple testing with the false discovery rate method.

^a^ BCG, Bacillus Calmette–Guerin; MIBC progression, progression of primary NMIBC to MIBC.; Relapse, recurrence or progression of the disease after initial treatment

^b^ BCAN: *n* = 174, Dressler 2024: *n* = 242, E‐MTAB‐4321: *n* = 476, Groeneveld 2024: *n* = 198, GSE120736: *n* = 145, GSE124305: *n* = 133, GSE128192: *n* = 112, GSE128701: *n* = 136, GSE128959: *n* = 70, GSE13507: *n* = 171, GSE198269: *n* = 394, GSE203149: *n* = 170, GSE32548: *n* = 131, GSE48075: *n* = 142, GSE48276: *n* = 116, GSE83586: *n* = 307, GSE86411: *n* = 132, GSE87304: *n* = 305, IMvigor: *n* = 220, Stroggilos 2020: *n* = 117, TCGA BLCA: *n* = 407.

^c^ Dressler 2024: *n* = 167, E‐MTAB‐4321: *n* = 460, Groeneveld 2024: *n* = 87, GSE120736: *n* = 84, GSE128959: *n* = 64, GSE13507: *n* = 68, GSE32548: *n* = 92, GSE48075: *n* = 70, GSE83586: *n* = 58, GSE86411: *n* = 132, Stroggilos 2020: *n* = 98.

^d^ BCAN: *n* = 174, Dressler 2024: *n* = 75, E‐MTAB‐4321: *n* = 16, Groeneveld 2024: *n* = 82, GSE120736: *n* = 61, GSE124305: *n* = 133, GSE128192: *n* = 112, GSE128701: *n* = 136, GSE128959: *n* = 3, GSE13507: *n* = 41, GSE198269: *n* = 394, GSE203149: *n* = 170, GSE32548: *n* = 38, GSE48075: *n* = 72, GSE48276: *n* = 116, GSE83586: *n* = 243, GSE87304: *n* = 305, IMvigor: *n* = 220, Stroggilos 2020: *n* = 19, TCGA BLCA: *n* = 407.

^e^ Comparison of the NMIBC and MIBC groups, the ‘unknown’ category was excluded.

**TABLE 2:** Pathological characteristic of transcriptomic and proteomic bulk cancer samples analysed in the current study.

Variable	All samples^a^	NMIBC^b^	MIBC^c^	Test type^d^	Significance^d^	Effect size^d^
Samples, *N*	4439	1505	2872			
Cancer tissue type	Bladder: 95% (4236)	Bladder: 100% (1505)	Bladder: 93% (2669)	*χ* ^2^	*p* <.001	*V* =.16
	Urinary organ, non‐bladder: 4.1% (183)	Urinary organ, non‐bladder: 0% (0)	Urinary organ, non‐bladder: 6.4% (183)			
	Non‐urinary organ, distant metastasis:.45% (20)	Non‐urinary organ, distant metastasis: 0% (0)	Non‐urinary organ, distant metastasis:.7% (20)			
Invasiveness	Non‐muscle invasive: 34% (1505)	Non‐muscle invasive: 100% (1505)	Non‐muscle invasive: 0% (0)			
	Muscle invasive: 65% (2872)	Muscle invasive: 0% (0)	Muscle invasive: 100% (2872)			
	Unknown: 1.4% (62)	Unknown: 0% (0)	Unknown: 0% (0)			
Pathological tumour stage	T2: 21% (952)	T2: 0% (0)	T2: 33% (952)			
	T3: 14% (600)	T3: 0% (0)	T3: 21% (600)			
	T4: 4.9% (219)	T4: 0% (0)	T4: 7.6% (219)			
	T1/Ta/Tis: 31% (1373)	T1/Ta/Tis: 91% (1373)	T1/Ta/Tis: 0% (0)			
	T2‐4:.36% (16)	T2‐4: 0% (0)	T2‐4:.56% (16)			
	T3‐4:.56% (25)	T3‐4: 0% (0)	T3‐4:.87% (25)			
	Unknown: 28% (1254)	Unknown: 8.8% (132)	Unknown: 37% (1060)			
Pathological node stage	N0: 25% (1113)	N0: 26% (387)	N0: 25% (726)	*χ* ^2^	*p* <.001	*V* =.34
	N1: 4.6% (205)	N1: 0% (0)	N1: 7.1% (205)			
	N2: 2.9% (127)	N2: 0% (0)	N2: 4.4% (127)			
	N3:.38% (17)	N3: 0% (0)	N3:.59% (17)			
	Unknown: 67% (2977)	Unknown: 74% (1118)	Unknown: 63% (1797)			
Pathological metastasis stage	M0: 15% (683)	M0: 16% (237)	M0: 16% (446)	*χ* ^2^	*p* <.001	*V* =.2
	M1: 1.3% (58)	M1: 0% (0)	M1: 2% (58)			
	Unknown: 83% (3698)	Unknown: 84% (1268)	Unknown: 82% (2368)			
Pathological grade	G1: 1.4% (61)	G1: 4.1% (61)	G1: 0% (0)	*χ* ^2^	*p* <.001	*V* =.47
	G2: 3.1% (139)	G2: 8.4% (126)	G2:.42% (12)			
	G3: 12% (511)	G3: 14% (213)	G3: 10% (292)			
	Unknown: 84% (3728)	Unknown: 73% (1105)	Unknown: 89% (2568)			
Histological grade	Low grade: 10% (443)	Low grade: 27% (402)	Low grade: 1.4% (41)	*χ* ^2^	*p* <.001	*V* =.57
	High grade: 16% (730)	High grade: 15% (232)	High grade: 17% (498)			
	Unknown: 74% (3266)	Unknown: 58% (871)	Unknown: 81% (2333)			

Qualitative variables are presented as percentages and counts of the categories within the complete observation set. Qualitative variables are shown as medians with interquartile ranges and ranges. Statistical significance of differences between non‐muscle invasive (NMIBC) and muscle invasive cancers (MIBC) was assessed by *χ* ^2^ test with Cramer's *V* effect size statistic (qualitative variables), Mann–Whitney test with *r* effect size statistic (quantitative variables) or Peto–Peto test (survival). *p* values were corrected for multiple testing with the false discovery rate method.

^a^ BCAN: *n* = 174, Dressler 2024: *n* = 242, E‐MTAB‐4321: *n* = 476, Groeneveld 2024: *n* = 198, GSE120736: *n* = 145, GSE124305: *n* = 133, GSE128192: *n* = 112, GSE128701: *n* = 136, GSE128959: *n* = 192, GSE13507: *n* = 188, GSE198269: *n* = 394, GSE203149: *n* = 171, GSE32548: *n* = 131, GSE48075: *n* = 142, GSE48276: *n* = 116, GSE83586: *n* = 307, GSE86411: *n* = 132, GSE87304: *n* = 305, IMvigor: *n* = 221, Stroggilos 2020: *n* = 117, TCGA BLCA: *n* = 407.

^b^ Dressler 2024: *n* = 167, E‐MTAB‐4321: *n* = 460, Groeneveld 2024: *n* = 87, GSE120736: *n* = 84, GSE128959: *n* = 154, GSE13507: *n* = 103, GSE32548: *n* = 92, GSE48075: *n* = 70, GSE83586: *n* = 58, GSE86411: *n* = 132, Stroggilos 2020: *n* = 98.

^c^ BCAN: *n* = 174, Dressler 2024: *n* = 75, E‐MTAB‐4321: *n* = 16, Groeneveld 2024: *n* = 82, GSE120736: *n* = 61, GSE124305: *n* = 133, GSE128192: *n* = 112, GSE128701: *n* = 136, GSE128959: *n* = 35, GSE13507: *n* = 62, GSE198269: *n* = 394, GSE203149: *n* = 171, GSE32548: *n* = 38, GSE48075: *n* = 72, GSE48276: *n* = 116, GSE83586: *n* = 243, GSE87304: *n* = 305, IMvigor: *n* = 221, Stroggilos 2020: *n* = 19, TCGA BLCA: *n* = 407.

^d^ Comparison of the NMIBC and MIBC groups, the ‘unknown’ category was excluded.

Normalised gene and protein expression, genetic alteration data and clinical information provided by the study authors were analysed. Gene and protein expression values were analysed in a $\mathrm{lo}g_{2}$‐transformed form. Missing gene expression Z‐scores (BCAN cohort) and log_2_ protein expression values (Groeneveld 2024 and Dressler 2024) for variables with less than 50% missing entries were imputed with k‐nearest neighbour regressors (Rpackage *impute*).^44^ For some analyses (e.g., clustering, network analyses, prediction of drug resistance), bulk cancer transcriptome and proteome data were processed with ComBat to adjust for the between cohort variability.^45^ Genetic alteration data were analysed in a binarised form (0: wild‐type or two copies of a gene; 1: one or more mutations, at least one copy deletion, at least one additional copy).

Single‐sample gene set enrichment scores of metagenes, signatures of Reactome Pathways (extracted from the MSig Database) and cell proliferation^46^, ^47^, ^48^, ^49^, ^50^, ^51^, ^52^ were computed with the GSVA algorithm.^53^ Levels of infiltrating immune and stromal cells in bulk cancer transcriptome samples were obtained by immune deconvolution with xCell^54^ and QuanTIseq algorithms.^55^ Predictions of drug resistance with transcriptome data were done by RIDGE linear models, linear regression models that incorporate a L2 regularisation term to shrink coefficients and reduce over‐fitting, trained with gene expression and drug resistance information gathered in in vitro drug screening data of GDSC, CTRP2 and PRISM experiments (package *htGLMNET*).^56^, ^57^, ^58^, ^59^ Linear modelling (LM) scores of activity of collecTRI regulons, curated sets of transcription factor‐target gene interactions derived from experimental evidence, and PROGENy signalling pathways, a pathway activity inference method that estimates signalling pathway activation from downstream gene expression changes, were predicted for bulk cancer transcriptome samples with LM tools of *decoupleR*.^60^, ^61^, ^62^ Bulk cancer transcriptome samples were assigned to UROMOL classes of NMIBC^9^ and consensus classes of MIBC^22^ by nearest centroid classifiers from R packages *classifyNMIBC* and *consensusMIBC*.

### 2.2 Harmonisation of clinical and pathological information

Information on muscle invasiveness, if not provided in clinical meta‐data accompanying the molecular data of bulk cancer transcriptome and proteome cohorts or available in the genuine publications, was derived from pathological staging with Ta, T1, Tis and CIS stage tumours classified as NMIBC and T2–T4 specimens as MIBC. To harmonise highly variable and partly incomplete classification of cancer tissue (primary, recurrent, metastatic) and anatomical location between the bulk cancer cohorts, tissue source was subsumed under ‘bladder’, ‘urinary organ, non‐bladder’, and ‘non‐urinary organ, distant metastasis’ based on clinical/pathological metadata and genuine publications.

Survival outcomes were harmonised as follows:

1. Death and overall survival: any‐cause death during the study follow‐up
2. Tumour death and tumour‐specific survival: death due to urothelial cancer or its metastases during the study follow‐up.
3. Relapse and relapse‐free survival: any recurrence or progression of urothelial cancer after initial treatment during the study follow‐up.
4. MIBC progression: progression of primary NMIBC to MIBC during the study follow‐up. Please note that progression of MIBC has not been analysed.

### 2.3 Descriptive statistics, statistical hypothesis testing

Numeric variables are referenced as medians with interquartile ranges, ranges and numbers of complete cases. Qualitative and ordinal features are provided as counts of observations and percentages of the categories, and numbers of complete cases. For survival, Kaplan–Meier estimates of survival times for the 25%, 50% and 75% survival quantiles with 95% confidence intervals (95% CIs) are provided. Estimates and meta‐estimates obtained in statistically hypothesis testing and modelling are presented as estimates of expected values, means or coefficients with 95% CI. Normality and equality of variance were investigated by Shapiro–Wilk and Levene tests.

Statistical significance of differences in numeric variables was determined by T, Mann–Whitney, one‐way ANOVA or Kruskal–Wallis tests with Cohen's *d*, biserial *r* or $\eta^{2}$ effect size statistics. Statistical significance of differences in distributions of categorical variables was assessed by $\chi^{2}$ test with Cramer's *V* effect size metric. Differences in survival were evaluated by Peto–Peto tests and modelled by univariable Cox proportional hazard models (packages *survival* and *survminer*).^63^, ^64^

*P* values were adjusted for multiple testing with the false discovery rate (FDR)^65^ separately for each analytic task and cohort. If not indicated otherwise, effects with FDR‐corrected *p* < .05 were considered significant. As additional control for false‐positive effects, significant effects shared by multiple cohorts are discussed in the reports and subjected to meta‐analyses with DerSimonian–Lair algorithm (differentially regulated genes, gene signatures, drug resistance estimates, regulon and signalling activity scores, metabolic reaction activity estimates: > = 10 cohorts; proteins and protein signatures: > = 2 cohorts).

### 2.4 Definition and technical validation of UC clusters, assignment of transcriptomes and proteomes to UC clusters

UC clusters were defined in the TCGA BLCA cohort with a two‐step procedure. In the first step, metagenes, that is, micro‐clusters of tightly co‐regulated genes, were defined by a toroidal 16 × 16 unit self‐organising map with squared Euclidean distance between the genes and nodes (R packages *cohonen* and *clustTools*).^66^, ^67^ Potential over‐fitting by the SOM and HTKmeans algorithm were addressed by, respectively, holdout validation and cross‐validation. Hyper‐parameters of the SOM (topology, distance measure) were chosen by comparing metrics of explained clustering variance, topology error and neighbourhood preservation in the training (75% observations) and test (25% observations not used in SOM development) subsets of the TCGA BLCA cohort (Figure S1). Hyper‐parameters of the HTKmeans algorithm (number of clusters, shrinkage parameter lambda) were selected by assessing cluster separation and potential misclassification (silhouette method), explained clustering variance, and neighbourhood preservation in the entire TCGA BLCA dataset and fivefold cross‐validation (Figure S2). In the second step, the TCGA BLCA cancer samples were clustered by their single‐sample gene set enrichment analysis (ssGSEA) metagene scores with the hard‐threshold regularised KMEANS algorithm (*clustTools*)^68^ yielding three UC Clusters #1, #2 and #3. Bulk cancer transcriptome samples in validation cohorts (IMvigor, GSE13507, GSE32548, GSE48075, GSE48276, GSE83586, GSE86411, GSE87304, GSE120736, GSE124305, GSE128192, GSE128701, GSE128959, GSE198269, GSE203149, E‐MTAB‐4321 and Groeneveld 2024) were assigned to the clusters by an inverse distance‐weighted nearest neighbour classifier.^69^ Quality of the clustering structure in the TCGA BLCA and validation collectives was assessed by numeric statistics^70^, ^71^ visualisations of UMAP embeddings (uniform manifold approximation and projection)^72^ comparison of ssGSEA scores of the cluster‐defining metagenes, and analysis of squared Euclidean distances between the clusters (Tables S5–S10).

Bulk cancer transcriptome samples in the BCAN cohort, bulk cancer proteome samples and DepMap urothelial cancer cell lines were assigned to UC clusters by multinomial Elastic Net classifiers, a regularised linear classification model that combines L1 and L2 penalties to perform feature selection while controlling multicollinearity (Tables S11–S14).

### 2.5 Genetic alterations, differential expression of genes, proteins and signatures, regulon and signalling activity, predicted drug resistance

Enrichment of genetic alterations in UC clusters of the TCGA BLCA, IMvigor and BCAN cohorts, as compared with expected frequency, was assessed by weighted permutation testing with *perich* package as described in ref. 73 and Supporting Information.

Differences in expression levels of genes and proteins, ssGSEA scores of gene and protein signature, LM activity scores of regulons and signalling pathways (bulk cancer transcriptomes), and predicted drug resistance metrics in bladder clusters were evaluated with a combined procedure. First, significance of differences between the clusters was assessed by one‐way ANOVA with $\eta^{2}$ effect size statistic. Next, differences between the clusters and the cohort means were investigated by post‐hoc one‐sample *T*‐tests. Significantly differentially regulated features were identified by pFDR (ANOVA) <.05, $\eta^{2}\;\geq$.06 and pFDR (*T*‐test) <.05 (R packages *fastTest* and *microViz*).

### 2.6 Network analyses

Correlation and co‐expression networks were built with matrices of Spearman's ρ pairwise correlation coefficients.^74^, ^75^ Differentially regulated genes, proteins, gene/protein signatures or regulons served as network vertices. The network edges were defined by pairwise correlations with ρ≥.5 or ρ≥.3, and were weighted by ρ values. Similarity networks of differentially regulated Reactome Pathway gene signatures were constructed with matrices of Jaccard's similarity coefficients *J* which measure overlaps between member genes/proteins of signature pairs. The network edges were defined by *J* ≥.3 and were weighted by *J* values (R packages *igraph* and *graphExtra*).^75^ Communities of the networks were identified with the Leiden algorithm^76^ and named after their characteristic biological features.

### 2.7 Modelling of metabolism

Activity of metabolic reactions of the RECON2 knowledge model^77^ was assessed by evaluation of the model's gene/protein–reaction association rules in a Monte Carlo simulation.^78^, ^79^ In this simulation, estimates of differential expression for all available genes or proteins were used. Enrichment of RECON metabolic subsystems with significantly activated and inhibited reactions was investigated by comparing the subsystem's reaction frequency in the activated or inhibited reaction set with 10 000 random draws from the entire reaction pool. Metabolic reaction modelling and enrichment analyses were done with *BiGGR* and *biggrExtra*.

### 2.8 CRISPR‐Cas9 gene effects

Effects of gene KO were measured by Chronos scores, a computational method that estimates the fitness consequences of gene disruption by simulating cell growth dynamics after CRISPR perturbation. The Chronos scores were transformed by multiplication by −1, hence, positive values correspond to growth inhibition attributed to the gene KO. Biologically relevant KO effects were assumed for Chronos scores >.5.^80^, ^81^ Biological effects of gene KO in the clusters and differences in gene KO effects between the clusters were assessed by bootstrap tests (R package *boot*).^82^

### 2.9 Cell culture

UMUC3 cells (Sigma‐Aldrich) were cultured in DMEM‐low glucose medium (PAN‐Biotech). RT112, obtained from the German Collection of Microorganisms and Cell Cultures GmbH (DSMZ), and 5637 cells (kindly provided by Prof. Holm, Department of Oral and Maxillofacial Surgery, Medical University Innsbruck) were cultured in RPMI 1640 medium (PAN‐Biotech). 639V (DSMZ) and VMCUB1 (DSMZ) cells were cultured in DMEM‐high glucose medium (BioWest). UMUC6 cells, obtained from the UK Health Security Agency, were cultured in Gibco Minimum Essential Media (MEM, ThermoFischer Scientific) with 1% non‐essential amino acids. All media were supplemented with 10% foetal calf serum (PAN‐Biotech), 1% penicillin/streptomycin (Lonza) and 1% GlutaMAX (Gibco, Thermo Fisher Scientific). Routine mycoplasma testing was performed for all cell cultures and STR profiling was performed to verify the authenticity of the cell lines.

### 2.10 Chemicals

Afatinib, cobimetinib, decitabine, erdafitinib, gefitinib, gemcitabine, neratinib and trametinib were purchased from MedChemExpress as 10 mM stock solution in DMSO. Cisplatin was purchased from Merck‐Sigma and dissolved in DMSO to 1 mg/mL stock solutions. 1S.3R‐RSL‐3 was kindly provided by Timon Adoph (Department of Medicine I, Gastroenterology, Hepatology, Endocrinology & Metabolism, Medical University of Innsbruck), and stored as 4.53 mM stock solutions in DMSO. Stocks were stored as small aliquots at −80°C until further use.

### 2.11 In vitro drug screen

UMUC3, 5637, RT112, 639V, VMCUB1 and UMUC6 cells were seeded into 96‐well plates at densities of 4000, 8000, 10 000, 5000, 3000 and 8000 cells/well, respectively. Cells were cultured for 4 days at 37°C with 5% CO_2_ in an IncucyteS3 live‐cell imaging system. Based on the in silico drug prediction results, promising compounds were selected, and cells were treated with a range of concentrations (Afatinib: 2.5, 5, 7.5, 10 µM; Gefitinib: 5, 10, 15, 20 µM; Neratinib and Erdafitinib: 1, 4, 7, 10 µM; Cobimetinib and Trametinib:.01,.1, 1, 10 µM; Cisplatin: 2, 4, 6, 8, 10 µM; Gemcitabine: 1, 4, 7, 10 nM; Decitabine:.5, 1, 5, 10 µM; 1S,3R‐RSL‐3: 1, 10, 100, 1000 nM). Confluence was determined after 96 h using the Incucyte® Base Analysis Software with integrated AI‐driven analysis tools.

### 2.12 RNA‐seq

Total RNA was isolated from the three cell line cluster models; UMUC3, 5637 and RT112, using the FavorPrep Tissue Total RNA purification Mini Kit (FavorGen BioTech Corp.) following the manufacturer's protocol. Control cells were used or cells treated for 72 h with either 600 nM neratinib, 4 µM afatinib, 2 µM trametinib or 7 µM erdafitinib. Gene expression analysis was performed using the ExpressoSeq rapid 3′ RNA‐Seq service (XPseq Analytics GmbH). Raw sequencing reads were aligned to the human genome (GENCODE Release 47, GRCh38.p14) using the Minimap2 aligner (version 2.28). Read counting and differential gene expression analysis was performed with the Rsubread (version 2.22.1) and edgeR (version 4.6.3) packages in R (version 4.5.0). Gene set enrichment analysis was performed using the Camera function with the MsigDB Hallmark gene sets (version v2024.1.Hs). Visualisation of gene set activity was performed by calculating relative activity scores with the GSVA package (version 2.2.0).

## 3 RESULTS

### 3.1 Characteristic of the analysed UC collective

For the development and validation of a novel UC molecular classifier framework, we used a large, pooled collective obtained from 4148 patients. This collective consisted of 4080 bulk cancer transcriptome datasets originating from 19 published cohorts^9^, ^12^, ^23^, ^24^, ^25^, ^26^, ^27^, ^28^, ^29^, ^30^, ^31^, ^32^, ^34^, ^35^, ^36^, ^37^, ^38^, ^39^, ^40^ as well as 422 bulk cancer proteomes originating from three publicly available cohorts.^39^, ^41^, ^42^ For a subset of the Groeneveld 2024 cohort, both bulk transcriptomes and proteomes were available.^39^ The GSE13507^26^ and GSE128959^36^ cohorts included patient‐matched transcriptome datasets of primary, recurrent and pathologically progressing UC (Tables 1 and 2, and S3 and S4). The investigated collective included NMIBC (1453 samples, 1221 patients), MIBC (2031 samples, 1935 patients) and cancers without staging information. The investigated samples (*n* = 4439) were obtained from primary tumours (95%), followed by recurrent (3.3%) and progressing (.9%) cancers and metastases (.8%). Detailed information on demographic, clinical and pathological characteristics of the analysed patients in this current study is presented in Tables 1 and 2, and S3 and S4. There were no clinically relevant differences in age distribution, race, body mass index and smoking status between clusters of patients.

Regarding the recurrence rates in the pooled NMIBC collective, as outlined in Table 1, 54 patients experienced relapse, 96 individuals had no relapse during the follow‐up, while for 1230 patients no relapse information was available. The relapse rate among NMIBC cases with the information provided was 36% within a median follow‐up of 590 days, that is, 19 months. For the pooled MIBC collective, there were 187 cases of relapses, 297 cases without relapse and 2351 individuals with unknown relapse status. This translates to a 38% recurrence rate in MIBC with a median follow‐up of 460 days, that is, 14.9 months. These data correspond well with the estimates of relapses reported in the literature.^83^, ^84^ As shown in Table 2, there were no non‐bladder samples, 1505 NMIBC bladder samples, 2669 bladder samples, 183 non‐bladder urinary organs and 20 distant metastasis samples of NMIBC. The location of the sample origin did not have any impact on UC cluster distribution (Figure S5).

### 3.2 Development of molecular UC clusters

To develop new distinct UC clusters, we used the TCGA BLCA cohort,^12^ which had the most complete clinical information and good transcriptome coverage, as the training cohort. Technical validation of the bioinformatics pipeline was performed in 17 independent bulk UC transcriptome cohorts (IMvigor, GSE13507, GSE32548, GSE48075, GSE48276, GSE83586, GSE86411, GSE87304, GSE120736, GSE124305, GSE128192, GSE128701, GSE128959, GSE198269, GSE203149, E‐MTAB‐4321, Groeneveld 2024). The full development of the bioinformatics pipeline is described in the Methods, Supporting Information and Tables S5–S10. The UC cluster performance was substantially better in cohorts with predominantly MIBC samples (e.g., TCGA BLCA, GSE48276) than in NMIBC cohorts (e.g., E‐MTAB‐4321, Figure S3 and Tables S9–S14). From the development and validation, we generated three distinct UC clusters, further referred to as Clusters #1, #2 and #3.

### 3.3 Identification of three unique molecular UC clusters in NMIBC and MIBC

**FIGURE 1:**
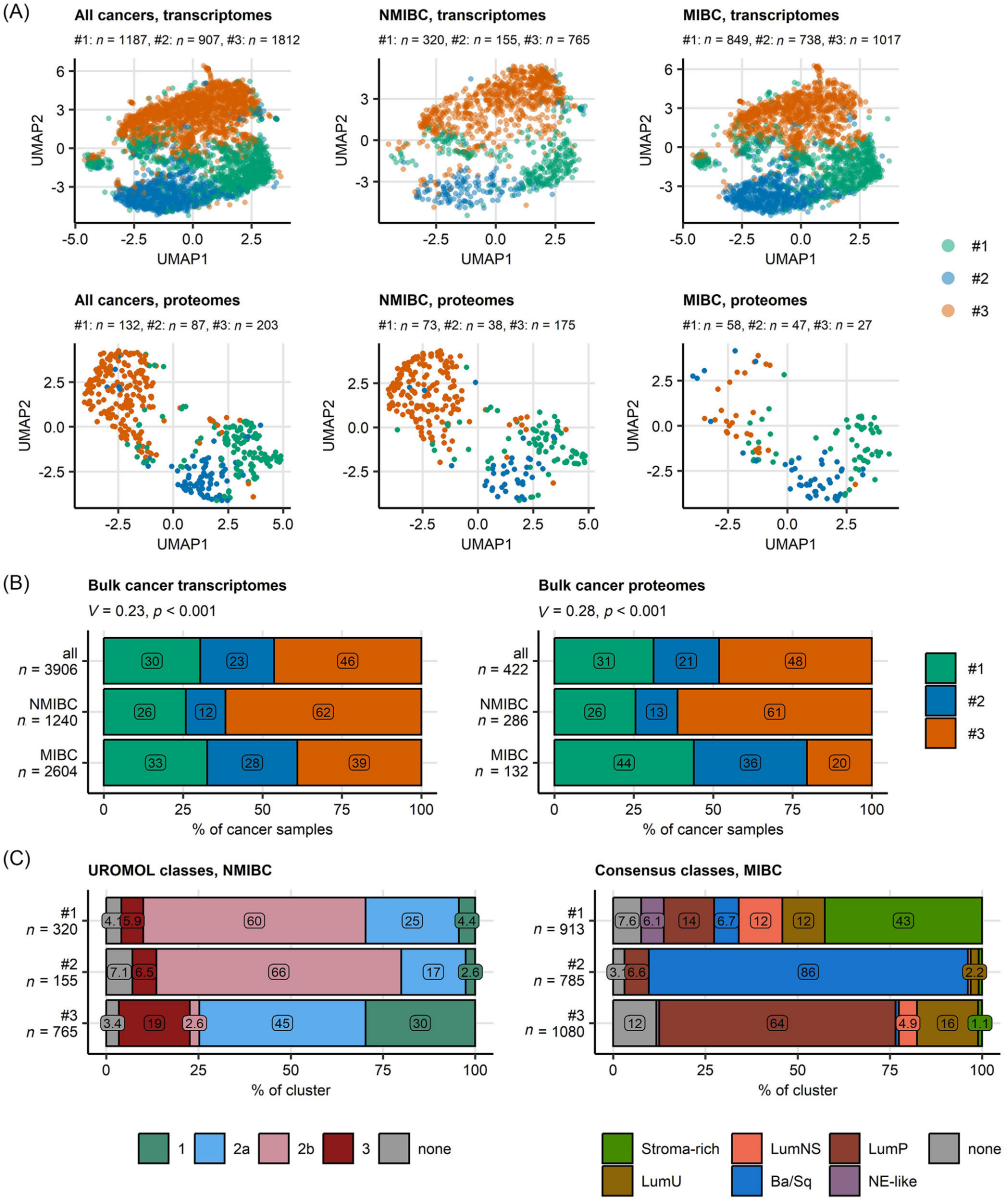
Bladder cancer clusters of bulk cancer transcriptomes and proteomes. Bladder cancer clusters were developed in the TCGA BLCA bulk cancer transcriptome cohort by regularised KMEANS clustering of metagenes defined by a self‐organising map. Bulk cancer samples in other transcriptomic cohorts were assigned to bladder cancer clusters by a k‐nearest neighbour classifier trained in the TCGA BLCA cohort. Bulk cancer transcriptomes in the BCAN cohort and bulk cancer proteome samples were assigned to bladder cancer clusters by Elastic Net classifiers trained in the TCGA BLCA cohort. Predictions of NMIBC UROMOL and MIBC consensus classes for, respectively, non‐muscle invasive bladder cancer (NMIBC) and muscle invasive bladder cancer (MIBC) bulk cancer transcriptome samples were made by nearest centroid classifiers (R packages classifyNMIBC and consensusMIBC) fed with ComBat batch effect‐adjusted log_2 mRNA expression levels of 12 394 genes measured in all collectives. (A) Visualisation of bladder cancer clusters via dimensionality reduction of mRNA and protein expression data. UMAP (uniform manifold approximation and projection) embeddings of scores of cluster‐defining metagenes (256 metagenes), and ComBat‐adjusted log_2 expression levels of proteins used by the Elastic Net classifier of bladder cancer cluster assignment (141 protein) were computed. The embeddings for all available bulk cancer samples, NMIBC and MIBC specimens are visualised as scatter plots. Each point represents a single cancer sample. Point colour codes for cluster assignment. Numbers of samples in bladder cancer clusters are displayed in the plot captions. (B) Size of bladder cancer clusters in all, NMIBC and MIBC cancers. Differences of sizes of bladder cancer clusters between NMIBC and MIBC collectives were assessed by *χ* ^2^ test with Cramer's *V* effect size statistic. Percentages of samples assigned to bladder cancer clusters in all, NMIBC and MIBC bulk transcriptome and bulk proteome collectives are depicted in stack plots. Effect sizes and *p* values are displayed in the plot captions. Numbers of samples are indicated in the *Y* axes. (C) Assignment of bulk cancer transcriptomes to bladder cancer clusters, NMIBC UROMOL classes and MIBC consensus classes. Percentages of NMIBC and MIBC samples in, respectively, NMIBC UROMOL classes and MIBC consensus classes within bladder cancer clusters are presented in stack plots. Numbers of samples in bladder cancer clusters are indicated in the *Y* axes. Ba/Sq, basal/squamous‐like; LumNS, luminal non‐specified; LumP, luminal papillary; LumU, luminal unstable; NE‐like, neuroendocrine‐like; none, not assigned. Data sources for bulk transcriptome samples: TCGA BLCA, IMvigor, GSE13507, GSE32548, GSE48075, GSE48276, GSE83586, GSE86411, GSE87304, GSE120736, GSE124305, GSE128192, GSE128701, GSE128959, GSE198269, GSE203149, E‐MTAB‐4321 and Groeneveld 2024 cohorts. Data sources for bulk proteome samples: Groeneveld 2024, Dressler 2024 and Stroggilos 2020 cohorts.

After the development of the three new clusters, their separation was compared with the current consensus classes. Most samples in the pooled collectives of bulk UC transcriptomes and bulk UC proteomes were assigned to Cluster #3 (transcriptome: 46%, proteome: 48%), followed by Cluster #1 (transcriptome: 30%, proteome: 31%) and Cluster #2. The proportion of Cluster #3 was significantly higher in NMIBC (transcriptome: 63%, proteome: 61%) than MIBC (transcriptome: 30%, proteome: 20%, Figures 1A,B and S4).

Next, we investigated how the novel UC clusters relate to UROMOL classes of NMIBC and consensus classes of MIBC (Figures 1C and S33 and S34 and Tables S15 and S63–S65). In NMIBC, UC clusters #1 and #2 consisted primarily of UROMOL class 2b (Cluster #1: 59%, Cluster #2: 61%), where UC Cluster #3 mainly contained UROMOL classes 2a (44%), 1 (30%) and 3 (20%). In MIBC, UC Cluster #1 consisted of stroma‐rich (42%), luminal non‐specified (13%), luminal unstable (12%) and luminal papillary (12%) consensus classes. UC Cluster #2 consisted of 86% basal/squamous tumours. Cluster #3 mainly included predominantly luminal–papillary tumours (63%) and luminal unstable tumours (17%). Due to the very low frequency of neuroendocrine‐like MIBC, we were not able to find substantial overlaps of differentially regulated genes with our bladder cancer clusters. To overcome this limitation, we compared gene and protein expression of published markers of neuroendocrine‐like differentiation between bladder cancer clusters in bulk cancer transcriptomes, proteomes and DepMap cell lines. As presented in Figure S35, high expression of these genes and proteins was detected predominantly in Clusters #1 and #2. In summary, Clusters #1 and #2 contained the more aggressive UC, while Cluster #3 contained the least aggressive tumours.

To compare our clusters with pan‐cancer studies, we used PAM50 signatures proposed by Parker et al., for mammary carcinoma^85^ and later applied to multiple epithelial entities. Zhao et al. used the PAM50 signature to classify cancers in the TCGA pan‐carcinoma collective as luminal A, luminal B and basal neoplasms.^86^ We extracted the PAM50 subset assignment data from the genuine publication by Zhao et al., and compared percentages of samples in PAM50 molecular subsets in bladder cancer clusters. As demonstrated in Figure S36 and Tables S66 and S67, 85% of specimens in bladder Cluster #2 were classified by Zhao et al., as PAM50 basal cancers. This underlines the very distinct basal/squamous phenotype of Cluster #2 cancers which was reproducible in both the PAM50 and MIBC consensus classification systems. In turn, roughly half of cancers in bladder Clusters #1 and #3 were assigned to the luminal A PAM50 subset. This may indicate that the pan‐carcinoma PAM50 classification system is not able to capture the diversity of luminal urothelial cancers. A reason for this might be fact that the PAM50 gene signature has only a few genes coding for stromal and ECM proteins, which were crucial for the distinction between stroma‐rich bladder Cluster #1 and ECM/immune‐desert bladder cancer Cluster #3.

### 3.4 Distinct clinical, pathological and prognostic characteristics of the UC clusters

**FIGURE 2:**
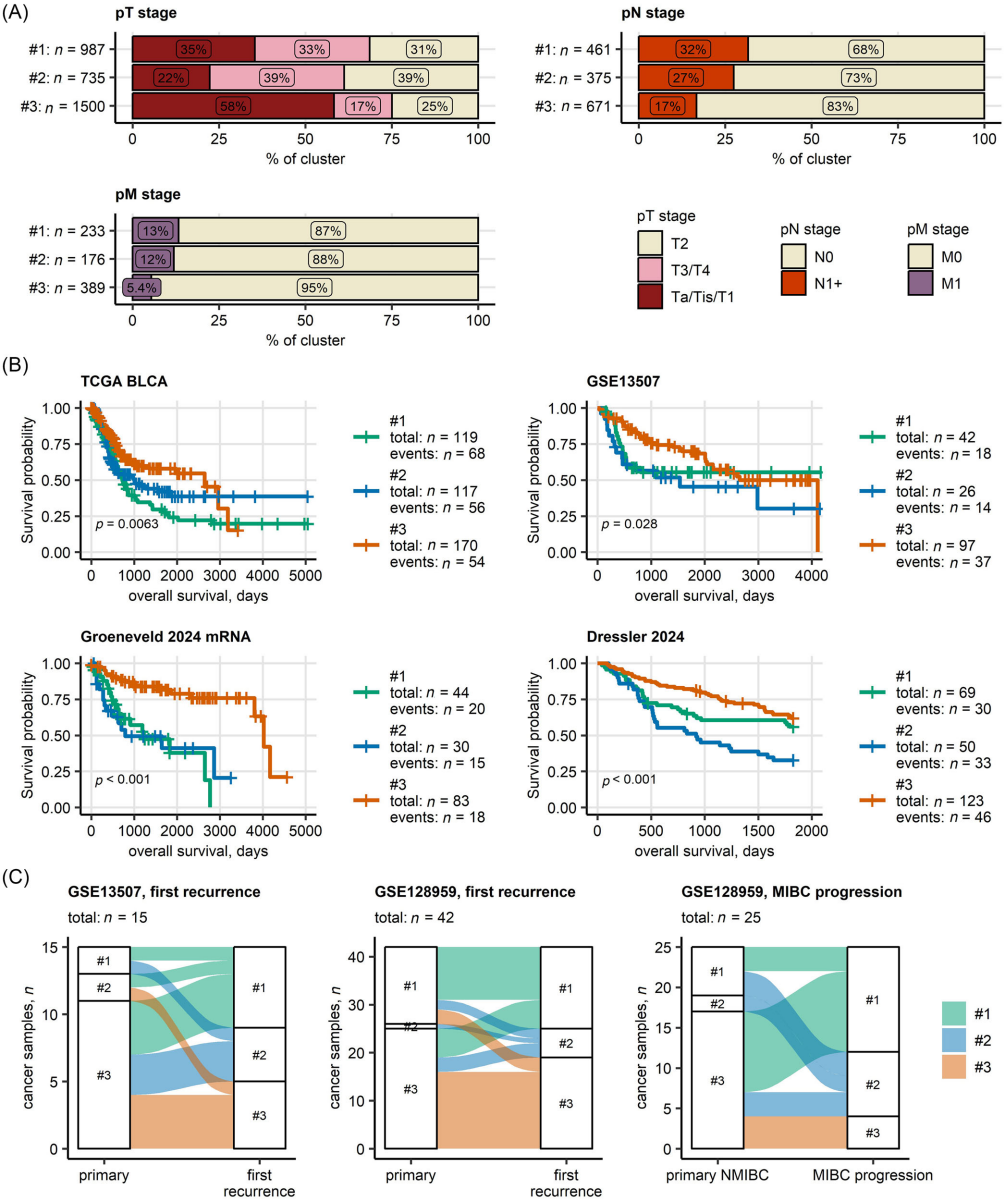
Pathological staging and overall survival in bladder cancer clusters. Evolution of bladder cancer clusters during recurrence and progression. (A) Pathological stages of tumour (pT), lymph nodes (pN) and metastases (pM) in bladder cancer clusters of urothelial cancers. Percentages of the stages within bladder cancer clusters in the pooled collective of all bulk transcriptome and bulk proteome specimens are presented in stack plots. Numbers of samples in bladder cancer clusters are indicated in the *Y* axes. Data sources, bulk cancer transcriptomes: TCGA BLCA, IMvigor, GSE13507, GSE32548, GSE48075, GSE48276, GSE83586, GSE86411, GSE87304, GSE120736, GSE124305, GSE128192, GSE128701, GSE128959, GSE198269, GSE203149, E‐MTAB‐4321, Groeneveld 2024 mRNA and BCAN cohorts. Data sources, bulk cancer proteomes: Groeneveld 2024 protein, Dressler 2024 and Stroggilos 2020 cohorts. (B) Overall survival was compared between bladder cancer clusters of transcriptomic and proteomic cohorts with at least 80 patients with complete survival information (transcriptomic cohorts: TCGA BLCA, IMvigor, GSE13507 and Groeneveld 2024; proteomic cohorts: Dressler 2024). Statistical significance of global differences in overall survival between bladder cancer clusters was determined by Peto–Peto test. *p* values were corrected for multiple testing with the false discovery rate (FDR) method. Fractions of surviving patients are presented in Kaplan–Meier plots for collectives, where significant global differences in survival between bladder cancer clusters were observed (pFDR < .05). FDR‐corrected *p* values are displayed in the plots. Numbers of observations and deaths in bladder cancer clusters are indicated in the plot legends. (C) Bulk mRNA sequencing results for patient‐matched primary and recurrent cancers, and patient‐matched primary non‐muscle invasive (NMIBC) and muscle invasive bladder cancer (MIBC) progression specimens were available for the GSE13507 and GSE128959 cohorts. Changes of bladder cluster assignment between patient‐matched primary cancer and first recurrence specimens, and between patient‐matched primary NMIBC and MIBC progression samples are visualised in alluvial plots. Total numbers of samples are displayed in the plot captions.

To dive deeper into the features of the clusters, we investigated their distinct clinical and pathologic characteristics (Tables 3 and S16–S19, and Figures 2A and S10). There were no clinically relevant differences in age distribution and smoking status between clusters of patients. Clusters #1 and #2 were enriched with T2 (pooled collective, Cluster #1: 31%, 2: 39%, 3: 25%) and T3/T4 stages (pooled collective, Cluster #1: 33%, 2: 39%, 3: 17%). Cluster #3 consisted predominantly of Ta/T1/Tis stages. Rates of lymph node (N1+) and distant metastases (pM1) were higher in Cluster #1 (pooled collective, pN1+: 32%, pM1: 13%) than in Clusters #2 (pooled collective, pN1+: 27%, M1: 12%) and #3 (pooled collective, N1+: 17%, M1: 5.4%). Similarly, frequencies of high histological grade cancers were higher in Clusters #1 (pooled collective: 65%) and #2 (80%) than in Cluster #3 (53%). In line with the aggressive pathologic phenotype of Clusters #1 and #2, cystectomy rates were higher in Clusters #1 (pooled collective: 34%) and #2 (38%) than in Cluster #3 (14%).

**TABLE 3:** Demographic, clinical and pathological characteristic of bladder cancer clusters in a pooled collective of urothelial cancers.

Variable^a^	#1^b^	#2^c^	#3^d^	Significance^e^	Effect size^e^
Samples, *N*	1383	1041	2078		
Age, years	68 [IQR: 60–75]	69 [IQR: 61–76]	66 [IQR: 58–74]	*p* =.0014	*η* ^2^ =.0061
	Range: 22–96	Range: 35–94	Range: 24–90		
	Complete: *n* = 638	Complete: *n* = 493	Complete: *n* = 898		
Sex	Female: 11% (148)	Female: 14% (141)	Female: 9.7% (201)	*p* =.0015	*V* =.079
	Male: 42% (575)	Male: 35% (366)	Male: 40% (823)		
	Unknown: 48% (660)	Unknown: 51% (534)	Unknown: 51% (1054)		
Race	Asian:.43% (6)	Asian:.58% (6)	Asian: 2.1% (43)	*p* <.001	*V* =.11
	Black or African American:.87% (12)	Black or African American: 1.5% (16)	Black or African American:.91% (19)		
	White: 19% (261)	White: 19% (202)	White: 13% (262)		
	Other:.87% (12)	Other:.67% (7)	Other:.58% (12)		
	Unknown: 79% (1092)	Unknown: 78% (810)	Unknown: 84% (1742)		
Smoking history	Never: 5.1% (71)	Never: 5.9% (61)	Never: 4.7% (98)	ns (*p* =.8)	*V* =.027
	Current or previous: 13% (185)	Current or previous: 15% (152)	Current or previous: 11% (221)		
	Unknown: 81% (1127)	Unknown: 80% (828)	Unknown: 85% (1759)		
Cancer tissue type	Bladder: 96% (1322)	Bladder: 95% (992)	Bladder: 96% (1985)	ns (*p* =.85)	*V* =.0087
	Urinary organ, non‐bladder: 4% (55)	Urinary organ, non‐bladder: 4% (42)	Urinary organ, non‐bladder: 4.1% (86)		
	Non‐urinary organ, distant metastasis:.43% (6)	Non‐urinary organ, distant metastasis:.67% (7)	Non‐urinary organ, distant metastasis:.34% (7)		
Pathology grade	G1:.87% (12)	G1:.29% (3)	G1: 2.2% (46)	*p* <.001	*V* =.14
	G2: 1.7% (24)	G2: 1.3% (14)	G2: 4.9% (101)		
	G3: 13% (176)	G3: 12% (122)	G3: 10% (213)		
	Unknown: 85% (1171)	Unknown: 87% (902)	Unknown: 83% (1718)		
Histology grade	Low grade: 8.5% (118)	Low grade: 4.8% (50)	Low grade: 13% (275)	*p* <.001	*V* =.22
	High grade: 16% (224)	High grade: 19% (200)	High grade: 15% (306)		
	Unknown: 75% (1041)	Unknown: 76% (791)	Unknown: 72% (1497)		
Invasiveness	Non‐muscle invasive: 28% (393)	Non‐muscle invasive: 19% (193)	Non‐muscle invasive: 45% (940)	*p* <.001	*V* =.24
	Muscle invasive: 70% (971)	Muscle invasive: 80% (832)	Muscle invasive: 53% (1107)		
	Unknown: 1.4% (19)	Unknown: 1.5% (16)	Unknown: 1.5% (31)		
pT stage	T2: 22% (310)	T2: 27% (285)	T2: 18% (374)	*p* <.001	*V* =.16
	T3: 16% (224)	T3: 20% (205)	T3: 8.9% (184)		
	T4: 6.4% (89)	T4: 7% (73)	T4: 3.1% (65)		
	Ta/Tis/T1: 25% (349)	Ta/Tis/T1: 16% (164)	Ta/Tis/T1: 42% (875)		
	T3/T4: 1.1% (15)	T3/T4:.77% (8)	T3/T4:.096% (2)		
	Unknown: 29% (396)	Unknown: 29% (306)	Unknown: 28% (578)		
pN stage	N0: 23% (315)	N0: 26% (272)	N0: 27% (559)	*p* <.001	*V* =.16
	N1+: 11% (146)	N1+: 9.9% (103)	N1+: 5.4% (112)		
	Unknown: 67% (922)	Unknown: 64% (666)	Unknown: 68% (1407)		
pM stage	M0: 15% (202)	M0: 15% (155)	M0: 18% (368)	*p* =.0029	*V* =.12
	M1: 2.2% (31)	M1: 2% (21)	M1: 1% (21)		
	Unknown: 83% (1150)	Unknown: 83% (865)	Unknown: 81% (1689)		
Neoadjuvant systemic chemotherapy	No: 19% (269)	No: 25% (261)	No: 18% (373)	ns (*p* =.51)	*V* =.036
	Yes: 8.2% (113)	Yes: 9% (94)	Yes: 6.2% (129)		
	Unknown: 72% (1001)	Unknown: 66% (686)	Unknown: 76% (1576)		
History of BCG therapy	No: 18% (253)	No: 18% (187)	No: 18% (374)	ns (*p* =.41)	*V* =.047
	Yes: 4.4% (61)	Yes: 3.1% (32)	Yes: 4.2% (87)		
	Unknown: 77% (1069)	Unknown: 79% (822)	Unknown: 78% (1617)		
Cystectomy	No: 14% (192)	No: 10% (108)	No: 17% (352)	*p* <.001	*V* =.25
	Yes: 10% (143)	Yes: 11% (111)	Yes: 5% (103)		
	Unknown: 76% (1048)	Unknown: 79% (822)	Unknown: 78% (1623)		
Adjuvant systemic chemotherapy	No: 9.9% (137)	No: 13% (134)	No: 12% (255)	*p* =.002	*V* =.099
	Yes: 21% (295)	Yes: 19% (200)	Yes: 16% (340)		
	Unknown: 69% (951)	Unknown: 68% (707)	Unknown: 71% (1483)		

Qualitative variables are presented as percentages and counts of complete observations in a cluster. Quantitative variables are shown as medians with interquartile ranges and ranges. Statistical significance of differences between bladder cancer clusters was determined by *χ* ^2^ test (qualitative variables) or Kruskal–Wallis test. Cramer's *V* and *η* ^2^ served as effect size statistics. *p* values were corrected for multiple testing with the false discovery rate method.

^a^ BCG, Bacillus Calmette–Guerin; pM stage, pathological metastasis stage; pN stage, pathological lymph node stage; pT stage, pathological tumour stage.

^b^ BCAN: *n* = 64, Dressler 2024: *n* = 69, E‐MTAB‐4321: *n* = 153, Groeneveld 2024: *n* = 60, NA: *n* = 23, GSE120736: *n* = 36, GSE124305: *n* = 44, GSE128192: *n* = 34, GSE128701: *n* = 50, GSE128959: *n* = 75, GSE13507: *n* = 51, GSE198269: *n* = 131, GSE203149: *n* = 52, GSE32548: *n* = 34, GSE48075: *n* = 34, GSE48276: *n* = 36, GSE83586: *n* = 82, GSE86411: *n* = 41, GSE87304: *n* = 79, IMvigor: *n* = 75, Stroggilos 2020: *n* = 40, TCGA BLCA: *n* = 120.

^c^ BCAN: *n* = 47, Dressler 2024: *n* = 50, E‐MTAB‐4321: *n* = 81, Groeneveld 2024: *n* = 37, NA: *n* = 18, GSE120736: *n* = 28, GSE124305: *n* = 44, GSE128192: *n* = 37, GSE128701: *n* = 34, GSE128959: *n* = 24, GSE13507: *n* = 31, GSE198269: *n* = 88, GSE203149: *n* = 34, GSE32548: *n* = 23, GSE48075: *n* = 35, GSE48276: *n* = 33, GSE83586: *n* = 85, GSE86411: *n* = 28, GSE87304: *n* = 92, IMvigor: *n* = 56, Stroggilos 2020: *n* = 19, TCGA BLCA: *n* = 117.

^d^ BCAN: *n* = 63, Dressler 2024: *n* = 123, E‐MTAB‐4321: *n* = 242, Groeneveld 2024: *n* = 101, NA: *n* = 22, GSE120736: *n* = 81, GSE124305: *n* = 45, GSE128192: *n* = 41, GSE128701: *n* = 52, GSE128959: *n* = 93, GSE13507: *n* = 106, GSE198269: *n* = 175, GSE203149: *n* = 85, GSE32548: *n* = 74, GSE48075: *n* = 73, GSE48276: *n* = 47, GSE83586: *n* = 140, GSE86411: *n* = 63, GSE87304: *n* = 134, IMvigor: *n* = 90, Stroggilos 2020: *n* = 58, TCGA BLCA: *n* = 170.

^e^Comparison of bladder cancer clusters, the ‘unknown’ category was excluded.

Next, significant differences in overall survival between UC clusters were investigated by Kaplan–Meier analysis and Cox proportional hazard modelling as shown in Figure 2B. Cluster #1 (median overall survival [OS], TCGA BLCA: 24 months; vs. Cluster #3: HR = 1.8, 95% CI, 1.3–2.6) and Cluster #2 (median OS, TCGA BLCA: 33 months; HR = 1.5, 95% CI, 1.0–2.2) had shorter OS and a higher mortality risk versus Cluster #3 (median OS, TCGA BLCA: 87 months). Similar significant differences in OS between UC clusters were observed for NMIBC (Dressler 2024 cohort), MIBC (TCGA BLCA, Dressler 2024) and Ta–T3 UC (TCGA BLCA, Dressler 2024, Groeneveld 2024). Tumour‐specific survival was significantly poorer in Clusters #1 and #2 versus Cluster #3 in both the TCGA BLCA and GSE13507 cohorts. The risk of recurrence was also significantly higher in Clusters #1 and #2 versus Cluster #3 in the TCGA BLCA cohort. In the E‐MTAB‐4321 cohort, Clusters #1 (HR = 2.7, 95% CI, 1.1–6.6) and #2 (HR = 4.3, 95% CI, 1.7–11) had a significantly higher NMIBC–MIBC progression risk versus Cluster #3 (Figures 2B and S11–S13, and Table S20). Collectively, the clinical, pathological and survival characteristics of the UC clusters point to a more aggressive, high‐risk phenotype of Clusters #1 and #2.

**FIGURE 3:**
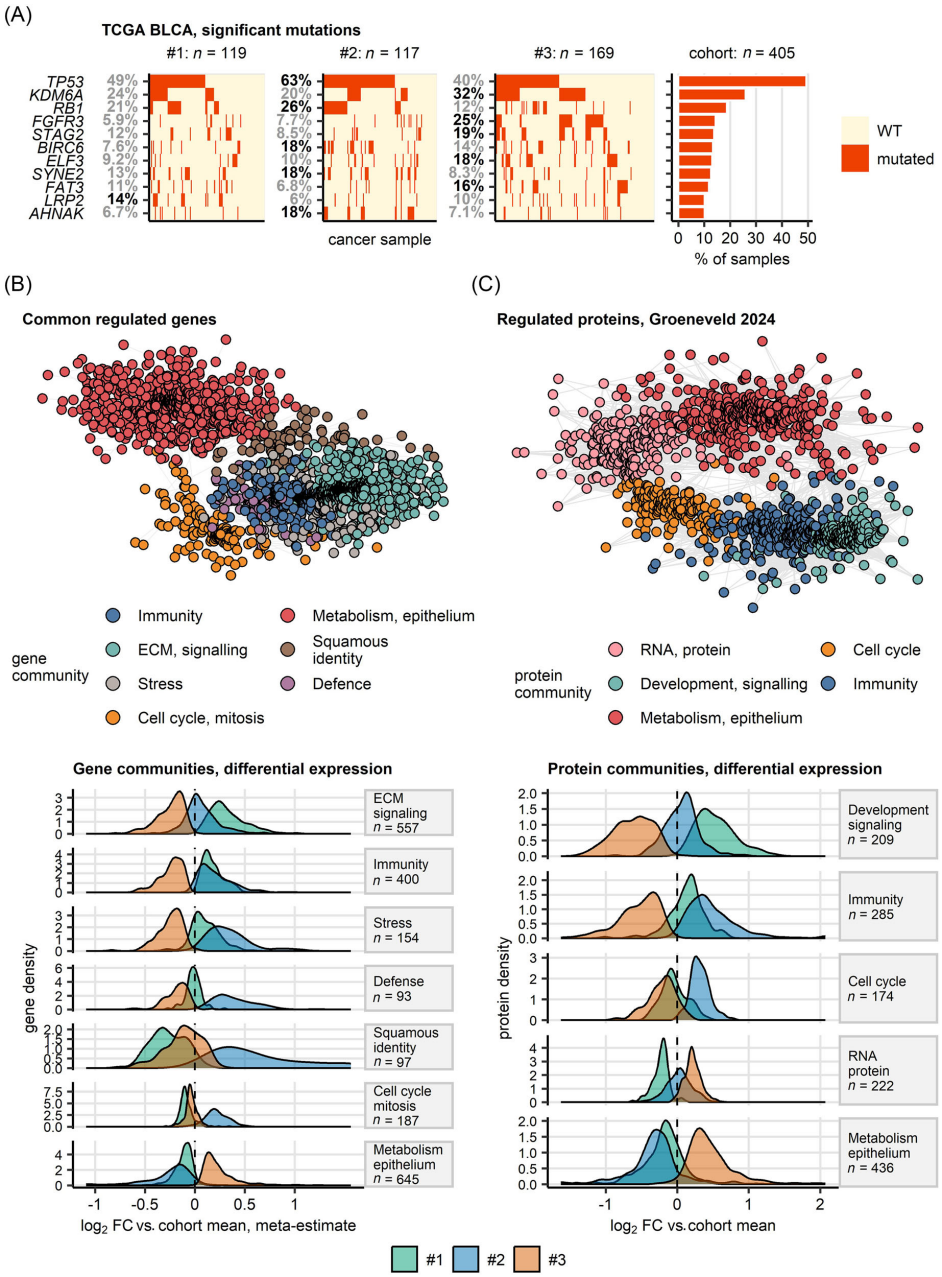
Genetics, transcriptome and proteome of bladder cancer clusters. (A) Enrichment analysis of somatic mutation in bladder cancer clusters. Enrichment of somatic mutations among bulk cancer samples assigned to bladder cancer clusters in TCGA BLCA, IMvigor and BCAN cohorts was assessed by permutation tests. Mutation status of genes with significant enrichment (raw *p* < .05 without multiple testing correction) in at least one of the clusters and overall mutation rate ≥ 10% in the TCGA BLCA cohort is shown in oncoplots. Each oncoplot tile represents a cancer sample. Percentages of samples with mutations within the clusters are indicated in the *Y* axes of the oncoplots; significant enrichment is highlighted by bold font. The overall mutation percentages are presented in the bar plot. Numbers of specimens in bladder cancer cluster and in the entire cohort are displayed above the plots. (B, C) Co‐regulation networks of genes (B) and proteins (C) differentially expressed in bladder cancer clusters as compared with the cohort means. log_2‐transformed gene and protein expression levels (no ComBat adjustment) were compared between bladder cancer clusters in transcriptomic and proteomic bulk cancer cohorts by one‐way ANOVA with *η* ^2^ effect size statistic. Differences between mean log_2 expression levels in the clusters and the respective cohort means were evaluated by one‐sample *T*‐test. *p* values were corrected for multiple comparisons with the false discovery rate (FDR) method. Significantly differentially regulated genes and proteins were defined by ANOVA pFDR <.05, *η* ^2^ ≥.06 and *T*‐test pFDR <.05. Meta‐estimates of log_2 fold differential gene expression for features differentially regulated between the clusters in at least 10 cohorts were computed with the DerSimonian–Lair inverse variance method. Co‐expression patterns of the differentially regulated genes shared by at least 10 out of 19 transcriptomic cohorts and of the differentially regulated proteins in proteomic collectives were investigated by network analysis. The co‐regulation transcriptomic network was built in a pooled dataset of ComBat‐adjusted gene expression values (total samples: *n* = 3906; sample sources TCGA BLCA: *n* = 407, IMvigor: *n* = 221, GSE13507: *n* = 188, GSE32548: *n* = 131, GSE48075: *n* = 142, GSE48276: *n* = 116, GSE83586: *n* = 307, GSE86411: *n* = 132, GSE87304: *n* = 305, GSE120736: *n* = 145, GSE124305: *n* = 133, GSE128192: *n* = 112, GSE128701: *n* = 136, GSE128959: *n* = 192, GSE198269: *n* = 394, GSE203149: *n* = 171, E‐MTAB‐4321: *n* = 476, and Groeneveld 2024: *n* = 198). The proteomic co‐regulation networks were built separately for each proteomic cohort (sample numbers: Groeneveld 2024: *n* = 63, Dressler 2024: *n* = 242 and Stroggilos 2020: *n* = 117). The networks’ edges were defined by pairwise correlations of gene or protein levels with Spearman's *ρ* ≥.5 and weighted by the corresponding *ρ* values. Isolated vertices, that is, genes or proteins without correlation partners were removed. Communities of co‐regulated genes and proteins were identified by the Leiden algorithm and named after their key biological processes revealed by biological process gene ontology enrichment. The co‐expression networks were visualised as force‐directed graphs with the Fruchterman–Reingold algorithm (B: pooled transcriptome cohort; C: Groeneveld 2024 proteome cohort). Single genes are represented by points, correlations between genes with *ρ* ≥.5 are depicted as lines. Point colour codes for the community assignment. Density of meta‐estimates (B) and estimates (C) of log_2 fold changes of expression in the clusters as compared with the cohort mean for members of gene and protein communities are presented in density plots; numbers of genes and proteins in the communities are indicated in the plot facets.

As demonstrated in Figure S12, inclusion of bladder cancer clusters in the models with clinical predictors did not improve the accuracy (Harrell's *C*‐index) or confidence (Integrated Brier score) in any of the cohorts except for the Dressler 2024 collective. Accordingly, as shown in Figure S13, in the models of overall survival in the TCGA, GSE13507 and Groeneveld 2024 mRNA cohorts adjusted for clinical/pathological confounders, bladder cancer clusters were not significant predictors of overall survival. Our bladder cancer clusters are therefore not independent prognostic factors for overall survival. We could show that there are very consistent differences in pathological staging between bladder cancer clusters, with the high‐risk clusters #1 and #2 being enriched with high stage cancers (Figures 3 and S10). Furthermore, our clustering algorithm was blinded to any survival information, and hence unlikely to deliver a strong and independent risk stratification scheme.

In the GSE13507 and GSE128959 cohorts, patient‐matched samples of primary, recurrent and progressing tumours were available. During the first recurrence and pathologic progression, a fraction of Cluster #3 malignancies was reclassified as Clusters #1 and #2 (Figures 2C and S14). This suggests that a number of cancers in the low‐risk Cluster #3 may evolve into the more aggressive Clusters #1 and #2 during UC development.

### 3.5 Biological and genetic differences between the three UC clusters

In addition, we analysed the distinct biological and genetic differences between our three new UC clusters. To identify genes, which can be used as cluster markers applicable to both mRNA and protein data, we used differential gene and protein expression and receiver‐operating characteristic analysis in UC clusters of bulk UC transcriptomes and bulk proteomes (area under the curve [AUC] ≥.714).^87^ A total of 85 genes were proposed as universal markers of UC clusters shared between the transcriptome and proteome data, including *MYH11* and *COL14A1* for Cluster #1, *STAT1* and *S100A8* for Cluster #2 and *SSH3* and *S100P* for Cluster #3 markers. The marker set shared between the bulk UC transcriptome and UC cell lines encompassed 216 genes, including *FGFR1* and *COL6A2* for Cluster #1, *IFI44* and *FGFBP1* for Cluster #2 and *SRC* and *ERBB3* for Cluster #3 (Figures S7–S9).

Genome‐wide information on somatic mutations was available for the TCGA BLCA, IMvigor and BCAN, and copy number variant data were provided for the TCGA BLCA and Groeneveld 2024 cohorts. In these cohorts, no consistent differences shared by more than one cohort were observed for overall numbers of genetic alterations (Figure S15 and Table S21). As investigated by permutation testing, mutations of *TP53* were significantly more frequent in Cluster #2 (TCGA BLCA: 63%, IMvigor: 68%) than in Clusters #1 (TCGA BLCA: 49%, IMvigor: 51%) and #3 (TCGA BLCA: 40%, IMvigor: 51%). Mutations of *RB1*, *KMT2D* and *PIK3CA* were also enriched in Cluster #2. Mutations of *FGFR3* were significantly enriched in Cluster #3 (TCGA BLCA: 25%, IMvigor: 35%, BCAN: 27%) versus Clusters #1 (TCGA BLCA: 5.9%, IMvigor: 7%, BCAN: 15%) and #2 (TCGA BLCA: 7.7%, IMvigor: 9.1%, BCAN: 8.5%; Figures 3A and S15–S17, and Tables S21 and S22).

Furthermore, in an analysis of longitudinal, patient‐matched transcriptomes from primary, recurrent and progressing tumours, our UC clusters displayed distinct and highly reproducible patterns of transcriptome and proteome regulation. This translated to 4361 differentially regulated genes shared by at least 10 out of 19 analysed bulk UC transcriptome cohorts and 886 differentially regulated proteins shared by at least two out of three investigated bulk UC proteome cohorts (Figures 3B,C and S18–S20, and Tables S23–S30 and S61–S62). Such data were supported by a gene set variation analysis of Reactome Pathways in UC clusters, which returned 1041 differentially regulated signatures shared by at least 10 bulk UC transcriptome cohorts and 661 differentially regulated signatures shared by at least two bulk UC proteome cohorts (total 1615 signatures, Figures S21 and S22 and Tables S31–S34). Collectively, our data indicated, among others, an activation of genes, proteins and biological processes related to immunity, extracellular matrix (ECM) signalling, collagen and proteoglycans, development signalling and G protein‐coupled receptors (GPCR) signalling in Cluster #1. In Cluster #2, the activation of genes, proteins and signatures associated, but not limited to, immunity, stress, DNA replication and damage/repair, cell cycle and mitosis, and WNT, NOTCH, RUNX, EGFR/ERBB, growth factors and MAPK signalling was observed. Specifically in Cluster #3, these analyses revealed an upregulation of epithelial metabolism, citric acid cycle, oxidative phosphorylation, fatty acid oxidation and lipid metabolism.

**FIGURE 4:**
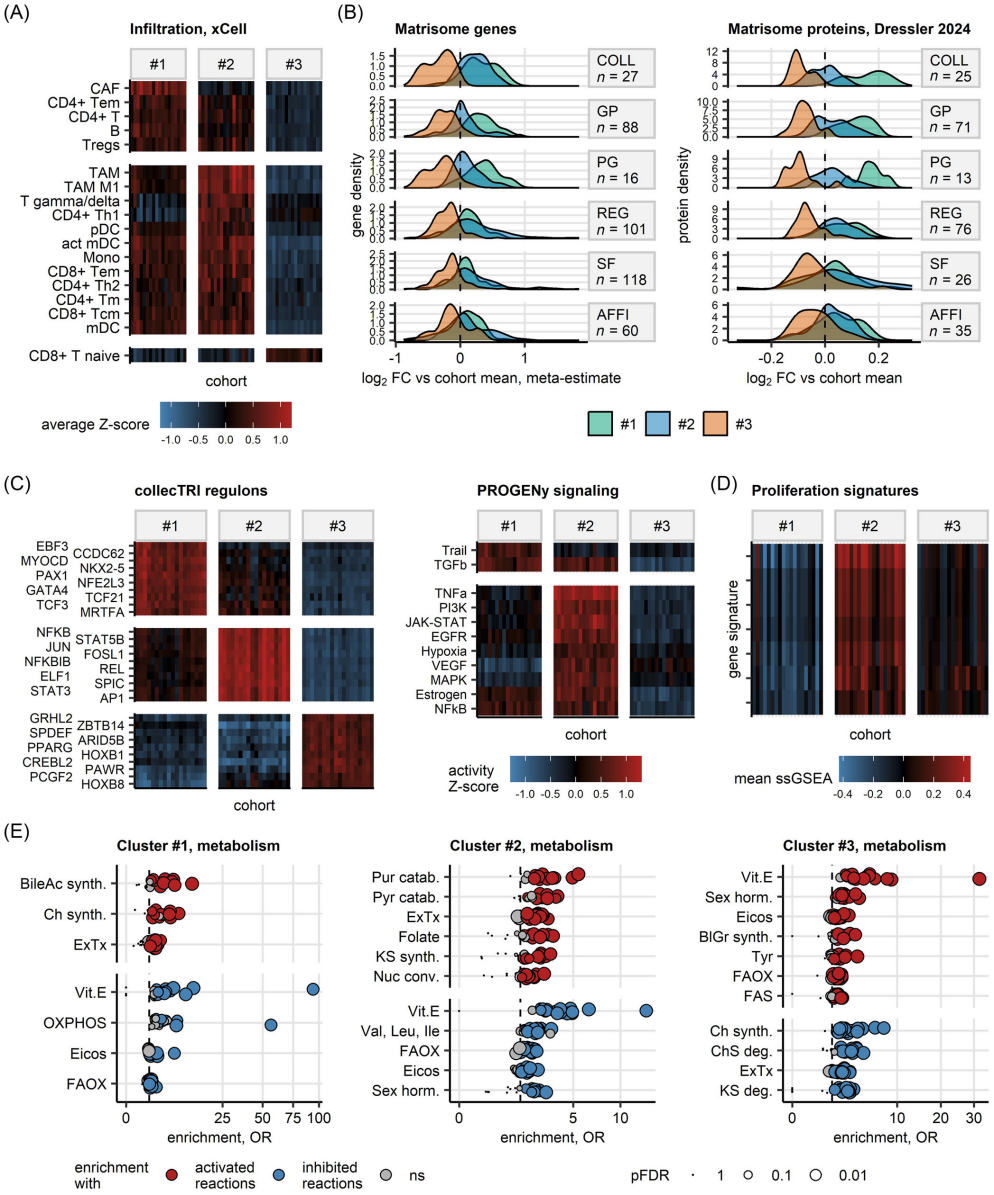
Tumour microenvironment, signalling and metabolism of bladder cancer clusters. (A) Fractions of infiltrating immune and stromal cells predicted by xCell immunedeconvolution of bulk cancer transcriptomes (*n* = 18 cohorts; TCGA BLCA, IMvigor, GSE13507, GSE32548, GSE48075, GSE48276, GSE83586, GSE86411, GSE87304, GSE120736, GSE124305, GSE128192, GSE128701, GSE128959, GSE198269, GSE203149, E‐MTAB‐4321 and Groeneveld 2024). The fractions of infiltrating cells were compared between bladder cancer clusters by Kruskal–Wallis test with *η* ^2^ effect size statistic. *p* values were corrected for multiple testing with the false discovery rate (FDR) method. Significant differences were considered for pFDR <.05 and *η* ^2^ ≥.06. Levels of infiltrating cell populations found to differ between bladder cancer clusters in at least 10 cohorts are visualised in a summary heat map. The heat map tiles represent cohorts and clusters; tile colour corresponds to average cell fraction Z‐score in the cluster and cohort. (B) Differential expression of matrisome genes and proteins in bladder cancer clusters. Differential gene and protein expression in bladder cancer clusters as compared with the cohort means was assessed by one‐way ANOVA and one‐sample post‐hoc *T*‐test as presented in B and C. Meta‐estimates of fold‐regulation. Meta‐estimates of log_2 fold differential gene expression for features differentially regulated between the clusters in at least 10 out of 19 transcriptomic cohorts (TCGA BLCA, IMvigor, GSE13507, GSE32548, GSE48075, GSE48276, GSE83586, GSE86411, GSE87304, GSE120736, GSE124305, GSE128192, GSE128701, GSE128959, GSE198269, GSE203149, E‐MTAB‐4321, Groeneveld 2024 and BCAN) were computed with the DerSimonian–Lair inverse variance method. Density of meta‐estimates and estimates of log_2 fold changes of expression in the clusters as compared with the cohort mean for significantly differentially regulated matrisome genes and proteins shared by, respectively, at least 10 and two cohorts are presented in density plots. The differentially regulated gene and proteins were classified by matrisome categories (AFFI, affiliated proteins; COLL, collagens; GP, glycoproteins; PR, proteglycans; REG, regulatory proteins; SF, secreted factors); numbers of differentially regulated genes and proteins in the matrisome categories are displayed in the plot facets. (C) Activity of transcriptional regulons and signalling pathways in bladder cancer cluster predicted with bulk cancer transcriptome data by decoupleR modelling algorithms (*n* = 18 cohorts; TCGA BLCA, IMvigor, GSE13507, GSE32548, GSE48075, GSE48276, GSE83586, GSE86411, GSE87304, GSE120736, GSE124305, GSE128192, GSE128701, GSE128959, GSE198269, GSE203149, E‐MTAB‐4321 and Groeneveld 2024). The activity linear modelling (LM) scores of regulons and signalling pathways were predicted for single bulk cancer transcriptome samples by, respectively, univariable and multivariable modelling with collecTRI and PROGENy knowledge models. LM scores were compared between bladder cancer clusters by one‐way ANOVA with *η* ^2^ effect size statistic. Differences between mean LM scores in the clusters and the respective cohort means were evaluated by one‐sample *T*‐test. *p* values were corrected for multiple comparisons with the FDR method. Significantly differentially regulated regulons and signalling pathways were defined by ANOVA pFDR <.05, *η* ^2^ ≥.06 and *T*‐test pFDR <.05. Activities of the top 10 strongest activated regulons shared by at least 10 cohorts and activities of the differentially regulated signalling pathways shared by at 10 cohorts are visualised in summary heat maps. The heat map tiles represent cohorts and clusters; tile colour corresponds to average cell fraction Z‐score in the cluster and cohort. (D) Gene signatures of cell proliferation in bladder cancer clusters. Single‐sample gene set enrichment analysis (ssGSEA) scores of seven published gene signatures of cell proliferation were compared between bladder cancer clusters in 19 transcriptomic cohorts (TCGA BLCA, IMvigor, GSE13507, GSE32548, GSE48075, GSE48276, GSE83586, GSE86411, GSE87304, GSE120736, GSE124305, GSE128192, GSE128701, GSE128959, GSE198269, GSE203149, E‐MTAB‐4321, Groeneveld 2024 and BCAN) by one‐way ANOVA with *η* ^2^ effect size statistic. *p* values were corrected for multiple testing with the FDR method. Significant differences were considered for pFDR <.05 and *η* ^2^ ≥.06. ssGSEA scores of proliferation signatures found to differ significantly between the clusters in at least 10 cohorts are visualised in a summary heat map. The heat map tiles represent cohorts and clusters; tile colour corresponds to average ssGSEA score in the cluster and cohort. (E) Metabolism of bladder cancer clusters predicted for bulk cancer transcriptome data (*n* = 19 cohorts; TCGA BLCA, IMvigor, GSE13507, GSE32548, GSE48075, GSE48276, GSE83586, GSE86411, GSE87304, GSE120736, GSE124305, GSE128192, GSE128701, GSE128959, GSE198269, GSE203149, E‐MTAB‐4321, Groeneveld 2024 and BCAN). Activity of RECON metabolic reactions was modelled by Monte Carlo simulations with tools of BiGGR and biggrExtra packages fed with log_2 fold‐regulation estimates and standard errors of differential expression for all measured genes. Enrichment of RECON metabolic subsystems with significantly activated and inhibited reactions was assessed by comparison with 10 000 random draws from the entire reaction pool. Enrichment *p* values were corrected for multiple testing with the FDR method. Odds ratio (OR) served as a metric of enrichment magnitude. Significant enrichment with activated or inhibited reactions was considered for pFDR <.05 and OR ≥ 1.44. For the significant subsystems shared by at least 10 cohorts, OR values are presented in dot plots. Each point represents a single cohort. Point colour codes for reaction activity status and significance, point size codes for FDR‐corrected *p* value (BileAc synth., bile acid synthesis; BlGr synth., blood group synthesis; Ch synth., chondroitin synthesis; ChS deg., chondroitin sulphate degradation; Eicos, eicosanoid metabolism; ExTx, extracellular transport; FAOX, fatty acid oxidation; FAS, fatty acid synthesis; KS synth., keratan sulphate synthesis; Nuc conv., nucleotide conversion; OXPHOS, oxidative phosphorylation; Pur catab., purine catabolism; Pyr catab., pyrimidine catabolism; Sex horm., sex hormone metabolism; Tyr, tyrosine metabolism; Val, Leu, Ile, valine, leucine and isoleucine metabolism; Vit.E, vitamin E).

Next, we delved into characteristics of stroma cell infiltration and ECM in our UC clusters, that is, the key components of the tumour microenvironment (TME). As quantified by xCell and QuanTIseq immune deconvolution.^54^, ^55^ Cluster #1 cancers were extensively infiltrated by cancer‐associated fibroblasts, B cells, CD4+ and regulatory T cells. High levels of dendritic cells, monocytes, inflammatory M1 tumour‐associated macrophages, Th1 CD4+ T cells, and central and effector memory CD8+ T cells were predicted for Cluster #2. In turn, for Cluster #3, an ‘immune‐desert phenotype’ was observed (Figures 4A and S23 and S24, and Table S35). Cluster #1 cancers were ECM‐rich with abundant expression of collagen, glycoprotein and proteoglycan genes and proteins.^88^ High expression of regulatory and secreted ECM proteins hallmarked Cluster #2 UC. By contrast, Cluster #3 was ECM‐scarce, suggesting an ‘ECM‐desert phenotype’ (Figures 4B and S25, and Tables S36 and S37).

To identify transcription factors driving the modulation of transcriptomes and proteomes in UC clusters and their distinct TME phenotypes, we resorted to modelling of activity of transcription factor = target gene interaction with a decoupler LM tool^60^, ^62^ fed with gene expression information from 18 UC cohorts. Using a similar LM algorithm, we assessed the activity of signalling pathways^61^, ^62^ in UC clusters of 18 UC cohorts. Full details of the analysis are provided in Figures 4C and S26 and S27, and Tables S38–S43. In general, high stroma differentiation, with main signalling pathways Trail and transforming growth factor (TGF)‐ß, and regulons like EBF3, TCF3 and PAX1, was observed for Cluster #1. A high inflammatory response for Cluster #2 was observed, with main signalling pathways TNF‐α, PI3K, JAK/STAT, MAPK and EGFR, and regulons including NFKB, AP1, JUN and STATs. Cluster #3 was characterised as signalling quiescent, but with increased metabolic switches like PPARG and CREBL2, which may drive fatty acid metabolism. Nevertheless, these are predictions derived from expression levels of target genes of transcription factors and only call for direct measurements of transcription factor activity.

In addition, differential gene and protein expression data indicated an elevated pace of cell cycle progression and mitosis in UC Cluster #2. These findings were supported by a gene set enrichment analysis: ssGSEA scores of seven published gene signatures of cell proliferation were significantly higher in Cluster #2 than in the other UC clusters (Figure 4D).

Furthermore, based on differential gene and protein expression information, we modelled activity of metabolic reactions and subsystems^77^, ^79^ in UC clusters by Monte Carlo simulations and enrichment analyses.^78^, ^89^ Complete information regarding the included metabolic reactions and their activated or inhibited enrichment in our clusters is provided in Figures 4E and S28 and Tables S44–S47.

To summarise, distinct biological and genetic differences between the three UC clusters were found. Cluster #1 is ECM and immune‐rich, Cluster #2 is highly immune‐active and Cluster #3 has an ECM‐ and immune‐desert phenotype.

### 3.6 Differential predicted anti‐cancer drug response in UC clusters using machine learning

**FIGURE 5:**
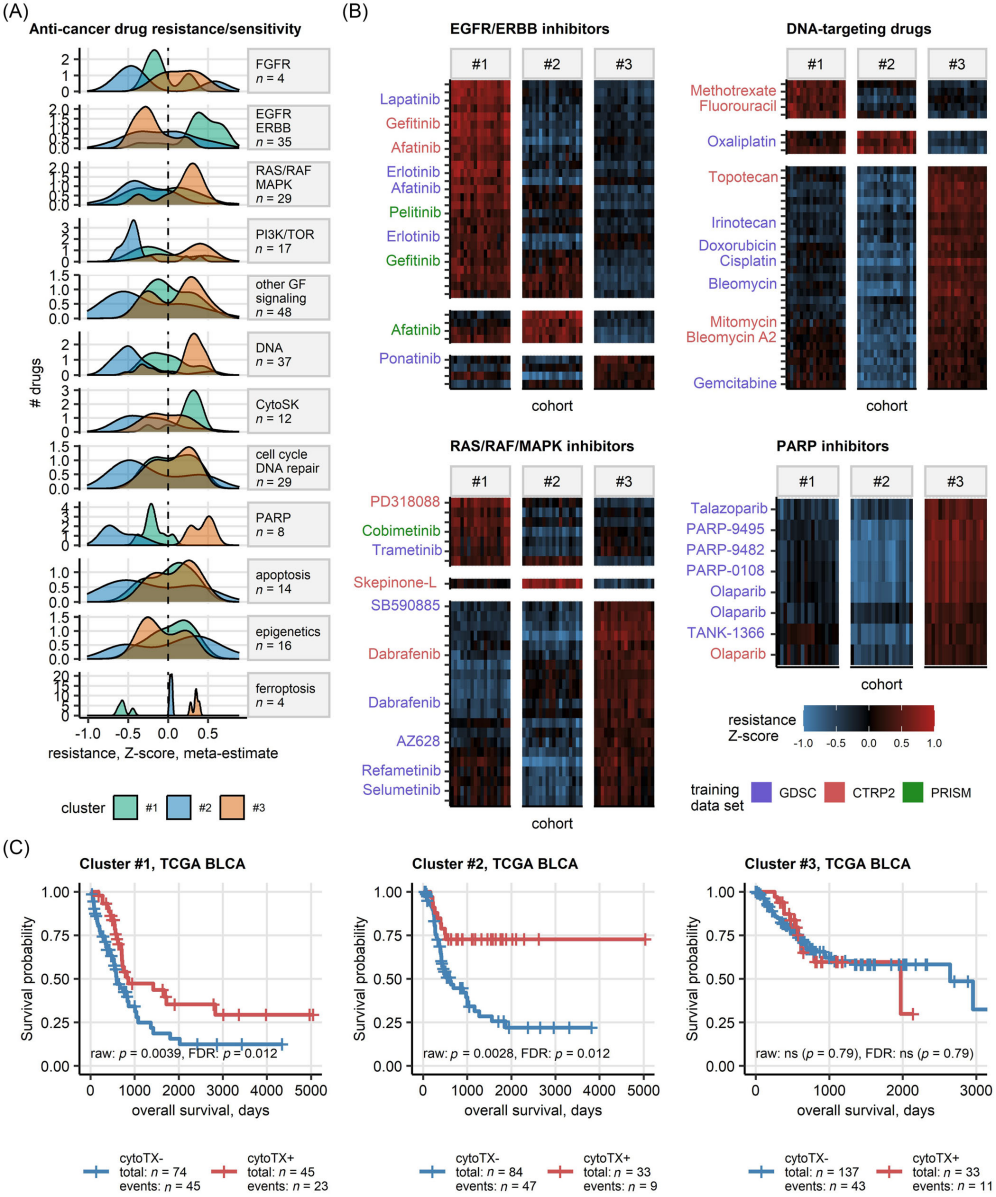
Predictions of resistance and sensitivity to anti‐cancer drugs in bladder cancer clusters. Based on whole‐transcriptome expression data, resistance to anti‐cancer drugs was predicted for bulk cancer transcriptome samples (*n* = 18 cohorts; TCGA BLCA, IMvigor, GSE13507, GSE32548, GSE48075, GSE48276, GSE83586, GSE86411, GSE87304, GSE120736, GSE124305, GSE128192, GSE128701, GSE128959, GSE198269, GSE203149, E‐MTAB‐4321 and Groeneveld 2024) by RIDGE linear models trained with anti‐cancer drug resistance data and baseline gene expression of epithelial cancer cell lines recorded in the GDSC, CTRP2 and PRISM drug screening experiments. Predicated drug resistance in form of Z‐scores of IC50 (concentration resulting 50% growth inhibition) and AUC (area under the dose–response curve) was compared between bladder cancer clusters by one‐way ANOVA with *η* ^2^ effect size statistic. Differences between mean resistance Z‐scores in the clusters and the respective cohort means were evaluated by one‐sample *T*‐test. *p* values were corrected for multiple comparisons with the false discovery rate (FDR) method. Significant differences in predicted resistance were considered for ANOVA pFDR <.05, *η* ^2^ ≥.06 and *T*‐test pFDR <.05. Meta‐estimates of differences of resistance Z‐scores the cluster and the cohort mean for compounds with significant differences in predicted resistance shared by at least 10 cohorts were computed with the DerSimonian–Lair inverse variance method. (A) Densities of meta‐estimates of resistance Z‐scores between bladder cancer clusters and the cohort mean for the compounds with significant differences in predicted resistance shared by at least 10 cohorts. The compounds were categorised by their molecular targets (CytoSK, cytoskeleton; GF, growth factors). *Note*: Density curves shifted to right are characteristic for resistance, density curves shifted to left denote predicted sensitivity. The compound numbers are displayed in the plot facets. (B) Resistance Z‐scores of compounds targeting EGFR/ERBB and RAS/RAF/MAPK signalling, DNA and PARP with significant differences between bladder cancer clusters shared by at least 10 cohorts. The resistance Z‐scores are visualised in a summary heat map. The heat map tiles represent cohorts and clusters; tile colour corresponds to average resistance Z‐score in the cluster and cohort. Names of compounds of clinical relevance are indicated in the *Y* axes of the heat maps. The compound name text colour codes for the in vitro drug screening data used for resistance prediction (blue: GDSC; red: CTRP2; green: PRISM). (C) Overall survival in bladder cancer clusters was compared between TCGA BLCA patients with systemic cytotoxic chemotherapy (cytoTX+, gemcitabine, platinum, topoisomerase inhibitors, alkylating agents, methotrexate) and without systemic cytotoxic chemotherapy (cytoTX−). Statistical significance was determined by Peto–Peto test corrected for multiple testing with the false discovery rate (FDR) method. Fractions of surviving patients are shown in Kaplan–Meier plots. Raw unadjusted *p* values and FDR‐corrected *p* values of survival differences are displayed in the plots. Total numbers of observations and deaths are displayed in the plot captions. Numbers of patients and deaths in cytotoxic treatment subsets in bladder cancer clusters are indicated in the plot legends.

To explore the potential of our novel molecular classification scheme for personalised treatment of UC, we assessed in silico anti‐cancer drug response of single samples in 18 bulk UC transcriptome cohorts.^59^, ^73^, ^90^ Subsequently, we compared the predicted response scores of 656 anti‐cancer agents between our UC clusters (Figure 5A,B and Tables S48 and S49). Cluster #1 tumours were predicted to respond to PARP inhibitors (e.g., olaparib), RAS/RAF/MAPK inhibitors (e.g., BRAF inhibitor dabrafenib), cytotoxic drugs (e.g., topoisomerase inhibitors irinotecan and topotecan) and ferroptosis inducers (e.g., erastin). Cluster #1 tumours were predicted to be resistant to EGFR/ERBB inhibitors, cytoskeleton‐targeting drugs and ERK/MEK inhibitors. For Cluster #2, in line with the activation of EGFR/ERBB/MAPK signalling, a broad sensitivity to EGFR/ERBB (e.g., gefitinib, lapatinib) and ERK/MEK inhibitors (e.g., refametinib, selumetinib, cobimetinib, trametinib) was predicted. Additionally, Cluster #2 cancers were predicted to be sensitive to PARP inhibitors, folate anti‐metabolites (methotrexate), cytoskeleton‐ (e.g., taxanes, vinblastine) and DNA‐targeting cytotoxic drugs (topoisomerase inhibitors, gemcitabine, cisplatin), which was consistent with their increased cell proliferation. For Cluster #3, only few treatment vulnerabilities, like EGFR/ERBB inhibitors or selected epigenetic drugs (e.g., HDAC inhibitor vorinostat, IDH2 blocker AGI‐6780) were identified. In turn, for Cluster #3 tumours, general resistance to cytotoxic drugs, PARP inhibitors and multiple RAS/RAF/MAPK inhibitors was predicted.

Based on the differential gene and protein expression analyses, we could not propose any of the clusters as particularly susceptible to ADC therapy in general. Yet, given the highest expression of EGFR in Cluster #2, it may be put forward for therapy with anti‐EGFR ADCs, like depatuxizumab–mafodotin. In turn, the high expression of TROP2/TACSD2 and NECTIN4 proteins in Cluster #3 may render these cancers sensitive to Sacituzumab govitecan or EV (Figure S32).

For a subset of patients in the TCGA BLCA dataset, detailed information on pharmacological treatment was available,^91^ which enabled a comparison of OS of patients with and without at least one round of systemic cytotoxic chemotherapy in UC clusters. The systemic cytotoxic chemotherapy effectively prolonged survival in Cluster #1 (median OS, with cytotoxic therapy: 860 days, without cytotoxic therapy: 590 days) and in Cluster #2 (median OS, with cytotoxic therapy: not reached, without cytotoxic therapy: 620 days). In turn, cytotoxic chemotherapy was ineffective in Cluster #3 patients (median OS, with cytotoxic therapy: 2000 days, without cytotoxic therapy: 2600 days; Figures 5C and S5 and Table S50).

### 3.7 UC cell lines recapitulate key biological features of UC clusters and distinct treatment approaches were predicted in silico

**FIGURE 6:**
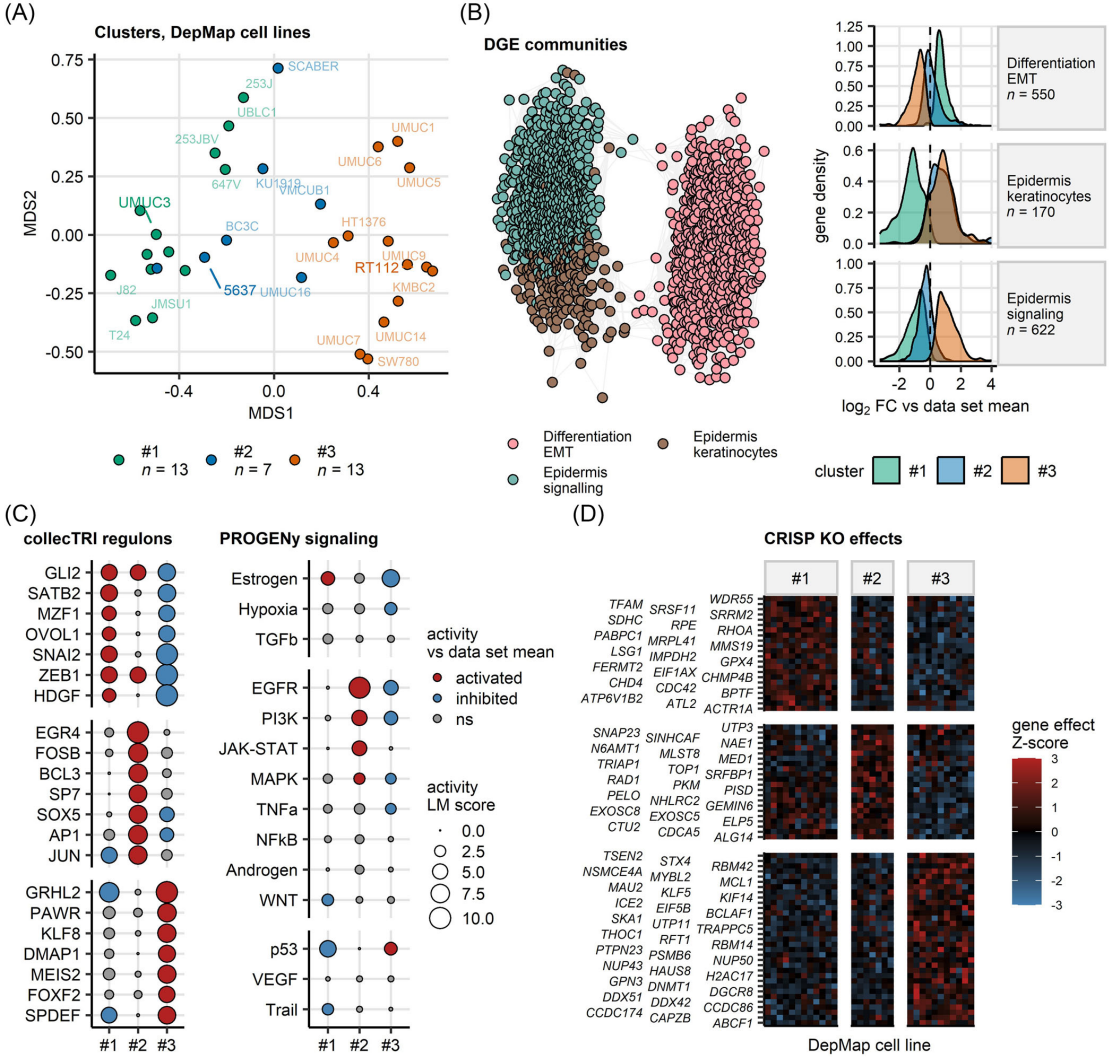
Phenotype of bladder cancer clusters of urothelial cancer cell lines. DepMap urothelial cell lines (*n* = 33) were assigned to bladder cancer clusters by an Elastic Net classifier trained in the TCGA BLCA bulk cancer cohort. (A) Visualisation of bladder cancer clusters of DepMap cell lines via multi‐dimensional scaling (MDS). The MDS embeddings of transcript levels used by the Elastic Net classifier are visualised as a scatter plot. Each point represents a single cell line. Point colour codes for cluster assignment; points are labelled with cell line names. Numbers of cell lines in bladder cancer clusters are indicated in the plot legend. (B) Co‐regulation network of genes differentially expressed between bladder cancer clusters of DepMap cell lines. log_2‐transformed gene expression levels were compared between bladder cancer clusters by one‐way ANOVA with *η* ^2^ effect size statistic. Differences between mean log_2 expression levels in the clusters and the respective DepMap collection means were evaluated by one‐sample *T*‐test. *p* values were corrected for multiple comparisons with the false discovery rate (FDR) method. Significantly differentially regulated genes and proteins were defined by ANOVA pFDR <.05, *η* ^2^ ≥.06 and *T*‐test pFDR <.05. Co‐expression patterns of the differentially regulated genes were investigated by a network analysis. The networks edges were defined by pairwise correlations of gene or protein levels with Spearman's *ρ* ≥.5 and weighted by the corresponding *ρ* values. Isolated vertices, that is, genes without correlation partners were removed. Communities of co‐regulated genes were identified by the Leiden algorithm and named after their key biological processes revealed by biological process gene ontology enrichment. The co‐expression network was visualised as a force‐directed graph with the Fruchterman–Reingold algorithm with single genes represented by points, and correlations between genes with *ρ* ≥.5 depicted as edges. Point colour codes for the community assignment. Density of estimates of log_2 fold changes of expression in the clusters as compared with the DepMap collection mean for members of gene and protein communities are presented in density plots; numbers of genes in the communities are indicated in the plot facets. (C) Activity of transcriptional regulons and signalling in bladder cancer clusters of DepMap cell lines. Activity of collecTRI transcriptional regulons and PROGENy signalling pathways in bladder cancer clusters as compared with the DepMap collection means was modelled by, respectively, univariable and multivariable linear decoupleR modelling algorithms. The algorithms were fed with *p* value‐weighted log_2 fold‐change estimates of differential expression for all available genes. *p* values were corrected for multiple testing with the FDR method. Linear modelling (LM) score served as a metric of activity in a cluster as compared with the DepMap collection mean. The LM scores for the top seven strongest cluster‐specific regulons and all investigated signalling pathways are presented in bubble plots. Point sizes code for absolute values of LM scores. Point colours code for the activity status and significance. (D) Identification of essential genes specific for bladder cancer clusters by analysis of the DepMap CRISPR‐Cas9 knockout screen. Distribution of Chronos scores (positive values: growth inhibition) of CRISPR‐Cas9 knockouts in bladder cancer clusters of DepMap urothelial cancer cell lines (#1: *n* = 12, #2: *n* = 7, #3: *n* = 11) was modelled by bootstrapping. For *n* = 77 genes, biologically significant knockout effects with Chronos score >.5 in at least one cluster, and significant differences in Chronos scores between the clusters were discerned. Their Chronos scores are presented in a heat map.

Cancer cell lines belong to the indispensable toolbox of tumour biology and pharmacology. We investigated if the phenotypes of UC clusters of bulk UC can be emulated by UC cell lines, which may hence serve to study biology and treatment response of UC clusters in an in vitro setting. We reliably assigned 33 UC cell lines to UC clusters, with complete clustering described in the Methods and Supporting Information. Cluster #1 cell lines were 253J, TCCSUP, UBLC1, 253JBV, T24, UMUC3, JMSU1, 647V, 639V, J82, SW1710, SLR20 and UMUC11. Cell lines VMCUB1, SCABER, 5637, BC3C, KU1919, UMUC16 and UMUC13 were assigned to Cluster #2. Cluster #3 cell lines were SW780, HT1376, KMBC2, UMUC1, RT4, RT112, UMUC6, UMUC14, UMUC7, RT11284, UMUC5, UMUC9 and UMUC4 (Figure 6A and Table S51). These UC clusters displayed similar modulation of transcriptome and Reactome Pathway gene signatures and also showed comparable activity patterns of transcription factors and signalling as UC cluster of bulk UC. Figures 6B,C and S6 and S29–S31, and Tables S52–S59 provide a comprehensive overview of hallmark genes in representative UC cluster cell line models.

To identify essential genes of UC clusters as potential oncogenic drivers,^81^ we compared the effects of CRISPR‐Cas9 KOs between the clusters of UC cell lines. This comparison, done with bootstrap modelling, identified, among others, mevalonate kinase *MVK*, small GTPase *RHOA*, and the detoxification enzyme and ferroptosis inhibitor glutathione peroxidase (*GPX)* as essential genes for Cluster #1. DNA methylase *N6AMT1* and glycolysis enzyme *PKM* were identified as Cluster #2 essential genes. Apoptosis‐related *MCL1* and transcription factor *KLF5* were identified as essential genes for Cluster #3 (Figure 6D and Table S60).

**FIGURE 7:**
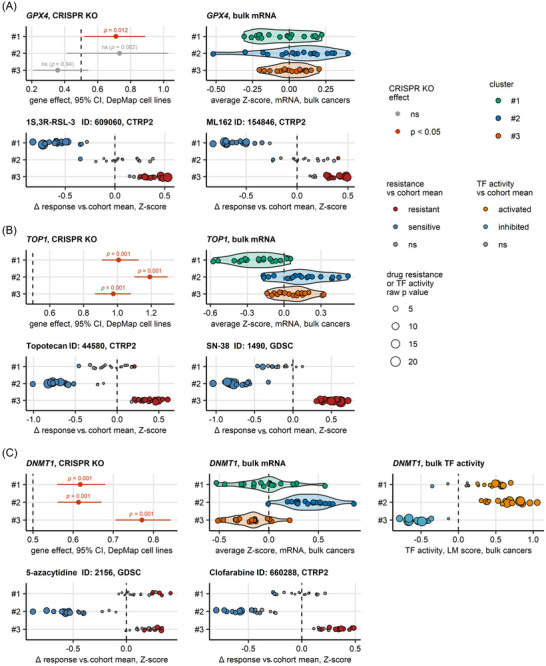
Identification of druggable essential genes for bladder cancer data by integration of cell line and bulk cancer information. Candidate essential genes of bladder cancer clusters with existing pharmacological inhibitors or modulators were identified by inspection of the CRISPR‐Cas9 knockout screen results of DepMap urothelial cell lines (D, #1: *n* = 12, #2: *n* = 7, #3: *n* = 11 cell lines), differential gene expression in bulk cancer transcriptomes (Figure 4B, *n* = 19 cohorts), activity of transcriptional regulons predicted for bulk cancer transcriptomes by collecTRI/decoupleR (Figure 5C, *n* = 18 cohorts) and drug response predictions made for bulk cancer transcriptomes by RIDGE machine learning (Figure 6, *n* = 18 cohorts). Estimates of distribution of Chronos score of effects of CRISPR‐Cas9 knockouts in bladder cancer cluster of the cell lines were obtained by bootstrapping. The estimated means Chronos scores with 95% confidence intervals (95% CI) in the clusters of DepMap cell lines are shown in Forest plots (points: means, whiskers: 95% CI; significant biological effects with Chronos scores >.5 labelled in red). Distributions of average Z‐scores of log_2‐transformed mRNA levels in bulk cancers are depicted in violin plots, with averages of single cohorts and clusters represented by points. Linear modelling (LM) scores of activity of collecTRI regulons (TF: transcription factors) are presented in dot plots. Point sizes in these dot plots correspond to −log10‐transformed *p* values; point colours code for activity status and significance. Estimates of differences in drug resistance Z‐scores between bladder cancer clusters and the cohort means are presented in dot plots. Point sizes in these dot plots correspond to −log10‐transformed *p* values; point colours code for resistance status and significance. Compound name and identifier, and name of the dataset used for prediction of resistance are indicated in the plot titles. (A) GPX4 as a candidate essential gene specific for Cluster #1, and GPX4 inhibitors RSL‐3 and ML162 and ferroptosis inducers as candidate therapy of Cluster #1 cancers. (B) Topoisomerase‐coding TOP1 as a candidate essential gene for Cluster #2, and topoisomerase I inhibitors Topotecan and SN‐38 as candidate therapy for Cluster #2 cancers. (C) DNA methylase‐coding DNMT1 as a candidate essential gene for Cluster #2. Note the substantial biological effects of DNMT1 knockout in all clusters, and upregulation of DNMT1 expression and activity in Cluster #2. DNMT1 inhibitors azacytidine and clofarabine are proposed as candidate therapeutic options for Cluster #2 cancers.

Combining the CRISPR‐Cas9 KO data of UC cell lines with gene expression, transcription factor activity and pharmaco‐informatics of bulk cancers, we sought to dissect molecular targets for future therapy of UC tailored for particular UC clusters (Figure 7). The largest effects of *GPX4* KO were observed in Cluster #1 UC cell lines, and Cluster #1 bulk UC were predicted to be sensitive to GPX4 inhibitors and ferroptosis inducers (e.g., 1S,3R‐RSL‐3, ML162). This suggests GPX4 blockade as a candidate treatment approach for Cluster #1 UC (Figure 7A). Furthermore, the topoisomerase *TOP1* and DNA methylase gene *DNMT1* were identified by the CRISPR‐Cas9 screening as essential for Cluster #2 cell lines, found over‐expressed by Cluster #2 UC, and Cluster #2 cancers were predicted to respond to TOP1 and DNMT1 inhibitors (TOP1: topotecan, SN‐38; DNMT1: 5‐azacytidine, clofarabine). Hence, topoisomerase and DNA methylase inhibition may prove attractive therapy options for Cluster #2 cancers (Figure 7B,C).

### 3.8 In vitro drug screening identified several promising compounds to target the different clusters

**FIGURE 8:**
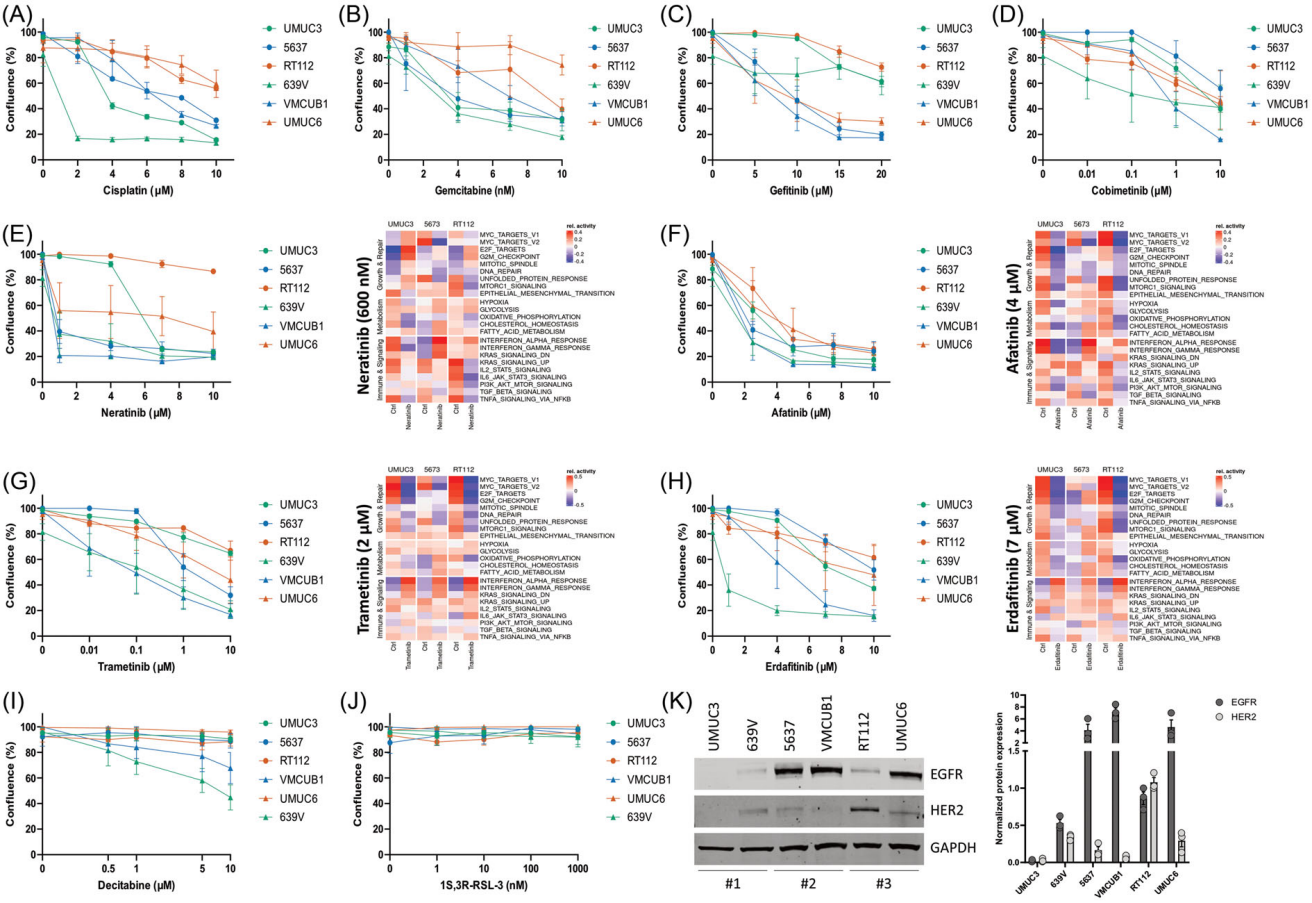
In vitro response to anti‐cancer drugs and RNA‐seq in urothelial cancer cell lines representative for the three bladder cancer clusters. Response to chemotherapy drugs (A) cisplatin and (B) gemcitabine, (C) EGFR/ERBB inhibitor gefitinib and (D) MAPK inhibitor cobimetinib. Drug response graphs are shown with the corresponding RNA‐seq data for EGFR/ERBB inhibitors (E) neratinib and (F) afatinib, (G) MAPK inhibitor trametinib and (H) FGFR inhibitor erdafitinib. Drug response graphs are furthermore given for the DNA methyltransferase inhibitor (I) decitabine and the ferroptosis activator (J) 1S,3R‐RSL‐3. Confluence was measured after 4 days using the IncuCyte for Cluster #1 cell lines UMUC3 and 639V, Cluster #2 cell lines 5637 and VMCUB1, and Cluster #3 cell lines RT112 and UMUC6. Points represent mean measurements for three independent biological replicates with error bars representing the SEM, with colour‐coding for the cell line/cluster assignment. Heatmaps show regulation of growth and repair, metabolism and immune and signalling pathways in the control versus treated cell line models. Tile colour corresponds to the relative activity of the gene signatures. (K) Pathway analysis showing western blot bands, analysing protein expression levels of EGFR and HER2 in the different cluster cell lines, and the protein expression normalised by the housekeeper GAPDH. Points represent mean measurements for three independent biological replicates with error bars representing the SEM.

In addition to the in silico drug prediction, we tested several promising compounds in vitro using the representative cell line models. We selected UMUC3 and 639 V, 5637 and VMCUB1, and RT112 and UMUC6, respectively for Clusters #1, #2 and #3, as a promising models representing their cluster, based on transcriptome and Reactome Pathway gene signatures and comparable activity patterns of transcription factors and signalling. For the in vitro drug screen, cells were treated with a range of different inhibitors (Figure 8). No differences between the clusters were observed in response to the tested MAPK inhibitor cobimetinib (Figure 8D), EGFR inhibitor afatinib (Figure 8F), FGFR inhibitor erdafitinib (Figure 8H), DNA methyltransferase inhibitor decitabine (Figure 8I) and ferroptosis activator 1S,3R‐RSL‐3 (Figure 8J). Chemotherapy agents’ cisplatin and gemcitabine were most effective in the Cluster #1 cell lines compared with the Clusters #2 and #3 cell lines (Figure 8A,B).

Our pharmaco‐informatics data furthermore pinpointed EGFR/ERBB and MEK/ERK inhibition as novel potential approaches to treatment of Cluster #2 UC. Sensitivity of the Cluster #2 cell lines to most of the tested EGFR/ERBB inhibitors was substantially higher than that of Cluster #1 and Cluster #3 cell lines (Figure 8). Gefitinib and neratinib were particularly effective in 5637 and VMCUB1 cells, with an intermediate response for the Cluster #1 cell lines (Figure 8C,E). In addition, the Cluster #3 cell line UMUC6 responded well to gefitinib and neratinib treatment, most likely due to the high protein expression levels of the EGFR (Figure 8K). In general, the EGFR protein expression was highest in the Cluster #2 cell lines, and lowest in the Cluster #1 cell lines. Furthermore, the protein expression levels of HER2 were highest in Cluster #3 cell lines, and lowest in the Cluster #1 cell lines (Figure 8K). The in vitro investigated MEK/ERK inhibitor trametinib was also more effective at inhibition of the Cluster #2 cell lines as compared with the Cluster #1 cell line UMUC3 and Cluster #3 cell lines (Figure 8G). These findings are according to our in silico prediction, and EGFR/ERBB and MEK/ERK inhibitors might be a promising treatment approach for Cluster #2 tumours.

### 3.9 RNA‐seq data revealed the effect of treatment strategies on gene expression in the different clusters

To investigate the underlying mechanisms and molecular drivers of the differential responses to afatinib, neratinib, trametinib and erdafitinib, we performed RNA‐seq on the representative cell lines from the three clusters (Figure 8 and Table S68). The concentration for each drug was selected based on our proliferation assays, ensuring the analysis captured a potent transcriptional response. Treatment with the EGFR inhibitor neratinib (600 nM, Figure 8E) significantly downregulated activity signatures for the key EGFR effector KRAS and the oncogene MYC in the sensitive 5637 cell line. In contrast, inhibition of KRAS signalling was absent in UMUC3 cells and inconsistent in RT112 cells. Of note, neratinib also specifically induced the interferon‐alpha/gamma response gene signatures in 5637 cells. For the EGFRi afatinib (4 µM, Figure 8F), gene expression confirmed the potent anti‐proliferative effect across all three cell line clusters as proliferation‐related signatures were consistently downregulated in all tested cell lines. Furthermore, key downstream EGFR pathways, including PI3K/AKT signalling, were inhibited in all three cell lines. Similar to what was seen with neratinib, the KRAS pathway was significantly downregulated in 5637 and RT112. While the anti‐proliferative effect was universal, afatinib's impact on other biological processes was highly divergent across the cell lines. Induction of the interferon‐alpha/gamma response gene signatures was again restricted to 5637 cells, whereas UMUC3 and RT112 cells showed a reduction in IL6/JAK/STAT3 signalling activity. Energy and metabolism‐related pathways were significantly downregulated in UMUC3 and RT112 cells, but not in 5637 cells. In addition, RT112 exhibited a broad downregulation across various other signalling pathways, which was also seen upon treatment with neratinib.

Gene expression analysis for the MAPK inhibitor trametinib (2 µM, Figure 8G) indicated a significant downregulation of proliferation‐related pathways in all cell lines, with the most profound effects observed in UMUC3 and 5637 cells, which is in‐line with the observed anti‐proliferative effects. These changes align with on‐target MAPK pathway inhibition and mirror the effects of upstream EGFR blockade.^92^, ^93^ We observed a uniform induction of the interferon‐alpha/gamma signatures in all cell lines, along with a less pronounced upregulation of the IL‐6/JAK/STAT3 response pathway. Regarding the metabolism, a decrease in oxidative phosphorylation and cholesterol homeostasis was observed in UMUC3 and RT112, while these were upregulated in 5637. RNA‐seq of the cells treated with the FGFRi erdafitinib (7 µM, Figure 8H) revealed clear downregulation of proliferation‐related signatures in UMUC3 and RT112 cells, but not for 5637 cells. This aligns with the known dependency of these cell lines on FGFR activity, which is driven by FGFR1 overexpression in UMUC3^94^ and FGFR3‐TACC3 fusion in RT112.^95^ Metabolism‐related signatures were frequently downregulated in the sensitive cell lines, and the interferon‐alpha/gamma response gene signatures were significantly induced. In contrast, metabolism‐related signatures were conversely upregulated in the 5637 cell line. Overall, the RNA‐seq data support the in vitro *drug* screen, showing decreased proliferation and inhibition of related pathways, providing insight into the mechanisms of action of the selected compounds.

## 4 DISCUSSION

Previous consensus molecular classifications based on transcriptomic data have advanced our understanding of biological heterogeneity in UC.^9^, ^22^ Yet, their exclusivity for either NMIBC or MIBC and unclear validity in bulk UC proteome and UC cell line data have hindered integration into clinical guidelines, routine practice and in vitro applicability. To address these challenges in UC research, we developed and validated a molecular classification scheme universally applicable to NMIBC and MIBC, bulk UC transcriptome, bulk UC proteome and UC cell lines, in a large semi‐supervised analysis of over 4000 UC samples and 33 UC cell lines. The highly consistent sets of UC cluster marker genes, and the reproducible clinical and biological features of the novel clusters across the transcriptome and proteome data confirm the robustness of our novel molecular classification system.

Several molecular alterations reported in our clusters could explain the clinical outcomes. Cluster #1 is associated with poor survival and is characterised by strong stromal signatures. A role of stromal growth factors has been found in acquisition of resistance to anti‐PD‐L1 therapies in bladder cancer, and epithelial to mesenchymal transition is frequently induced by TGF‐ß.^96^ Immune deconvolution revealed high macrophage presence and elevated T and B cell infiltration, consistent with the literature reporting lymphocyte enrichment in more invasive disease stages.^22^, ^97^ Further classification of T cell subtypes in stroma and tumour could therefore improve tumour stratification. In addition, our immune deconvolution can be an addition, specifying on UC, to the study of Thorsson et al., who have identified six immune subtypes general across different cancer types.^98^ Cluster #1 also showed high expression levels of the ECM. Because of Cluster #1's high ECM expression, targeting ECM components, alone or in combination with standard therapy or DNA‐targeting agents, may be investigated in the future.^99^ A high ECM score in UC has furthermore been positively associated with immune cells and fibroblasts, and they suggested that tumours with abundant ECM respond better to immune checkpoint inhibition.^100^ Given the anatomic location of UC and the ECM‐specific gene expression patterns, a liquid biopsy‐based stratification using ECM‐derived urinary peptides may be a valuable approach,^101^, ^102^, ^103^ especially for NMIBC where extended RNA sequencing or molecular profiling are rarely reported. Different urine‐based biomarker panels have been studied in the past to detect primary and recurrent UC and to support patient screening in a non‐invasive setting.^104^, ^105^ Developing a panel that could separate between the three clusters could be a promising next step for clinical implication, however, urinomics for Cluster #3 might be more challenging because of the lack of ECM.

Cluster #2 represents an aggressive phenotype, with relatively low OS and enrichment for cancers assigned to the basal/squamous molecular class of MIBC.^14^, ^22^ This cluster showed pronounced immune infiltration and active JAK/STAT signalling, which is in line with several previous studies.^22^, ^106^, ^107^ STAT3 is phosphorylated in response to interleukin (IL)‐6 or EGF in various human tumours. The Hippo signalling pathway is highly conserved and is known as a major regulator of proliferation and apoptosis. Its effector YAP1 was recently implicated in regulating IL6/STAT3 signalling and cellular stemness in UC^108^ making it a promising target. These results have clinical relevance as STAT3 activation was reported as an indicator of poor prognosis in advanced UC.^109^ JAK/STAT components and S100 family proteins may serve as valuable markers for identifying this subtype.^41^ Cluster #2 tumours also exhibit a hypoxic microenvironment, which contributes to enhanced proliferation, angiogenesis, therapy resistance and poor prognosis.^22^, ^110^, ^111^, ^112^, ^113^

Cluster #3 includes primarily NMIBC or luminal–papillary MIBC with low progression risks.^12^ These tumours often harbour *FGFR3* mutations and lack significant immune infiltration (‘immune desert’).^12^, ^22^, ^114^, ^115^ The minimal ECM presence further aligns with a more favourable prognosis.^100^ Interestingly, we observed that Cluster #3 tumours could evolve into the more aggressive clusters #1 and #2 after the first recurrence or when the tumour progressed. The progression of UC into clinically more aggressive subtypes has been observed in many patients, and while several studies have tried to analyse this progression and its molecular profile, much information is still unknown.^116^, ^117^ We believe that, given the low sample numbers in our longitudinal analyses, the questions if and how bladder cancer clusters evolve during recurrences and progression calls for further prospective studies. Prediction model implementation, such as the use of biomarkers, might also be a promising next step to make clinical predictions and intervene at an earlier stage to prevent progression or recurrence.^118^, ^119^ Furthermore, a previous discrepancy was observed comparing primary tumours with synchronous or metachronous distant metastases. Here, protein‐based IHC showed no differences between primary and metastatic tumours, while transcriptome‐based molecular subtypes showed more heterogeneous results, with a switch of luminal towards stroma‐rich tumours.^120^ Our research can be an addition to previous publications looking into molecular changes during progression, and changes in gene expression from primary tumours to progression and recurrence sites might be analysed using IHC in future studies.

Current treatment options for UC with an aberrant histology (UCAH) subtype are limited in efficacy, partly due to tumour heterogeneity.^8^ UCAH has worse survival and is underrepresented in clinical trials.^121^, ^122^ which mostly focus on pure UC.^5^, ^6^ To address this, we propose cluster‐specific treatment strategies applicable to both NMIBC and MIBC, supported by drug prediction using machine learning and cell line validation.

Combining the in silico prediction and in vitro drug screen, ferroptosis inducers, several cytotoxic drugs, growth factor inhibitors and DNA‐targeting compounds like PARP inhibitors showed potential in the treatment of Cluster #1 tumours. These findings are supported by several other papers that show that ferroptosis induction is an emerging new approach for UC^123^, ^124^, ^125^ and might be a valid strategy with chemotherapy or DNA‐targeting agents like PARPi, which have shown synergistic effects in other tumour types.^125^, ^126^, ^127^, ^128^, ^129^ 1S,3R‐RSL‐3 can bind to GPX4, resulting in increased levels of lipid peroxides and membrane damage. Zhao et al., previously showed that knockdown of staphylococcal nuclease and tumour domain containing 1 (SND1), or GPX4 itself, could overcome chemotherapy resistance in bladder cancer.^130^ This synergistic effect might explain why treatment with 1S,3R‐RSL‐3 alone did not show any effect in vitro.

A similar explanation could be given for the use of DNA methyltransferase inhibitors in Cluster #2. Where the in silico prediction showed DNMT1 to be essential for Cluster #2, decitabine mono‐treatment did not show any effect in vitro. Previously, Wu et al., showed that treatment with low doses of decitabine can increase the sensitivity to chemotherapy agents in vitro and in vivo, mainly because of inhibited STAT3 signalling.^131^ In future studies, the synergy between DNA methyltransferase inhibitors and chemotherapy agents should be further investigated.

In Cluster #2, cytotoxic agents, EGFR/ERBB inhibitors (e.g., neratinib, afatinib or gefitinib) and MEK/ERK inhibitors (e.g., trametinib) showed strong efficacy, aligning with the known basal‐like UC responsiveness to EGFR‐targeted agents.^14^, ^132^ Enhanced MAPK activity in this cluster supports MEK inhibition as a possible therapeutic approach.^133^, ^134^, ^135^ Furthermore, given the immune‐active profile, anti‐PD‐1/L1 should be further explored in Cluster #2 tumours.^136^, ^137^ A previous study showed that the presence of *RB1* and *TP53* mutations, which are common in Cluster #2 patients, correlates with the response to ICIs^138^ and thus highlighting the benefit of ICI in this cluster.

Since most therapeutic approaches were proposed for Cluster #2 tumours, we were interested in the mechanisms underlying treatment strategies for this highly aggressive basal/squamous‐like cluster. It is well‐known that EGFR is frequently expressed in basal‐type bladder cancers, being associated with adverse prognostic indicators, lamina propria invasion and high‐grade cytology.^139^ In line with this fact, patient‐derived xenografts (PDXs) confirmed the effectiveness of EGFR inhibitors in basal/squamous UC models.^140^ Furthermore, previous in vitro data already provided additional support for the potential benefit of treatment with anti‐EGFR tyrosine kinase inhibitors (TKIs) and chemotherapeutic agents in patients with UC harbouring squamous features, particularly through dual targeting of the EGFR signalling pathway at multiple levels.^132^, ^141^ This concept was evaluated in a clinical trial using cetuximab and erlotinib in chemotherapy‐refractory, advanced colorectal cancer, which showed synergistic effects on growth inhibition of colon cancer cell lines.^142^ We performed RNA‐seq on treated cell lines to assess mechanistic insights from drug response assays. Neratinib reduced proliferation, especially in Cluster #2, with EGFR inhibition affecting MAPK signalling and MYC activity.^143^ Its effectiveness may be related to high EGFR expression in this cluster. Afatinib broadly inhibited cell growth across clusters, although its lower HER2 potency^144^, ^145^, ^146^ may explain variable effects compared to neratinib. Differences in HER2 levels between 5637 and UMUC3 cells may support this hypothesis.^147^

Cluster #3 tumours may be inhibited by FGFR inhibitors, consistent with the high frequency of *FGFR3* mutations or fusions in this group.^12^, ^148^, ^149^ Our in silico drug analysis did not account for mutational status, however, erdafitinib, an approved FGFR inhibitor^7^, ^150^ showed anti‐proliferative effects across all clusters. These results are in line with several previous publications investigating FGFR expression levels rather than mutational status, and they suggest that FGFR inhibition may be effective regardless of the mutational status, which could be explained by additional mechanisms of action.^73^, ^151^ Luminal tumours also express high levels of ERBB2 and micropapillary tumours often harbour *ERBB2* activating mutations,^152^, ^153^, ^154^ which is in line with our findings that EGFR/ERBB inhibition was predicted in silico as novel strategy in Cluster #3. However, this effect could not be recapitulated in vitro. The use of epigenetics has also been suggested in this study for Cluster #3 tumours, and in other papers as a novel therapy approach for UC.^155^, ^156^, ^157^

Limitations of our study include variability in the cohort sizes and cell line heterogeneity. Besides this, we developed our UC clusters in a cohort consisting predominantly of MIBC. Hence, the clustering scheme is likely less effective in NMIBC than in MIBC. Furthermore, the drug sensitivity analysis is trained with cell lines spanning over different tumour entities, containing only 33 UC cell lines with an epithelial origin. The absence of the TME in cell line models can limit immune‐related interpretations.^158^, ^159^, ^160^, ^161^ In particular, immune deconvolution as compared with direct quantification of immune infiltration by flow cytometry, single cell RNA sequencing or immunohistochemistry, may deliver false positives or poorly calibrated levels of immune cells in the tumour samples. Analogically, biological processes identified by gene set variation analyses with gene signatures need careful validation with cell lines, patient‐derived material and animal models. Therefore, spatial transcriptomics or single‐cell RNA sequencing could be used in future studies to enhance the tumour cluster validation, including more information on the TME. In addition, follow‐up research should leverage advanced models, such as patient‐derived organoids and animal models, which better recapitulate UC biology. In recent years, several studies analysed these models having different molecular backgrounds, representing the molecular heterogeneity in UC.^140^, ^162^, ^163^ Applying our clustering scheme to these models may allow deeper investigation into tumour mechanisms and therapy response. Since an IHC‐based subtyping algorithm may offer a faster, less expensive and clinically applicable alternative to transcriptomic profiling.^164^ IHC‐based profiling of our proposed markers, such as MYH11 and COL14A1 for Cluster #1, STAT1 and S100A8 for Cluster #2, and SSH3 and S100P for Cluster #3 should be validated in a follow‐up study. This IHC‐based stratification can then be incorporated into clinical trials investigating cluster‐specific treatment response and might be a main cornerstone in routine clinical practice.

## 5 CONCLUSION

In this study, we introduce a novel molecular classification scheme for UC, comprising three distinct clusters that were consistently derived from whole‐transcriptome analyses and validated across independent proteomic and transcriptomic datasets. This framework is applicable to both NMIBC and MIBC and stratifies patients into biologically and clinically distinct entities with different prognostic profiles. To bridge discovery and translation, we assigned publicly available UC cell lines to each cluster, enabling functional validation and drug testing in representative in vitro models. Moreover, we predicted and prioritised promising therapeutic compounds and proposed a tailored treatment strategy: DNA‐targeting agents for Cluster #1; cytotoxic agents, EGFR/ERBB and MEK/ERK inhibition for Cluster #2; and selected epigenetic drugs, as well as EGFR/ERBB and FGFR inhibition, for Cluster #3. This unique taxonomy may serve as a practical basis for diagnostic stratification and personalised treatment guidance that can be tested in clinical trials. It would be very important to achieve a consensus based on results obtained with highly complex ‘multi‐omics’ technologies. Future efforts should focus on validating our results and testing hypotheses related to therapeutic opportunities in patient‐derived models and prospective clinical cohorts to accelerate potential clinical implementation.

## AUTHOR CONTRIBUTIONS

Renate Pichler, Nils C. H. van Creij, Piotr Tymoszuk, Andreas Seeber and Zoran Culig made the study concept and design. Renate Pichler, Nils C. H. van Creij and Piotr Tymoszuk performed the bioinformatic analysis, cluster development and cluster validation. Renate Pichler, Nils C. H. van Creij, Teresa Sellemond, Hamed Wafa, Walther Parson and Zoran Culig performed and analysed the in vitro work. Renate Pichler, Nils C. H. van Creij and Florian Handle performed and analysed the RNA sequencing. Renate Pichler, Nils C. H. van Creij, Piotr Tymoszuk and Zoran Culig wrote the manuscript. Agnieszka Martowicz, Eva Comperat, Steffen Ormanns, Michael Günther, Maxim Noeparast, Frédéric R. Santer, José Daniel Subiela, Petros Grivas and Roger Li provided their expertise on the topic. Renate Pichler supervised the study. All the authors read and approved the manuscript.

## CONFLICT OF INTEREST STATEMENT

Florian Handle is the owner and CEO of XPseq Analytics GmbH. Petros Grivas reports in the last 2 years consulting with MSD, Bristol Myers Squibb, AstraZeneca, EMD Serono, Pfizer, Janssen, Roche, Astellas Pharma, Gilead Sciences, Strata Oncology AbbVie, Bicycle Therapeutics, Replimune, Daiichi Sankyo, Foundation Medicine, Eli Lilly, Urogen, Tyra Biosciences, Natera, and research funding from MSD, EMD Serono, Gilead Sciences, Acrivon Therapeutics, ALX Oncology and Genentech (paid to institution). Renate Pichler reports consulting with MSD, AstraZeneca, Janssen, Astellas Pharma, Eisai, Ipsen, Merck, and research funding from Ipsen, Astellas Pharma and AstraZeneca. The other authors declare no conflicts of interest.

## FUNDING INFORMATION

The authors received no specific funding for this work.

## ETHICS STATEMENT

The authors have nothing to report.

## CONSENT FOR PUBLICATION

Not applicable.

## Supporting information



Supporting Information

Supporting Information

## Data Availability

The analysed cohorts are freely available. The entire pipeline is available as a GitHub repository (https://github.com/PiotrTymoszuk/BLCA‐cluster‐paper). The RNA‐seq data from this study have been deposited in the Gene Expression Omnibus (GEO) repository under accession number GSE308936.
