# Supplementary material for: Multi‐omic profiling defines three distinct molecular subtypes of urothelial carcinoma with implications for precision therapy
Source: Clin Transl Med. 2026 Mar 10;16(3):e70638. doi: 10.1002/ctm2.70638 (PMC12973134; doi:10.1002/ctm2.70638)
Supplement: Supplementary file 1 — Supporting Information [file CTM2-16-e70638-s001.docx]

# Supplementary Methods

## Software

Data handling and analyses were done with R version $\geq$ 4.2.3. Tabular, list, and vector data were managed with *tidyverse* package bundle (1), *rlang* (2) and [*trafo*](https://github.com/PiotrTymoszuk/trafo) packages. Text data were handled with *stringi* package (3).

Tabular data were presented with *flextable* (4). Most analysis results were visualized with *ggplot2* (5) (e.g. box, bar, stack, dot, bubble, Forest, violin, and density plots), [*ExDA*](https://github.com/PiotrTymoszuk/ExDA) (e.g. box and dot plots), [*microViz*](https://github.com/PiotrTymoszuk/microViz) (Volcano and Forest plots, heat maps), and [_clustTools](https://github.com/PiotrTymoszuk/clustTools) (e.g. uniform manifold approximation and projection [UMAP] embeddings plots), as well as *igraph* (6), [*graphExtra*](https://github.com/PiotrTymoszuk/graphExtra) and *ggnetwork* (7) (force-directed graphs of networks) packages. Overlapping text labels were handled by *ggrepel* (8). HTML elements in plots were managed by *ggtext* (9). Extended color palettes were obtained with *ggthemes* package (10). Figures were built with *cowplot* (11).

Parts of the manuscript and **Supplementary Material** were written in markdown with *rmarkdown* (12) and *bookdown* (13) packages. Figures, tables, and URL links were managed in markdown documents with [*figur*](https://github.com/PiotrTymoszuk/figur) package tools.

Other R packages specific for particular analysis tasks are referenced in the corresponding paragraphs of **Supplementary Methods**. R packages used in the analyses are listed with short descriptions of their usage, sources, links to sources, and publications in **Supplementary Table S1**.

## Data sources

Data resources (transcriptomic and proteomic bulk cancer cohorts, gene signature data bases and source publications, cell line collections, and drug screening data) are listed in **Supplementary Table S2**.

### Bulk cancer transcriptome

The [cBioportal](https://www.cbioportal.org/), [Gene Expression Omnibus (GEO)](https://www.ncbi.nlm.nih.gov/geo/) and [Array Express](https://www.ebi.ac.uk/biostudies/arrayexpress) repositories, as well as the [PubMed literature portal](https://pubmed.ncbi.nlm.nih.gov/) were searched for eligible whole-transcriptome data sets of urothelial cancers. The selection criteria were $\geq$ 100 unique urothelial cancer samples (re-analysed samples were excluded), at least 18000 genes detected, transcriptome measurements with a commercial microarray or RNAseq platform, and availability of $log_{2}$-normalized expression or gene/transcript count data or expression Z-scores. One study, GSE57933, was excluded from potential data sources, since its expression information could not be programmatically accessed with tools of the *GEOquery* package (14) or ad-hoc developed R tools. The following 19 whole-genome expression data sets were re-analyzed in the current report:

- **TCGA BLCA** (15,16). RNA sequencing, somatic mutation, copy number alteration, and protein expression data (reverse phase protein array [RPPA]) data sets for 407 muscle-invasive bladder cancer (MIBC) specimens were fetched from the cBioportal and [GDC](https://portal.gdc.cancer.gov/analysis_page?app=Projects) repositories with in-house developed scripts. The accompanying clinical information consisted of demographic variables (e.g. age, sex, BMI, smoking history), pathological features (e.g. pathological staging, histology, grade, extra-capsular extension), and survival information (overall, tumor-specific and relapse-free survival). The relapse-free survival was defined as time after initial treatment without disease or pathological progression. Manually curated data on chemotherapy treatment status, drug names, and clinical response for the TCGA BLCA cohort were obtained from Supplementary Table S2 of the publication by Moiso (17). Assignment of cancer samples to PAM50 molecular classes was derived from Supplementary Table S2 of the publication by Zhao et al. (18).
- **IMvigor** (19,20). The IMvigor data set consisted of 221 bladder and ureter MIBC specimens of the IMvigor210 trial subjected to RNA and DNA sequencing. The study participants were treated with an anti-PD-L1 drug (atezolizumab) following a platinum chemotherapy or in patients no illegible for a platinum chemotherapy. The cohort’s data included gene expression, somatic mutation and clinical information (e.g. sex, race, smoking, best overall response and overall survival). The data sets were obtained with the R package [*IMvigor210CoreBiologies*](https://github.com/SiYangming/IMvigor210CoreBiologies).
- **GSE13507** GEO collective (21) comprised 188 bladder cancer samples (primary cancers: n = 165, recurrent cancers: n = 23; non-muscle invasive bladder cancers [NMIBC]: n = 103, MIBC: n = 62), including n = 15 donor-matched longitudinal transcriptome samples of primary and recurrent non-muscle invasive cancers. Transcriptome was measured with the Illumina human-6 v2.0 expression beadchip microarray. The clinical information included age and sex variables, pathological staging, muscle-invasiveness, and overall survival.
- **GSE32548** GEO cohort (22) consisted of 131 bladder cancer samples (NMIBC: n = 92, MIBC: n = 38). Gene expression was determined with the Illumina HumanHT-12 V3.0 expression beadchip microarray. Pathological tumor stage and tumor grade were provided as accompanying clinical data.
- **GSE48075** GEO data set (23) encompassed 142 NMIBC (n = 70) and MIBC specimens (n = 72). Gene expression was quantified by the Illumina HumanHT-12 V3.0 expression beadchip microarray. The clinical information included age pathological, overall and tumor-specific survival along with *TP53*, *RAS* and *RB1* somatic mutation status for a subset of patients.
- **GSE48276** GEO data set (23) consisted of 116 bladder cancers (NMIBC: n = 0, MIBC: n = 116), whose transcriptome was gauged with the Illumina HumanHT-12 WG-DASL V4.0 R2 expression beadchip microarray platform. The clinical data included age, sex, race, pathological staging, histological subtype, as well as overall and tumor-specific survival.
- **GSE83586** collective (24) from the GEO repository comprised 307 predominantly advanced bladder cancer samples (NMIBC: n = 58, MIBC: n = 243). Gene expression was determined with an Affymetrix Human Gene 1.0 ST microarray and was accompanied by information on pathological tumor stage and grade.
- **GSE86411** GEO data set (25) consisted of 132 conventional and micropapillary urothelial cancers (no information on invasiveness) with gene expression data obtained with the Illumina HumanHT-12 WG-DASL V4.0 R2 expression beadchip microarray.
- **GSE87304** collective (26) was obtained from GEO and encompassed 305 MIBC specimens. Gene expression was quantified by the Affymetrix Human Exon 1.0 ST microarray. The clinical data included age, bladder cancer molecular subtype, tumor and node pathological stage and treatment information.
- **GSE120736** GEO cohort (27) consisted of 145 samples of primary (n = 118) and recurrent bladder cancers (n = 27). The collective included n = 84 NMIBC and n = 61 MIBC specimens. Whole-genome expression was measured by the Illumina HumanHT-12 V4.0 expression beadchip microarray platform. Gender, histological grade, muscle invasiveness, and pathological tumor stage were provided in the accompanying clinical information.
- **GSE124305** data set (28) was fetched from the GEO portal and encompassed 133 specimens of residual, platinum-resistant (prior cis-platin therapy) MIBC. The samples were retrieved by radical cystectomy. Gene expression was measured by the Affymetrix Human Exon 1.0 ST microarray technology. The adjunct clinical information included gender, pathological tumor stage, and consensus molecular subtype.
- **GSE128192** GEO cohort (29) consisted of 112 sarcomatoid and conventional bladder MIBC. Gene expression was determined by a Illumina HumanHT-12 WG-DASL V4.0 R2 expression beadchip microarray.
- **GSE128701** collective (30) was obtained from GEO and included 136 MIBC samples. Transcriptome was quantified with the Affymetrix Human Exon 1.0 ST microarray and was complemented with information of age, pathological tumor stage, and gender.
- **GSE128959** GEO data set (31) consisted of longitudinally collected samples of primary cancers, recurrent and progressing tumors (total: n = 192, primary: n = 55, recurrent: n = 103, NMIBC - MIBC progression: n = 34, NMIBC: n = 154, MIBC: n = 35). The cohort’s data sets contain n = 42 donor-matched primary and recurrent cancer samples, and n = 25 donor-matched NMIBC - MIBC progression cancer specimens. Gene expression was determined by the Affymetrix Human Gene 1.0 ST microarray. Grade, pathological tumor stage, and LUND2 molecular subtype were provided in the clinical data set of the cohort.
- **GSE198269** GEO data set (32) consisted of 394 platinum-refractory bladder and ureter muscle invasive cancers. Transcriptome was gauged with the Affymetrix Human Exon 1.0 ST microarray. The clinical information included anatomical location and treatment.
- **GSE203149** GEO collective (33) encompassed 171 MIBC samples, whose gene expression was determined with the Affymetrix Clariom S Assay HT microarray.
- **E-MTAB-4321** data (34) set was obtained from the Array Express data portal and included 476 predominantly early stage and muscle non-invasive cancers (NMIBC: n = 460, MIBC: n = 16). Whole-genome expression was measured with the Illumina HiSeq 2000 RNA sequencing platform. The accompanying clinical information consisted of age, gender, pathological tumor stage, histological grade and subtype, as well as information of surgery, prior BCG treatment and time to T2 progression (progression-free survival).
- **Groeneveld 2024** data set information (35) was obtained from the [GitHub repository](https://github.com/csgroen/cit_blca_proteomics/tree/main/analysis/data) accompanying the publication. The cohort consisted of NMIBC (n = 87), MIBC (n = 82) samples and samples without invasiveness information, with transcriptome measured by RNA sequencing (total: n = 198 samples). Additionally, for the same samples, proteome was quantified by LC-MS (liquid chromatography - mass spectrometry). The accompanying information included *TP53*, *FGFR3*, and *RB1* mutation status, copy number data, basic clinical information (sex, age, muscle invasiveness, pathological staging, cystectomy status), and data on overall survival.
- **BCAN** data set (36) of metastatic urothelial cancers (urinary organs: n = 154, non-urinary organs metastasis specimens: 20) was downloaded from cBioportal repository. The RNA sequencing information consisted of normalized Z-scores derived from $log_{2}$ expression signals and was accompanied by clinical information (sex, age, race, smoking, tumor location, pathological staging, survival). The mRNA expression Z-score data set of the BCAN study had missing observation observations, which were imputed by a 9-nearest neighbor regressor prior to analyses (function impute.knn(), package *impute*) (37). Because of its unique gene expression data format (Z-scores), the cohort’s information was not used in all analyses with transcriptome data.

Clinical and pathological characteristic of the investigated patients and cancer samples is provided in **Tables 1** and **2**, **Supplementary Tables S3** and **S4**.

GEO gene expression, annotation and clinical data sets were downloaded with the *GEOquery* package (14). TCGA BLCA, IMvigor, E-MATB-4321, Groeneveld 2024, and BCAN data sets were imported in R with in-house developed scripts.

Normalized gene expression values provided by the authors in form of microarray signals or sequencing reads were transformed with the $log_{2}\left( x \right)$ (microarray) or $log_{2}\left( x+1 \right)$ (RNA sequencing) functions to improve normality. Such expression values are further referred to as $log_{2}$-transformed expression. For clustering, modeling of drug resistance and consensus class assignment, but not for immunedeconvolution, signature calculation or analyses of differential gene expression, $log_{2}$-transformed expression levels of 12394 genes detected in all bulk transcriptome data besides BCAN cohort were adjusted for cohort (batch) effects with the ComBat algorithm (function multi_process(), package [*htGLMNET*](file:///C:\Users\a0073386\Downloads\'https:\github.com\PiotrTymoszuk\htGLMNET'), re-implementation of the genuine algorithm by Leek at al.) (38).
Expression Z-scores in the BCAN collective were neither $log_{2}$-transformed nor ComBat adjusted.

Cancer samples were assigned to UROMOL classes of NMIBC (34) and consensus molecular classes of MIBC (39) by nearest centroid classifiers from packages [*classifyNMIBC*](https://github.com/sialindskrog/classifyNMIBC) and [*consensusMIBC*](https://github.com/cit-bioinfo/consensusMIBC). The classifier functions, classifyNMIBC() and getConsensusClass(), were provided with ComBat-adjusted whole-transcriptome expression. The minimal correlation between the centroid and expression vector was set to minCor = 0.2.

Estimates of fractions of infiltrating stromal and immune cells in bulk cancer transcriptome samples were obtained by immunedeconvolution of the expression data with the QuanTIseq and xCell algorithms (function deconvolute(), package *immunedeconv*) (40–42). Reactome Pathway gene signatures were extracted from a local copy of the [MSig database](https://www.gsea-msigdb.org/gsea/msigdb/index.jsp), version 7.5.1, by a search with the regular expression "^REACTOME_". Gene signatures of proliferating cells were retrieved from seven publications returned by a PubMed search (43–49). Single sample gene enrichment analysis scores (ssGSEA scores) for the gene signatures of Reactome Pathways and proliferation were computed with the genuine GSVA (gene set variation analysis) algorithm by Hänzelmann et al. (50) implemented by calculate() function from [*gseaTools*](https://github.com/PiotrTymoszuk/gseaTools) package.

Somatic mutations were identified in the TCGA BLCA, IMvigor and BCAN cohorts by the Mutect2 (51), FMOne (20) and Caris MI TumorSeek 592-Gene Panel tools, and published by the study authors as a MAF-format file (TCGA, BCAN) or a numeric matrix (IMvigor). In the current project, presence of any non-synonymous and non-silent mutations in a particular gene was coded as 1, while presence of the wild-type allele was coded as 0 (function extract_mutations(), package [*microViz*](https://github.com/PiotrTymoszuk/microViz)). Analogically, for gene deletion data, presence of two gene copies was coded as 0, while homozygous or heterozygous deletion was coded as 1. For gene amplification data, presence of two gene copies was coded as 0, presence of at least one additional gene copy was coded as 1. Training data for modeling of drug resistance with transcriptome information of bulk cancer samples consisted of drug response measures such as IC50 (i.e. concentration at which 50% of cell growth is inhibited) or AUC (area under the curve of dose response) determined in cancer cell lines in vitro and transcriptome measurements in untreated cells. Three training data sets were used for the modeling: GDSC1/2 (52) and CTRP2 (53), and PRISM (54). The training data sets were restricted to cancer cell lines of epithelial origin (e.g. lymphoma or neural-origin cell lines were excluded).

The GDSC1/2 data set contained IC50 determinations from two drug screens, GDSC1 (viability measured by metabolic assays, 327 compounds) and GDSC2 (viability measured with a fluorescent DNA dye, 297 compounds), made in 589 epithelial cancer cell lines with their baseline transcriptomes characterized by RMA-normalized microarray measurements. The GDSC1 and GDSC2 IC50 data were merged and analyzed together. The CTRP2 data set included AUC determinations for 545 compounds in 547 epithelial cancer cell lines of the Cancer Cell Line Encyclopedia, whose basal gene expression was characterized by RNA sequencing and provided in form of RSEM normalized TPM. The PRISM data set contained AUC measurements for 1448 in 398 epithelial cancer cell lines of the Cancer Cell Line Encyclopedia, whose basal expression measurements were shared with the CTRP2 data set. The drug resistance and baseline RNA sequencing data were obtained from the [DepMap repository](https://depmap.org/portal/)(55).

Resistance to anti-cancer drugs was predicted for bulk cancer samples by RIDGE regularized linear regression models trained with the GDSC1/2, CTRP2, and PRISM expression and drug resistance data of epithelial origin cancer cell lines as proposed before (56). The tuning, training, and prediction pipeline used bioinformatic tools of our package [*htGLMNET*](https://github.com/PiotrTymoszuk/htGLMNET), which re-implements the genuine regularized linear regression functions of *glmnet* (57), and was used by us before for predictions of drug resistance in testicular cancer (58). For training of the RIDGE models, 60% of genes with the highest median expression were used. The cell line and bulk cancer gene expression values were batch-corrected with ComBat (function multi_process(), [*htGLMNET*](https://github.com/PiotrTymoszuk/htGLMNET)) (38). The RIDGE models were trained with function train() from [*htGLMNET*](https://github.com/PiotrTymoszuk/htGLMNET) package, which automatically determines the optimal value of the regularization parameter $\lambda$ and performs basic model quality tests via cross-validation. Predictions for the bulk cancer samples were obtained by calling predict() method (package [*htGLMNET*](https://github.com/PiotrTymoszuk/htGLMNET)). Valid predictions used in later analyses were considered for correlation of the predicted and observed drug resistance in the cell culture training data with Superman’s $\rho\geq$ 0.5, and 10-fold cross-validated $R^{2}\geq$ 0.13 (performance statistics computed by summary(), [*htGLMNET*](https://github.com/PiotrTymoszuk/htGLMNET)). In total, such valid predictions were made for 329 compounds with the GDSC1/2 data set, 190 compounds with the CTRP2 data set, and 137 substances with the PRISM data set. Prior to further analyses, the resistance metrics were converted to Z-scores (function zScores(), package [*microViz*](https://github.com/PiotrTymoszuk/microViz)) to facilitate interpretation of the resistance predictions by model trained with three independent drug screening data sets.

Activity of transcriptional regulons and signaling pathway was predicted for bulk cancer transcriptome samples with, respectively, collecTRI (59) and PROGENy knowledge (60) models, and uni- and multivariable linear modeling tools of *decoupleR* package (61). The modeling functions, run_ulm() (univariable modeling, collecTRI) and run_mlm() (multivariable modeling, PROGENy) were fed with $log_{2}$ expression levels for all available genes and collecTRI/PROGENy tables (get_collectri() and get_progeny() functions). The functions returned metrics of regulon and pathway activity in form of linear modeling scores (LM scores).

### Bulk cancer proteome

Bulk cancer proteome data sets were selected by screening the [PubMed portal](https://pubmed.ncbi.nlm.nih.gov/) for studies which measured whole proteome of urothelial cancers by LC-MS (liquid chromatography mass spectrometry) and provided normalized protein counts. One study by Yao et al (62) passed the selection criteria but was excluded from analyses because of the missing assignment of proteome data to clinical information. Three proteome collectives were eligible for analysis:

- **Groeneveld 2024** (35) with 63 specimens (NMIBC: n = 87, MIBC: n = 82) with 3766 proteins quantified. The accompanying clinical information included sex, age, pathological staging, cystectomy status, and overall survival data. As outlined above, the data set included also RNA sequencing information and mutation status for selected oncogenes and tumor suppressor genes. The cohort’s data (normalized protein expression, clinical information) was downloaded from the project’s [GitHub repository](https://github.com/csgroen/cit_blca_proteomics/tree/main/analysis/data) and processed with an in-house developed R script.
- **Dressler 2024** (63) with 242 samples and 9265 proteins quantified. The collective consisted of n = 167 NMIBC samples and n = 75 MIBC specimens. The accompanying clinical information included pathological staging, and overall, progression-free and relapse-free survival. The normalized protein expression and clinical information was extracted from supplementary tables of the publication with a custom R script.
- **Stroggilos 2020** (64) with 117 samples (NMIBC: n = 98, MIBC: n = 19 tumors) and 1600 quantified proteins. The accompanying clinical data included sex, pathological tumor staging, grade, presence of carcinoma in situ, tumor size and multiplicity, squamous differentiation, and EORTC risk strata information. For 16 samples, RNA sequencing data were available as well. The normalized protein expression and clinical information was extracted from supplementary tables of the publication with a dedicated R script.

Protein expression levels were presented and evaluated in the $log_{2}$-transformed form. Missing expression information in the protein expression data sets of the Groeneveld 2024 and Dressler 2024 cohort was imputed with with the 9-nearest neighbor algorithm prior to analyses (function impute.knn(), package *impute*) (37).

ssGSEA scores of protein signatures of Reactome Pathways and signatures of cell proliferation (43–49) were computed with calculate() function ([*gseaTools*](https://github.com/PiotrTymoszuk/gseaTools) package) provided with $log_{2}$ expression data as described above for the bulk transcriptome data sets.

Characteristic of patients and cancer samples in the proteome cohorts are presented in **Tables 1** and **2**, **Supplementary Tables S3** and **S4**.

### DepMap urothelial cancer cell lines

Characteristic, RNA sequencing data, status of gene mutation and copy number, and results of CRISPR-Cas9 knockout (KO) screening for 39 urothelial cancer cell lines (253J [ID: ACH-000011], T24 [ID: ACH-000018], 253JBV [ID: ACH-000026], SLR20 [ID: ACH-000127], CAL29 [ID: ACH-000142], RT4 [ID: ACH-000242], SW780 [ID: ACH-000384], J82 [ID: ACH-000396], RT112 [ID: ACH-000473], KU1919 [ID: ACH-000486], UMUC3 [ID: ACH-000522], VMCUB1 [ID: ACH-000545], HT1197 [ID: ACH-000547], SW1710 [ID: ACH-000566], BC3C [ID: ACH-000593], TCCSUP [ID: ACH-000720], HT1376 [ID: ACH-000724], UBLC1 [ID: ACH-000741], JMSU1 [ID: ACH-000753], BFTC905 [ID: ACH-000802], UMUC1 [ID: ACH-000834], SCABER [ID: ACH-000839], KMBC2 [ID: ACH-000862], 647V [ID: ACH-000896], 5637 [ID: ACH-000905], 639V [ID: ACH-000973], RT11284 [ID: ACH-001183], UMUC13 [ID: ACH-001407], UMUC14 [ID: ACH-001408], UMUC16 [ID: ACH-001409], UMUC4 [ID: ACH-001410], UMUC5 [ID: ACH-001411], UMUC10 [ID: ACH-001412], UMUC11 [ID: ACH-001413], UMUC6 [ID: ACH-001414], UMUC7 [ID: ACH-001415], UMUC9 [ID: ACH-001416], DSH1 [ID: ACH-002235], LB831BLC [ID: ACH-002266]) were obtained from the [DepMap repository](https://depmap.org/portal/)(55). Versions of the data sets used in the current report were 2024 Q2 and later. As presented in **Supplementary Table S51**, for 33 DepMap urothelial cancer cell lines, reliable assignment to our novel bladder cancer clusters was predicted. These cell lines were used in further analyses. The RNA sequencing data, genuinely provided as TPM, were presented and evaluated following $log_{2}\left( x+1 \right)$ transformation.

ssGSEA scores of Reactome Pathway gene signatures for the cell lines were computed with calculate() function (package [*gseaTools*](https://github.com/PiotrTymoszuk/gseaTools)) as described above. The CRISPR-Cas9 KO data in form of Chronos gene effect scores were multiplied by -1, so that positive values correspond to the extend of cell line’s growth inhibition given the gene knockout. Gene effect scores > 0.5 were considered biologically significant (65,66).

### Gene sets

Gene signatures of Reactome Pathways were extracted from a local copy of the [MSig database](https://www.gsea-msigdb.org/gsea/msigdb/index.jsp), version 7.5.1, by a search with the regular expression "^REACTOME_". Gene signatures of proliferating cells were retrieved from seven publications returned by a PubMed search (43–49). Symbols of genes coding for targets for approved and experimental antibody-drug conjugates (ADC) were extracted from publications by Chen et al. and Wang et al. (67,68) (*CA9*, *CD276*, *CDH6*, *CEACAM5*, *CLDN1*, *CLDN18*, *DLK1*, *EGFR*, *EPCAM*, *EPHA5*, *ERBB2*, *ERBB3*, *ERBB4*, *FAP*, *FGFR2*, *FGFR3*, *FOLH1*, *FOLR1*, *ITGB6*, *KIT*, *MET*, *MSLN*, *NECTIN4*, *PTK7*, *ROR1*, *TACSTD2*, *TF*, *TM4SF1*, and *VTCN1*). Markers of neuroendocrine differentiation in urothelial carcinoma were extracted from publications by Da Costa et al. (Table S1 of the article) (69), Akbulut et al. (70), Zhang et al. (71) and Balanis el al. (72) (Table S1, top 30 markers of neuroendocrine bladder cancer), and appended with the top 30 genes with the highest centroid scores in the neuroendocrine-like (NE-like) consensus class of MIBC in R package [*consensusMIBC*](https://github.com/cit-bioinfo/consensusMIBC) (39) (*ACTA2*, *ACTL6B*, *AIRE*, *ALL2*, *APLP1*, *ASCL1*, *C1QC*, *CBFA2T2*, *CD68*, *CENPF*, *CGA*, *CHGB*, *CNIH2*, *COL1A1*, *COL1A2*, *CRMP1*, *CTCFL*, *DACH1*, *DLL3*, *DLX6*, *ENO2*, *FABP5*, *FN1*, *FOXG1*, *FXYD6*, *GNG4*, *HDAC5*, *HES6*, *HMGB2*, *HOXA6*, *HOXD11*, *INSM1*, *ISL1*, *ITPR3*, *KIF1A*, *KPNA2*, *LDHA*, *MSI1*, *MSN*, *MT2A*, *MYL9*, *NEUROD1*, *NEUROD2*, *NKX2-1*, *NKX2-2*, *NUSAP1*, *ONECUT1*, *ONECUT2*, *PEG10*, *PHTF1*, *PLCG2*, *PLEKHG4B*, *PLP2*, *POU3F2*, *POU3F3*, *PTTG1*, *RND2*, *SCRT1*, *SOX2*, *SV2A*, *SYN1*, *SYP*, *TMEM97*, *TOP2A*, *TUBA1A*, *TUBB2A*, *TUBB2B*, *UCHL1*, *ZKSCAN3*, *ZNF250*, *ZNF3*, *ZNF446*, *ZNF470*, *ZNF589*, *ZNF629*, *ZNF7*, and *ZNF74*). PAM50 genes used for definition of PAM50 molecular subsets in the pan-cancer collective of TCGA tumors were extracted from Supplementary Table S1 of the publication by Zhao et al. (18) (*ACTR3B*, *ANLN*, *BAG1*, *BCL2*, *BIRC5*, *BLVRA*, *CCNB1*, *CCNE1*, *CDC20*, *CDC6*, *CDH3*, *CENPF*, *CEP55*, *CXXC5*, *EGFR*, *ERBB2*, *ESR1*, *EXO1*, *FGFR4*, *FOXA1*, *FOXC1*, *GPR160*, *GRB7*, *KIF2C*, *KRT14*, *KRT17*, *KRT5*, *MAPT*, *MDM2*, *MELK*, *MIA*, *MKI67*, *MLPH*, *MMP11*, *MYBL2*, *MYC*, *NAT1*, *NDC80*, *NUF2*, *ORC6L*, *PGR*, *PHGDH*, *PTTG1*, *RRM2*, *SFRP1*, *SLC39A6*, *TMEM45B*, *TYMS*, *UBE2C*, and *UBE2T*).

### Harmonization of clinical and pathological information

Information on muscle invasiveness, if not provided in clinical meta-data accompanying the molecular data of bulk cancer transcriptome and proteome cohorts or available in the genuine publications, was derived from pathological staging with Ta, T1, Tis and CIS stage tumors classified as NMIBC and T2 - T4 specimens as MIBC.

To harmonize highly variable and partly incomplete classification of cancer tissue (primary, recurrent, metastatic) and anatomical location between the bulk cancer cohorts, tissue source was subsumed under “bladder”, “urinary organ, non-bladder”, and “non-urinary organ, distant metastasis” based on clinical/pathological metadata and genuine publications.

Survival outcomes were harmonized as follows:

- death and overall survival: any-cause death during the study follow-up
- tumor death and tumor-specific survival: death due to urothelial cancer or its metastases during the study follow-up
- relapse and relapse-free survival: any recurrence or progression of urothelial cancer after initial treatment during the study follow-up
- MIBC progression: progression of primary NMIBC to MIBC during the study follow-up. Please note that progression of MIBC has not been analyzed.

## Exploratory data analysis, descriptive statistics, hypothesis testing, statistical significance, meta-analyses, and effect sizes

Numbers were rounded to two significant digits. Numeric variables are referenced in the text and tables as medians with interquartile ranges, ranges, and numbers of complete cases. Qualitative and ordinal features are provided as counts of observations and percentages of their categories, and numbers of complete cases. Descriptive statistics for survival data consisted of Kaplan-Meier estimates of survival times for the 25%, 50%, and 75% survival quantiles with their 95% confidence intervals (95% CI). These descriptive statistics were computed with functions explore(), fast_num_stats(), count_binary(), and quantile.survfit() from [*ExDA*](https://github.com/PiotrTymoszuk/ExDA), [*microViz*](https://github.com/PiotrTymoszuk/microViz), and *survival* (73) packages.

Estimates and meta-estimates of differential gene/protein expression, differences in ssGSEA gene/protein signature scores, differences in LM regulon/signaling activity scores and differences in predicted drug resistance metrics obtained by T test, estimates of metabolic reaction activity modeled by Monte Carlo simulations, bootstrapped estimates of gene effects, genetic alteration enrichment scores, and hazard ratio (HR) estimates were presented as estimates of means or HR with 95% CI. The 95% CI were obtained from T or Z statistic distribution (T tests, survival models) or computed with the BCA method (Monte Carlo, bootstrap, genetic alteration enrichment scores) (74).

Distribution and variability statistics of numeric features (mean, variance, median, interquartile ranges, range, mean-variance ratio, Gini coefficients, fraction of observations with unique values) were computed with function distr_stats() from [*microViz*](https://github.com/PiotrTymoszuk/microViz). Normality and equality of variance assumptions were checked prior to T tests and ANOVA with Shapiro-Wilk and Levene tests (functions f_shapiro_test() and f_levene_test(), package [*fastTest*](https://github.com/PiotrTymoszuk/fastTest)).

Statistical significance of differences in levels of numeric variables between two group was determined by two-tailed Student’s and Welch’s T test or Mann-Whitney test with Cohen’s d and biserial r effect size statistics (function compare_variables() from package [*ExDA*](https://github.com/PiotrTymoszuk/ExDA), functions f_t_test(), f_wilcox_test() from [*fastTest*](https://github.com/PiotrTymoszuk/fastTest)). Statistical significance of differences between the cluster’s mean and the cohort’s average value of a numeric feature was tested with one-sample T test with Cohen’s d effect size statistic (avg_deviation(), package [*microViz*](https://github.com/PiotrTymoszuk/microViz)). Statistical significance of differences in levels of numeric variables between three and more groups was investigated by one-way ANOVA or Kruskal-Wallis test with $\eta^{2}$ effect size statistic (functions f_one_anova() and f_kruskal_test() from package [*fastTest*](https://github.com/PiotrTymoszuk/fastTest), function compare_variables() from [*ExDA*](https://github.com/PiotrTymoszuk/ExDA)). Differences in distribution of qualitative variables were assessed by $\chi^{2}$ test with Cramer’s V effect size statistic (f_chisq_test() from package [*fastTest*](https://github.com/PiotrTymoszuk/fastTest), compare_variables() from [*ExDA*](https://github.com/PiotrTymoszuk/ExDA)). Correlations were explored by Pearson’s and Spearman’s permutation tests with r and $\rho$ correlation coefficients (f_cor_test(), package [*fastTest*](https://github.com/PiotrTymoszuk/fastTest)). Differences in survival were assessed by Kaplan-Meier analyses and Peto-Peto tests (surv_pvalue(), package *survminer*) (75). Other statistical hypothesis test specific for particular analytic tasks are described elsewhere in **Supplementary Methods**.

P values were adjusted for multiple testing with the false discovery rate (FDR) (76) separately for each cohort and analysis task (differential expression of genes, proteins and gene signatures, modeling of regulons, signaling, metabolism, gene effects, drug resistance and gene effects, enrichment of gene ontology terms and genetic alterations), or for all p values in an analysis task (clinical information, survival). Except for results of permutation tests for enrichment of genetic alterations and bootstrap modeling of gene effects, differences and estimates with FDR-adjusted p < 0.05 were considered statistically significant. For genetic permutation tests and bootstrapping of gene effects, p values were found to depend strongly on the number of algorithm’s iterations; hence statistically significant enrichment of genetic alterations and statistically significant gene effects were assumed for raw p < 0.05 without multiple testing correction.

Consistency of results of analyses with multi-cohort bulk transcriptome data such as differential gene expression or modeling of metabolic reaction activity, was routinely tested by computing Jaccard’s similarity coefficients J between sets of differentially regulated features (function set_similarity(), package [*microViz*](https://github.com/PiotrTymoszuk/microViz)). The J coefficients measured the fractions of overlapping differentially regulated features between the cohorts. Candidate outliers in such analyses, e.g. collectives with strongly dissimilar sets of differentially regulated genes or metabolic reactions, were identified by a visual inspection of scatter plots of multi-dimensional scalings (MDS) of the Jaccard’s distance matrices (function plot.set_sim(type = "mds"), package [*microViz*](https://github.com/PiotrTymoszuk/microViz)).

While interpreting analysis results with bulk cancer transcription and proteome cohorts, we focused on effects shared by, respectively, at least 10 and at least two cohorts. For such common regulated features, meta-estimates of regulation (e.g. differential gene expression) were computed with the DerSimonian-Lair inverse variance algorithm assuming non-equal distribution of between-cohort variances (77). The meta-estimate-calculating function f_meta() was provided with estimates of interest with their errors (e.g. $log_{2}$ fold-change estimated of differential gene expression with their standard errors).

Effect size of differences in variable levels or distribution, correlation, relative risk, and model fit was assessed with the following metrics:

- biserial r for differences between two groups, non-parametric tests (78): small for r < 0.2, medium for 0.2 $\leq$ r < 0.3, large for r $\geq$ 0.3
- Cohen’s d for differences between two groups, parametric tests (79): small for d < 0.5, medium for 0.5 $\leq$ d < 0.8, large for d $\geq$ 0.8
- $\eta^{2}$ for differences between three or more groups (80): small for $\eta^{2}$ < 0.06, moderate for 0.06 $\leq$ $\eta^{2}$ < 0.14, large for $\eta^{2}$ $\geq$ 0.14
- Cramer’s V for comparison of category frequencies of a categorical variable (78): small for r < 0.2, medium for 0.2 $\leq$ r < 0.3, large for r $\geq$ 0.3
- r and $\rho$ correlation coefficients (79): small for r or $\rho$ < 0.3, medium for 0.3 $\leq$ r or $\rho$ < 0.5, large for r or $\rho$ $\geq$ 0.5
- odds ratio (OR) for enrichment and relative probability (79): very small for OR < 1.44, small for 1.44 $\leq$ OR < 2.48, moderate for 2.48 $\leq$ OR < 4.27, large for OR $\geq$ 4.27
- $R^{2}$ for explained variance of a model fit or prediction (79): weak for $R^{2}$ < 0.13, moderate for 0.13 $\leq$ $R^{2}$ < 0.26, large for $R^{2}$ $\geq$ 0.26
- Cohen’s $\kappa$ for concordance between the model-predicted and observed class assignment (81,82): minimal for $\kappa$ < 0.2, weak for 0.2 $\leq$ $\kappa$ < 0.6, moderate for 0.6 $\leq$ $\kappa$ < 0.8, strong for $\kappa$ $\geq$ 0.8
- area under the curve (AUC), receiver-operating characteristic (ROC) for identification of the outcome or particular class (e.g. cancer sample cluster) (83): weak for AUC < 0.556; moderate for 0.556 $\leq$ AUC < 0.714, strong for AUC $\geq$ 0.714
- Harrell’s concordance index (C) for concordance between the model-predicted and observed survival (83): weak for C < 0.556; moderate for 0.556 $\leq$ C < 0.714, strong for C $\geq$ 0.714

## Development and technical validation of bladder cancer clusters

Bladder cancer clusters were defined in the TCGA BLCA cohort bulk cancer transcriptome data set with a combined procedure involving selection of variably expressed genes, definition of metagenes, computation of metagene’s ssGSEA scores, and semi-supervised regularized clustering of metagene’s ssGSEA scores. For technical validation of the clustering scheme, IMvigor, GSE13507, GSE32548, GSE48075, GSE48276, GSE83586, GSE86411, GSE87304, GSE120736, GSE124305, GSE128192, GSE128701, GSE128959, GSE198269, GSE203149, E-MTAB-4321, and Groeneveld 2024 bulk cancer transcriptome cohorts were used.

### Pre-processing and selection of variably expressed genes

A total of 12394 genes were shared by 18 bulk cancer transcriptome cohorts used in development and technical validation of bladder cancer clusters. $log_{2}$ expression values of these common genes were adjusted for batch/cohort effects with the ComBat algorithm (function multi_process(), package [*htGLMNET*](https://github.com/PiotrTymoszuk/htGLMNET)). For definition of metagenes, genes with substantial variability within each of the bulk transcriptome cohorts were selected. Our motivation was to exclude genes with negligible expression and house-keeping genes, whose expression tends to be constant between cancer samples. By a visual inspection of density curves of Gini coefficients (computed with distr_stats(), package [*microViz*](https://github.com/PiotrTymoszuk/microViz)), we selected 3234 genes with Gini coefficients of the ComBat-adjusted $log_{2}$ expression levels exceeding 0.07 in all cohorts for the metagene definition (**Supplementary Figure S1**, **Supplementary Tables S5** and **S6**). Prior to metagene definition, the gene expression values were converted to median-centered Z-scores (function center_data(), package [*clustTools*](https://github.com/PiotrTymoszuk/clustTools)).

### Definition of metagenes

Metagenes were defined as micro-clusters of tightly co-regulated transcripts identified with the self-organizing map (SOM) algorithm (84–86). SOM structures with dimensions of 16 $\times$ 16 nodes and hexagonal unit shapes, which employed popular gene - node distance measures (Euclidean, Manhattan, squared Euclidean, cosine distance) and plain or toroidal topology were constructed in a training subset of the TCGA BLCA data set (n = 305 randomly chosen cancer samples, 75% of total). The algorithm operated in the batch mode with automatic selection of the learning rate (85), Gaussian neighborhood function, and a maximum of 3000 iterations. The SOM objects were built with function som_cluster() from [*clustTools*](https://github.com/PiotrTymoszuk/clustTools) package. Convergence of the algorithm was assessed by plotting the mean distance between the genes and the SOM nodes for subsequent iterations (method plot() called for SOM objects, [*clustTools*](https://github.com/PiotrTymoszuk/clustTools)). As inferred from a plateau of the mean distance between the nodes and observation, the convergence was reached within the first 2800 iterations (not shown).

Prediction of the metagene assignment for the test portion of the TCGA BLCA cohort, i.e. remaining n = 102 observations not used by SOM, was done with predict(type = "class") method (package [*clustTools*](https://github.com/PiotrTymoszuk/clustTools)). Performance of the metagene schemes in the training and test portions of the TCGA BLCA collective was assessed by neighborhood preservation (mean fraction of the five nearest neighbor genes in the genuine data assigned to the same metagene, function np(), package [*clustTools*](https://github.com/PiotrTymoszuk/clustTools)) (87), and fraction of explained clustering variance (ratio of the between-cluster sum of squares to the total total sum of squares, method var(), package [*clustTools*](https://github.com/PiotrTymoszuk/clustTools)). Additionally, for the training portion of the TCGA BLCA collective, topological errors were computed, i.e. mean fractions of the neighbor nodes in the initial SOM that remain neighbors after the complete algorithm run (84,85) (function te(), [*clustTools*](https://github.com/PiotrTymoszuk/clustTools)). These performance statistics are summarized in **Supplementary Figure S1BC** and **Supplementary Table S5**.

In general, all tested SOM variants demonstrated a similar performance and a high level of consistence of performance metrics between the training and test portion of the TCGA BLCA data. For definition of the final metagene scheme, the toroidal SOM operating with squared Euclidean distance was selected. The gene - metagene assignment was extracted from the SOM object via predict(type = "class") (package [*clustTools*](https://github.com/PiotrTymoszuk/clustTools)). Assignment of the metagene-defining genes to metagenes along with their biological characteristic resolved by a gene ontology (GO) enrichment analysis (function GOana(), package [*microViz*](https://github.com/PiotrTymoszuk/microViz)) (88) is presented in **Supplementary Table S6**.

### Semi-supervised clustering of cancer samples by metagene expression

ssGSEA scores of the metagenes were computed for bulk cancer transcriptome samples in the TCGA BLCA collective and remaining 17 validation cohorts with the gene set variation analysis method by calling calculate() function from [*gseaTools*](https://github.com/PiotrTymoszuk/gseaTools) package (50). Clusters of cancer samples were developed by semi-supervised clustering of the bulk cancer transcriptome specimens in the TCGA BLCA cohort by their normalized, mean-centered metagene ssGSEA scores. For clustering, the novel hard-threshold regularized KMEANS algorithm (HTKmeans) was used. In brief, this procedure allows for removal of features (in our case: metagenes) that are unlikely to contribute to a meaningful clustering scheme with a distance threshold defined by the shrinkage parameter $\lambda$. As such, this procedure is expected to yield more stable and reproducible clustering solutions in a multi-dimensional setting, i.e. where the number of candidate clustering features is similar or greater than the number of observations, as compared with canonical clustering procedures such as KMEANS (89).

Choice of the optimal number of cluster (k) and the optimal $\lambda$ value was motivated by a trade-off of the maximal clustering variance (ratio of between-cluster sum of squares to the total sum of squares), maximal mean silhouette width (90) and maximal neighborhood preservation (mean fraction of the five nearest neighbor observations placed in the same cluster) (87), and the minimal potential misclassification rate (fraction of observations with negative silhouette statistics) (90) in the entire TCGA BLCA cohort and for out-of-fold cluster predictions in 5-fold cross-validation (91) (functions summary() and cv(), package [*clustTools*](https://github.com/PiotrTymoszuk/clustTools)). These performance statistics for k = 2 to k = 6 clustering solutions in the entire TCGA BLCA data set and five-fold cross-validation, and the process of $\lambda$ selection are presented in **Supplementary Figure S2** and **Supplementary Table S7**. The clustering solution with k = 3 clusters was characterized by a high clustering variance and a low potential misclassification rate, and was hence selected for definition of bladder cancer clusters #1, #2 and #3.

Cancer samples in the validation bulk cancer transcriptome cohorts were assigned to bladder cancer clusters trained in the TCGA BLCA cohort with an inverse distance weighted k-nearest neighbor classifier (92) operating with normalized (z-Scores) ssGSEA scores of the metagenes (function prediter(), package [*clustTools*](https://github.com/PiotrTymoszuk/clustTools)). Assignment of the bulk cancer transcriptome specimens to bladder cancer clusters is presented in **Supplementary Table S8**.

### Technical validation of bladder cancer clusters in bulk cancer transcriptome samples

Technical validation of bladder cancer clusters included visual inspection of the cluster embeddings as well as comparison of cluster sizes, performance statistics, and similarity measures in the TCGA BLCA and bulk cancer validation cohorts.

Clustering structures were visualized and visually assessed by representing two-dimensional UMAP (uniform manifold approximation and projection) embeddings of normalized ssGSEA scores of the metagenes in scatter plots (functions componentns(red_fun = "umap") and plot(), package [*clustTools*](https://github.com/PiotrTymoszuk/clustTools)). Such plots were generated for pooled muscle invasive (MIBC) and non-muscle invasive bladder cancers (NMIBC), pooled collectives of cancers with and without history of BCG (Bacillus Calmette Guerin) therapy, pooled collectives of cancers with and without history of neoadjuvant systemic chemotherapy, pooled collectives of cancers of the bladder and non-bladder urinary organs, as well as for single bulk transcriptome cohorts (**Figure 2A**, **Supplementary Figure S3A** and **S5**).

For the TCGA BLCA cohort used for training of bladder cancer clusters and the remaining 17, numeric statistics of explained clustering variance (ratio of the between-cluster sum of squares to the total sum of squares), cluster separation (mean silhouette width), fraction of potentially misclassified observations (fraction of observation with negative silhouette values) (90), and neighborhood preservation (mean fraction of the five nearest neighbor observations placed in the same cluster) (87) were computed with summary() method (package [*clustTools*](https://github.com/PiotrTymoszuk/clustTools)). These performance statistics are presented in **Supplementary Figure S3B** and **Supplementary Table S9**. They point towards good reproducibility of the clustering structures in most validation cohorts, which was however better in collectives with high fraction of MIBC (e.g. GSE48276 or Imvigor) than in predominantly NMIBC cohorts (e.g. E-MTAB-4321).

Fractions of the samples in bladder cancer clusters were compared between the pooled MIBC and NMIBC collectives (**Figure 2B**), and between single cohorts (**Supplementary Figure S4A**). This analysis revealed high degree of consistency of distribution of the cluster sizes between the bulk transcriptome data sets. Of note, fraction of cluster #3 cancers was significantly higher in pooled NMIBC collective as compared with the MIBC pool ($\chi^{2}$ test, Cramer’s V effect size).

Values of ssGSEA scores of the metagenes were compared between the clusters by FDR-corrected Kruskal-Wallis test and $\eta^{2}$ effect size statistic. Differentially regulated metagenes were defined by pFDR < 0.05 and $\eta^{2}$ $\geq$ 0.06, which corresponds to significant, moderate-to-strong difference in metagene levels between the clusters (function f_kruskal_test(), package [*fastTest*](https://github.com/PiotrTymoszuk/fastTest)). Out of 256 metagenes used for bladder cluster definition, 213 features were found to be significantly differentially regulated between the clusters in at least 10 cohorts (**Supplementary Figure S4B**). This suggests prominent and highly reproducible differences in cluster-defining features between, and, hence, good separation of bladder cancer clusters.

To assess separation of bladder cancer clusters with an independent method, we computed squared Euclidean distances between the cluster pairs in respect to ssGSEA scores of the metagenes for each of the cohorts. Similarity of the corresponding clusters in the TCGA BLCA cohort and in the validation cohorts was assessed by squared Euclidean cross-distances. The distances were computed with cross_distance() function from [*clustTools*](https://github.com/PiotrTymoszuk/clustTools) package and depicted as bubble plots in **Supplementary Figure S4CD**. Results of these analyses demonstrate high degree of similarity of the corresponding clusters in the training and validation cohorts. Separation of the non-corresponding clusters was substantially better for cluster #3 and the reaming clusters than between clusters #1 and #2.

## Assignment of bulk cancer proteomes, BCAN transcriptome specimens, and DepMap urothelial cancer cell lines to bladder cancer clusters

Bulk cancer proteomes in the Groeneveld 2024, Dressler 2024, and Stroggilos 2020 collectives, bulk cancer transcriptome samples in the BCAN cohort, and DepMap urothelial cancer cell lines were assigned to bladder cancer clusters by multinomial Elastic Net classifiers (57,93) trained in the TCGA BLCA clusters.

The optimal values of regularization/shrinkage parameter $\lambda$ for the Elastic Net models were found by minimizing the model’s deviance in repeated cross-validation. The lambda finding and model training process was automated by function tune_glmnet() from [*htGLMNET*](https://github.com/PiotrTymoszuk/htGLMNET) package. Non-zero coefficients of the Elastic Net models, i.e. weights for expression values of genes, which contribute to the cluster assignment prediction, were extracted from the model objects by coef() function (package [*htGLMNET*](https://github.com/PiotrTymoszuk/htGLMNET)) and are listed in **Supplementary Table S11**. Prior to making predictions, the models’ accuracy (accuracy and Cohen’s $\kappa$ statistics) (81), mean sensitivity/specificity at bladder cluster detection (area under the receiver-operating characteristic [AUC]), and confidence/calibration (log-loss and Brier score metrics; low values are characteristic for well calibrated and confident predictions) (94) were evaluated in transcriptomic bulk cancer validation cohorts (IMvigor, GSE13507, GSE32548, GSE48075, GSE48276, GSE83586, GSE86411, GSE87304, GSE120736, GSE124305, GSE128192, GSE128701, GSE128959, GSE198269, GSE203149, E-MTAB-4321, and Groeneveld 2024). In this evaluation, the metagene-based bladder cluster assignment served as the ground truth. These performance statistics were computed with a wrapper around multiClassSummary() function (package *caret*) (95) and are listed in **Supplementary Table S13**. Cluster assignment probabilities and cluster assignment obtained by nodal voting were obtained by calling predict(type = "response") and predict(type = "class") for the Elastic Net model objects and numeric matrices of predictors (gene or protein expression values) in the proteome cohorts, BCAN collective, or DepMap cell line collection. Details of the Elastic Net models and predictions specific for the proteomic, BCAN, and cell line data are outlined below.

### Bulk cancer proteome samples

For subsets of the Groeneveld 2024 and Stroggilos 2020 patients, both RNA sequencing and proteome data were available. We made use of these data for selection of predictors for the proteome Elastic Net classifier. Such predictors were defined as genes whose transcript and protein levels correlated significantly with r $\geq$ 0.5 (Pearson’s permutation test, function f_cor_test(), package [*fastTest*](https://github.com/PiotrTymoszuk/fastTest), **Supplementary Table S12**).

Transcript expression levels in the TCGA BLCA cohort ($log_{2}\left( TPM+1 \right)$) and protein expression levels in the Groeneveld 2024, Dressler 2024, and Stroggilos 2020 cohorts ($log_{2}$ protein counts) were adjusted for batch/cohort effects with the ComBat algorithm (function multi_process(), package [*htGLMNET*](https://github.com/PiotrTymoszuk/htGLMNET)). These expression values were used as the bladder cancer cluster predictors by the Elastic Net model trained in the TCGA BLCA cohort.

The proteome assignment model had a moderate to high accuracy (accuracy: 0.77 to 0.93, Cohen’s $\kappa$: 0.62 to 0.89), as well as high mean sensitivity/specificity (AUC: 0.91 to 0.99) in the validation bulk cancer transcriptome cohorts. Brier scores ranged between 0.12 to 0.32.

Assignment of the proteome samples to bladder cancer clusters is presented in **Supplementary Tables 14**. UMAP embeddings of the ComBat adjusted $log_{2}$ expression levels of proteins used by the Elastic Net model to assign proteome samples to bladder cancer clusters (non-zero coefficient predictors, UMAP computed with reduce_data(), package [*clustTools*](https://github.com/PiotrTymoszuk/clustTools)) are visualized in **Figure 2A**. Sizes of bladder cancer clusters in pooled MIBC and NMIBC collectives of bulk cancer transcriptomes and bulk cancer proteomes were compared in **Figure 2B**. Similar to the transcriptome specimens, cluster #3 was significantly larger in NMIBC proteomes as compared with MIBC proteomes ($\chi^{2}$ test with Cramer’s V effect size, function f_chi_test(), package [*fastTest*](https://github.com/PiotrTymoszuk/fastTest)).

### BCAN bulk transcriptome samples

Expression levels of all genes shared between the TCGA BLCA ($log_{2}\left( TPM+1 \right)$) and BCAN (Z-scores) cohorts were adjusted for batch/cohort effects with the ComBat algorithm (function multi_process(), package [*htGLMNET*](https://github.com/PiotrTymoszuk/htGLMNET)). These gene expression values were used as the bladder cancer cluster predictors by the Elastic Net model trained in the TCGA BLCA cohort.

The BCAN assignment model had a moderate to high accuracy (accuracy: 0.81 to 0.95, Cohen’s $\kappa$: 0.67 to 0.92), as well as high mean sensitivity/specificity (AUC: 0.95 to 1) in the validation bulk cancer transcriptome cohorts. Brier scores ranged between 0.093 to 0.26.

Assignment of the BCAN cohort specimens to bladder cancer clusters is presented in **Supplementary Tables 14**.

### DepMap urothelial cancer cell lines

Choice of predictors for the Elastic Net model of bladder cancer clusters to be used for assignment of cell lines was motivated by an expression criterion. Because urothelial cancer cell lines are unlikely to abundantly express genes associated with non-malignant cells in bulk cancer samples (e.g. markers of immune cells or endothelium), we restricted the candidate predictor pool to 6897 genes expressed in both in the TCGA BLCA cohort and the DepMap collection with a median of at least 5 TPM. Expression levels of these genes in the TCGA BLCA ($log_{2}\left( TPM+1 \right)$) and DepMap cell lines ($log_{2}\left( TPM+1 \right)$) cohorts were adjusted for batch/cohort effects with the ComBat algorithm (function multi_process(), package [*htGLMNET*](https://github.com/PiotrTymoszuk/htGLMNET)). These gene expression values were used as the bladder cancer cluster predictors by the Elastic Net model trained in the TCGA BLCA cohort.

The cell line assignment model had a moderate to high accuracy (accuracy: 0.77 to 0.91, Cohen’s $\kappa$: 0.65 to 0.87), as well as high mean sensitivity/specificity (AUC: 0.93 to 0.99) in the validation bulk cancer transcriptome cohorts. Brier scores ranged between 0.15 to 0.32.

Reliable assignment of cell lines to bladder cancer clusters was assumed for cluster assignment probability p > 0.5. A total of 33 cell lines passed this criterion (cluster #1: n = 13, #2: n = 7, #3: 13 cell lines, **Supplementary Table S51**). Multi-dimensional scaling (MDS) of the ComBat adjusted $log_{2}$ expression levels of genes used by the Elastic Net model to assign the DepMap cell lines to bladder cancer clusters (non-zero coefficient predictors, MDS computed with reduce_data(), package [*clustTools*](https://github.com/PiotrTymoszuk/clustTools)) is visualized in **Figure 7A**. Cell lines representative for clusters #1 (UMUC3), #2 (5637), and #3 (RT112) were used in in vitro treatment experiments with selected anti-cancer drugs (**Figure 7E**).

## Correlation with cluster centroids and similarity of bladder cancer clusters in bulk cancer transcriptome, bulk cancer proteome, and DepMap urothelial cancer cell line data

As an additional measure to assess similarity of bladder cancer clusters in the bulk cancer transcriptomes, bulk cancer proteomes, and DepMap urothelial cell lines, we computed Spearman’s coefficients $\rho$ of correlations with centroids of the clusters in the TCGA BLCA training cohort. The centroids were defined as mean expression levels of the clustering features, i.e. metagenes of genes/proteins used by Elastic Net classifiers, in bladder clusters #1, #2, and #3 in the TCGA BLCA collective. For the bulk cancer transcriptomes assigned to the clusters with the k-nearest neighbor classifier (cohorts: IMvigor, GSE13507, GSE32548, GSE48075, GSE48276, GSE83586, GSE86411, GSE87304, GSE120736, GSE124305, GSE128192, GSE128701, GSE128959, GSE198269, GSE203149, E-MTAB-4321, and Groeneveld 2024), the correlations with centroids were computed for the data sets of ssGSEA scores of the metagenes listed in **Supplementary Table S6**. For the bulk cancer transcriptomes in the BCAN cohort, bulk cancer proteomes in the Groeneveld 2024, Dressler 2024 and Stroggilos 2020 cohort, and DepMap urothelial cell lines, the correlations were calculated in the data sets of Z-score expression levels and ComBat-processed $log_{2}$ expression levels used by the Elastic Net classifiers of bladder cancer clusters and listed in **Supplementary Table S11**. The correlation coefficients were computed with a dedicated R wrapper around function cor() from *stats* package. The coefficients of correlation with the centroids are provided in **Supplementary Tables S8**, **S14**, and **S51**.

Matrices of Average correlations with the cluster centroids in the cohort’s and DepMap cell lines were additionally visualized as scatter plots of two-dimensional multi-dimensional scaling (MDS) embeddings in **Supplementary Figure S6** (functions reduce_data(red_fun = "mds") and plot(), [*clustTools*](https://github.com/PiotrTymoszuk/clustTools)).

## Bladder cancer clusters, UROMOL classes of NMIBC, and consensus classes of MIBC

Bulk cancer transcriptome samples in the TCGA BLCA, IMvigor, GSE13507, GSE32548, GSE48075, GSE48276, GSE83586, GSE87304, GSE120736, GSE124305, GSE128192, GSE128701, GSE128959, GSE198269, GSE203149, Groeneveld 2024, and BCAN were assigned to UROMOL classes of NMIBC (34) (NMIBC samples) and to consensus classes of MIBC (MIBC samples) (39) as described in **Data sources**. Distribution of UROMOL classes of NMIBC and consensus classes of MIBC in bladder cancer clusters #1, #2, and #3 for pooled collectives of NMIBC and MIBC samples was presented in stack plots in **Figure 2C**. Frequencies of UROMOL classes of NMIBC and consensus classes of MIBC in single cohorts are summarized in **Supplementary Table S15**.

## Makers of bladder cancer clusters shared by bulk cancer transcriptome, bulk cancer proteome, and urothelial cancer cell lines

Markers of bladder cancer clusters in bulk cancer transcriptome data (cohorts: TCGA BLCA, IMvigor, GSE13507, GSE32548, GSE48075, GSE48276, GSE83586, GSE86411, GSE87304, GSE120736, GSE124305, GSE128192, GSE128701, GSE128959, GSE198269, GSE203149, E-MTAB-4321, Groeneveld 2024, and BCAN), bulk cancer proteome data (cohorts: Groeneveld 2024, Dressler 2024, and Stroggilos 2020), and DepMap urothelial cancer cell lines were identified by a two-step procedure. First, $log_{2}$ expression values for all available genes/proteins were compared between the clusters with FDR-corrected one-way ANOVA with $\eta^{2}$ effect size metric (function f_one_anova(), package [*fastTest*](https://github.com/PiotrTymoszuk/fastTest)). Significant differences in expression of at least moderate effect size were defined by pFDR < 0.05 and $\eta^{2}$ $\geq$ 0.06. Subsequently, for the genes/proteins with significant differences between bladder cancer clusters, receiver-operating characteristic (ROC) analysis was performed in a cluster versus rest manner (function classify(), package [*microViz*](https://github.com/PiotrTymoszuk/microViz)). Significant markers of a particular cluster were considered for area under the ROC curve AUC $\geq$ 0.714 as proposed for strong markers identified in ROC analyses by Rice et al. (83).

Average Z-scores of $log_{2}$ expression values of bladder cancer cluster markers shared by at least 10 bulk cancer transcriptome cohorts and at least two bulk cancer proteome collectives are shown in heat maps and box plots in **Supplementary Figures S7** and **S9A**. Average Z-scores of $log_{2}$ expression values of bladder cancer cluster markers shared by at least 10 bulk cancer transcriptome cohorts and the DepMap cell line collection are presented in **Supplementary Figure S8** and **S9B**.

## Demographic, clinical, and pathological characteristic of bladder cancer clusters

Demographic (e.g. age, sex, smoking history), clinical (e.g. cancer therapy types), and pathological (e.g. staging, tumor grade) characteristics were compared between bladder cancer clusters in single bulk cancer transcriptome and bulk cancer proteome cohorts, and pooled collectives of NMIBC and MIBC (**Figure 3A**, **Table 3**, **Supplementary Figure S10**, **Supplementary Table S16** - **S19**). Statistical significance for numeric features was determined by Kruskal-Wallis test with $\eta^{2}$ effect size metric. Statistical significance for categorical variables was assessed by $\chi^{2}$ test with Cramer’s V effect size statistic. P values were corrected for multiple testing with the FDR method. Statistical hypothesis tests were conducted with function compare_variables() from [*ExDA*](https://github.com/PiotrTymoszuk/ExDA).

## Survival in bladder cancer clusters

Overall, tumor-specific, relapse-free, and progression-free survival information available for the TCGA BLCA, IMvigor, GSE13507, Groeneveld 2024, BCAN, Dressler 2024, and E-MTAB-4321 collectives. Survival analyses were conducted for four subsets in each of these cohorts: all patients, patients with Ta - T3 cancers, NMIBC and MIBC patients; subsets with less than 80 individuals with complete survival information or with less than 5 survival events were excluded. In the GSE13507 collective with patient-matched specimens of primary, recurrent, and progressing cancers, the bladder cancer cluster assignment of the primary cancer was used as the independent variable in the survival analyses.

Survival in bladder cancer clusters was assessed by Kaplan-Meier analyses and Peto-Peto tests (functions surv_fit(), quantile.survfit() and surv_pvalue(), packages *survminer* and *survival*) (73,75). Survival curves were generated with ggsurvplot() function from *survminer* package. The analysis results are presented in **Figure 3B** and **Supplementary Table S20**.

Risk of death, tumor-specific death, relapse, and progression in bladder cancer clusters #1 and #2 as compared with cluster #3 was modeled by univariable Cox proportional hazard regression (functions coxph() and as_coxex(), packages *survival* and [*coxExtensions*](https://github.com/PiotrTymoszuk/coxExtensions)). The proportional hazard assumption was checked with summary(type = "assumptions") method, which internally implements the *zph* algorithm by Grambsch et. al. (96). Inference statistics, i.e. Cox proportional hazard model coefficients with 95% CI, were extracted from the models with summary(type = "inference") method. The coefficients of the univariable Cox proportional hazard models expressed as hazard ratios are depicted in Forest plots in **Supplementary Figure S11**.

In order to assess, if bladder cancer clusters are independent predictors of death risk, uni- and multi-parameter Cox proportional hazard models of overall survival with bladder cancer clusters, clinical predictors, and bladder cancer clusters along with clinical predictors were constructed in the, TCGA BLCA (observations: n = 345, events: 152; clinical predictors: age, sex, pathological tumor stage, pathological node stage), GSE13507 (observations: n = 163, events: 67; clinical predictors: age, sex, pathological tumor stage, pathological node stage), Groeneveld 2024 mRNA (observations: n = 114, events: 43; clinical predictors: age, sex, pathological tumor stage, pathological node stage), and Dressler 2024 proteome cohort (observations: n = 242, events: 109; clinical predictors: pathological tumor stage). The models were constructed with functions coxph() and as_coxex() from packages survival (73) and [*coxExtensions*](https://github.com/PiotrTymoszuk/coxExtensions), the proportional hazard assumption was checked as described for the univariable Cox proportional hazard models. Performance of the bladder cancer-only, clinical predictor-only, and combined cluster/clinical predictor Cox proportional hazard models was compared with the following numeric statistics: (1) accuracy of the predicted risk and actual was quantified by Harrell’s concordance index (97), (2) model confidence was metered by integrated Brier score (IBS; low values are expected for a highly confident model) (98), and (3) explained survival variance was measured by $R^{2}$ statistic (99). The performance statistics were retrieved from the models with function summary(type = "fit") from [*coxExtensions*](https://github.com/PiotrTymoszuk/coxExtensions) package. Model errors for unique follow-up time points expressed as Brier scores (squared difference between the predicted death risk and 0/1-coded death status) (94,98) were computed with function surv_brier() from [*coxExtensions*](https://github.com/PiotrTymoszuk/coxExtensions) package. Inference statistics for the multi-parameter models were computed as described above for the univariable models. Statistical significance of the bladder cancer cluster term in the cluster/clinical predictor models was assessed by comparing the full models with the bladder cluster-only models with likelihood ratio test (LRT; function anova(), *survival* package) (73). Results of the multi-parameter modeling of overall survival are presented in **Supplementary Figures S12** and **S13**.

## Evolution of bladder cancer clusters during tumor recurrence and MIBC progression

Longitudinal patient-matched bulk cancer transcriptome samples of primary tumors, recurrent tumors, as well as primary NMIBC and progressing MIBC were available in the GSE13507 and GSE128959 cohorts, which allowed for comparison of evolving cluster assignment during cancer recurrence and NMIBC - MIBC progression. Changes of the bladder cancer cluster assignment were visualized with alluvial plots (functions from *ggalluvial* package) (100) in **Figure 3C** and as heat maps of transition matrices in **Supplementary Figure S14**.

## Genetic landscape of bladder cancer clusters

Detailed genome-scale gene mutation data were provided for the TCGA BLCA (n = 1.7003^{4} genes). For the IMvigor and BCAN cohort, mutations status for, respectively, n = 561 and n = 393 genes was available. For the GSE48075 and Groeneveld 2024 cohort, information on mutations in *TP53*, *RAS*, *RB1*, and *FGFR3* genes was provided. Whole genome level copy number data were available for the TCGA BLCA and Groeneveld 2024 collectives. The gene mutation, amplification, and deletions data were analyzed as binary variables as outlined in **Data sources**.

Total numbers of mutated, amplified, and deleted genes per sample were compared between bladder cancer clusters of the TCGA BLCA, IMvigor, BCAN, and Groeneveld 2024 cohorts by FDR-corrected Kruskal-Wallis test with $\eta^{2}$ effect size statistic (function f_kruskal_test(), package [*fastTest*](https://github.com/PiotrTymoszuk/fastTest), **Supplementary Figure S15**).

Enrichment of mutations, amplifications, and deletions in bladder cancer clusters of the TCGA BLCA, Groeneveld 2024, IMvigor, and BCAN cohorts was investigated by permutation tests with perTest() function from [*perich*](https://github.com/PiotrTymoszuk/perich) as described in our recent work (101). In brief, the observed frequency of mutation/amplification/deletion in a cluster was compared with a frequency expected at random. The random frequency estimates were obtained from 10000 permutations weighted by overall mutation, amplification, or deletion frequency in the samples. By the weighting, the permutation procedure accounts for higher probabilities of random genetic alterations expected for samples with genetically unstable phenotype manifested by accumulation of mutations and copy number variants. Magnitude of the enrichment was measured by an enrichment score (ES) defined as a ratio of the observed and expected counts of events averaged over the permutations.
Because the enrichment p values (i.e. probability that the frequency of a mutation in the cluster is not higher than expected) depended strongly on the number of permutations, we defined significant enrichment by raw p < 0.05 without any adjustment for multiple testing and ES $\geq$ 1.2. The testing results for amplifications and deletions did not yielded biologically relevant gene variants in any of the clusters, which were shared by the TCGA BLCA and Groeneveld 2024 cohorts (not shown). The testing results for mutations found to be significantly enriched in at least one bladder cancer cluster in the TCGA BLCA, IMvigor, and BCAN cohorts are listed in **Supplementary Table S22**. Such significant mutations in genes with the highest overall mutation rates in the TCGA BLCA, IMvigor, and BCAN cohorts are presented in oncoplots (function plot_bionco(), package [*microViz*](https://github.com/PiotrTymoszuk/microViz)) in **Figure 4A** and **Supplementary Figure S16**.

Additionally, we compared rates of mutations of *FGFR3*, *TP53*, and *RB1* genes between bladder cancer clusters in the TCGA BLCA, IMvigor, BCAN, Groeneveld 2024, and GSE48075 cohort by FDR-adjusted $\chi^{2}$ test with Cramer’s V effect size statistic (function f_chi_test(), package [*fastTest*](https://github.com/PiotrTymoszuk/fastTest)). Results of this analysis are shown in **Supplementary Figure S17**.

## Differential regulation of transcriptome and proteome in bladder cancer cancer clusters

$log_{2}$-transformed of Z-scores (BCAN cohort only) expression levels of genes and proteins were compared between bladder cancer clusters of bulk cancer transcriptomes (cohorts: TCGA BLCA, IMvigor, GSE13507, GSE32548, GSE48075, GSE48276, GSE83586, GSE86411, GSE87304, GSE120736, GSE124305, GSE128192, GSE128701, GSE128959, GSE198269, GSE203149, E-MTAB-4321, Groeneveld 2024, and BCAN), bulk cancer proteomes (cohorts: Groeneveld 2024, Dressler 2024, and Stroggilos 2020), and DepMap urothelial cell lines.

Differentially regulated genes and proteins were identified by a two-step procedure. In the first step, differences between the clusters were assessed by FDR-corrected one-way ANOVA with $\eta^{2}$ effect size statistic (function f_one_anova(), package [*fastTest*](https://github.com/PiotrTymoszuk/fastTest)). In the second step, differences in expression between the cluster and the cohort’s average were assessed by one-sample two-tailed T test, which returned also estimates of $log_{2}$ fold differences of expression levels with their standard errors and 95% CI (function avg_deviation(), package [*microViz*](https://github.com/PiotrTymoszuk/microViz)). Differentially regulated genes/proteins were identified by pFDR(ANOVA) < 0.05, $\eta^{2}$ $\geq$ 0.06, and pFDR(T test) < 0.05. The differentially regulated genes in bulk cancer transcriptome cohorts are listed in **Supplementary Table S23**. The differentially regulated proteins in bulk cancer proteome cohorts are listed in **Supplementary Table S25**. The differentially regulated genes in DepMap urothelial cancer cell lines are summarized in **Supplementary Table S52**; estimates of the strongest up- and downregulated genes in the clusters of DepMap cell lines are presented in Forest plots in **Supplementary Figure S29**.

For genes found to be differentially regulated in at least 10 bulk transcription cohorts and proteins found to be differentially regulated in at least two bulk proteome cohorts, meta-analysis of differential expression was performed. The corresponding estimates of differential expression with their standard errors obtained in single cohorts were used to compute meta-estimates of differential regulation in the clusters as compared with the cohort average with the DerSimonian-Lair algorithm (function f_meta(), package [*fastTest*](https://github.com/PiotrTymoszuk/fastTest))(77). Meta-estimates of the top strongest differentially regulated genes and proteins in bladder cancer clusters of bulk cancer transcriptomes and bulk cancer proteomes are displayed in Forest plots in **Supplementary Figures S18** and **S19**. The complete meta-analysis results are listed in **Supplementary Tables S24** and **S26**.

## Communities of differentially regulated genes and proteins in bladder cancer clusters

Genes found to be differentially regulated between bladder cancer clusters in at least 10 bulk cancer transcriptome cohorts, proteins found to be differentially regulated between bladder cancer clusters in each of the bulk cancer proteome cohort, and genes found to be differentially regulated between bladder cancer clusters of DepMap urothelial cancer cell lines were subjected to weighted gene/protein co-expression network analysis (WGCNA) (6,102).

For the differentially regulated genes in bulk cancer transcriptomic cohorts, the network analysis was performed in a pooled collective of ComBat-adjusted transcriptomes (function multi_process(), package [*htGLMNET*](https://github.com/PiotrTymoszuk/htGLMNET)). For the differentially regulated proteins, the networks were constructed separately for each of the Groeneveld 2024, Dressler 2024, and Stroggilos 2020 collectives. Genes constituted the network vertices, the edges were defined by pairwise correlations of the expression levels with Spearman’s $\rho$ $\geq$ 0.5 and were weighted with the $\rho$ values (function as_iGraph(), package [*graphExtra*](https://github.com/PiotrTymoszuk/graphExtra)).
Meta-estimates (bulk cancer transcriptomes) and estimates (proteome cohorts, DepMap cell lines) of differential gene expression were assigned to the networks’ vertices as attributes (function set_vertex_attributes(), [*graphExtra*](https://github.com/PiotrTymoszuk/graphExtra)). Isolated vertices, i.e. genes without correlation partners with $\rho$ $\geq$ 0.5 were removed (function prune_degree(cutoff = 0), package [*graphExtra*](https://github.com/PiotrTymoszuk/graphExtra)). Communities of co-regulated genes and proteins were identified with the Leiden algorithm with modularity objective function, resolution parameters within 0.7 - 1.2 range, and 100 iterations (function cluster_leiden()) (6,103). Communities with less than 20 genes or proteins were removed from the networks (functions comm_lump() and prune_vertices(), [*graphExtra*](https://github.com/PiotrTymoszuk/graphExtra)). Assignment of the differentially regulated genes and proteins to the communities is presented in **Supplementary Tables S27**, **S29**, and **S53**. The communities were named after their characteristic biological processes retrieved by biological process gene ontology (GO) enrichment analyses for the community members (function GOana(), package [*microViz*](https://github.com/PiotrTymoszuk/microViz)). In such enrichment analyses, GO terms with at least 10 gene/protein members of the community, FDR-adjusted p < 0.05, and enrichment odds ratio versus entire genome OR $\geq$ 1.44 were considered significant (**Supplementary Tables S28**, **S30**, and **S54**).

The networks were visualized as force-directed graphs with the Fruchterman-Reingold algorithm (method plot(), package [*graphExtra*](https://github.com/PiotrTymoszuk/graphExtra), internally using tools of *ggnetwork* package) (6,7). Distribution of meta-estimates and estimates of differential gene/protein expression in the gene/protein communities was displayed in density plots (in-house developed R function). These visualizations of the co-expression networks are presented in **Figures 4BC** and **7B**, and **Supplementary Figure S20**.

## Signatures of Reactome Pathways and cell proliferation in bladder cancer clusters

ssGSEA scores of gene and protein signatures of Reactome Pathways and cell proliferation were computed in bulk cancer transcriptome cohorts (TCGA BLCA, IMvigor, GSE13507, GSE32548, GSE48075, GSE48276, GSE83586, GSE86411, GSE87304, GSE120736, GSE124305, GSE128192, GSE128701, GSE128959, GSE198269, GSE203149, E-MTAB-4321, Groeneveld 2024, and BCAN), bulk cancer proteome cohorts (Groeneveld 2024, Dressler 2024, and Stroggilos 2020), and DepMap urothelial cancer cell lines as described in **Data sources**.

Regulation gene and protein signatures between the clusters and in the cluster as compared with the cohort’s mean was investigated by FDR-corrected one-way ANOVA with $\eta^{2}$ effect size metric and one-sample T test as described for differentially regulated genes and proteins (**Differential regulation of transcriptome and proteome in bladder cancer cancer cluster**). The T tests returned also estimates of differences of ssGSEA scores between the clusters and cohort’s mean along with their standard errors and 95% CI. Differentially regulated gene and protein signatures were identified by pFDR(ANOVA) < 0.05, $\eta^{2}$ $\geq$ 0.06, pFDR(T test) < 0.05 (**Supplementary Tables S31**, **S33**, and **S55**). Average ssGSEA scores of the signatures of cell proliferation in bulk transcriptome cohorts are presented in a heat map in **Figure 5D**.

Significantly differentially regulated Reactome Pathways gene signatures shared by at least 10 bulk cancer transcriptome cohorts, significant protein signatures shared by at least two bulk cancer proteome cohorts, and significant gene signatures in DepMap cell lines were subjected to a similarity network analysis. To this end, we computed pairwise Jaccard similarity coefficients J for the signatures, which measure the overlapping fractions of the signatures’ member genes or proteins. The networks were constructed with the differentially regulated signatures as vertices, and edges defined by J $\geq$ 0.3 and weighted by the J values (function as_iGraph(), package [*graphExtra*](https://github.com/PiotrTymoszuk/graphExtra)). Isolated vertices were remover from the networks (function prune_degree(cutoff = 0), [*graphExtra*](https://github.com/PiotrTymoszuk/graphExtra)). Regulation status of the signatures (cluster versus cohort mean) was set as the vertex attribute (function set_vertex_attributes()). Communities of co-regulated genes and proteins were identified with the Leiden algorithm with modularity objective function, resolution parameters set to 1, and 100 iterations (function cluster_leiden()). Communities with less than three signatures were removed from the networks (functions comm_lump() and prune_vertices(), [*graphExtra*](https://github.com/PiotrTymoszuk/graphExtra)). The communities were named after their most characteristic biological processes retrieved by inspection of the community member names. The signature - community assignment is presented in **Supplementary Tables S32**, **S34**, and **S56**. The similarity networks with the community assignment and the signatures’ regulation status were visualized as force-directed graphs with the Fruchterman-Reingold algorithm (function plot(), [*graphExtra*](https://github.com/PiotrTymoszuk/graphExtra)) in **Supplementary Figures S21**, **S22**, and **S30**.

## Predicted immune and stromal cell infiltration in bladder cancer clusters

Fractions of infiltrating immune and stromal cells in bulk cancer transcriptome samples were resolved by QuanTIseq and xCell immunedeconvolution (41,42) as described in **Data sources** (cohorts: TCGA BLCA, IMvigor, GSE13507, GSE32548, GSE48075, GSE48276, GSE83586, GSE86411, GSE87304, GSE120736, GSE124305, GSE128192, GSE128701, GSE128959, GSE198269, GSE203149, E-MTAB-4321, and Groeneveld 2024). The predicted infiltration levels were compared between bladder cancer clusters by FDR-corrected Kruskal-Wallis tests with $\eta^{2}$ effect size statistics (function f_kruskal_test(), package [*fastTest*](https://github.com/PiotrTymoszuk/fastTest)). Significant differences were considered for pFDR < 0.05 and $\eta^{2}$ $\geq$ 0.06 (**Supplementary Table S35**). Average Z-scores of xCell-predicted cancer sample fractions for the infiltrating cell populations found to be significant in at least 10 cohorts are presented in a heat map in **Figure 5A**. Representative data for the infiltrating cell populations found to be significant in at least 10 cohorts are shown in box plot panels in **Supplementary Figures S23** and **S24**.

## Differential regulation of matrisome genes and proteins

Matrisome is a system of comprehensive classification of extracellular matrix genes and proteins (104,105). Analyses and meta-analyses of differential gene expression were described above (**Differential regulation of transcriptome and proteome in bladder cancer cancer cluster**). From these data, the differential expression results for matrisome genes and proteins were extracted and assigned to matrisome categories (Collagens, ECM Glycoproteins, Proteoglycans, ECM Regulators, Secreted Factors, and ECM-affiliated Proteins) with matrianalyze() function from [*MatrisomeAnalyzeR*](https://github.com/Matrisome/MatrisomeAnalyzeR) package (104,105) (**Supplementary Tables S36**, **S37**, and **S57**). Distributions of meta-estimates of differential expression for matrisome genes found to be significantly regulated in at least 10 bulk cancer transcriptome cohorts and distributions of estimates of differential expression for significant matrisome proteins are presented in density plots in **Figure 5B** and **Supplementary Figure S25A**. Expression Z-scores for the significantly differentially regulated matrisome proteins are shown in heat maps in **Supplementary Figure S25B**. Estimates of differential expression and expression Z-scores for matrisome genes found to be differentially regulated between bladder cancer clusters of DepMap urothelial cancer cell lines are depicted in **Supplementary Figure S31**.

## Activity of transcriptional regulons and signaling pathways in bulk cancer transcriptomes, candidate transcriptional hubs

For bulk cancer transcriptome samples LM activity scores of collecTRI regulons and PROGENy signaling pathways (59,60) were predicted as described in **Data sources** (cohorts: TCGA BLCA, IMvigor, GSE13507, GSE32548, GSE48075, GSE48276, GSE83586, GSE86411, GSE87304, GSE120736, GSE124305, GSE128192, GSE128701, GSE128959, GSE198269, GSE203149, E-MTAB-4321, and Groeneveld 2024). Differences of the LM scores between bladder cancer clusters, and between the clusters and the cohort means were investigated by FDR-corrected one-way ANOVA with $\eta^{2}$ effect size metric and one-sample T test as described for differentially regulated genes and proteins (**Differential regulation of transcriptome and proteome in bladder cancer cancer cluster**). The T tests returned also estimates of differences of LM scores between the clusters and cohort’s mean along with their standard errors and 95% CI. Differentially regulated regulons and signaling pathways were identified by pFDR(ANOVA) < 0.05, $\eta^{2}$ $\geq$ 0.06, pFDR(T test) < 0.05 (**Supplementary Tables S38** and **S43**). Average LM scores of regulons and pathways found to be differentially modulated in at least 10 cohorts are shown in heat maps in **Figure 5C**.

For the regulons found to be differentially regulated in at least 10 bulk cancer transcriptome cohorts, meta-estimates of differences of LM scores between the clusters and cohort mean were computed with the DerSimonian-Lair algorithm (see: **Differential regulation of transcriptome and proteome in bladder cancer cancer cluster**) and are listed in **Supplementary Table S39**.

These common significant regulons were also subjected to a weighted correlation network analysis performed essentially as described for the differentially regulated genes (**Communities of differentially regulated genes and proteins in bladder cancer clusters**). This analysis was done with a pooled collective of LM scores in all bulk cancer transcriptome samples. The significantly regulated regulons constituted the network’s vertices, the edges were defined by pairwise correlations of LM scores with Spearman’s $\rho$ $\geq$ 0.3 and were weighted by the $\rho$ values. Isolated vertices were removed from the network. The meta-estimates of regulon activity in the clusters as compared with the cohort mean were set as the vertex attributes. Communities of correlating regulons were identified with the Leiden algorithm with modularity objective function, resolution parameter set to 1, and 100 iterations. The communities were named after their characteristic biological processes and families of transcription factors identified by a prompt to Google’s Gemini Flash 2.5 large language model with the community member lists. The community names were subsequently verified by a human analyst.

In a search for candidate transcriptional hubs, vertex importance statistics were computed for the regulon community members (functions split_vertices() and summary(), package [*graphExtra*](https://github.com/PiotrTymoszuk/graphExtra)). They were degree (number of direct neighbors/correlation partners as a measure of connectivity), hub score (eigenvector of the $\rho$ correlation matrix as a measure of overall correlation strength), and betweenness (number of the shortest paths between each two vertices of the community which pass through the vertex of interest as a measure of connectivity and non-redundancy) (6). The regulon - community assignment scheme and the vertex importance statistics are presented in **Supplementary Table S40**. The correlation network with the community assignment was visualized as a force-directed graph with the Fruchterman-Reingold algorithm; distribution of meta-estimates of regulon activity in bladder cancer clusters of the community members are shown in a density plot (**Supplementary Figure S26A**). The vertex importance statistics for the top essential regulons in the communities (top 10 hub score values) are shown in dot plots in **Supplementary Figure 26B**.

## Modeling of regulon and signaling activity in bulk cancer proteomes and transcriptomes of DepMap cell lines

Activity of collecTRI transcriptional regulons and PROGENy signaling pathways in bladder cancer clusters as compared with the data set mean was modeled in collectives of bulk cancer proteomes (Groeneveld 2024, Dressler 2024, and Stroggilos 2020) and DepMap urothelial cancer cell lines with *deceoupleR* package linear regression tools (collecTRI: univariable regression, function run_ulm(); PROGENy: multivariable regression, function run_mlm()) (59–61). The modeling response was linear modeling (LM) activity score of activation/inhibition of the regulon or signaling pathway. For the proteome data, the modeling functions were provided with T statistics of differential protein expression in the clusters as compared with the cohort mean obtained by one-sample T tests. For the DepMap cell lines, the modeling functions were fed with $log_{2}$ fold change estimates of differential gene expression in the clusters as compared with the data set means weighted by the corresponding raw p values (see: **Differential regulation of transcriptome and proteome in bladder cancer cancer clusters**). The p values of regulon and pathway activity were corrected for multiple testing with the FDR method. Significantly activated regulons/pathways were considered for LM score > 0 and pFDR < 0.05, significantly inhibited regulons/pathways were defined by LM score < 0 and pFDR < 0.05 (**Supplementary Tables S42**, **S43**, **S58**, and **S59**).

LM scores of regulons and signaling pathways predicted to be significantly differentially modulated in at least two proteome collectives are depicted in heat maps in **Supplementary Figure S27**. LM scores of regulons and signaling pathways predicted to be significantly differentially regulated in bladder cancer clusters of DepMap cell lines are shown in bubble plots in **Figure 7C**.

## Modeling of metabolism

Activity of metabolic reactions of the RECON2 knowledge model (106) was modeled in bladder cancer clusters of bulk cancer transcriptome cohorts (TCGA BLCA, IMvigor, GSE13507, GSE32548, GSE48075, GSE48276, GSE83586, GSE86411, GSE87304, GSE120736, GSE124305, GSE128192, GSE128701, GSE128959, GSE198269, GSE203149, E-MTAB-4321, Groeneveld 2024, and BCAN) and bulk cancer proteome cohorts (Groeneveld 2024, Dressler 2024, and Stroggilos 2020) by Monte Carlo simulations with differential gene/protein expression estimates. The modeling pipeline used bioinformatic tools of *BiGGR* and [*biggrExtra*](https://github.com/PiotrTymoszuk/biggrExtra) packages essentially as described in our previous publications (58,107,108).

In brief, for the modeling of activities of metabolic reactions in the cancer sample clusters as compared with the cohort’s averages, estimates of differential expression in the clusters versus data set mean with the corresponding standard errors for all genes and proteins were used (see: **Differential regulation of transcriptome and proteome in bladder cancer cancer cluster**). This analysis exploited the gene - reaction association rules in the RECON2 model of the BiGGR database and the simple gene - protein - reaction principle described by Gavai and colleagues (109,110). The gene - reaction association rules were evaluated for n = 3000 random draws from normal distributions of $log_{2}$ gene/protein expression differences (mean: estimate of $log_{2}$ differential expression, standard deviation: standard error of $log_{2}$ differential expression). This procedure yielded estimates of mean $log_{2}$ fold-changes in reaction activity with their 95% CI computed with the BCA method. P values were corrected for multiple testing with the FDR method. These modeling tasks were accomplished with build_geneSBML() function from [*biggrExtra*](https://github.com/PiotrTymoszuk/biggrExtra) package. Significantly differentially regulated metabolic reactions are listed in **Supplementary Tables S44** and **S46**.

Enrichment of RECON metabolic subsystems with significantly activated and inhibited reactions was investigated by comparing the subsystem’s reaction frequencies in the activated/inhibited reaction set with n = 10000 random draws from the entire reaction pool (function suba(), [*biggrExtra*](https://github.com/PiotrTymoszuk/biggrExtra)). The enrichment p values were corrected for multiple testing with the FDR method. Odds ratio (OR) served as the enrichment effect size metric. Significant enrichment was considered for pFDR < 0.05 and OR $\geq$ 1.44. OR and p values for metabolic subsystems found to be significantly enriched with activated or inhibited reactions in at least 10 transcriptome cohorts and at least two proteome cohorts are presented in bubble plots in **Figure 5E** and **Supplementary Figure S28**. The enrichment analysis results are summarized in **Supplementary Tables 45** and **S47**.

## Pharmacoinformatics of bladder cancer clusters

Resistance to anti-cancer drugs in form of Z-scores was predicted by RIDGE modeling of the gene expression data for bulk cancer transcriptome samples as described in **Data sources** (cohorts: TCGA BLCA, IMvigor, GSE13507, GSE32548, GSE48075, GSE48276, GSE83586, GSE86411, GSE87304, GSE120736, GSE124305, GSE128192, GSE128701, GSE128959, GSE198269, GSE203149, E-MTAB-4321, and Groeneveld 2024).

Differences of the resistance Z-scores between bladder cancer clusters, and between the clusters and the cohort means were investigated by FDR-corrected one-way ANOVA with $\eta^{2}$ effect size metric and one-sample T test as described for differentially regulated genes and proteins (**Differential regulation of transcriptome and proteome in bladder cancer cancer cluster**). The T tests returned also estimates of differences of resistance Z-scores between the clusters and cohort’s mean along with their standard errors and 95% CI. Drugs with predicted significant differences in resistance were identified by pFDR(ANOVA) < 0.05, $\eta^{2}$ $\geq$ 0.06, pFDR(T test) < 0.05 (**Supplementary Table S48**). For significant effects shared by at least 10 bulk cancer transcriptome cohorts, meta estimates of differences in resistance Z-scores between the clusters and the cohort mean were computed with the DerSimonian-Lair algorithm (function f_meta(), package [*fastTest*](https://github.com/PiotrTymoszuk/fastTest), **Supplementary Table S49**). Distributions of these meta-estimates of resistance for selected drug categories (FGFR inhibitors, EGFR/ERBB inhibitors, inhibitors of the RAS/RAF/MAPK and PI3K/TOR pathways, inhibitors of other growth factor signaling pathways, drugs targeting DNA, cytoskeleton, cell cycle and DNA repair machinery, PARP inhibitors, apoptosis inducers, drug targeting epigenetic machinery, and ferroptosis inducers) are presented in density plots in **Figure 6A**. Mean resistance Z-scores for EGFR/ERBB and RAS/RAF/MAPK inhibitors, DNA targeting drugs, and PARP inhibitors found to be significant in at least 10 cohorts are shown in heat maps in **Figure 6B**.

To assess feasibility of ADC therapy in bladder cancer clusters, expression levels of genes and proteins coding for targets of approved and experimental ADC drugs (67,68) were compared between bladder cancer clusters and cohort means as outlined above for differential gene expression analyses. The results are shown in **Supplementary Figure S32**.

## Overall survival of patients with systemic cytotoxic chemotherapy treatment in bladder cancer clusters

With the drug treatment information extracted from the publication by Moiso (17), we identified TCGA BLCA cohort patients in each of the bladder cancer cluster who received at least one cycle of systemic cytotoxic therapy (gemcitabine, platinum, topoisomerase inhibitors, alkylating agents, or metothrexate). Overall survival of these patients was compared with overall survival of patients with other therapy or no therapy; such comparisons were done separately for cluster #1, #2, and #3 individuals. Statistical significance of the differences in overall survival were assessed by Kaplan-Meier analysis and Peto-Peto tests (functions surv_fit(), quantile.survfit(), and surv_pvalue(), packages *survminer* and *survival*) (73,75). The Peto-Peto test p values were corrected for multiple testing with the FDR method. Survival curves were visualized with function ggsurvplot() from *survminer* package (75). The analysis results are presented in **Figure 6C** and **Supplementary Table S50**.

## Identification of essential genes for bladder cancer clusters by re-analysis of CRISPR-Cas9 knockout screening data

Results of the CRISPR-Cas9 KO screening experiment with DepMap cell lines were imported and processed as described in **Data sources**. Of note, for a better interpretability, the Chronos gene effect scores were multiplied by -1, so that positive values are correspond to cell growth inhibition upon the gene KO. Gene effects with Chronos scores > 0.5 were considered biologically relevant (65,66). Genes without such biologically relevant effects in any of the DepMap urothelial cancer cell lines were excluded from further analyses.
Analogically, genes with biologically relevant effects in > 99% of cell lines in the entire DepMap collection of approximately 1000 cell lines, i.e bona-fide house-keeping genes with non-cancer specific functions, were were also excluded from further analyses. Distributions of Chronos gene effect scores in bladder cancer clusters for the remaining 1574 genes were modeled by bootstrapping with n = 1000 iterations (a wrapper around functions of *boot* package) (111). This testing procedure allowed us to determine expected gene effects in the clusters with their 95% CI computed with the BCA method, and calculate p values for significant gene effects (null hypothesis: Chronos score $\leq$ 0.5). In addition, we assessed differences of gene effects between the clusters with bootstrapped F statistic (i.e. ratio of the between cluster variance to the within cluster variance of Chronos gene effect scores). Because p values in these two hypothesis tests depended strongly on the iteration number, significant differences were considered for raw p < 0.05 without any multiple testing correction. Hence, the essential cluster-specific genes were defined by significant biological effects in at least one of the clusters (bootstrapped Chronos score estimate > 0.5 with p < 0.05) and significant differences of biological effects between clusters (bootstrapped F > 1 with raw p < 0.05). Such essential cluster-specific genes (n = 77) are listed in **Supplementary Table S60**. Their gene effect Z-scores in bladder cancer clusters of DepMap urothelial cancer cell lines are depicted in a heat map in **Figure 7D**.

## Comparison of differentially regulated transcriptomes in bladder cancer clusters, UROMOL classes of NMIBC, and consensus classes of MIBC

Comparisons of differentially regulated transcriptomes of bladder cancer clusters, UROMOL classes (112), and consensus classes (39) were conducted in cohorts with at least 80 bulk NMIBC or MIBC specimens. Differences in $log_{2}$-transformed gene expression levels in bladder cancer clusters as compared with the cohort average and in UROMOL classes of NMIBC as compared with the cohort average were investigated in the GSE13507 (bladder cancer clusters, #1: n = 13, #2: n = 10, #3: n = 73; UROMOL classes, 1: n = 32, 2a: n = 33, 2b: n = 12, 3: n = 19), GSE32548 (bladder cancer clusters, #1: n = 15, #2: n = 9, #3: n = 63; UROMOL classes, 1: n = 21, 2a: n = 35, 2b: n = 19, 3: n = 12), GSE86411 (bladder cancer clusters, #1: n = 41, #2: n = 28, #3: n = 62; UROMOL classes, 1: n = 9, 2a: n = 56, 2b: n = 61, 3: n = 5), GSE128959 (bladder cancer clusters, #1: n = 51, #2: n = 14, #3: n = 87; UROMOL classes, 1: n = 19, 2a: n = 68, 2b: n = 48, 3: n = 17), and E-MTAB-4321 cohort (bladder cancer clusters, #1: n = 137, #2: n = 67, #3: n = 234; UROMOL classes, 1: n = 83, 2a: n = 155, 2b: n = 119, 3: n = 81 samples). Differences in $log_{2}$-transformed gene expression levels in bladder cancer clusters as compared with the cohort average and in consensus classes of MIBC as compared with the cohort average were investigated in the TCGA (bladder cancer clusters, #1: n = 110, #2: n = 116, #3: n = 145; consensus classes, stroma-rich: n = 49, luminal-unstable: n = 28, luminal non-specified: n = 34, basal/squamous: n = 113, luminal-papillary: n = 140, neuroendocrine-like: n = 7), GSE83586 (bladder cancer clusters, #1: n = 67, #2: n = 52, #3: n = 82; consensus classes, stroma-rich: n = 31, luminal-unstable: n = 25, luminal non-specified: n = 15, basal/squamous: n = 52, luminal-papillary: n = 74, neuroendocrine-like: n = 4), GSE83586 (bladder cancer clusters, #1: n = 65, #2: n = 77, #3: n = 85; consensus classes, stroma-rich: n = 29, luminal-unstable: n = 27, luminal non-specified: n = 20, basal/squamous: n = 77, luminal-papillary: n = 64, neuroendocrine-like: n = 10), GSE87304 (bladder cancer clusters, #1: n = 75, #2: n = 91, #3: n = 123; consensus classes, stroma-rich: n = 28, luminal-unstable: n = 45, luminal non-specified: n = 16, basal/squamous: n = 95, luminal-papillary: n = 100, neuroendocrine-like: n = 5), GSE128192 (bladder cancer clusters, #1: n = 33, #2: n = 36, #3: n = 40; consensus classes, stroma-rich: n = 17, luminal-unstable: n = 9, luminal non-specified: n = 9, basal/squamous: n = 34, luminal-papillary: n = 39, neuroendocrine-like: n = 1), and GSE203149 cohort (bladder cancer clusters, #1: n = 51, #2: n = 33, #3: n = 69; consensus classes, stroma-rich: n = 16, luminal-unstable: n = 15, luminal non-specified: n = 16, basal/squamous: n = 28, luminal-papillary: n = 76, neuroendocrine-like: n = 2). Statistical significance was assessed by one-way ANOVA with $\eta^{2}$ effect size metric and post-hoc one-sample T test, in an analogical way as described above for the differential gene and protein expression analyses. P values were adjusted for multiple testing with the false discovery rate (FDR) method. Significant differences were considered for pFDR(ANOVA) < 0.05, $\eta^{2}\geq$ 0.06, and pFDR(T test) < 0.05. Subsequently, common differentially regulated genes were defined as genes significantly regulated in at least three cohorts (**Supplementary Table S61** and **62**). Similarities of pairs of bladder cancer clusters, UROMOL classes, and consensus classes in respect to differentially regulated genes identified in at least three cohorts were quantified by Jaccard’s coefficient J (function set_similarity(), package [*microViz*](https://github.com/PiotrTymoszuk/microViz), **Supplementary Table S63**). Numbers of concordantly and discordantly up- and downregulated genes in pairs of bladder cancer clusters, UROMOL classes, and consensus classes were visualized in Euler plots with and in-house developed wrapper around function euler() from *eulerr* package (113). Biological process GO term enrichment analyses for differentially up- and downregulated gene shared by pairs of bladder cancer clusters, UROMOL classes, and consensus classes of MIBC were performed with *goana* algorithm (88) and GOana() function from [*microViz*](https://github.com/PiotrTymoszuk/microViz) as described above for gene and protein communities. Significantly enriched terms were identified by enrichment odds ratio OR $\geq$ 1.44, pFDR < 0.05, and at least three shared regulated genes per term (**Supplementary Table S64**). Common markers of bladder cancer clusters, UROMOL classes, consensus classes were identified among genes with differential expression between the subsets (pFDR(ANOVA) < 0.05, $\eta^{2}\geq$ 0.06) by receiver-operating characteristics (ROC) with area under the ROC curve AUC >= 0.714 in at least three cohorts (**Supplementary Table S65**).

In these analyses for bladder cancer clusters and consensus classes of MIBC, no substantial similarities of between the neuroendocrine-like class and any of the bladder cancer clusters was revealed (neuroendocrine-like and #1: J = 0.02, #2: J = 0.025, #3: J = 0.0024), which was in part attributed to very small sizes of the neuroendocrine-like subset. Hence, to assess neuroendocrine features of bladder cancer clusters, expression of genes and proteins coding for published markers of neuroendocrine differentiation (39,69–72) was compared between bladder cancer clusters and the cohort mean as described above for the differential gene and protein expression analyses. Results of these comparisons are presented in **Supplementary Figure S35**.

## Bladder cancer clusters and PAM50 molecular subsets of the TCGA BLCA cohort

Samples in the pan-carcinoma TCGA collective were assigned to luminal A, luminal B, and basal molecular clusters defined by expression of PAM50 genes (114) by Zhao et colleagues (18). The PAM50 cluster assignment was extracted for n = 404 for TCGA BLCA cancer specimens from the genuine report and percentages of samples in PAM50 molecular subsets were compared between bladder cancer clusters (**Supplementary Figure S36A**, **Supplementary Table S66**).

Differential expression of PAM50 transcripts in bladder cancer clusters and PAM50 molecular subsets of the TCGA BLCA cohort as compared with the cohort average was investigated as described above for differential gene and protein expression by FDR-adjusted one-way ANOVA with $\eta^{2}$ effect size statistic and one-sample post-hoc T test. Differential regulation was considered for pFDR(ANOVA) < 0.05, $\eta^{2}\geq$ 0.06, and pFDR(T test) < 0.05. The results of the differential PAM50 gene expression analysis are presented in **Supplementary Figure S36B** and **Supplementary Table S67**.

# Data and code availability

The analyzed data sets are freely available. The analysis pipelines are available as GitHub repositories [BLCA-cluster-paper](https://github.com/PiotrTymoszuk/BLCA-cluster-paper) and [BLCA_subset_schemes](https://github.com/PiotrTymoszuk/BLCA_subset_schemes).

An interactive HTML presentation with Supplementary Material is available from a [dedicated GitHub repository](https://github.com/PiotrTymoszuk/BLCA_cluster_supplements).

# Supplementary Tables

Table 1: R packages used in the current project. Package names, sources, usage, links to the sources, and versions are listed. The table is available as a supplementary Excel file.

|  |
| --- |
|  |

Table 2: Sources of data used and analyzed in the current report. Data types and availability, biological material short descriptions, usage in analyses, numbers of entries, links to sources, and publications are listed. The table is available as a supplementary Excel file.

|  |
| --- |
|  |

Table 3: Demographic and clinical characteristic of the analyzed transcriptomic and proteomic cohorts of urothelial cancer patients. Qualitative variables are presented as percentages and counts of their categories within the complete observation sets. Quantitative variables are shown as medians with interquartile ranges and ranges. The table is available as a supplementary Excel file.

|  |
| --- |
|  |

Table 4: Pathological characteristic of the analyzed collectives of transcriptomic and proteomic bulk cancer samples. Qualitative variables are presented as percentages and counts of their categories within the complete observation sets. Quantitative variables are shown as medians with interquartile ranges and ranges. The table is available as a supplementary Excel file.

|  |
| --- |
|  |

Table 5: Choice of the self-organizing map (SOM) algorithm used for definition of metagenes. Several SOM structures with 16 x 16 hexagonal units were constructed in a training portion of the TCGA BLCA bulk transcriptome cohort. The SOM structures employed different distance metrics (Euclidean, Manhattan, squared Euclidean, cosine) and plain or toroidal topology, and operated with ComBat-adjusted log2 expression levels of >3000 genes passing a variability criterion. Selection of the SOM toroidal/squared Euclidean distance algorithm for definition of metagenes, i.e. sets of tightly co-regulated genes, was motivated by the lowest topological error, good neighborhood preservation (measure of cluster separation), and good clustering variance (measure of explanatory performance) in the training and test subsets of the TCGA BLCA cohort.

| **Topology^a^** | **Distance metric** | **TCGA cohort subset** | **Topology error** | **Neighborhood preservation** | **Clustering variance** |
| --- | --- | --- | --- | --- | --- |
| plain | cosine | training | 0.375 | 0.639 | 0.831 |
|  |  | test |  | 0.513 | 0.767 |
|  | Euclidean | training | 0.331 | 0.573 | 0.841 |
|  |  | test |  | 0.557 | 0.748 |
|  | Manhattan | training | 0.357 | 0.585 | 0.834 |
|  |  | test |  | 0.583 | 0.752 |
|  | Euclidean² | training | 0.316 | 0.573 | 0.839 |
|  |  | test |  | 0.545 | 0.734 |
| toroidal | cosine | training | 0.317 | 0.638 | 0.843 |
|  |  | test |  | 0.534 | 0.776 |
|  | Euclidean | training | 0.281 | 0.576 | 0.852 |
|  |  | test |  | 0.548 | 0.762 |
|  | Manhattan | training | 0.318 | 0.583 | 0.845 |
|  |  | test |  | 0.567 | 0.765 |
|  | Euclidean² | training | 0.281 | 0.576 | 0.851 |
|  |  | test |  | 0.547 | 0.750 |
| ^a^Euclidean²: squared Euclidean distance. | | | | | |

Table 6: Assignment of genes to metagenes. >3000 genes passing a variability criterion were assigned to metagenes, i.e. sets of tightly co-regulated genes by a 16 x 16 node toroidal self-organizing map (SOM) with squared Euclidean distance between the data points and nodes. The SOM was fed with ComBat-adjusted log2-transformed gene expression levels for bulk cancer samples of the entire TCGA BLCA data set. Genes assigned to the same SOM node formed a metagene. Biological function of the metagenes was resolved by a biological process gene ontology (GO) term enrichment analysis. Gene symbols, Entrez IDs, and significantly enriched GO terms are summarized for all 256 metagenes in a supplementary Excel file.

|  |
| --- |
|  |

Table 7: Choice of the metagene clustering algorithm. Single sample gene set enrichment scores were computed for metagenes in the bulk cancer mRNA TCGA BLCA cohort. The TCGA BLCA bulk cancer samples were subjected to hard-threshold regularized KMEANS clustering in respect to their metagene scores. The regularization parameter lambda was found by maximizing cross-validated clustering variance. Clustering solutions for increasing cluster number k were generated, and their cluster separation, potential misclassification, neighborhood preservation, and clustering variances were evaluated in the entire data set and out-of-fold predictions in 5-fold cross-validation (CV). The k = 3 solution offered a good trade-off between the clustering variance and cluster separation and was employed for definition of bladder cancer clusters.

| **Cluster number, k** | **Regularization, lambda** | **Evaluation data set** | **Mean silhouette width^a^** | **Misclassification^b^** | **Neighborhood preservation^c^** | **Clustering variance^d^** |
| --- | --- | --- | --- | --- | --- | --- |
| 2 | 0.125 | training | 0.323 | 0.0197 | 0.926 | 0.464 |
|  |  | 5-fold CV | 0.318 | 0.0392 | 0.893 | 0.453 |
| 3 | 0.225 | training | 0.3 | 0.0246 | 0.89 | 0.582 |
|  |  | 5-fold CV | 0.287 | 0.0834 | 0.832 | 0.574 |
| 4 | 0.25 | training | 0.234 | 0.059 | 0.834 | 0.646 |
|  |  | 5-fold CV | 0.211 | 0.142 | 0.74 | 0.629 |
| 5 | 0.325 | training | 0.181 | 0.133 | 0.8 | 0.681 |
|  |  | 5-fold CV | 0.156 | 0.177 | 0.675 | 0.656 |
| 6 | 0.475 | training | 0.155 | 0.147 | 0.741 | 0.698 |
|  |  | 5-fold CV | 0.124 | 0.224 | 0.64 | 0.677 |
| ^a^Measure of cluster separation | | | | | | |
| ^b^Fraction of samples with negative silhouette widths | | | | | | |
| ^c^Mean fraction of the five nearest neighbors of a sample placed in the same cluster. | | | | | | |
| ^d^Ratio of the total between-clusters sum of squares to the total sum of squares. | | | | | | |

Table 8: Assignment of bulk cancer transcriptome samples to bladder cancer clusters. Bladder cancer clusters were developed by classifying specimens of the TCGA BLCA training cohort in respect to single sample gene set enrichment scores (ssGSEA) metagene scores by hard-threshold regularized KMEANS clustering. ssGSEA scores of the metagenes were computed for independent validation cohorts of bulk cancer transcriptomes (cohorts: IMvigor, GSE13507, GSE32548, GSE48075, GSE48276, GSE83586, GSE86411, GSE87304, GSE120736, GSE124305, GSE128192, GSE128701, GSE128959, GSE198269, GSE203149, E-MTAB-4321, and Groeneveld 2024) Their samples were assigned to bladder cancer clusters by an inverse distance-weighted k-nearest neighbor classifier trained in the TCGA BLCA cohort. Quality of clustering and similarity with bladder cancer clusters of training cohort were assessed by Spearman's rho coefficients of correlation with the cluster's centroids in the TCGA BLCA cohort. The assignment table is available as a supplementary Excel file.

|  |
| --- |
|  |

Table 9: Technical validation of bladder cancer clusters in cohorts of transcriptomic bulk cancer samples. Bladder cancer clusters were developed by classifying specimens of the TCGA BLCA training cohort in respect to single sample gene set enrichment scores (ssGSEA) metagene scores by hard-threshold regularized KMEANS clustering. ssGSEA scores of the metagenes were computed for independent validation cohorts of bulk cancer transcriptomes. Their samples were assigned to bladder cancer clusters by an inverse distance-weighted k-nearest neighbor classifier trained in the TCGA BLCA cohort. Cluster separation, potential misclassification rate, nearest neighborhood preservation, and clustering variance was evaluated in the TCBA BLCA training cohort and the validation bulk cancer transcriptome collectives.

| **Cohort** | **Mean silhouette width^a^** | **Misclassification^b^** | **Neighborhood preservation^c^** | **Clustering variance^d^** |
| --- | --- | --- | --- | --- |
| TCGA BLCA | 0.300 | 0.0246 | 0.890 | 0.582 |
| IMvigor | 0.285 | 0.0814 | 0.855 | 0.537 |
| GSE13507 | 0.205 | 0.1120 | 0.783 | 0.373 |
| GSE32548 | 0.224 | 0.0534 | 0.840 | 0.449 |
| GSE48075 | 0.319 | 0.0563 | 0.863 | 0.614 |
| GSE48276 | 0.301 | 0.0172 | 0.909 | 0.574 |
| GSE83586 | 0.215 | 0.0554 | 0.858 | 0.471 |
| GSE86411 | 0.263 | 0.0909 | 0.876 | 0.497 |
| GSE87304 | 0.205 | 0.0721 | 0.849 | 0.478 |
| GSE120736 | 0.179 | 0.1790 | 0.796 | 0.348 |
| GSE124305 | 0.235 | 0.1050 | 0.792 | 0.450 |
| GSE128192 | 0.221 | 0.0714 | 0.807 | 0.448 |
| GSE128701 | 0.173 | 0.1400 | 0.809 | 0.345 |
| GSE128959 | 0.191 | 0.1510 | 0.808 | 0.419 |
| GSE198269 | 0.199 | 0.0888 | 0.825 | 0.414 |
| GSE203149 | 0.168 | 0.1050 | 0.809 | 0.407 |
| E-MTAB-4321 | 0.174 | 0.1130 | 0.812 | 0.352 |
| Groeneveld 2024 | 0.306 | 0.0404 | 0.899 | 0.594 |
| ^a^Measure of cluster separation | | | | |
| ^b^Fraction of samples with negative silhouette widths | | | | |
| ^c^Mean fraction of the five nearest neighbors of a sample placed in the same cluster. | | | | |
| ^d^Ratio of the total between-clusters sum of squares to the total sum of squares. | | | | |

Table 10: Single sample gene enrichment analysis (ssGSEA) scores of the cluster-defining metagenes in bladder cancer clusters. Statistical significance of differences in metagene scores between bladder cancer clusters was determined by Kruskal-Wallis test with eta-square effect size metric. P values were corrected for multiple comparisons with the false discovery rate (FDR) method. Significant differences were considered for pFDR < 0.05 and eta-square >= 0.06. Median ssGSEA scores with interquartile ranges and ranges for metagenes found to be significant in at least 10 cohorts are summarized in a table. The table is available as a supplementary Excel file.

|  |
| --- |
|  |

Table 11: Coefficients of the Elastic Net classifiers used for assignment of bulk cancer transcriptome samples in the BCAN cohort, bulk cancer proteome samples, and DepMap urothelial cancer cell lines to bladder cancer clusters. The multinomial Elastic Net models of bladder cancer cluster assignment were trained with bulk cancer transcriptomic data of the TCGA BLCA cohort. As explanatory variables, ComBat-processed expression Z-scores (BCAN cohort classifier), ComBat-processed log2-transformed mRNA levels of genes correlating with protein levels (proteome cohort classifier), or Combat-processed log2-transformed mRNA levels of genes expressed with >= 5 TPM (cell line classifier) were used. The optimal values of the regularization parameters lambda was chosen by minimizing the classification error in repeated cross-validation. Non-zero beta coefficients are listed. The table is available as a supplementary Excel file.

|  |
| --- |
|  |

Table 12: Selection of explanatory variables for an Elastic Net classifier used for assignment of bulk cancer proteomes to bladder cancer clusters. For subsets of samples in the Groeneveld 2024 and Stroggilos 2020 cohorts, both mRNA sequencing and proteome data were available. Correlation of log2-transformed gene expression counts and log2-transformed protein mass spectrometry counts was investigated by Pearson's permutation test with correlation coefficient r as an effect size measure. P values were corrected for multiple testing with the false discovery rate (FDR) method. Genes detected in all transcriptomic and proteomic bulk cancer cohorts whose transcript and protein product levels correlated with raw (no FDR) p < 0.05 and r >= 0.5 in at least one of the cohorts were selected as explanatory variables for the Elastic Net classifier used for assignment of bulk cancer proteomes to bladder cancer clusters. Those genes are listed in a table available as a supplementary Excel file.

|  |
| --- |
|  |

Table 13: Coefficients of the Elastic Net classifiers used for assignment of bulk cancer transcriptome samples in the BCAN cohort, bulk cancer proteome samples, and DepMap urothelial cancer cell lines to bladder cancer clusters. The multinomial Elastic Net models of bladder cancer cluster assignment were trained with bulk cancer transcriptomic data of the TCGA BLCA cohort. As explanatory variables, ComBat-processed expression Z-scores (BCAN cohort classifier), ComBat-processed log2-transformed mRNA levels of genes correlating with protein levels (proteome cohort classifier), or Combat-processed log2-transformed mRNA levels of genes expressed with >= 5 TPM (cell line classifier) were used. The optimal values of the regularization parameters lambda was chosen by minimizing the classification error in repeated cross-validation. Performance of the Elastic Net classifiers at prediction of the cluster assignment was evaluated in cohorts of bulk cancer transcriptomes. Bladder cancer cluster classification based on metagene scores served as the ground truth.

| **Model application** | **Cohort** | **Accuracy^a^** | **Kappa^b^** | **log Loss^c^** | **Brier score^d^** | **AUC^e^** | **Mean sensitivity^f^** | **Mean specificity^f^** |
| --- | --- | --- | --- | --- | --- | --- | --- | --- |
| BCAN | TCGA BLCA | 1.00 | 1.00 | 0.078 | 0.020 | 1.00 | 1.00 | 1.00 |
|  | IMvigor | 0.90 | 0.84 | 0.260 | 0.140 | 0.98 | 0.89 | 0.95 |
|  | GSE13507 | 0.84 | 0.70 | 0.420 | 0.240 | 0.96 | 0.78 | 0.89 |
|  | GSE32548 | 0.95 | 0.91 | 0.280 | 0.140 | 0.99 | 0.92 | 0.96 |
|  | GSE48075 | 0.90 | 0.84 | 0.230 | 0.130 | 0.99 | 0.86 | 0.94 |
|  | GSE48276 | 0.95 | 0.92 | 0.190 | 0.093 | 1.00 | 0.95 | 0.97 |
|  | GSE83586 | 0.90 | 0.84 | 0.310 | 0.170 | 0.98 | 0.88 | 0.94 |
|  | GSE86411 | 0.86 | 0.78 | 0.380 | 0.210 | 0.97 | 0.88 | 0.93 |
|  | GSE87304 | 0.89 | 0.82 | 0.320 | 0.180 | 0.98 | 0.86 | 0.94 |
|  | GSE120736 | 0.81 | 0.67 | 0.400 | 0.220 | 0.96 | 0.75 | 0.88 |
|  | GSE124305 | 0.86 | 0.79 | 0.340 | 0.200 | 0.98 | 0.86 | 0.93 |
|  | GSE128192 | 0.89 | 0.84 | 0.320 | 0.170 | 0.98 | 0.89 | 0.95 |
|  | GSE128701 | 0.82 | 0.73 | 0.420 | 0.240 | 0.97 | 0.82 | 0.91 |
|  | GSE128959 | 0.85 | 0.74 | 0.440 | 0.240 | 0.95 | 0.80 | 0.91 |
|  | GSE198269 | 0.86 | 0.78 | 0.370 | 0.200 | 0.97 | 0.85 | 0.92 |
|  | GSE203149 | 0.81 | 0.69 | 0.430 | 0.240 | 0.96 | 0.79 | 0.90 |
|  | E-MTAB-4321 | 0.81 | 0.68 | 0.450 | 0.260 | 0.95 | 0.77 | 0.89 |
|  | Groeneveld 2024 | 0.93 | 0.89 | 0.220 | 0.110 | 0.99 | 0.92 | 0.96 |
| proteome cohorts | TCGA BLCA | 1.00 | 0.99 | 0.150 | 0.058 | 1.00 | 0.99 | 1.00 |
|  | IMvigor | 0.87 | 0.81 | 0.340 | 0.190 | 0.97 | 0.86 | 0.94 |
|  | GSE13507 | 0.86 | 0.75 | 0.410 | 0.220 | 0.97 | 0.83 | 0.91 |
|  | GSE32548 | 0.90 | 0.83 | 0.290 | 0.150 | 0.99 | 0.87 | 0.94 |
|  | GSE48075 | 0.92 | 0.86 | 0.230 | 0.120 | 0.99 | 0.89 | 0.95 |
|  | GSE48276 | 0.93 | 0.89 | 0.290 | 0.150 | 0.99 | 0.92 | 0.96 |
|  | GSE83586 | 0.90 | 0.84 | 0.290 | 0.160 | 0.98 | 0.89 | 0.95 |
|  | GSE86411 | 0.85 | 0.76 | 0.420 | 0.240 | 0.96 | 0.86 | 0.92 |
|  | GSE87304 | 0.89 | 0.83 | 0.350 | 0.190 | 0.97 | 0.87 | 0.94 |
|  | GSE120736 | 0.81 | 0.68 | 0.470 | 0.270 | 0.93 | 0.77 | 0.90 |
|  | GSE124305 | 0.86 | 0.80 | 0.340 | 0.190 | 0.97 | 0.86 | 0.93 |
|  | GSE128192 | 0.85 | 0.77 | 0.390 | 0.220 | 0.97 | 0.85 | 0.92 |
|  | GSE128701 | 0.78 | 0.66 | 0.480 | 0.290 | 0.94 | 0.78 | 0.88 |
|  | GSE128959 | 0.77 | 0.62 | 0.540 | 0.320 | 0.91 | 0.74 | 0.88 |
|  | GSE198269 | 0.89 | 0.82 | 0.350 | 0.190 | 0.97 | 0.89 | 0.94 |
|  | GSE203149 | 0.83 | 0.73 | 0.410 | 0.220 | 0.96 | 0.82 | 0.91 |
|  | E-MTAB-4321 | 0.82 | 0.71 | 0.490 | 0.270 | 0.94 | 0.79 | 0.90 |
|  | Groeneveld 2024 | 0.93 | 0.89 | 0.230 | 0.120 | 0.99 | 0.92 | 0.96 |
| DepMap cell lines | TCGA BLCA | 0.98 | 0.97 | 0.240 | 0.100 | 1.00 | 0.98 | 0.99 |
|  | IMvigor | 0.86 | 0.78 | 0.440 | 0.240 | 0.96 | 0.85 | 0.93 |
|  | GSE13507 | 0.85 | 0.72 | 0.500 | 0.270 | 0.95 | 0.79 | 0.90 |
|  | GSE32548 | 0.89 | 0.80 | 0.420 | 0.220 | 0.98 | 0.84 | 0.93 |
|  | GSE48075 | 0.89 | 0.81 | 0.330 | 0.160 | 0.99 | 0.86 | 0.93 |
|  | GSE48276 | 0.91 | 0.87 | 0.370 | 0.190 | 0.99 | 0.90 | 0.96 |
|  | GSE83586 | 0.89 | 0.83 | 0.390 | 0.210 | 0.98 | 0.88 | 0.94 |
|  | GSE86411 | 0.85 | 0.76 | 0.510 | 0.280 | 0.96 | 0.86 | 0.92 |
|  | GSE87304 | 0.86 | 0.78 | 0.450 | 0.250 | 0.96 | 0.84 | 0.93 |
|  | GSE120736 | 0.83 | 0.70 | 0.520 | 0.290 | 0.94 | 0.78 | 0.90 |
|  | GSE124305 | 0.87 | 0.81 | 0.410 | 0.220 | 0.98 | 0.87 | 0.94 |
|  | GSE128192 | 0.88 | 0.83 | 0.490 | 0.260 | 0.97 | 0.88 | 0.94 |
|  | GSE128701 | 0.77 | 0.65 | 0.560 | 0.320 | 0.94 | 0.76 | 0.88 |
|  | GSE128959 | 0.81 | 0.67 | 0.510 | 0.280 | 0.94 | 0.75 | 0.89 |
|  | GSE198269 | 0.86 | 0.78 | 0.450 | 0.240 | 0.96 | 0.84 | 0.92 |
|  | GSE203149 | 0.81 | 0.70 | 0.560 | 0.300 | 0.93 | 0.78 | 0.90 |
|  | E-MTAB-4321 | 0.80 | 0.67 | 0.570 | 0.320 | 0.93 | 0.77 | 0.89 |
|  | Groeneveld 2024 | 0.91 | 0.86 | 0.300 | 0.150 | 0.99 | 0.89 | 0.95 |
| ^a^Fraction of samples with correct cluster assignment. | | | | | | | | |
| ^b^Cohen's κ: non-random concordance between the predicted cluster assignment and ground truth. High values are expected for a highly accurate model. | | | | | | | | |
| ^c^Cross-entrophy, a metric of model confidence. Low values are expected for a highly confident model. | | | | | | | | |
| ^d^Mean square difference between the cluster assignment probability returned by the model and the 0/1 coded cluster assignment. A measure of model confidence and clibration. Low values are expected for a highly confident and well calibrated model. | | | | | | | | |
| ^e^Area under the receiver-operating characteristic. | | | | | | | | |
| ^f^Sensitivity and specificity of cluster indeitfication averaged over all bladder cancer clusters. | | | | | | | | |

Table 14: Assignment of bulk cancer transcriptomes in the BCAN cohort, and bulk cancer proteomes in the Groeneveld 2024, Dressler 2024, and Stroggilos 2020 cohorts to bladder cancer clusters. The assignment was accomplished by Elastic Net classifiers trained in the TCGA BLCA collective Probabilities of assignment to particular bladder cancer clusters, and the final assignment derived from nodal voting are listed. Quality of clustering and similarity with bladder cancer clusters of training cohort were assessed by Spearman's rho coefficients of correlation with the cluster's centroids in the TCGA BLCA cohort. The table is available as a supplementary Excel file.

|  |
| --- |
|  |

Table 15: NMIBC UROMOL classes and MIBC consensus classes in bladder cancer clusters. Bulk cancer transcriptome samples were assigned to NMIBC UROMOL classes and MIBC consensus classes by nearest centroid classifiers implemented by R packages classifyNMIBC and consensusMIBC. The table is available as a supplementary Excel file.

|  |
| --- |
|  |

Table 16: Demographic, clinical, and pathological characteristic of bladder cancer clusters in pooled collectives of non-muscle invasive (NMIBC) and muscle invasive (MIBC) bladder cancers. Qualitative variables are presented as percentages and counts of complete observations in a cluster. Quantitative variables are shown as medians with interquartile ranges and ranges. Statistical significance of differences between bladder cancer clusters was determined by chi-square test (qualitative variables) or Kruskal-Wallis test. Cramer's V and eta-square served as effect size statistics. P values were corrected for multiple testing with the false discovery rate method.

| **Cancer specimen subset** | **Variable^a^** | **#1^b^** | **#2^c^** | **#3^d^** | **Significance^e^** | **Effect size^e^** |
| --- | --- | --- | --- | --- | --- | --- |
| NMIBC | age, years | 68 [IQR: 61 - 76] range: 24 - 96 complete: n = 179 | 71 [IQR: 65 - 80] range: 37 - 89 complete: n = 90 | 67 [IQR: 59 - 75] range: 24 - 89 complete: n = 403 | p = 0.002 | η² = 0.019 |
|  | sex | female: 11% (43) male: 47% (183) unknown: 42% (167) | female: 15% (29) male: 41% (79) unknown: 44% (85) | female: 9.4% (88) male: 46% (431) unknown: 45% (421) | ns (p = 0.11) | V = 0.079 |
|  | pathology grade | G1: 3.1% (12) G2: 5.6% (22) G3: 19% (76) unknown: 72% (283) | G1: 1.6% (3) G2: 5.7% (11) G3: 12% (24) unknown: 80% (155) | G1: 4.9% (46) G2: 9.9% (93) G3: 12% (113) unknown: 73% (688) | p = 0.002 | V = 0.11 |
|  | histology grade | low grade: 28% (112) high grade: 15% (58) unknown: 57% (223) | low grade: 23% (44) high grade: 25% (48) unknown: 52% (101) | low grade: 26% (246) high grade: 13% (126) unknown: 60% (568) | p = 0.0092 | V = 0.13 |
|  | history of BCG therapy | no: 28% (111) yes: 8.4% (33) unknown: 63% (249) | no: 32% (61) yes: 7.3% (14) unknown: 61% (118) | no: 21% (200) yes: 4.4% (41) unknown: 74% (699) | ns (p = 0.48) | V = 0.063 |
|  | cystectomy | no: 38% (149) yes: 3.8% (15) unknown: 58% (229) | no: 38% (74) yes: 2.6% (5) unknown: 59% (114) | no: 32% (305) yes: 2.1% (20) unknown: 65% (615) | ns (p = 0.53) | V = 0.047 |
| MIBC | age, years | 68 [IQR: 59 - 74] range: 22 - 90 complete: n = 459 | 68 [IQR: 60 - 75] range: 35 - 94 complete: n = 403 | 65 [IQR: 58 - 74] range: 28 - 90 complete: n = 495 | p = 0.04 | η² = 0.0051 |
|  | sex | female: 11% (105) male: 40% (392) unknown: 49% (474) | female: 13% (112) male: 34% (287) unknown: 52% (433) | female: 10% (113) male: 35% (392) unknown: 54% (602) | ns (p = 0.097) | V = 0.067 |
|  | race | Asian: 0.62% (6) Black or African American: 1.2% (12) White: 27% (261) Other: 1.2% (12) unknown: 70% (680) | Asian: 0.72% (6) Black or African American: 1.9% (16) White: 24% (202) Other: 0.84% (7) unknown: 72% (601) | Asian: 3.9% (43) Black or African American: 1.7% (19) White: 24% (262) Other: 1.1% (12) unknown: 70% (771) | p < 0.001 | V = 0.11 |
|  | smoking history | never: 7.3% (71) current or previous: 19% (185) unknown: 74% (715) | never: 7.3% (61) current or previous: 18% (152) unknown: 74% (619) | never: 8.9% (98) current or previous: 20% (221) unknown: 71% (788) | ns (p = 0.75) | V = 0.027 |
|  | cancer tissue type | bladder: 94% (910) urinary organ, non-bladder: 5.7% (55) non-urinary organ, distant metastasis: 0.62% (6) | bladder: 94% (783) urinary organ, non-bladder: 5% (42) non-urinary organ, distant metastasis: 0.84% (7) | bladder: 92% (1014) urinary organ, non-bladder: 7.8% (86) non-urinary organ, distant metastasis: 0.63% (7) | ns (p = 0.2) | V = 0.024 |
|  | pathology grade | G1: 0% (0) G2: 0.21% (2) G3: 10% (99) unknown: 90% (870) | G1: 0% (0) G2: 0.36% (3) G3: 11% (95) unknown: 88% (734) | G1: 0% (0) G2: 0.63% (7) G3: 8.9% (98) unknown: 91% (1002) | ns (p = 0.34) | V = 0.093 |
|  | histology grade | low grade: 0.62% (6) high grade: 17% (166) unknown: 82% (799) | low grade: 0.72% (6) high grade: 18% (152) unknown: 81% (674) | low grade: 2.6% (29) high grade: 16% (180) unknown: 81% (898) | p < 0.001 | V = 0.18 |
|  | pT stage | T2: 32% (310) T3: 23% (224) T4: 9.2% (89) Ta/Tis/T1: 0% (0) T3/T4: 1.5% (15) unknown: 34% (333) | T2: 34% (285) T3: 25% (205) T4: 8.8% (73) Ta/Tis/T1: 0% (0) T3/T4: 0.96% (8) unknown: 31% (261) | T2: 34% (374) T3: 17% (184) T4: 5.9% (65) Ta/Tis/T1: 0% (0) T3/T4: 0.18% (2) unknown: 44% (482) | p = 0.0014 | V = 0.059 |
|  | pN stage | N0: 25% (243) N1+: 15% (146) unknown: 60% (582) | N0: 28% (231) N1+: 12% (103) unknown: 60% (498) | N0: 24% (269) N1+: 10% (112) unknown: 66% (726) | ns (p = 0.097) | V = 0.076 |
|  | pM stage | M0: 16% (158) M1: 3.2% (31) unknown: 81% (782) | M0: 16% (137) M1: 2.5% (21) unknown: 81% (674) | M0: 16% (173) M1: 1.9% (21) unknown: 82% (913) | ns (p = 0.37) | V = 0.066 |
|  | neoadjuvant systemic chemotherapy | no: 28% (269) yes: 12% (113) unknown: 61% (589) | no: 31% (261) yes: 11% (94) unknown: 57% (477) | no: 34% (373) yes: 12% (129) unknown: 55% (605) | ns (p = 0.47) | V = 0.036 |
|  | history of BCG therapy | no: 15% (142) yes: 2.9% (28) unknown: 82% (801) | no: 15% (126) yes: 2.2% (18) unknown: 83% (688) | no: 16% (174) yes: 4.2% (46) unknown: 80% (887) | ns (p = 0.2) | V = 0.088 |
|  | cystectomy | no: 4.4% (43) yes: 13% (128) unknown: 82% (800) | no: 4.1% (34) yes: 13% (106) unknown: 83% (692) | no: 4.2% (47) yes: 7.5% (83) unknown: 88% (977) | ns (p = 0.11) | V = 0.11 |
|  | adjuvant systemic chemotherapy | no: 13% (123) yes: 30% (295) unknown: 57% (553) | no: 15% (123) yes: 24% (200) unknown: 61% (509) | no: 16% (177) yes: 31% (340) unknown: 53% (590) | ns (p = 0.097) | V = 0.069 |
| ^a^pT, pN, pM: pathological tumor, node, and metastasis stages; BCG: Bacillus Calmette-Guerin. | | | | | | |
| ^b^NMIBC: Dressler 2024: n = 35, E-MTAB-4321: n = 144, Groeneveld 2024: n = 14, NA: n = 6, GSE120736: n = 16, GSE128959: n = 52, GSE13507: n = 14, GSE32548: n = 15, GSE48075: n = 11, GSE83586: n = 13, GSE86411: n = 41, Stroggilos 2020: n = 32  MIBC: BCAN: n = 64, Dressler 2024: n = 34, E-MTAB-4321: n = 9, Groeneveld 2024: n = 40, NA: n = 16, GSE120736: n = 20, GSE124305: n = 44, GSE128192: n = 34, GSE128701: n = 50, GSE128959: n = 21, GSE13507: n = 28, GSE198269: n = 131, GSE203149: n = 52, GSE32548: n = 19, GSE48075: n = 23, GSE48276: n = 36, GSE83586: n = 68, GSE87304: n = 79, IMvigor: n = 75, Stroggilos 2020: n = 8, TCGA BLCA: n = 120 | | | | | | |
| ^c^NMIBC: Dressler 2024: n = 26, E-MTAB-4321: n = 75, Groeneveld 2024: n = 2, NA: n = 2, GSE120736: n = 8, GSE128959: n = 14, GSE13507: n = 11, GSE32548: n = 11, GSE48075: n = 3, GSE83586: n = 3, GSE86411: n = 28, Stroggilos 2020: n = 10  MIBC: BCAN: n = 47, Dressler 2024: n = 24, E-MTAB-4321: n = 6, Groeneveld 2024: n = 29, NA: n = 14, GSE120736: n = 20, GSE124305: n = 44, GSE128192: n = 37, GSE128701: n = 34, GSE128959: n = 10, GSE13507: n = 15, GSE198269: n = 88, GSE203149: n = 34, GSE32548: n = 12, GSE48075: n = 32, GSE48276: n = 33, GSE83586: n = 79, GSE87304: n = 92, IMvigor: n = 56, Stroggilos 2020: n = 9, TCGA BLCA: n = 117 | | | | | | |
| ^d^NMIBC: Dressler 2024: n = 106, E-MTAB-4321: n = 241, Groeneveld 2024: n = 71, NA: n = 13, GSE120736: n = 60, GSE128959: n = 88, GSE13507: n = 78, GSE32548: n = 66, GSE48075: n = 56, GSE83586: n = 42, GSE86411: n = 63, Stroggilos 2020: n = 56  MIBC: BCAN: n = 63, Dressler 2024: n = 17, E-MTAB-4321: n = 1, Groeneveld 2024: n = 13, NA: n = 8, GSE120736: n = 21, GSE124305: n = 45, GSE128192: n = 41, GSE128701: n = 52, GSE128959: n = 4, GSE13507: n = 19, GSE198269: n = 175, GSE203149: n = 85, GSE32548: n = 7, GSE48075: n = 17, GSE48276: n = 47, GSE83586: n = 96, GSE87304: n = 134, IMvigor: n = 90, Stroggilos 2020: n = 2, TCGA BLCA: n = 170 | | | | | | |
| ^e^Comparison of bladder cancer clusters within the NMIBC and MIBC collectives, the 'unknown' category was excluded. | | | | | | |

Table 17: Demographic characteristic of bladder cancer clusters in single cohorts. Qualitative variables are presented as percentages and counts of their categories within the complete observation set in a cluster. Quantitative variables are shown as medians with interquartile ranges and ranges. Statistical significance was determined by chi-square test with Cramer's V effect size statistic or Kruskal-Wallis test with eta-square effect size metric. P values were corrected for multiple comparisons with the false discovery rate method.

| **Cohort** | **Variable** | **#1** | **#2** | **#3** | **Significance^a^** | **Effect size^a^** |
| --- | --- | --- | --- | --- | --- | --- |
| TCGA BLCA | patients, N | 120 | 117 | 170 |  |  |
|  | age, years | 70 [IQR: 63 - 77] range: 48 - 90 complete: n = 120 | 68 [IQR: 61 - 76] range: 43 - 90 complete: n = 117 | 67 [IQR: 59 - 75] range: 34 - 89 complete: n = 170 | ns (p = 0.17) | η² = 0.0073 |
|  | sex | female: 26% (31) male: 74% (89) | female: 31% (36) male: 69% (81) | female: 24% (40) male: 76% (130) | ns (p = 0.39) | V = 0.068 |
|  | race | Asian: 1.7% (2) Black or African American: 3.3% (4) White: 94% (113) unknown: 0.83% (1) | Asian: 3.4% (4) Black or African American: 9.4% (11) White: 82% (96) unknown: 5.1% (6) | Asian: 22% (37) Black or African American: 4.7% (8) White: 68% (115) unknown: 5.9% (10) | p < 0.001 | V = 0.24 |
|  | smoking history | never: 22% (26) current or previous: 76% (91) unknown: 2.5% (3) | never: 26% (31) current or previous: 68% (80) unknown: 5.1% (6) | never: 31% (52) current or previous: 67% (114) unknown: 2.4% (4) | ns (p = 0.32) | V = 0.085 |
| IMvigor | patients, N | 75 | 56 | 90 |  |  |
|  | sex | female: 21% (16) male: 79% (59) | female: 16% (9) male: 84% (47) | female: 30% (27) male: 70% (63) | ns (p = 0.4) | V = 0.14 |
|  | race | Asian: 2.7% (2) Black or African American: 4% (3) Other: 5.3% (4) White: 88% (66) | Asian: 1.8% (1) Black or African American: 0% (0) Other: 1.8% (1) White: 96% (54) | Asian: 3.3% (3) Black or African American: 3.3% (3) Other: 4.4% (4) White: 89% (80) | ns (p = 0.71) | V = 0.092 |
|  | smoking history | never: 32% (24) current or previous: 68% (51) | never: 27% (15) current or previous: 73% (41) | never: 36% (32) current or previous: 64% (58) | ns (p = 0.71) | V = 0.074 |
| GSE13507 | patients, N | 51 | 31 | 106 |  |  |
|  | age, years | 66 [IQR: 59 - 72] range: 40 - 85 complete: n = 42 | 71 [IQR: 64 - 76] range: 37 - 82 complete: n = 26 | 66 [IQR: 59 - 74] range: 24 - 88 complete: n = 97 | ns (p = 0.19) | η² = 0.0069 |
|  | sex | female: 16% (8) male: 67% (34) unknown: 18% (9) | female: 26% (8) male: 58% (18) unknown: 16% (5) | female: 13% (14) male: 78% (83) unknown: 8.5% (9) | ns (p = 0.19) | V = 0.15 |
| GSE48075 | patients, N | 34 | 35 | 73 |  |  |
|  | age, years | 72 [IQR: 67 - 75] range: 43 - 83 complete: n = 24 | 69 [IQR: 65 - 77] range: 53 - 89 complete: n = 32 | 64 [IQR: 58 - 72] range: 44 - 86 complete: n = 17 | ns (p = 0.24) | η² = 0.0059 |
| GSE48276 | patients, N | 36 | 33 | 47 |  |  |
|  | age, years | 67 [IQR: 59 - 69] range: 48 - 83 complete: n = 33 | 68 [IQR: 63 - 70] range: 44 - 73 complete: n = 17 | 67 [IQR: 60 - 74] range: 41 - 90 complete: n = 23 | ns (p = 0.87) | η² = -0.013 |
|  | sex | female: 17% (6) male: 75% (27) unknown: 8.3% (3) | female: 12% (4) male: 39% (13) unknown: 48% (16) | female: 8.5% (4) male: 40% (19) unknown: 51% (24) | ns (p = 0.87) | V = 0.062 |
|  | race | Asian: 2.8% (1) Black or African American: 2.8% (1) White: 86% (31) unknown: 8.3% (3) | Asian: 0% (0) Black or African American: 3% (1) White: 48% (16) unknown: 48% (16) | Asian: 0% (0) Black or African American: 2.1% (1) White: 47% (22) unknown: 51% (24) | ns (p = 0.87) | V = 0.099 |
| GSE87304 | patients, N | 79 | 92 | 134 |  |  |
|  | age, years | 62 [IQR: 55 - 71] range: 22 - 81 complete: n = 78 | 64 [IQR: 57 - 70] range: 35 - 81 complete: n = 92 | 62 [IQR: 56 - 69] range: 33 - 84 complete: n = 130 | ns (p = 0.53) | η² = -0.0024 |
| GSE120736 | patients, N | 36 | 28 | 81 |  |  |
|  | sex | female: 17% (6) male: 83% (30) | female: 25% (7) male: 75% (21) | female: 14% (11) male: 86% (70) | ns (p = 0.37) | V = 0.12 |
| GSE124305 | patients, N | 44 | 44 | 45 |  |  |
|  | sex | female: 27% (12) male: 73% (32) | female: 36% (16) male: 64% (28) | female: 20% (9) male: 80% (36) | ns (p = 0.23) | V = 0.15 |
| GSE128701 | patients, N | 50 | 34 | 52 |  |  |
|  | age, years | 69 [IQR: 60 - 78] range: 40 - 87 complete: n = 48 | 73 [IQR: 64 - 79] range: 54 - 93 complete: n = 34 | 69 [IQR: 57 - 75] range: 39 - 89 complete: n = 51 | ns (p = 0.27) | η² = 0.0048 |
|  | sex | female: 18% (9) male: 80% (40) unknown: 2% (1) | female: 29% (10) male: 71% (24) unknown: 0% (0) | female: 15% (8) male: 85% (44) unknown: 0% (0) | ns (p = 0.27) | V = 0.14 |
| E-MTAB-4321 | patients, N | 153 | 81 | 242 |  |  |
|  | age, years | 69 [IQR: 61 - 76] range: 24 - 96 complete: n = 153 | 72 [IQR: 65 - 81] range: 46 - 94 complete: n = 81 | 69 [IQR: 62 - 76] range: 29 - 89 complete: n = 242 | ns (p = 0.065) | η² = 0.01 |
|  | sex | female: 24% (37) male: 76% (116) | female: 26% (21) male: 74% (60) | female: 21% (51) male: 79% (191) | ns (p = 0.6) | V = 0.046 |
| Groeneveld 2024 mRNA | patients, N | 60 | 37 | 101 |  |  |
|  | age, years | 70 [IQR: 61 - 76] range: 52 - 95 complete: n = 54 | 69 [IQR: 63 - 78] range: 45 - 89 complete: n = 31 | 61 [IQR: 56 - 71] range: 30 - 84 complete: n = 84 | p = 0.0036 | η² = 0.054 |
|  | sex | female: 15% (9) male: 75% (45) unknown: 10% (6) | female: 24% (9) male: 59% (22) unknown: 16% (6) | female: 9.9% (10) male: 73% (74) unknown: 17% (17) | ns (p = 0.09) | V = 0.17 |
| BCAN | patients, N | 64 | 47 | 63 |  |  |
|  | age, years | 66 [IQR: 58 - 72] range: 28 - 81 complete: n = 64 | 70 [IQR: 64 - 75] range: 48 - 83 complete: n = 47 | 66 [IQR: 59 - 74] range: 28 - 85 complete: n = 63 | ns (p = 0.17) | η² = 0.019 |
|  | sex | female: 16% (10) male: 83% (53) unknown: 1.6% (1) | female: 34% (16) male: 66% (31) unknown: 0% (0) | female: 24% (15) male: 76% (48) unknown: 0% (0) | ns (p = 0.17) | V = 0.17 |
|  | race | Asian: 1.6% (1) Black or African American: 6.2% (4) Other: 12% (8) White: 80% (51) | Asian: 2.1% (1) Black or African American: 8.5% (4) Other: 13% (6) White: 77% (36) | Asian: 4.8% (3) Black or African American: 11% (7) Other: 13% (8) White: 71% (45) | ns (p = 0.88) | V = 0.083 |
|  | smoking history | never: 33% (21) current or previous: 67% (43) unknown: 0% (0) | never: 32% (15) current or previous: 66% (31) unknown: 2.1% (1) | never: 22% (14) current or previous: 78% (49) unknown: 0% (0) | ns (p = 0.45) | V = 0.11 |
| Groeneveld 2024 protein | patients, N | 23 | 18 | 22 |  |  |
|  | age, years | 66 [IQR: 58 - 74] range: 47 - 89 complete: n = 22 | 69 [IQR: 57 - 78] range: 45 - 89 complete: n = 16 | 69 [IQR: 57 - 74] range: 49 - 82 complete: n = 21 | ns (p = 0.87) | η² = -0.027 |
|  | sex | female: 8.7% (2) male: 87% (20) unknown: 4.3% (1) | female: 11% (2) male: 78% (14) unknown: 11% (2) | female: 14% (3) male: 82% (18) unknown: 4.5% (1) | ns (p = 0.87) | V = 0.07 |
| Stroggilos 2020 | patients, N | 40 | 19 | 58 |  |  |
|  | sex | female: 5% (2) male: 75% (30) unknown: 20% (8) | female: 16% (3) male: 37% (7) unknown: 47% (9) | female: 16% (9) male: 81% (47) unknown: 3.4% (2) | ns (p = 0.15) | V = 0.2 |
| ^a^Comparison of bladder cancer clusters, the 'unknown' category was excluded. | | | | | | |

Table 18: Therapy in bladder cancer clusters in single cohorts. Qualitative variables are presented as percentages and counts of their categories within the complete observation set in a cluster. Quantitative variables are shown as medians with interquartile ranges and ranges. Statistical significance was determined by chi-square test with Cramer's V effect size statistic or Kruskal-Wallis test with eta-square effect size metric. P values were corrected for multiple comparisons with the false discovery rate method.

| **Cohort** | **Variable^a^** | **#1** | **#2** | **#3** | **Significance^b^** | **Effect size^b^** |
| --- | --- | --- | --- | --- | --- | --- |
| TCGA BLCA | patients, N | 120 | 117 | 170 |  |  |
|  | neoadjuvant systemic chemotherapy | no: 97% (116) yes: 3.3% (4) | no: 97% (114) yes: 2.6% (3) | no: 98% (167) yes: 1.8% (3) | ns (p = 0.8) | V = 0.033 |
|  | history of BCG therapy | no: 61% (73) yes: 11% (13) unknown: 28% (34) | no: 62% (72) yes: 8.5% (10) unknown: 30% (35) | no: 68% (115) yes: 8.2% (14) unknown: 24% (41) | ns (p = 0.8) | V = 0.047 |
|  | adjuvant systemic chemotherapy | no: 62% (75) yes: 38% (45) | no: 72% (84) yes: 28% (33) | no: 81% (137) yes: 19% (33) | p = 0.011 | V = 0.17 |
| IMvigor | patients, N | 75 | 56 | 90 |  |  |
|  | neoadjuvant systemic chemotherapy | no: 33% (25) yes: 44% (33) unknown: 23% (17) | no: 27% (15) yes: 48% (27) unknown: 25% (14) | no: 18% (16) yes: 61% (55) unknown: 21% (19) | ns (p = 0.081) | V = 0.18 |
|  | history of BCG therapy | no: 80% (60) yes: 20% (15) | no: 86% (48) yes: 14% (8) | no: 64% (58) yes: 36% (32) | p = 0.03 | V = 0.2 |
|  | adjuvant systemic chemotherapy | no: 23% (17) yes: 77% (58) | no: 21% (12) yes: 79% (44) | no: 19% (17) yes: 81% (73) | ns (p = 0.87) | V = 0.035 |
| GSE13507 | patients, N | 51 | 31 | 106 |  |  |
|  | adjuvant systemic chemotherapy | no: 61% (31) yes: 22% (11) unknown: 18% (9) | no: 71% (22) yes: 13% (4) unknown: 16% (5) | no: 81% (86) yes: 10% (11) unknown: 8.5% (9) | ns (p = 0.12) | V = 0.16 |
| GSE48276 | patients, N | 36 | 33 | 47 |  |  |
|  | neoadjuvant systemic chemotherapy | no: 44% (16) yes: 11% (4) unknown: 44% (16) | no: 33% (11) yes: 9.1% (3) unknown: 58% (19) | no: 21% (10) yes: 19% (9) unknown: 60% (28) | ns (p = 0.18) | V = 0.25 |
| E-MTAB-4321 | patients, N | 153 | 81 | 242 |  |  |
|  | history of BCG therapy | no: 78% (120) yes: 22% (33) | no: 83% (67) yes: 17% (14) | no: 83% (201) yes: 17% (41) | ns (p = 0.54) | V = 0.051 |
|  | cystectomy | no: 93% (142) yes: 7.2% (11) | no: 89% (72) yes: 11% (9) | no: 95% (230) yes: 5% (12) | ns (p = 0.43) | V = 0.08 |
| Groeneveld 2024 mRNA | patients, N | 60 | 37 | 101 |  |  |
|  | cystectomy | no: 33% (20) yes: 57% (34) unknown: 10% (6) | no: 16% (6) yes: 68% (25) unknown: 16% (6) | no: 66% (67) yes: 17% (17) unknown: 17% (17) | p < 0.001 | V = 0.5 |
| BCAN | patients, N | 64 | 47 | 63 |  |  |
|  | neoadjuvant systemic chemotherapy | no: 52% (33) yes: 44% (28) unknown: 4.7% (3) | no: 62% (29) yes: 36% (17) unknown: 2.1% (1) | no: 73% (46) yes: 27% (17) unknown: 0% (0) | ns (p = 0.17) | V = 0.16 |
|  | cystectomy | no: 30% (19) yes: 67% (43) unknown: 3.1% (2) | no: 53% (25) yes: 47% (22) unknown: 0% (0) | no: 63% (40) yes: 37% (23) unknown: 0% (0) | p = 0.0039 | V = 0.28 |
|  | adjuvant systemic chemotherapy | no: 22% (14) yes: 78% (50) | no: 34% (16) yes: 66% (31) | no: 24% (15) yes: 76% (48) | ns (p = 0.38) | V = 0.11 |
| Groeneveld 2024 protein | patients, N | 23 | 18 | 22 |  |  |
|  | cystectomy | no: 48% (11) yes: 48% (11) unknown: 4.3% (1) | no: 28% (5) yes: 61% (11) unknown: 11% (2) | no: 68% (15) yes: 27% (6) unknown: 4.5% (1) | ns (p = 0.075) | V = 0.3 |
| ^a^BCG: Bacillus Calmette-Guerin. | | | | | | |
| ^b^Comparison of bladder cancer clusters, the 'unknown' category was excluded. | | | | | | |

Table 19: Pathological characteristic of bladder cancer clusters in single cohorts. Qualitative variables are presented as percentages and counts of their categories within the complete observation set in a cluster. Quantitative variables are shown as medians with interquartile ranges and ranges. Statistical significance was determined by chi-square test with Cramer V effect size statistic or Kruskal-Wallis test with eta-square effect size metric. P values were corrected for multiple comparisons with the false discovery rate method.

| **Cohort** | **Variable^a^** | **#1** | **#2** | **#3** | **Significance^b^** | **Effect size^b^** |
| --- | --- | --- | --- | --- | --- | --- |
| TCGA BLCA | histology grade | low grade: 0% (0) high grade: 99% (119) unknown: 0.83% (1) | low grade: 0.85% (1) high grade: 99% (116) unknown: 0% (0) | low grade: 12% (20) high grade: 87% (148) unknown: 1.2% (2) | p < 0.001 | V = 0.25 |
|  | histology subtype | Non-Papillary: 78% (93) Papillary: 20% (24) unknown: 2.5% (3) | Non-Papillary: 83% (97) Papillary: 15% (18) unknown: 1.7% (2) | Non-Papillary: 47% (80) Papillary: 53% (90) unknown: 0% (0) | p < 0.001 | V = 0.37 |
|  | pT stage | T2: 16% (19) T3: 60% (72) T4: 18% (22) unknown: 5.8% (7) | T2: 26% (30) T3: 52% (61) T4: 15% (18) unknown: 6.8% (8) | T2: 41% (69) T3: 36% (61) T4: 11% (18) unknown: 13% (22) | p < 0.001 | V = 0.13 |
|  | pN stage | N0: 48% (58) N1+: 45% (54) unknown: 6.7% (8) | N0: 59% (69) N1+: 32% (37) unknown: 9.4% (11) | N0: 64% (109) N1+: 22% (38) unknown: 14% (23) | p = 0.0015 | V = 0.19 |
| GSE13507 | histology grade | low grade: 35% (18) high grade: 47% (24) unknown: 18% (9) | low grade: 42% (13) high grade: 42% (13) unknown: 16% (5) | low grade: 70% (74) high grade: 22% (23) unknown: 8.5% (9) | p < 0.001 | V = 0.31 |
|  | invasiveness | non-muscle invasive: 27% (14) muscle invasive: 55% (28) unknown: 18% (9) | non-muscle invasive: 35% (11) muscle invasive: 48% (15) unknown: 16% (5) | non-muscle invasive: 74% (78) muscle invasive: 18% (19) unknown: 8.5% (9) | p < 0.001 | V = 0.44 |
|  | pT stage | Ta/Tis/T1: 27% (14) T2: 33% (17) T3: 12% (6) T4: 9.8% (5) unknown: 18% (9) | Ta/Tis/T1: 35% (11) T2: 19% (6) T3: 19% (6) T4: 6.5% (2) unknown: 19% (6) | Ta/Tis/T1: 74% (78) T2: 7.5% (8) T3: 6.6% (7) T4: 3.8% (4) unknown: 8.5% (9) | p < 0.001 | V = 0.22 |
|  | pN stage | N0: 71% (36) N1+: 9.8% (5) unknown: 20% (10) | N0: 65% (20) N1+: 19% (6) unknown: 16% (5) | N0: 88% (93) N1+: 3.8% (4) unknown: 8.5% (9) | p = 0.026 | V = 0.22 |
| GSE32548 | pathology grade | G1: 2.9% (1) G2: 15% (5) G3: 82% (28) | G1: 4.3% (1) G2: 22% (5) G3: 74% (17) | G1: 18% (13) G2: 42% (31) G3: 41% (30) | p < 0.001 | V = 0.19 |
|  | invasiveness | non-muscle invasive: 44% (15) muscle invasive: 56% (19) unknown: 0% (0) | non-muscle invasive: 48% (11) muscle invasive: 52% (12) unknown: 0% (0) | non-muscle invasive: 89% (66) muscle invasive: 9.5% (7) unknown: 1.4% (1) | p < 0.001 | V = 0.47 |
|  | pT stage | Ta/Tis/T1: 44% (15) T2: 56% (19) unknown: 0% (0) | Ta/Tis/T1: 48% (11) T2: 52% (12) unknown: 0% (0) | Ta/Tis/T1: 89% (66) T2: 9.5% (7) unknown: 1.4% (1) | p < 0.001 | V = 0.47 |
| GSE48075 | invasiveness | non-muscle invasive: 32% (11) muscle invasive: 68% (23) | non-muscle invasive: 8.6% (3) muscle invasive: 91% (32) | non-muscle invasive: 77% (56) muscle invasive: 23% (17) | p < 0.001 | V = 0.58 |
|  | pT stage | Ta/Tis/T1: 32% (11) T2: 41% (14) T3: 24% (8) T4: 2.9% (1) | Ta/Tis/T1: 8.6% (3) T2: 49% (17) T3: 31% (11) T4: 11% (4) | Ta/Tis/T1: 77% (56) T2: 14% (10) T3: 5.5% (4) T4: 4.1% (3) | p < 0.001 | V = 0.29 |
| GSE83586 | pathology grade | G2: 1.2% (1) G3: 89% (73) unknown: 9.8% (8) | G2: 3.5% (3) G3: 93% (79) unknown: 3.5% (3) | G2: 17% (24) G3: 81% (114) unknown: 1.4% (2) | p < 0.001 | V = 0.25 |
|  | invasiveness | non-muscle invasive: 16% (13) muscle invasive: 83% (68) unknown: 1.2% (1) | non-muscle invasive: 3.5% (3) muscle invasive: 93% (79) unknown: 3.5% (3) | non-muscle invasive: 30% (42) muscle invasive: 69% (96) unknown: 1.4% (2) | p < 0.001 | V = 0.28 |
|  | pT stage | Ta/Tis/T1: 16% (13) T2: 82% (67) T3: 1.2% (1) T4: 0% (0) unknown: 1.2% (1) | Ta/Tis/T1: 3.5% (3) T2: 92% (78) T3: 0% (0) T4: 1.2% (1) unknown: 3.5% (3) | Ta/Tis/T1: 30% (42) T2: 69% (96) T3: 0% (0) T4: 0% (0) unknown: 1.4% (2) | p < 0.001 | V = 0.14 |
| GSE86411 | histology subtype | conventional: 63% (26) micropapillary: 37% (15) | conventional: 96% (27) micropapillary: 3.6% (1) | conventional: 57% (36) micropapillary: 43% (27) | p = 0.0013 | V = 0.32 |
| GSE120736 | histology grade | low grade: 17% (6) high grade: 64% (23) unknown: 19% (7) | low grade: 7.1% (2) high grade: 89% (25) unknown: 3.6% (1) | low grade: 40% (32) high grade: 58% (47) unknown: 2.5% (2) | p = 0.0037 | V = 0.29 |
|  | invasiveness | non-muscle invasive: 44% (16) muscle invasive: 56% (20) | non-muscle invasive: 29% (8) muscle invasive: 71% (20) | non-muscle invasive: 74% (60) muscle invasive: 26% (21) | p < 0.001 | V = 0.37 |
|  | pT stage | Ta/Tis/T1: 36% (13) T2: 19% (7) T3: 22% (8) T4: 14% (5) unknown: 8.3% (3) | Ta/Tis/T1: 25% (7) T2: 36% (10) T3: 25% (7) T4: 11% (3) unknown: 3.6% (1) | Ta/Tis/T1: 72% (58) T2: 8.6% (7) T3: 14% (11) T4: 3.7% (3) unknown: 2.5% (2) | p = 0.0011 | V = 0.2 |
| GSE128192 | histology subtype | conventional: 71% (24) sarcomatoid: 29% (10) | conventional: 57% (21) sarcomatoid: 43% (16) | conventional: 95% (39) sarcomatoid: 4.9% (2) | p < 0.001 | V = 0.36 |
| GSE128959 | pathology grade | G1: 1.3% (1) G2: 13% (10) G3: 81% (61) unknown: 4% (3) | G1: 0% (0) G2: 17% (4) G3: 83% (20) unknown: 0% (0) | G1: 2.2% (2) G2: 34% (32) G3: 62% (58) unknown: 1.1% (1) | p = 0.032 | V = 0.12 |
|  | invasiveness | non-muscle invasive: 69% (52) muscle invasive: 28% (21) unknown: 2.7% (2) | non-muscle invasive: 58% (14) muscle invasive: 42% (10) unknown: 0% (0) | non-muscle invasive: 95% (88) muscle invasive: 4.3% (4) unknown: 1.1% (1) | p < 0.001 | V = 0.36 |
|  | pT stage | Ta/Tis/T1: 69% (52) T2: 19% (14) T3: 5.3% (4) T4: 4% (3) unknown: 2.7% (2) | Ta/Tis/T1: 58% (14) T2: 33% (8) T3: 8.3% (2) T4: 0% (0) unknown: 0% (0) | Ta/Tis/T1: 95% (88) T2: 4.3% (4) T3: 0% (0) T4: 0% (0) unknown: 1.1% (1) | p < 0.001 | V = 0.18 |
| E-MTAB-4321 | histology grade | low grade: 61% (94) high grade: 38% (58) unknown: 0.65% (1) | low grade: 42% (34) high grade: 57% (46) unknown: 1.2% (1) | low grade: 62% (149) high grade: 36% (88) unknown: 2.1% (5) | p = 0.0053 | V = 0.15 |
|  | histology subtype | papillary: 82% (126) solid: 5.9% (9) mixed: 3.3% (5) unknown: 8.5% (13) | papillary: 75% (61) solid: 4.9% (4) mixed: 3.7% (3) unknown: 16% (13) | papillary: 95% (230) solid: 0.41% (1) mixed: 0% (0) unknown: 4.5% (11) | p = 0.0037 | V = 0.1 |
|  | invasiveness | non-muscle invasive: 94% (144) muscle invasive: 5.9% (9) | non-muscle invasive: 93% (75) muscle invasive: 7.4% (6) | non-muscle invasive: 100% (241) muscle invasive: 0.41% (1) | p = 0.0043 | V = 0.16 |
| Groeneveld 2024 mRNA | invasiveness | non-muscle invasive: 23% (14) muscle invasive: 67% (40) unknown: 10% (6) | non-muscle invasive: 5.4% (2) muscle invasive: 78% (29) unknown: 16% (6) | non-muscle invasive: 70% (71) muscle invasive: 13% (13) unknown: 17% (17) | p < 0.001 | V = 0.66 |
|  | pT stage | Ta/Tis/T1: 23% (14) T2: 23% (14) T3: 27% (16) T4: 17% (10) unknown: 10% (6) | Ta/Tis/T1: 5.4% (2) T2: 22% (8) T3: 41% (15) T4: 16% (6) unknown: 16% (6) | Ta/Tis/T1: 70% (71) T2: 7.9% (8) T3: 2% (2) T4: 3% (3) unknown: 17% (17) | p < 0.001 | V = 0.34 |
|  | pN stage | N0: 38% (23) N1+: 30% (18) unknown: 32% (19) | N0: 49% (18) N1+: 24% (9) unknown: 27% (10) | N0: 55% (56) N1+: 0.99% (1) unknown: 44% (44) | p < 0.001 | V = 0.45 |
|  | pM stage | M0: 62% (37) M1: 25% (15) unknown: 13% (8) | M0: 54% (20) M1: 30% (11) unknown: 16% (6) | M0: 70% (71) M1: 5.9% (6) unknown: 24% (24) | p = 0.0015 | V = 0.29 |
| Groeneveld 2024 protein | invasiveness | non-muscle invasive: 26% (6) muscle invasive: 70% (16) unknown: 4.3% (1) | non-muscle invasive: 11% (2) muscle invasive: 78% (14) unknown: 11% (2) | non-muscle invasive: 59% (13) muscle invasive: 36% (8) unknown: 4.5% (1) | p = 0.033 | V = 0.4 |
| Dressler 2024 | invasiveness | muscle invasive: 49% (34) non-muscle invasive: 51% (35) | muscle invasive: 48% (24) non-muscle invasive: 52% (26) | muscle invasive: 14% (17) non-muscle invasive: 86% (106) | p < 0.001 | V = 0.37 |
|  | pT stage | Ta/Tis/T1: 51% (35) T2: 28% (19) T3/T4: 22% (15) | Ta/Tis/T1: 52% (26) T2: 32% (16) T3/T4: 16% (8) | Ta/Tis/T1: 86% (106) T2: 12% (15) T3/T4: 1.6% (2) | p < 0.001 | V = 0.19 |
| Stroggilos 2020 | invasiveness | non-muscle invasive: 80% (32) muscle invasive: 20% (8) | non-muscle invasive: 53% (10) muscle invasive: 47% (9) | non-muscle invasive: 97% (56) muscle invasive: 3.4% (2) | p < 0.001 | V = 0.4 |
|  | pT stage | Ta/Tis/T1: 80% (32) T2: 20% (8) | Ta/Tis/T1: 53% (10) T2: 47% (9) | Ta/Tis/T1: 97% (56) T2: 3.4% (2) | p < 0.001 | V = 0.4 |
| ^a^pT, pN, pM: pathological tumor, node, and metastasis stages. | | | | | | |
| ^b^Comparison of bladder cancer clusters, the 'unknown' category was excluded. | | | | | | |

Table 20: Overall, tumor-specific, relapse-free, and MIBC progression-free survival in bladder cancer clusters. Differences in survival between bladder cancer clusters were assessed by Kaplan-Meier analysis and Peto-Peto test. In each cohort, the analysis was conducted for all patients, patients with Ta - T3 tumors, and non-muscle invasive bladder cancers (NMIBC) and muscle invasive bladder cancers (MIBC) subsets with at least 80 samples with complete survival information. P values were corrected for multiple testing with the false discovery rate method. Survival 25%, 50% (median), and 75% quantiles of surviving patients in the clusters are summarized. The table is available as a supplementary Excel file.

|  |
| --- |
|  |

Table 21: Numbers of mutated, amplified, and deleted genes in bladder cancer clusters. Median alteration counts with interquartile ranges and ranges are shown. Statistical significance of differences between bladder cancer clusters was determined by Kruskal-Wallis test with eta-square effect size statistic. P values were corrected for multiple comparisons with the false discovery rate method.

| **Cohort** | **Variable** | **#1** | **#2** | **#3** | **Significance** | **Effect size** |
| --- | --- | --- | --- | --- | --- | --- |
| TCGA BLCA | Samples, N | 120 | 117 | 170 |  |  |
|  | mutated genes | 160 [IQR: 88 - 280] range: 1 - 1200 complete: n = 119 | 170 [IQR: 110 - 320] range: 24 - 1200 complete: n = 117 | 160 [IQR: 76 - 280] range: 21 - 2900 complete: n = 169 | ns (p = 0.62) | η² = 0.0016 |
|  | amplified alleles | 250 [IQR: 54 - 640] range: 0 - 4500 complete: n = 119 | 190 [IQR: 40 - 460] range: 0 - 1100 complete: n = 115 | 220 [IQR: 19 - 580] range: 0 - 2700 complete: n = 170 | ns (p = 0.62) | η² = 0 |
|  | deleted alleles | 33 [IQR: 2.5 - 190] range: 0 - 1200 complete: n = 119 | 24 [IQR: 6.5 - 170] range: 0 - 980 complete: n = 115 | 28 [IQR: 5 - 170] range: 0 - 1200 complete: n = 170 | ns (p = 0.92) | η² = 0 |
| IMvigor | Samples, N | 57 | 44 | 79 |  |  |
|  | mutated genes | 4 [IQR: 3 - 6] range: 0 - 15 | 7 [IQR: 5 - 9] range: 0 - 16 | 5 [IQR: 4 - 8] range: 1 - 20 | p < 0.001 | η² = 0.069 |
| Groeneveld 2024 | Samples, N | 58 | 35 | 94 |  |  |
|  | amplified alleles | 310 [IQR: 66 - 720] range: 0 - 4700 | 330 [IQR: 100 - 760] range: 0 - 1900 | 22 [IQR: 0 - 340] range: 0 - 2600 | p < 0.001 | η² = 0.087 |
|  | deleted alleles | 110 [IQR: 0 - 350] range: 0 - 2400 | 22 [IQR: 0 - 470] range: 0 - 1500 | 66 [IQR: 0 - 710] range: 0 - 2500 | ns (p = 0.91) | η² = 0 |
| BCAN | Samples, N | 60 | 47 | 60 |  |  |
|  | mutated genes | 18 [IQR: 13 - 24] range: 8 - 55 | 18 [IQR: 15 - 25] range: 10 - 99 | 20 [IQR: 14 - 26] range: 6 - 92 | ns (p = 0.75) | η² = 0 |

Table 22: Enrichment analysis for somatic gene mutations in bladder cancer clusters in the TCGA BLCA, IMvigor, and BCAN cohorts. Actual frequencies of somatic mutations in bladder cancer clusters were compared with the expected random mutation frequencies simulated by n = 1000 permutations weighted by overall mutation counts in cancer samples. Enrichment score, i.e. ratio of the actual and expected mutation count, gauged the enrichment magnitude. Numbers and percentages of samples with mutations, expected numbers of samples with mutations, enrichment scores with 95% confidence intervals (CI), raw p values, and p values adjusted for multiple testing with the false discovery rate (FDR) method are presented. The table is available as s supplementary Excel file.

|  |
| --- |
|  |

Table 23: Genes significantly differentially regulated in bladder cancer clusters as compared with the cohort average shared by at least 10 bulk cancer transcriptome cohorts. Differences in log2-transformed expression levels between bladder cancer clusters were assessed by one-way ANOVA with eta-square effect size statistic. Differences in log2-transformed expression between a bladder cancer cluster and the respective cohort average were investigated by one-sample T test. P values were corrected for multiple comparisons with the false discovery rate method (FDR). Significantly differentially regulated features were defined by pFDR(ANOVA) < 0.05, eta-square >= 0.06, and pFDR(T test) < 0.05. Results of statistical hypothesis testing and log2 fold-regulation estimates of difference in expression levels between the clusters and cohort averages with 95% confidence intervals are listed. The table is available as a supplementary Excel file.

|  |
| --- |
|  |

Table 24: Meta-estimates of differential gene expression in bladder cancer clusters as compared with the cohort average. The meta-estimates were computed for genes found to be significantly regulated in at least 10 bulk cancer transcriptome cohorts with the DerSimonian-Lair algorithm fed with log2 fold-regulation estimates of differential expression with the respective standard errors obtained for 19 cancer samples collectives. Meta-estimates of log2 fold-regulation of expression with 95% confidence intervals are summarized. The table is available as a supplementary Excel file.

|  |
| --- |
|  |

Table 25: Proteins significantly differentially regulated in bladder cancer clusters as compared with the cohort average shared by at least 10 bulk cancer proteome cohorts. Differences in log2-transformed expression levels between bladder cancer clusters were assessed by one-way ANOVA with eta-square effect size statistic. Differences in log2-transformed expression between a bladder cancer cluster and the respective cohort average were investigated by one-sample T test. P values were corrected for multiple comparisons with the false discovery rate method (FDR). Significantly differentially regulated features were defined by pFDR(ANOVA) < 0.05, eta-square >= 0.06, and pFDR(T test) < 0.05. Results of statistical hypothesis testing and log2 fold-regulation estimates of difference in expression levels between the clusters and cohort averages with 95% confidence intervals are listed. The table is available as a supplementary Excel file.

|  |
| --- |
|  |

Table 26: Meta-estimates of differential protein expression in bladder cancer clusters as compared with the cohort average. The meta-estimates were computed for proteins found to be significantly regulated in at least two bulk cancer proteome cohorts with the DerSimonian-Lair algorithm fed with log2 fold-regulation estimates of differential expression with the respective standard errors obtained for 3 cancer samples collectives. Meta-estimates of log2 fold-regulation of expression with 95% confidence intervals are summarized. The table is available as a supplementary Excel file.

|  |
| --- |
|  |

Table 27: Assignment of genes found to be differentially regulated between bladder cancer clusters in at least 10 bulk cancer transcriptome cohorts to communities in a gene co-expression network analysis. A co-expression network was created for pairs of the genes that correlated with Spearman rho of at least 0.5 in a pooled data set of ComBat-processed log2-transformed expression values measures in 18 bulk cancer transcriptome cohorts. Communities of the network were identified by Leiden algorithm. The communities were named by their key biological features revealed by a biological process gene ontology term enrichment analysis. Network vertex importance was measured by degree (number of correlations with rho >= 0.5), hub score (overall correlation strength), and betweenness (number of the shortest paths between gene pairs that pass through a gene). Assignment of the common differentially regulated genes to the communities is summarized along with regulation status in bladder cancer clusters and the vertex importance metrics. Top 5 genes of each community with the largest degree values are presented. The entire table is available as a supplementary Excel file.

| **Community name** | **Gene symbol** | **Regulation in cluster #1** | **Regulation in cluster #2** | **Regulation in cluster #3** | **Degree** | **Hub score** | **Betweenness** |
| --- | --- | --- | --- | --- | --- | --- | --- |
| Community 1: Immunity | *CD53* | upregulated | upregulated | downregulated | 420 | 1 | 8689 |
|  | *CSF1R* | upregulated | upregulated | downregulated | 414 | 0.94 | 10311 |
|  | *ITGB2* | upregulated | upregulated | downregulated | 408 | 0.97 | 28197 |
|  | *AIF1* | upregulated | upregulated | downregulated | 398 | 0.96 | 4475 |
|  | *IL10RA* | upregulated | upregulated | downregulated | 396 | 0.96 | 4514 |
|  | *FCER1G* | upregulated | upregulated | downregulated | 396 | 0.93 | 9791 |
| Community 2: ECM, signaling | *C1R* | upregulated | upregulated | downregulated | 455 | 0.84 | 8636 |
|  | *VIM* | upregulated | upregulated | downregulated | 448 | 0.74 | 17107 |
|  | *ZEB2* | upregulated | ns | downregulated | 434 | 0.81 | 7207 |
|  | *C1S* | upregulated | upregulated | downregulated | 401 | 0.76 | 7774 |
|  | *ANXA6* | upregulated | upregulated | downregulated | 357 | 0.66 | 18996 |
| Community 3: Stress | *EMP3* | upregulated | upregulated | downregulated | 397 | 0.8 | 13382 |
|  | *GLIPR1* | upregulated | upregulated | downregulated | 304 | 0.63 | 10427 |
|  | *SERPING1* | upregulated | upregulated | downregulated | 270 | 0.62 | 8908 |
|  | *CCL2* | upregulated | upregulated | downregulated | 252 | 0.61 | 3954 |
|  | *MT2A* | upregulated | upregulated | downregulated | 200 | 0.41 | 7685 |
|  | *IFITM2* | upregulated | upregulated | downregulated | 200 | 0.48 | 3688 |
| Community 4: Cell cycle, mitosis | *CKS2* | ns | upregulated | ns | 98 | 2.2e-05 | 6108 |
|  | *RACGAP1* | ns | upregulated | ns | 92 | 2.4e-05 | 3091 |
|  | *FEN1* | ns | upregulated | ns | 92 | 2.2e-05 | 103 |
|  | *MELK* | ns | upregulated | ns | 91 | 4.4e-05 | 7480 |
|  | *FOXM1* | downregulated | upregulated | ns | 90 | 4.6e-05 | 1251 |
|  | *ANLN* | ns | upregulated | downregulated | 90 | 2e-04 | 48988 |
|  | *CCNA2* | ns | upregulated | ns | 90 | 2.4e-05 | 64 |
| Community 5: Metabolism, epithelium | *TRAK1* | downregulated | downregulated | upregulated | 188 | 1.5e-11 | 47766 |
|  | *VSIG2* | ns | downregulated | upregulated | 155 | 3.3e-11 | 31027 |
|  | *PPARG* | ns | downregulated | upregulated | 145 | 8.7e-12 | 11425 |
|  | *SLC44A3* | downregulated | downregulated | upregulated | 126 | 3.1e-11 | 29708 |
|  | *CYB5A* | ns | downregulated | upregulated | 124 | 8.4e-12 | 28935 |
| Community 6: Squamous identity | *SERPINB5* | downregulated | upregulated | ns | 31 | 1.9e-06 | 36922 |
|  | *SEMA4B* | downregulated | ns | ns | 29 | 2.4e-07 | 190034 |
|  | *S100A2* | downregulated | upregulated | ns | 27 | 2.5e-05 | 229365 |
|  | *CD44* | ns | upregulated | downregulated | 26 | 0.0056 | 378299 |
|  | *GJB5* | downregulated | upregulated | ns | 24 | 6.2e-07 | 26760 |
|  | *LAMB3* | downregulated | upregulated | ns | 24 | 2.4e-05 | 144602 |
| Community 7: Defense | *IFITM1* | ns | upregulated | downregulated | 211 | 0.51 | 6190 |
|  | *PSMB9* | ns | upregulated | downregulated | 208 | 0.51 | 24343 |
|  | *HLA-F* | ns | upregulated | downregulated | 206 | 0.51 | 7797 |
|  | *CXCL10* | ns | upregulated | downregulated | 159 | 0.41 | 867 |
|  | *GBP1* | ns | upregulated | downregulated | 159 | 0.39 | 1751 |

Table 28: Biological process gene ontology (GO) term enrichment in communities of co-expressed genes found to be differentially regulated in bladder cancer clusters in at least 10 bulk cancer transcriptome cohorts. Communities of the co-expressed differentially regulated genes were identified by Leiden algorithm. Biological process GO enrichment for the community members was performed by GOANA algorithm. Enrichment of a GO term in a community as compared with the entire genome was measured by odds ratio. The enrichment p values were adjusted for multiple testing with the false discovery rate (FDR) method. Significantly enriched GO terms were identified by pFDR < 0.05, OR >= 1.44, and at least 10 genes assigned to a term. The table is available as a supplementary Excel file.

|  |
| --- |
|  |

Table 29: Assignment of proteins found to be differentially regulated between bladder cancer clusters in bulk cancer proteome cohorts to communities in a protein co-expression network analysis. Co-expression networks of the differentially regulated proteins were created in the Groeneveld 2024, Dressler 2024, and Stroggilos cohorts. The network edges were formed by pairs of proteins, whose log2 expression levels correlated with Spearman's rho of at least 0.5. Communities of co-expressed proteins were identified by Leiden algorithm. The communities were named by their key biological features revealed by a biological process gene ontology term enrichment analysis. Network vertex importance was measured by degree (number of correlations with rho >= 0.5), hub score (overall correlation strength), and betweenness (number of the shortest paths between protein pairs that pass through a protein). Assignment of the differentially regulated proteins to the communities is summarized along with regulation status in bladder cancer clusters and the vertex importance metrics. Top 5 proteins of each community with the largest degree values are presented. The entire table is available as a supplementary Excel file.

| **Cohort** | **Community name** | **Protein symbol** | **Regulation in cluster #1** | **Regulation in cluster #2** | **Regulation in cluster #3** | **Degree** | **Hub score** | **Betweenness** |
| --- | --- | --- | --- | --- | --- | --- | --- | --- |
| Dressler 2024 | Community 1: Transcription | LBP | upregulated | ns | downregulated | 78 | 0.13 | 2992 |
|  |  | THEMIS2 | upregulated | ns | downregulated | 38 | 0.032 | 3372 |
|  |  | RAB31 | upregulated | ns | downregulated | 35 | 0.015 | 2585 |
|  |  | CUTA | upregulated | ns | downregulated | 24 | 0.0099 | 2032 |
|  |  | SPARCL1 | upregulated | ns | downregulated | 18 | 0.0048 | 1442 |
|  | Community 2: ECM, development | TLN1 | upregulated | upregulated | downregulated | 429 | 0.97 | 3278 |
|  |  | FLNA | upregulated | ns | downregulated | 398 | 1 | 2543 |
|  |  | LRP1 | upregulated | upregulated | downregulated | 381 | 0.93 | 2047 |
|  |  | ILK | upregulated | ns | downregulated | 346 | 0.87 | 3268 |
|  |  | PALLD | upregulated | ns | downregulated | 334 | 0.88 | 2306 |
|  |  | PDLIM7 | upregulated | ns | downregulated | 334 | 0.89 | 3888 |
|  | Community 3: Immunity | VIM | upregulated | upregulated | downregulated | 433 | 0.99 | 3126 |
|  |  | ANXA6 | upregulated | upregulated | downregulated | 425 | 0.96 | 2876 |
|  |  | MSN | upregulated | upregulated | downregulated | 329 | 0.66 | 6328 |
|  |  | GLIPR2 | upregulated | upregulated | downregulated | 328 | 0.77 | 1793 |
|  |  | KCTD12 | upregulated | upregulated | downregulated | 323 | 0.73 | 4059 |
|  | Community 4: Glycolysis, signaling | EMILIN1 | upregulated | ns | downregulated | 386 | 0.98 | 1500 |
|  |  | DPYSL2 | upregulated | ns | downregulated | 377 | 0.94 | 2027 |
|  |  | C1R | upregulated | upregulated | downregulated | 354 | 0.83 | 2576 |
|  |  | COL6A3 | upregulated | ns | downregulated | 353 | 0.94 | 1639 |
|  |  | C1S | upregulated | upregulated | downregulated | 352 | 0.85 | 2080 |
|  | Community 5: Metabolism, epithelium | SSH3 | downregulated | downregulated | upregulated | 96 | 2.1e-16 | 1412 |
|  |  | VSIG2 | downregulated | downregulated | upregulated | 91 | 1.8e-16 | 1615 |
|  |  | SPA17 | downregulated | downregulated | upregulated | 78 | 1.1e-16 | 1027 |
|  |  | IDH1 | downregulated | downregulated | upregulated | 77 | 1.1e-16 | 1721 |
|  |  | ATP8B1 | downregulated | downregulated | upregulated | 75 | 1.1e-16 | 1106 |
|  |  | SNCG | downregulated | downregulated | upregulated | 75 | 3.5e-17 | 939 |
| Groeneveld 2024 | Community 1: RNA, protein | G3BP1 | downregulated | upregulated | ns | 132 | 0.0029 | 3854 |
|  |  | UTP3 | downregulated | upregulated | ns | 129 | 0.004 | 12599 |
|  |  | UTP14A | downregulated | upregulated | ns | 128 | 0.0028 | 11628 |
|  |  | SON | downregulated | ns | upregulated | 122 | 0.00095 | 7306 |
|  |  | PFDN4 | downregulated | ns | upregulated | 120 | 0.00021 | 2466 |
|  | Community 2: Development, signaling | CORO1C | upregulated | upregulated | downregulated | 308 | 0.99 | 1657 |
|  |  | CLIC4 | upregulated | ns | downregulated | 284 | 0.99 | 890 |
|  |  | LGALS1 | ns | upregulated | downregulated | 283 | 0.96 | 1594 |
|  |  | PCOLCE | upregulated | ns | downregulated | 280 | 0.99 | 1426 |
|  |  | GLIPR2 | upregulated | ns | downregulated | 274 | 0.94 | 3382 |
|  | Community 3: Metabolism, epithelium | ADIRF | ns | downregulated | upregulated | 265 | 0.00098 | 3252 |
|  |  | SSH3 | ns | downregulated | upregulated | 259 | 0.0011 | 2928 |
|  |  | COMT | ns | downregulated | upregulated | 257 | 0.00072 | 2634 |
|  |  | SNCG | ns | downregulated | upregulated | 248 | 0.00093 | 1510 |
|  |  | CAB39L | ns | downregulated | upregulated | 245 | 0.00089 | 2766 |
|  | Community 4: Cell cycle | EEF2 | ns | upregulated | downregulated | 168 | 0.08 | 5066 |
|  |  | GMPS | ns | upregulated | downregulated | 151 | 0.049 | 1868 |
|  |  | POLD1 | ns | upregulated | downregulated | 148 | 0.069 | 1708 |
|  |  | PES1 | ns | upregulated | ns | 143 | 0.015 | 3552 |
|  |  | GTPBP4 | ns | upregulated | ns | 142 | 0.018 | 1766 |
|  | Community 5: Immunity | MSN | ns | upregulated | downregulated | 321 | 0.99 | 4492 |
|  |  | AP2M1 | upregulated | upregulated | downregulated | 302 | 0.92 | 4662 |
|  |  | GNB4 | upregulated | ns | downregulated | 289 | 0.92 | 2003 |
|  |  | RHOG | ns | ns | downregulated | 289 | 0.89 | 686 |
|  |  | CD14 | ns | upregulated | downregulated | 286 | 0.9 | 748 |
| Stroggilos 2020 | Community 1: Epithelium, squamous | SSH3 | downregulated | downregulated | upregulated | 56 | 8.6e-11 | 3047 |
|  |  | KRT19 | downregulated | downregulated | upregulated | 52 | 9.2e-11 | 995 |
|  |  | CAPN1 | downregulated | downregulated | upregulated | 51 | 6.6e-11 | 3156 |
|  |  | CAST | downregulated | ns | upregulated | 48 | 8.3e-11 | 1156 |
|  |  | PKM | downregulated | ns | upregulated | 40 | 7.1e-11 | 4383 |
|  |  | PRXL2A | downregulated | ns | upregulated | 40 | 6.9e-11 | 2416 |
|  |  | KRT7 | ns | ns | upregulated | 40 | 7.1e-11 | 1087 |
|  | Community 2: ECM, development | COL6A3 | upregulated | ns | downregulated | 68 | 1 | 818 |
|  |  | FLNA | upregulated | ns | downregulated | 66 | 0.96 | 3465 |
|  |  | COL14A1 | upregulated | ns | downregulated | 66 | 1 | 725 |
|  |  | VIM | upregulated | ns | downregulated | 64 | 0.99 | 3442 |
|  |  | COL6A2 | upregulated | ns | downregulated | 63 | 0.97 | 101 |
|  | Community 3: Metabolism | GOT2 | downregulated | ns | upregulated | 15 | 3.3e-12 | 2425 |
|  |  | VDAC1 | ns | ns | upregulated | 15 | 1.7e-10 | 3020 |
|  |  | ACO2 | ns | downregulated | upregulated | 14 | 4.1e-12 | 1508 |
|  |  | ETFB | ns | ns | upregulated | 10 | 5e-12 | 465 |
|  |  | ATP5F1D | ns | ns | upregulated | 10 | 4.1e-11 | 4054 |
|  | Community 4: RNA | HNRNPK | downregulated | ns | upregulated | 25 | 3.8e-07 | 28816 |
|  |  | DAZAP1 | downregulated | ns | upregulated | 21 | 1e-08 | 6071 |
|  |  | RPL35A | downregulated | ns | upregulated | 21 | 2.1e-08 | 18663 |
|  |  | HSPA8 | downregulated | ns | upregulated | 20 | 4.5e-09 | 6390 |
|  |  | CFL1 | downregulated | ns | upregulated | 19 | 3.8e-09 | 4255 |
|  | Community 5: Transcription, translation | EEF1G | ns | upregulated | upregulated | 35 | 0.00024 | 41917 |
|  |  | PCNA | ns | upregulated | ns | 30 | 9.5e-05 | 9992 |
|  |  | HSP90B1 | ns | upregulated | downregulated | 27 | 0.0029 | 15715 |
|  |  | DEK | ns | upregulated | downregulated | 19 | 0.00016 | 2848 |
|  |  | NASP | ns | upregulated | downregulated | 18 | 8e-05 | 1896 |
|  | Community 6: Immunity | SAMHD1 | upregulated | upregulated | downregulated | 28 | 0.14 | 20835 |
|  |  | TAP2 | ns | upregulated | downregulated | 24 | 0.0037 | 29070 |
|  |  | STAT1 | ns | upregulated | downregulated | 23 | 0.0044 | 7355 |
|  |  | LYZ | ns | upregulated | downregulated | 22 | 0.024 | 1763 |
|  |  | LCP1 | upregulated | upregulated | downregulated | 21 | 0.14 | 13701 |

Table 30: Biological process gene ontology (GO) term enrichment in communities of co-expressed proteins found to be differentially regulated in bladder cancer clusters of bulk cancer proteome cohorts. Communities of the co-expressed differentially regulated genes were identified by Leiden algorithm. Biological process GO enrichment for the community members was performed by GOANA algorithm. Enrichment of a GO term in a community as compared with the entire genome was measured by odds ratio. The enrichment p values were adjusted for multiple testing with the false discovery rate (FDR) method. Significantly enriched GO terms were identified by pFDR < 0.05, OR >= 1.44, and at least five genes assigned to a term. The table is available as a supplementary Excel file.

|  |
| --- |
|  |

Table 31: Gene set variation analysis of Reactome Pathway gene signatures in bladder cancer clusters of bulk cancer transcriptomes. Statistical significance of differences in ssGSEA scores (single sample gene set enrichment score) of the signatures between bladder cancer clusters was assessed by one-way ANOVA with eta-square effect size statistic. Statistical significance of the difference between ssGSEA score mean in a cluster and the respective ssGSEA score average in the cohort was determined by one sample T test. P values were corrected for multiple testing with the false discovery rate (FDR) method. Significantly regulated gene signatures were considered for p(ANOVA) < 0.05, eta-square >= 0.06, and p(T test) < 0.05. The analysis results are presented for gene signatures found to be significantly regulated in at least 10 bulk cancer transcriptome cohorts. The table is available as a supplementary Excel file.

|  |
| --- |
|  |

Table 32: Assignment of differentially regulated Reactome Pathway gene signatures in bladder cancer clusters of bulk cancer transcriptomes to communities of co-regulated features in a network analysis. Co-expression networks of Reactome Pathway gene signatures found to be differentially regulated in bladder cancer clusters in at least 10 transcriptome cohorts were constructed based on Jaccard distances J that quantify overlapping gene members. The network edges were formed by gene signatures with pairwise J of at least 0.3. Communities of the network were identified by Leiden algorithm and the communities were named after their key biological processes. The table is available as a supplementary Excel file.

|  |
| --- |
|  |

Table 33: Protein set variation analysis of Reactome Pathway protein signatures in bladder cancer clusters of bulk cancer proteomes. Statistical significance of differences in ssGSEA scores (single sample gene set enrichment score) of the signatures between bladder cancer clusters was assessed by one-way ANOVA with eta-square effect size statistic. Statistical significance of the difference between ssGSEA score mean in a cluster and the respective ssGSEA score average in the cohort was determined by one sample T test. P values were corrected for multiple testing with the false discovery rate (FDR) method. Significantly regulated signatures were considered for p(ANOVA) < 0.05, eta-square >= 0.06, and p(T test) < 0.05. The analysis results are presented for signatures found to be significantly regulated in at least two bulk cancer transcriptome cohorts. The table is available as a supplementary Excel file.

|  |
| --- |
|  |

Table 34: Assignment of differentially regulated Reactome Pathway protein signatures in bladder cancer clusters of bulk cancer proteomes to communities of co-regulated features in a network analysis. Co-expression networks of Reactome Pathway signatures found to be differentially regulated in bladder cancer clusters in at least two proteome cohorts were constructed based on Jaccard distances J that quantify overlapping gene members. The network edges were formed by gene signatures with pairwise J of at least 0.3. Communities of the network were identified by Leiden algorithm and the communities were named after their key biological processes. The table is available as a supplementary Excel file.

|  |
| --- |
|  |

Table 35: Infiltration of non-malignant cells in bladder cancer clusters resolved by immunedeconvolution. Fractions of non-malignant cells in cancer samples were predicted by quanTIseq and xCell algorithms fed with whole-transcriptome expression data. Differences in infiltration levels between bladder cancer clusters were assessed by Kruskal-Wallis test with eta-square effect size metric. P values were corrected for multiple testing with the false discovery rate method. Median cell fractions with interquartile ranges and ranges are listed. The table is provided as a supplementary Excel file.

|  |
| --- |
|  |

Table 36: Meta-analysis of differential expression of matrisome genes in bladder cancer clusters of bulk cancer transcriptomes. Meta-estimates of log2 fold-regulation of differential expression were computed for matrisome genes found to be differentially regulated between at least 10 cohorts with the DerSimonian-Lair algorithm. Meta-estimates with 95% confidence intervals (CI) and p values corrected for multiple testing with the false discovery rate method are listed. The results for the top 3 up- and downregulated genes in each matrisome category are shown. The entire table is available as a supplementary Excel file.

| **Matrisome category** | **Gene symbol** | **Bladder cancer cluster** | **Regulation status** | **log2 fold-regulation vs cohort mean, meta-estimate, 95% CI** | **p value** |
| --- | --- | --- | --- | --- | --- |
| Collagens | *COL6A3* | #1 | upregulated | 0.76 [95%CI: 0.58 to 0.93] | 1.5e-16 |
|  | *COL3A1* | #1 | upregulated | 0.7 [95%CI: 0.52 to 0.88] | 6.2e-14 |
|  | *COL1A2* | #1 | upregulated | 0.66 [95%CI: 0.48 to 0.84] | 1.6e-12 |
|  | *COL6A3* | #3 | downregulated | -0.68 [95%CI: -0.81 to -0.56] | 1.5e-26 |
|  | *COL3A1* | #3 | downregulated | -0.67 [95%CI: -0.8 to -0.54] | 7.2e-24 |
|  | *COL1A2* | #3 | downregulated | -0.64 [95%CI: -0.77 to -0.5] | 2e-19 |
| ECM Glycoproteins | *SPP1* | #2 | upregulated | 0.94 [95%CI: 0.74 to 1.1] | 3.6e-19 |
|  | *LAMC2* | #2 | upregulated | 0.91 [95%CI: 0.73 to 1.1] | 7e-24 |
|  | *MGP* | #1 | upregulated | 0.89 [95%CI: 0.73 to 1.1] | 5.8e-24 |
|  | *POSTN* | #3 | downregulated | -0.8 [95%CI: -0.96 to -0.64] | 4.6e-22 |
|  | *AEBP1* | #3 | downregulated | -0.64 [95%CI: -0.77 to -0.52] | 1.5e-22 |
|  | *TNC* | #3 | downregulated | -0.64 [95%CI: -0.75 to -0.53] | 1.2e-27 |
| Proteoglycans | *DCN* | #1 | upregulated | 0.82 [95%CI: 0.65 to 0.98] | 4.9e-20 |
|  | *LUM* | #1 | upregulated | 0.76 [95%CI: 0.58 to 0.94] | 4.4e-15 |
|  | *SRGN* | #2 | upregulated | 0.63 [95%CI: 0.49 to 0.76] | 8.6e-19 |
|  | *LUM* | #3 | downregulated | -0.6 [95%CI: -0.74 to -0.47] | 3.6e-18 |
|  | *DCN* | #3 | downregulated | -0.59 [95%CI: -0.71 to -0.47] | 7.4e-22 |
|  | *SRGN* | #3 | downregulated | -0.59 [95%CI: -0.69 to -0.49] | 3.2e-31 |
| ECM Regulators | *PI3* | #2 | upregulated | 1.8 [95%CI: 1.4 to 2.2] | 3.6e-20 |
|  | *SERPINB3* | #2 | upregulated | 1.7 [95%CI: 1.3 to 2.1] | 1.4e-16 |
|  | *SERPINB4* | #2 | upregulated | 1.4 [95%CI: 1 to 1.7] | 5.5e-16 |
|  | *CTSE* | #2 | downregulated | -0.77 [95%CI: -0.97 to -0.57] | 1.5e-13 |
|  | *SULF1* | #3 | downregulated | -0.77 [95%CI: -0.91 to -0.63] | 2.1e-25 |
|  | *SULF2* | #3 | downregulated | -0.59 [95%CI: -0.69 to -0.5] | 6e-31 |
| Secreted Factors | *S100A8* | #2 | upregulated | 1.8 [95%CI: 1.5 to 2.2] | 3.1e-19 |
|  | *S100A7* | #2 | upregulated | 1.5 [95%CI: 1.2 to 1.8] | 1e-18 |
|  | *S100A9* | #2 | upregulated | 1.4 [95%CI: 1.1 to 1.7] | 6.3e-21 |
|  | *SCUBE2* | #2 | downregulated | -0.88 [95%CI: -0.99 to -0.76] | 3e-48 |
|  | *SFRP2* | #3 | downregulated | -0.81 [95%CI: -0.95 to -0.67] | 1.2e-27 |
|  | *S100A2* | #1 | downregulated | -0.75 [95%CI: -0.92 to -0.59] | 2e-17 |
| ECM-affiliated Proteins | *ANXA1* | #2 | upregulated | 0.98 [95%CI: 0.75 to 1.2] | 2.8e-16 |
|  | *LGALS7* | #2 | upregulated | 0.78 [95%CI: 0.55 to 1] | 5.2e-11 |
|  | *C1QB* | #2 | upregulated | 0.69 [95%CI: 0.56 to 0.82] | 9.1e-23 |
|  | *C1QC* | #2 | upregulated | 0.69 [95%CI: 0.56 to 0.83] | 4.4e-22 |
|  | *GREM1* | #1 | upregulated | 0.69 [95%CI: 0.49 to 0.89] | 5.8e-11 |
|  | *FREM2* | #2 | downregulated | -0.76 [95%CI: -0.9 to -0.62] | 3.3e-25 |
|  | *SEMA5A* | #2 | downregulated | -0.66 [95%CI: -0.83 to -0.49] | 6.6e-14 |
|  | *GREM1* | #3 | downregulated | -0.62 [95%CI: -0.73 to -0.52] | 1.7e-27 |

Table 37: Differential regulation of matrisome proteins in bladder cancer clusters of the Groeneveld 2024, Dressler 2024, and Stroggilos 2020 bulk cancer proteome cohorts. Significance of differential expression as compared with the cohort average was tested for log2-transformed expression values by one-way ANOVA and one-sample T test. P values were corrected for multiple testing with the false discovery rate method. log2 fold-regulation estimates with 95% confidence intervals in bladder cancer clusters as compared with the cohort averages and p values of the T test are summarized. The table is available as a supplementary Excel file.

|  |
| --- |
|  |

Table 38: Activity of transcriptional regulons in bladder cancer clusters predicted with bulk transcriptome data by univariable linear modeling with collecTRI model and decoupleR package. Statistical significance of differences in linear modeling (LM) activity scores of the collecTRI regulons was assessed by one-way ANOVA with eta-square effect size statistic. Statistical significance of the difference between LM score mean in a cluster and the respective LM score average in the cohort was determined by one sample T test. P values were corrected for multiple testing with the false discovery rate (FDR) method. Significant regulons were considered for p(ANOVA) < 0.05, eta-square >= 0.06, and p(T test) < 0.05. The analysis results are presented for regulons found to be significantly modulated in at least 10 bulk cancer transcriptome cohorts. The table is available as a supplementary Excel file.

|  |
| --- |
|  |

Table 39: Meta-analysis of activity of collecTRI transcriptional regulons in bladder cancer clusters of bulk cancer transcriptomes. Meta-estimates of differences in linear modeling (LM) scores between the cluster and cohort mean were computed for regulons found to be significantly differentially modulated in at least 10 cohorts. The meta-estimates were computed with the DerSimonian-Lair algorithm. Meta-estimates with 95% confidence intervals (CI) and p values corrected for multiple testing with the false discovery rate method are listed. The table is available as a supplementary Excel file.

|  |
| --- |
|  |

Table 40: Assignment of differentially modulated collecTRI regulons to network communities. A correlation network was constructed for regulons found to be differentially activated or inhibited in at least 10 bulk transcriptome cohorts in a pooled collective of normalized regulons' activity scores consisting of all available bulk cancer transcriptome samples. The network edges were defined for pairwise correlations between the activity scores with Spearman's rho >= 0.3. Communities of the network were identified by Leiden algorithm and the communities were named after their key biological processes. Network vertex importance was measured by degree (number of correlations with rho >= 0.3), hub score (overall correlation strength), and betweenness (number of the shortest paths between protein pairs that pass through a protein). Assignment of the regulons to the communities is summarized along with activity status in bladder cancer clusters, and vertex importance metrics. The table is available as a supplementary Excel file.

|  |
| --- |
|  |

Table 41: Activity of PROGENy signaling pathways in bladder cancer clusters predicted with bulk transcriptome data by multivariable linear modeling with decoupleR package. Statistical significance of differences in linear modeling (LM) activity scores of the signaling pathways was assessed by one-way ANOVA with eta-square effect size statistic. Statistical significance of the difference between LM score mean in a cluster and the respective LM score average in the cohort was determined by one sample T test. P values were corrected for multiple testing with the false discovery rate (FDR) method. Significant features were considered for p(ANOVA) < 0.05, eta-square >= 0.06, and p(T test) < 0.05. The analysis results are presented for pathways found to be significantly modulated in at least 10 bulk cancer transcriptome cohorts. The table is available as a supplementary Excel file.

|  |
| --- |
|  |

Table 42: Activity of collecTRI transcriptional regulons in bladder cancer clusters of the Groeneveld 2024, Dressler 2024, and Stroggilos 2020 of the bulk cancer proteome cohorts as compared with the cohort average. The regulon activity was modeled by the univariable linear regression tool of decoupleR package. The algorithm was fed with T statistics of differential protein expression in bladder cancer clusters as compared with the cohort average obtained by one-sample T test. Activity of the regulons was gauged by linear modeling score (LM score). P values were corrected for multiple testing with the false discovery rate (FDR) method. Significantly activated features were defined by pFDR < 0.05 and LM score > 0. Significantly inhibited features were considered for pFDR < 0.05 and LM score < 0. Regulons found to be significantly activated or inhibited in at least two bulk cancer proteome cohorts are listed. The table is available as a supplementary Excel file.

|  |
| --- |
|  |

Table 43: Activity of PROGENy signaling pathways in bladder cancer clusters of the Groeneveld 2024, Dressler 2024, and Stroggilos 2020 of the bulk cancer proteome cohorts as compared with the cohort average. The pathway activity was modeled by the multivariable linear regression tool of decoupleR package. The algorithm was fed with T statistics of differential protein expression in bladder cancer clusters as compared with the cohort average obtained by one-sample T test. Activity of the signaling pathways was gauged by linear modeling score (LM score). P values were corrected for multiple testing with the false discovery rate (FDR) method. Significantly activated features were defined by pFDR < 0.05 and LM score > 0. Significantly inhibited features were considered for pFDR < 0.05 and LM score < 0. Signaling pathways found to be significantly activated or inhibited in at least two bulk cancer proteome cohorts are listed. The table is available as a supplementary Excel file.

|  |
| --- |
|  |

Table 44: Activity of RECON metabolic reactions in bladder cancer clusters of bulk cancer transcriptome cohorts as compared with the respective cohort averages. Activity of metabolic reactions was modeled by a Monte Carlo simulation implemented by biggrExtra package. The algorithm was fed with log2 fold-regulation estimates of differential gene expression with their standard errors obtained by one-sample T test for all available genes. The log2 fold-regulation estimates of reaction activity with the corresponding standard errors and 95% confidence intervals were obtained by serial evaluation of the gene-reaction assignment rules of the RECON2 knowledge model for n = 3000 random draws from the simulated normal distributions of the differential gene expression in the clusters. P values were corrected for multiple testing with the false discovery rate (FDR) method. The modeling results for the significantly regulated reactions shared by at least 10 out of 19 cohorts are summarized in a supplementary Excel table.

|  |
| --- |
|  |

Table 45: Enrichment of RECON metabolic subsystems with significantly activated and inhibited metabolic reactions in bladder cancer clusters of bulk cancer transcriptome cohorts. Frequency of metabolic subsystem reaction in the sets of activated and inhibited reactions was compared with n = 10000 random draws from the entire reaction pool. Magnitude of enrichment over the entire reaction pool was quantified by odds ratio (OR). P values were corrected for multiple testing with the false discovery rate method. Significant enrichment was considered for p < 0.05 and OR of at least 1.44. Subsystems found to be significantly enriched within the sets of activated or inhibited reactions in at least 10 out of 19 analyzed cohorts. are listed. The table is available as a supplementary Excel file.

|  |
| --- |
|  |

Table 46: Activity of RECON metabolic reactions in bladder cancer clusters of the Groeneveld 2024, Dressler 2024, and, Stroggilos 2020 bulk cancer proteome cohorts as compared with the respective cohort averages. Activity of metabolic reactions was modeled by a Monte Carlo simulation implemented by biggrExtra package. The algorithm was fed with log2 fold-regulation estimates of differential protein expression with their standard errors obtained by one-sample T test for all available proteins. The log2 fold-regulation estimates of reaction activity with the corresponding standard errors and 95% confidence intervals were obtained by serial evaluation of the gene-reaction assignment rules of the RECON2 knowledge model for n = 3000 random draws from the simulated normal distributions of the differential gene expression in the clusters. P values were corrected for multiple testing with the false discovery rate (FDR) method. The modeling results for the significantly regulated reactions shared by at least two cohorts are summarized in a supplementary Excel table.

|  |
| --- |
|  |

Table 47: Enrichment of RECON metabolic subsystems with significantly activated and inhibited metabolic reactions in bladder cancer clusters of bulk cancer proteome cohorts. Frequency of metabolic subsystem reaction in the sets of activated and inhibited reactions was compared with n = 10000 random draws from the entire reaction pool. Magnitude of enrichment over the entire reaction pool was quantified by odds ratio (OR). P values were corrected for multiple testing with the false discovery rate method. Significant enrichment was considered for p < 0.05 and OR of at least 1.44. Subsystems found to be significantly enriched within the sets of activated or inhibited reactions in at least in at least two cohorts are listed. The table is available as a supplementary Excel file.

|  |
| --- |
|  |

Table 48: Differences in predicted anti-cancer drug resistance between bladder cancer clusters of bulk cancer transcriptome cohorts. Resistance to anti-cancer drugs in form of IC50 (concentration resulting in 50% growth inhibition) or AUC (area under the dose response curve) was predicted with RIDGE regularized linear models fed with whole-transcriptome ComBat-processed log2-transformed gene expression values. The RIDGE models were trained with in vitro drug screening data of epithelial cancer cells lines (GDSC, CTRP2, and PRISM experiments). Statistical significance of differences in the predicted resistance metrics between bladder cancer clusters was determined by one-way ANOVA with eta-square effect size statistic. Statistical significance of the difference between mean resistance in a cluster and the respective resistance average in the cohort was determined by one sample T test. P values were corrected for multiple testing with the false discovery rate (FDR) method. Significant differences in the predicted drug resistance were considered for p(ANOVA) < 0.05, eta-square >= 0.06, and p(T test) < 0.05. The analysis results are presented for drugs found to differ in significance between bladder cancer clusters in at least 10 out of 18 bulk cancer transcriptome cohorts. The table is available as a supplementary Excel file.

|  |
| --- |
|  |

Table 49: Meta-analysis of differences in predicted anti-cancer drug resistance in bladder cancer clusters of bulk transcriptome samples. Resistance to anti-cancer drugs was predicted for single bulk cancer samples by regularized RIDGE linear models fed with whole-transcriptome gene expression. The models were trained with data from three in vitro drug screening experiments (GDSC, CTRP2, and PRISM). Differences in the resistance between the clusters and the respective cohort means were investigated by one-way ANOVA and one-sample T test. For genes with significant differences in the predicted resistance shared by at least 10 out of 18 investigated cohorts, meta-estimates of difference in resistance metrics between the clusters and cohort means were computed with the DerSimonian-Lair algorithm. These meta-estimates are listed with the corresponding 95% confidence intervals and p values corrected for multiple testing with the false discovery rate method. The top 10 resistant and sensitive compounds in bladder cancer clusters are shown. The full table is available as a supplementary Excel file.

| **Bladder cancer cluster** | **Resistance status** | **Drug name, ID, training data set** | **Target classification** | **delta resistance Z-score versus cohort mean, 95% CI** | **p value** |
| --- | --- | --- | --- | --- | --- |
| #1 | resistant | Gefitinib, ID1010, GDSC2 | EGFR/ERBB | 0.76 [95% CI: 0.69 to 0.83] | 6.8e-99 |
|  |  | Sapitinib, ID1549, GDSC2 | EGFR/ERBB | 0.73 [95% CI: 0.68 to 0.79] | 2.4e-161 |
|  |  | Pelitinib, ID282, GDSC1 | EGFR/ERBB | 0.71 [95% CI: 0.65 to 0.76] | 7.6e-142 |
|  |  | AZD3759, ID1915, GDSC2 | EGFR/ERBB | 0.7 [95% CI: 0.62 to 0.77] | 8e-73 |
|  |  | Osimertinib, ID1919, GDSC2 | EGFR/ERBB | 0.7 [95% CI: 0.61 to 0.79] | 2.2e-56 |
|  |  | Lapatinib, ID1558, GDSC2 | EGFR/ERBB | 0.66 [95% CI: 0.6 to 0.73] | 8.3e-84 |
|  |  | Afatinib, ID1032, GDSC2 | EGFR/ERBB | 0.64 [95% CI: 0.56 to 0.72] | 2e-59 |
|  |  | Erlotinib, ID1168, GDSC2 | EGFR/ERBB | 0.64 [95% CI: 0.56 to 0.73] | 4.3e-52 |
|  |  | Ibrutinib, ID1799, GDSC2 | other GF signaling | 0.61 [95% CI: 0.53 to 0.68] | 2.8e-53 |
|  |  | Gefitinib, ID52926, CTRP2 | EGFR/ERBB | 0.61 [95% CI: 0.52 to 0.69] | 6.5e-42 |
|  | sensitive | ML210, ID609110, CTRP2 | ferroptosis | -0.62 [95% CI: -0.7 to -0.54] | 1.1e-52 |
|  |  | ML162, ID154846, CTRP2 | ferroptosis | -0.57 [95% CI: -0.64 to -0.51] | 1.3e-62 |
|  |  | 1S,3R-RSL-3, ID609060, CTRP2 | ferroptosis | -0.56 [95% CI: -0.63 to -0.5] | 4.6e-59 |
|  |  | BRD-K94991378, ID53556, CTRP2 | other | -0.48 [95% CI: -0.55 to -0.4] | 2.8e-33 |
|  |  | ABT-199, ID666541, CTRP2 | apoptosis | -0.46 [95% CI: -0.54 to -0.38] | 1.2e-30 |
|  |  | Dabrafenib, ID1373, GDSC2 | RAS/RAF/MAPK | -0.45 [95% CI: -0.52 to -0.39] | 5.9e-39 |
|  |  | BRD-K34222889, ID688888, CTRP2 | other | -0.44 [95% CI: -0.5 to -0.38] | 8.4e-53 |
|  |  | Erastin, ID63578, CTRP2 | ferroptosis | -0.44 [95% CI: -0.5 to -0.39] | 6.2e-58 |
|  |  | Eprosartan, PRISM | other | -0.44 [95% CI: -0.53 to -0.34] | 2.4e-18 |
|  |  | AZD6482, ID1066, GDSC1 | PI3K/TOR | -0.43 [95% CI: -0.49 to -0.37] | 2.3e-42 |
|  |  | TAK-715, PRISM | RAS/RAF/MAPK | -0.43 [95% CI: -0.51 to -0.36] | 1.6e-28 |
| #2 | resistant | Vorinostat, ID56554, CTRP2 | epigenetics | 0.89 [95% CI: 0.83 to 0.95] | 1.8e-174 |
|  |  | Skepinone-L, ID688799, CTRP2 | RAS/RAF/MAPK | 0.73 [95% CI: 0.65 to 0.8] | 2.1e-78 |
|  |  | 667880, ID1829, GDSC2 | other | 0.71 [95% CI: 0.65 to 0.77] | 5.3e-121 |
|  |  | Foretinib, ID628607, CTRP2 | EGFR/ERBB | 0.7 [95% CI: 0.61 to 0.79] | 8.9e-57 |
|  |  | Amuvatinib, ID293, GDSC1 | other GF signaling | 0.65 [95% CI: 0.56 to 0.74] | 1.3e-42 |
|  |  | HBX-41108, ID679843, CTRP2 | cell cycle, DNA repair | 0.6 [95% CI: 0.52 to 0.67] | 3.2e-56 |
|  |  | SR-33805, PRISM | other | 0.6 [95% CI: 0.52 to 0.69] | 1.6e-45 |
|  |  | Valdecoxib, ID32372, CTRP2 | other | 0.59 [95% CI: 0.5 to 0.68] | 6.1e-38 |
|  |  | Erdafitinib, PRISM | FGFR | 0.59 [95% CI: 0.52 to 0.66] | 5.3e-55 |
|  |  | TAF1-5496, ID1732, GDSC2 | other | 0.58 [95% CI: 0.5 to 0.67] | 7.6e-41 |
|  | sensitive | Dasatinib, ID52882, CTRP2 | other GF signaling | -1 [95% CI: -1.1 to -0.93] | 5.4e-135 |
|  |  | 993-D2, ID1360, GDSC1 | RAS/RAF/MAPK | -0.89 [95% CI: -1 to -0.77] | 2.9e-50 |
|  |  | Dasatinib, ID1079, GDSC2 | other GF signaling | -0.88 [95% CI: -0.99 to -0.78] | 1.6e-57 |
|  |  | WH-4-023, ID56, GDSC1 | other GF signaling | -0.87 [95% CI: -0.94 to -0.8] | 1.1e-137 |
|  |  | C-75, ID435, GDSC1 | apoptosis | -0.86 [95% CI: -0.92 to -0.79] | 3.2e-142 |
|  |  | Cisplatin, ID1496, GDSC1 | DNA | -0.79 [95% CI: -0.88 to -0.7] | 6.4e-68 |
|  |  | Vandetanib, ID349002, CTRP2 | EGFR/ERBB | -0.79 [95% CI: -0.9 to -0.68] | 5.9e-44 |
|  |  | PARP-9482, ID1460, GDSC1 | PARP | -0.78 [95% CI: -0.86 to -0.69] | 9.3e-67 |
|  |  | LGX818, PRISM | RAS/RAF/MAPK | -0.78 [95% CI: -0.85 to -0.71] | 1.3e-109 |
|  |  | Bleomycin (10 uM), ID1392, GDSC1 | DNA | -0.77 [95% CI: -0.86 to -0.69] | 9.9e-66 |
|  |  | IAP-7638, ID1429, GDSC1 | apoptosis | -0.77 [95% CI: -0.86 to -0.67] | 2.6e-58 |
|  |  | PARP-9495, ID1458, GDSC1 | PARP | -0.77 [95% CI: -0.85 to -0.69] | 2e-85 |
| #3 | resistant | Dasatinib, ID52882, CTRP2 | other GF signaling | 0.65 [95% CI: 0.6 to 0.71] | 7.7e-103 |
|  |  | WH-4-023, ID56, GDSC1 | other GF signaling | 0.59 [95% CI: 0.52 to 0.65] | 2.5e-68 |
|  |  | Dasatinib, ID1079, GDSC2 | other GF signaling | 0.57 [95% CI: 0.49 to 0.64] | 2.4e-50 |
|  |  | PARP-9482, ID1460, GDSC1 | PARP | 0.56 [95% CI: 0.49 to 0.63] | 1.6e-55 |
|  |  | CHIR-99021, ID1241, GDSC1 | PI3K/TOR | 0.55 [95% CI: 0.48 to 0.61] | 1.1e-57 |
|  |  | PARP-9495, ID1458, GDSC1 | PARP | 0.55 [95% CI: 0.48 to 0.62] | 2.3e-49 |
|  |  | LGX818, PRISM | RAS/RAF/MAPK | 0.55 [95% CI: 0.48 to 0.61] | 9.1e-59 |
|  |  | CHIR-99021, ID154, GDSC1 | PI3K/TOR | 0.54 [95% CI: 0.46 to 0.62] | 1e-40 |
|  |  | Cisplatin, ID1496, GDSC1 | DNA | 0.54 [95% CI: 0.48 to 0.61] | 6.3e-58 |
|  |  | Fulvestrant, PRISM | other | 0.54 [95% CI: 0.48 to 0.61] | 1.1e-61 |
|  | sensitive | Meloxicam, PRISM | other | -0.56 [95% CI: -0.62 to -0.51] | 6.9e-88 |
|  |  | 150412, ID1836, GDSC2 | other | -0.55 [95% CI: -0.63 to -0.47] | 2.7e-43 |
|  |  | Benzethonium, PRISM | other | -0.55 [95% CI: -0.6 to -0.5] | 3.7e-99 |
|  |  | Austocystin D, ID416775, CTRP2 | DNA | -0.53 [95% CI: -0.6 to -0.46] | 1.2e-49 |
|  |  | AZD8931, PRISM | EGFR/ERBB | -0.53 [95% CI: -0.59 to -0.47] | 3.3e-69 |
|  |  | LGK974, ID1598, GDSC2 | other | -0.49 [95% CI: -0.55 to -0.42] | 2.1e-46 |
|  |  | TAF1-5496, ID1732, GDSC2 | other | -0.48 [95% CI: -0.54 to -0.41] | 3.6e-51 |
|  |  | Barasertib, ID601923, CTRP2 | cell cycle, DNA repair | -0.48 [95% CI: -0.54 to -0.41] | 2.5e-42 |
|  |  | Afatinib, PRISM | EGFR/ERBB | -0.47 [95% CI: -0.53 to -0.41] | 6.2e-54 |
|  |  | Afatinib, ID1032, GDSC2 | EGFR/ERBB | -0.46 [95% CI: -0.52 to -0.39] | 2e-41 |
|  |  | PF-00299804, ID363, GDSC1 | EGFR/ERBB | -0.46 [95% CI: -0.51 to -0.41] | 7.3e-84 |

Table 50: Overall survival of TCGA BLCA and BCAN cohort patents in bladder cancer clusters stratified by systemic chemotherapy status. Statistical significance of differences in overall survival between patients with and without systemic chemotherapy within each cluster were assessed by Peto-Peto test. P values were corrected for multiple comparisons with the false discovery rate (FDR) method. Survival times with 95% confidence intervals for quantiles of surviving fractions, raw and FDR-adjusted significance are presented.

| **Cohort** | **Bladder cancer cluster** | **Cytotoxic therapy** | **Deaths, N** | **Total patients, N** | **Survival percentile** | **Survival time, days** | **Raw significance** | **FDR significance** |
| --- | --- | --- | --- | --- | --- | --- | --- | --- |
| BCAN | #1 | no | 3 | 10 | 25 | 1300 [95%: 570 to NA | ns (p = 0.41) | ns (p = 0.49) |
|  |  |  |  |  | 50 |  |  |  |
|  |  |  |  |  | 75 |  |  |  |
|  |  | yes | 25 | 47 | 25 | 1000 [95%: 730 to 1300 |  |  |
|  |  |  |  |  | 50 | 1600 [95%: 1200 to NA |  |  |
|  |  |  |  |  | 75 | 2900 [95%: 2400 to NA |  |  |
|  | #2 | no | 8 | 13 | 25 | 190 [95%: 120 to NA | ns (p = 0.16) | ns (p = 0.32) |
|  |  |  |  |  | 50 | 790 [95%: 190 to NA |  |  |
|  |  |  |  |  | 75 | 2500 [95%: 790 to NA |  |  |
|  |  | yes | 13 | 27 | 25 | 630 [95%: 410 to 1100 |  |  |
|  |  |  |  |  | 50 | 1100 [95%: 700 to NA |  |  |
|  |  |  |  |  | 75 |  |  |  |
|  | #3 | no | 6 | 13 | 25 | 600 [95%: 160 to NA | ns (p = 0.38) | ns (p = 0.49) |
|  |  |  |  |  | 50 |  |  |  |
|  |  |  |  |  | 75 |  |  |  |
|  |  | yes | 20 | 41 | 25 | 810 [95%: 470 to 1600 |  |  |
|  |  |  |  |  | 50 | 1600 [95%: 970 to NA |  |  |
|  |  |  |  |  | 75 | 2000 [95%: 1700 to NA |  |  |
| TCGA BLCA | #1 | no | 45 | 74 | 25 | 250 [95%: 150 to 490 | p = 0.0039 | p = 0.012 |
|  |  |  |  |  | 50 | 590 [95%: 530 to 1000 |  |  |
|  |  |  |  |  | 75 | 1100 [95%: 860 to NA |  |  |
|  |  | yes | 23 | 45 | 25 | 600 [95%: 490 to 740 |  |  |
|  |  |  |  |  | 50 | 860 [95%: 710 to NA |  |  |
|  |  |  |  |  | 75 |  |  |  |
|  | #2 | no | 47 | 84 | 25 | 300 [95%: 250 to 410 | p = 0.0028 | p = 0.012 |
|  |  |  |  |  | 50 | 620 [95%: 420 to 1000 |  |  |
|  |  |  |  |  | 75 | 1900 [95%: 1000 to NA |  |  |
|  |  | yes | 9 | 33 | 25 | 510 [95%: 320 to NA |  |  |
|  |  |  |  |  | 50 |  |  |  |
|  |  |  |  |  | 75 |  |  |  |
|  | #3 | no | 43 | 137 | 25 | 550 [95%: 410 to 940 | ns (p = 0.79) | ns (p = 0.79) |
|  |  |  |  |  | 50 | 2600 [95%: 1300 to NA |  |  |
|  |  |  |  |  | 75 | 3200 [95%: 3000 to NA |  |  |
|  |  | yes | 11 | 33 | 25 | 610 [95%: 470 to NA |  |  |
|  |  |  |  |  | 50 | 2000 [95%: 620 to NA |  |  |
|  |  |  |  |  | 75 |  |  |  |

Table 51: Assignment of DepMap urothelial cancer cell lines to bladder cancer clusters. The assignment was accomplished by an Elastic Net classifiertrained in the TCGA BLCA transcriptomic collective with ComBat-processed log2-transformed bulk cancer expression levels of genes present in a median of at least 5 TPM in the training data set and the DepMap urothelial cancer cell lines. Probabilities of assignment of the cell lines to particular bladder cancer clusters, the final assignment, derived from nodal voting and correlation with centroids of the clusters in the TCGA BLCA cohort are listed. Reliable assignment was considered for cluster assignment probabilities >= 0.5.

| **Bladder cancer cluster** | **Reliable assignment** | **DepMap ID** | **Cell line name** | **Probability, cluster #1** | **Probability, cluster #2** | **Probability, cluster #3** | **Correlation with centroid, cluster #1** | **Correlation with centroid, cluster #2** | **Correlation with centroid, cluster #3** |
| --- | --- | --- | --- | --- | --- | --- | --- | --- | --- |
| #1 | yes | ACH-000011 | 253J | 0.57 | 0.12 | 0.31 | 0.66 | 0.53 | 0.59 |
|  |  | ACH-000720 | TCCSUP | 0.84 | 0.12 | 0.036 | 0.83 | 0.64 | 0.58 |
|  |  | ACH-000741 | UBLC1 | 0.85 | 0.056 | 0.095 | 0.77 | 0.59 | 0.52 |
|  |  | ACH-000026 | 253JBV | 0.73 | 0.093 | 0.18 | 0.68 | 0.56 | 0.61 |
|  |  | ACH-000018 | T24 | 0.58 | 0.35 | 0.071 | 0.77 | 0.74 | 0.62 |
|  |  | ACH-000522 | UMUC3 | 0.83 | 0.11 | 0.068 | 0.72 | 0.54 | 0.53 |
|  |  | ACH-000753 | JMSU1 | 0.85 | 0.1 | 0.045 | 0.81 | 0.66 | 0.64 |
|  |  | ACH-000896 | 647V | 0.53 | 0.27 | 0.2 | 0.75 | 0.77 | 0.65 |
|  |  | ACH-000973 | 639V | 0.78 | 0.13 | 0.091 | 0.73 | 0.57 | 0.58 |
|  |  | ACH-000396 | J82 | 0.63 | 0.33 | 0.039 | 0.77 | 0.68 | 0.55 |
|  |  | ACH-000566 | SW1710 | 0.98 | 0.014 | 0.0071 | 0.79 | 0.58 | 0.5 |
|  |  | ACH-000127 | SLR20 | 0.62 | 0.086 | 0.29 | 0.8 | 0.62 | 0.69 |
|  |  | ACH-001413 | UMUC11 | 0.72 | 0.24 | 0.046 | 0.77 | 0.65 | 0.56 |
|  | no | ACH-001412 | UMUC10 | 0.41 | 0.33 | 0.26 | 0.74 | 0.76 | 0.73 |
| #2 | yes | ACH-000545 | VMCUB1 | 0.041 | 0.72 | 0.24 | 0.56 | 0.74 | 0.65 |
|  |  | ACH-000839 | SCABER | 0.042 | 0.9 | 0.058 | 0.65 | 0.87 | 0.59 |
|  |  | ACH-000905 | 5637 | 0.17 | 0.77 | 0.06 | 0.69 | 0.81 | 0.59 |
|  |  | ACH-000593 | BC3C | 0.085 | 0.87 | 0.044 | 0.72 | 0.77 | 0.64 |
|  |  | ACH-000486 | KU1919 | 0.15 | 0.53 | 0.32 | 0.69 | 0.76 | 0.72 |
|  |  | ACH-001409 | UMUC16 | 0.046 | 0.82 | 0.13 | 0.66 | 0.83 | 0.7 |
|  |  | ACH-001407 | UMUC13 | 0.39 | 0.52 | 0.087 | 0.68 | 0.61 | 0.49 |
|  | no | ACH-000142 | CAL29 | 0.22 | 0.45 | 0.33 | 0.75 | 0.79 | 0.72 |
|  |  | ACH-000802 | BFTC905 | 0.08 | 0.47 | 0.45 | 0.62 | 0.77 | 0.74 |
| #3 | yes | ACH-000384 | SW780 | 0.053 | 0.023 | 0.92 | 0.65 | 0.55 | 0.9 |
|  |  | ACH-000724 | HT1376 | 0.048 | 0.13 | 0.83 | 0.61 | 0.65 | 0.76 |
|  |  | ACH-000862 | KMBC2 | 0.091 | 0.044 | 0.87 | 0.68 | 0.64 | 0.82 |
|  |  | ACH-000834 | UMUC1 | 0.11 | 0.15 | 0.74 | 0.71 | 0.73 | 0.79 |
|  |  | ACH-000242 | RT4 | 0.0066 | 0.001 | 0.99 | 0.64 | 0.52 | 0.9 |
|  |  | ACH-000473 | RT112 | 0.042 | 0.1 | 0.86 | 0.63 | 0.65 | 0.86 |
|  |  | ACH-001414 | UMUC6 | 0.036 | 0.16 | 0.81 | 0.59 | 0.69 | 0.74 |
|  |  | ACH-001408 | UMUC14 | 0.12 | 0.033 | 0.84 | 0.71 | 0.63 | 0.85 |
|  |  | ACH-001415 | UMUC7 | 0.059 | 0.0078 | 0.93 | 0.71 | 0.57 | 0.87 |
|  |  | ACH-001183 | RT11284 | 0.022 | 0.049 | 0.93 | 0.56 | 0.59 | 0.86 |
|  |  | ACH-001411 | UMUC5 | 0.027 | 0.24 | 0.74 | 0.61 | 0.74 | 0.79 |
|  |  | ACH-001416 | UMUC9 | 0.037 | 0.0056 | 0.96 | 0.63 | 0.46 | 0.84 |
|  |  | ACH-001410 | UMUC4 | 0.078 | 0.019 | 0.9 | 0.56 | 0.41 | 0.73 |
|  | no | ACH-000547 | HT1197 | 0.12 | 0.4 | 0.48 | 0.63 | 0.67 | 0.64 |

Table 52: Genes significantly differentially regulated in bladder cancer clusters of DepMap urothelial cancer cell lines. Differences in log2-transformed expression levels between bladder cancer clusters were assessed by one-way ANOVA with eta-square effect size statistic. Differences in log2-transformed expression between a bladder cancer cluster and the respective cohort average were investigated by one-sample T test. P values were corrected for multiple comparisons with the false discovery rate method (FDR). Significantly differentially regulated features were defined by pFDR(ANOVA) < 0.05, eta-square >= 0.06, and pFDR(T test) < 0.05. Results of statistical hypothesis testing and log2 fold-regulation estimates of difference in expression levels between the clusters and cohort averages with 95% confidence intervals are listed. The table is available as a supplementary Excel file.

|  |
| --- |
|  |

Table 53: Assignment of genes found to be differentially regulated between bladder cancer clusters of DepMap urothelial cancer cell lines to communities in a gene co-expression network analysis. A co-expression network was created for pairs of the genes whose expression levels correlated with Spearman rho of at least 0.5. Communities of the network were identified by Leiden algorithm. The communities were named by their key biological features revealed by a biological process gene ontology term enrichment analysis. Network vertex importance was measured by degree (number of correlations with rho >= 0.5), hub score (overall correlation strength), and betweenness (number of the shortest paths between gene pairs that pass through a gene). Assignment of the genes to the communities is summarized along with regulation status in bladder cancer clusters and the vertex importance metrics. Top 5 genes of each community with the largest degree values are presented. The entire table is available as a supplementary Excel file.

| **Community name** | **Gene symbol** | **Regulation in cluster #1** | **Regulation in cluster #2** | **Regulation in cluster #3** | **Degree** | **Hub score** | **Betweenness** |
| --- | --- | --- | --- | --- | --- | --- | --- |
| Community 1: Differentiation, EMT | *FGFR1* | upregulated | ns | downregulated | 444 | 6.3e-08 | 514 |
|  | *ANXA6* | upregulated | ns | downregulated | 409 | 7.3e-08 | 1478 |
|  | *FGF2* | upregulated | ns | downregulated | 403 | 7.1e-08 | 743 |
|  | *EMP3* | upregulated | ns | downregulated | 402 | 1.7e-07 | 1089 |
|  | *MAP1B* | upregulated | ns | downregulated | 392 | 5.6e-08 | 508 |
| Community 2: Epidermis, signaling | *S100P* | downregulated | ns | upregulated | 648 | 0.95 | 285 |
|  | *EPN3* | downregulated | ns | upregulated | 637 | 1 | 129 |
|  | *KIAA1522* | downregulated | ns | upregulated | 636 | 0.97 | 388 |
|  | *TMBIM1* | downregulated | ns | upregulated | 635 | 0.98 | 140 |
|  | *IKZF2* | downregulated | ns | upregulated | 634 | 1 | 391 |
| Community 3: Epidermis, keratinocytes | *AQP3* | downregulated | ns | upregulated | 573 | 0.82 | 375 |
|  | *HCAR2* | downregulated | ns | ns | 557 | 0.83 | 23753 |
|  | *TRIM29* | downregulated | ns | upregulated | 553 | 0.82 | 650 |
|  | *KRT13* | downregulated | ns | upregulated | 539 | 0.78 | 381 |
|  | *HCAR3* | downregulated | ns | upregulated | 523 | 0.78 | 185 |

Table 54: Biological process gene ontology (GO) term enrichment in communities of co-expressed genes found to be differentially regulated in bladder cancer clusters of DepMap urothelial cancer cell lines. Communities of the co-expressed differentially regulated genes were identified by Leiden algorithm. Biological process GO enrichment for the community members was performed by GOANA algorithm. Enrichment of a GO term in a community as compared with the entire genome was measured by odds ratio. The enrichment p values were adjusted for multiple testing with the false discovery rate (FDR) method. Significantly enriched GO terms were identified by pFDR < 0.05, OR >= 1.44, and at least 10 genes assigned to a term. The table is available as a supplementary Excel file.

|  |
| --- |
|  |

Table 55: Gene set variation analysis of Reactome Pathway gene signatures in bladder cancer clusters of DepMap urothelial cancer cell lines. Statistical significance of differences in ssGSEA scores (single sample gene set enrichment score) of the signatures between bladder cancer clusters was assessed by one-way ANOVA with eta-square effect size statistic. Statistical significance of the difference between ssGSEA score mean in a cluster and the respective ssGSEA score average in the data set was determined by one sample T test. P values were corrected for multiple testing with the false discovery rate (FDR) method. Significantly regulated signatures were considered for p(ANOVA) < 0.05, eta-square >= 0.06, and p(T test) < 0.05. The analysis results are presented for signatures found to be significantly regulated. The table is available as a supplementary Excel file.

|  |
| --- |
|  |

Table 56: Assignment of differentially regulated Reactome Pathway gene signatures in bladder cancer clusters of DepMap urothelial cancer cell lines to communities of co-regulated features in a network analysis. Co-expression networks of Reactome Pathway gene signatures found to be differentially regulated between bladder cancer clusters of the DepMap data set were constructed based on Jaccard distances J that quantify overlapping gene members. The network edges were formed by gene signatures with pairwise J of at least 0.3. Communities of the network were identified by Leiden algorithm and the communities were named after their key biological processes. The table is available as a supplementary Excel file.

|  |
| --- |
|  |

Table 57: Differential expression of matrisome genes in bladder cancer clusters of DepMap urothelial cancer cell lines. Significance of differential expression as compared with the data set average was tested for log2-transformed expression values by one-way ANOVA and one-sample T test. P values were corrected for multiple testing with the false discovery rate method. log2 fold-regulation estimates with 95% confidence intervals in bladder cancer clusters as compared with the data set averages and p values of the T test are summarized. The table is available as a supplementary Excel file.

|  |
| --- |
|  |

Table 58: Activity of collecTRI transcriptional regulons in bladder cancer clusters of DepMap urothelial cancer cell lines as compared with the data set average. The regulon activity was modeled by the univariable linear regression tool of decoupleR package. The algorithm was fed with p value-weighted estimates of differential gene expression in bladder cancer clusters as compared with the cohort average obtained by one-sample T test. Activity of the regulons was gauged by linear modeling score (LM score). P values were corrected for multiple testing with the false discovery rate (FDR) method. Significantly activated features were defined by pFDR < 0.05 and LM score > 0. Significantly inhibited features were considered for pFDR < 0.05 and LM score < 0. Regulons found to be significantly activated or inhibited are listed. The table is available as a supplementary Excel file.

|  |
| --- |
|  |

Table 59: Activity of PROGENy signaling pathways in bladder cancer clusters of DepMap urothelial cancer cell lines as compared with the data set average. The pathway activity was modeled by the multivariable linear regression tool of decoupleR package. The algorithm was fed with p weighted estimates of differential gene expression in bladder cancer clusters as compared with the cohort average obtained by one-sample T test. Activity of the signaling pathways was gauged by linear modeling score (LM score). P values were corrected for multiple testing with the false discovery rate (FDR) method. Significantly activated features were defined by pFDR < 0.05 and LM score > 0. Significantly inhibited features were considered for pFDR < 0.05 and LM score < 0. Signaling pathways found to be significantly activated or inhibited are listed. The table is available as a supplementary Excel file.

|  |
| --- |
|  |

Table 60: Modeling of gene effects of CRISPR-Cas9 gene knockouts in bladder cancer clusters of DepMap cell lines. Distribution of Chronos scores of CRISP-Cas9 gene effects (positive values correspond to growth inhibition) in bladder cancer clusters of DepMap urothelial cancer cell lines was modeled by bootstrapping. Significant gene effects were considered for mean Chronos score > 0.5 with raw p value unadjusted for multiple testing p < 0.05 Estimates means of the gene effects with 95% confidence intervals (95% CI) are listed for genes with significant effects in at least one of the clusters. The analysis results are displayed for top 10 genes with the largest effects in the clusters. The full table is available as a supplementary Excel file.

| **Bladder cancer cluster** | **Gene symbol** | **Entrez ID** | **Gene effect, mean with 95% CI** | **Raw p value** | **FDR p value** |
| --- | --- | --- | --- | --- | --- |
| #1 | *ATP6V1B2* | 526 | 2.2 [95%CI: 1.9 to 2.4] | 0.001 | 0.0028 |
|  | *PSMB6* | 5694 | 1.5 [95%CI: 1.2 to 1.8] | 0.001 | 0.0028 |
|  | *WDR55* | 54853 | 1.4 [95%CI: 1.3 to 1.4] | 0.001 | 0.0028 |
|  | *CDC42* | 998 | 1.3 [95%CI: 1.1 to 1.5] | 0.001 | 0.0028 |
|  | *PABPC1* | 26986 | 1.3 [95%CI: 1.2 to 1.4] | 0.001 | 0.0028 |
|  | *CHD4* | 1108 | 1.2 [95%CI: 1.1 to 1.3] | 0.001 | 0.0028 |
|  | *RBM14* | 10432 | 1.2 [95%CI: 1.1 to 1.2] | 0.001 | 0.0028 |
|  | *SRSF11* | 9295 | 1.2 [95%CI: 1.1 to 1.3] | 0.001 | 0.0028 |
|  | *ACTR1A* | 10121 | 1.1 [95%CI: 0.85 to 1.3] | 0.001 | 0.0028 |
|  | *CHMP4B* | 128866 | 1.1 [95%CI: 0.84 to 1.3] | 0.001 | 0.0028 |
|  | *ELP5* | 23587 | 1.1 [95%CI: 0.89 to 1.3] | 0.001 | 0.0028 |
|  | *EXOSC8* | 11340 | 1.1 [95%CI: 0.96 to 1.2] | 0.001 | 0.0028 |
|  | *LSG1* | 55341 | 1.1 [95%CI: 1 to 1.2] | 0.001 | 0.0028 |
|  | *TRIAP1* | 51499 | 1.1 [95%CI: 0.9 to 1.2] | 0.001 | 0.0028 |
| #2 | *ATP6V1B2* | 526 | 1.8 [95%CI: 1.5 to 2] | 0.001 | 0.0028 |
|  | *TRIAP1* | 51499 | 1.6 [95%CI: 1.3 to 1.9] | 0.001 | 0.0028 |
|  | *EIF1AX* | 1964 | 1.5 [95%CI: 1 to 1.8] | 5e-04 | 0.0028 |
|  | *PSMB6* | 5694 | 1.4 [95%CI: 0.89 to 1.9] | 0.0015 | 0.004 |
|  | *ELP5* | 23587 | 1.3 [95%CI: 1.1 to 1.4] | 0.001 | 0.0028 |
|  | *EXOSC8* | 11340 | 1.2 [95%CI: 1 to 1.4] | 0.001 | 0.0028 |
|  | *PTPN23* | 25930 | 1.2 [95%CI: 0.99 to 1.3] | 0.001 | 0.0028 |
|  | *RBM14* | 10432 | 1.2 [95%CI: 1 to 1.3] | 0.001 | 0.0028 |
|  | *TOP1* | 7150 | 1.2 [95%CI: 1.1 to 1.3] | 0.001 | 0.0028 |
|  | *CAPZB* | 832 | 1.1 [95%CI: 0.94 to 1.4] | 0.001 | 0.0028 |
|  | *CHMP4B* | 128866 | 1.1 [95%CI: 0.87 to 1.3] | 0.001 | 0.0028 |
|  | *DDX42* | 11325 | 1.1 [95%CI: 1 to 1.2] | 0.001 | 0.0028 |
|  | *GPN3* | 51184 | 1.1 [95%CI: 1 to 1.3] | 0.001 | 0.0028 |
|  | *SNAP23* | 8773 | 1.1 [95%CI: 0.88 to 1.4] | 0.001 | 0.0028 |
|  | *WDR55* | 54853 | 1.1 [95%CI: 1 to 1.2] | 0.001 | 0.0028 |
| #3 | *ATP6V1B2* | 526 | 1.7 [95%CI: 1.5 to 1.9] | 0.001 | 0.0028 |
|  | *EIF1AX* | 1964 | 1.5 [95%CI: 1.1 to 1.9] | 0.001 | 0.0028 |
|  | *GPN3* | 51184 | 1.5 [95%CI: 1.2 to 1.7] | 0.001 | 0.0028 |
|  | *RBM14* | 10432 | 1.4 [95%CI: 1.4 to 1.5] | 0.001 | 0.0028 |
|  | *PTPN23* | 25930 | 1.3 [95%CI: 1 to 1.6] | 0.001 | 0.0028 |
|  | *TRIAP1* | 51499 | 1.3 [95%CI: 0.93 to 1.6] | 0.001 | 0.0028 |
|  | *CAPZB* | 832 | 1.2 [95%CI: 1.1 to 1.4] | 0.001 | 0.0028 |
|  | *NUP43* | 348995 | 1.2 [95%CI: 1 to 1.5] | 0.001 | 0.0028 |
|  | *THOC1* | 9984 | 1.2 [95%CI: 1 to 1.4] | 0.001 | 0.0028 |
|  | *WDR55* | 54853 | 1.2 [95%CI: 1 to 1.3] | 0.001 | 0.0028 |

Table 61: Differential gene expression in bladder cancer clusters of NMIBC, UROMOL molecular classes of NMIBC, and consensus classes of MIBC. The differential gene expression in the molecular subsets as compared with the cohort average was investigated by one-way ANOVA with eta-square effect size statistic and one-sample post-hoc T test. P values were corrected for multiple testing with the false discovery rate (FDR) method. Significant regulation was considered for ANOVA pFDR < 0.05, eta-square >= 0.06, and T test pFDR < 0.05. Log2 fold-change (FC) estimates with 95% confidence intervals (95%CI), and FDR-adjusted p values in one-way ANOVA and post-hoc tests are listed for genes found to be significantly regulated in at least three cohorts. The table is available as a supplementary Excel file.

|  |
| --- |
|  |

Table 62: Genes differentially regulated in at least three cohorts shared by bladder cancer clusters and UROMOL classes in NMIBC, and bladder cancer clusters and consensus classes in NMIBC. Molecular subset names, and regulation status in the molecular subsets as compared with the cohort mean are listed. The table is available as a supplementary Excel file.

|  |
| --- |
|  |

Table 63: Similarity of bladder cancer clusters and UROMOL classes of NMIBC, bladder cancer clusters and consensus classes of NMIBC in respect to shared differentially regulated genes. Numbers of concordantly up- and downregulated genes in the molecular subsets as compared with the cohort average, and Jaccard similarity coefficients are listed.

| **Cancer entity** | **Bladder cancer cluster** | **UROMOL/consensus class** | **Shared upregulated genes, N** | **Shared downregulated genes, N** | **Similarity coefficient, J** |
| --- | --- | --- | --- | --- | --- |
| NMIBC | #1 | 1 | 0 | 0 | 0 |
|  |  | 2a | 0 | 6 | 0.0028 |
|  |  | 2b | 454 | 24 | 0.35 |
|  |  | 3 | 0 | 1 | 0.00044 |
|  | #2 | 1 | 4 | 2 | 0.0044 |
|  |  | 2a | 0 | 3 | 0.0018 |
|  |  | 2b | 221 | 47 | 0.24 |
|  |  | 3 | 4 | 7 | 0.0062 |
|  | #3 | 1 | 26 | 78 | 0.056 |
|  |  | 2a | 8 | 253 | 0.13 |
|  |  | 2b | 0 | 0 | 0 |
|  |  | 3 | 9 | 404 | 0.21 |
| MIBC | #1 | Stroma-rich | 436 | 103 | 0.2 |
|  |  | LumU | 5 | 76 | 0.029 |
|  |  | LumNS | 51 | 47 | 0.035 |
|  |  | Ba/Sq | 43 | 37 | 0.022 |
|  |  | LumP | 2 | 20 | 0.0063 |
|  |  | NE-like | 14 | 42 | 0.02 |
|  | #2 | Stroma-rich | 107 | 10 | 0.025 |
|  |  | LumU | 5 | 6 | 0.0025 |
|  |  | LumNS | 8 | 0 | 0.0018 |
|  |  | Ba/Sq | 483 | 607 | 0.26 |
|  |  | LumP | 4 | 1 | 0.00099 |
|  |  | NE-like | 46 | 62 | 0.025 |
|  | #3 | Stroma-rich | 1 | 31 | 0.006 |
|  |  | LumU | 48 | 66 | 0.023 |
|  |  | LumNS | 98 | 55 | 0.031 |
|  |  | Ba/Sq | 0 | 3 | 0.00051 |
|  |  | LumP | 432 | 374 | 0.17 |
|  |  | NE-like | 6 | 6 | 0.0024 |

Table 64: Biological process gene ontology (GO) enrichment analysis for genes concordantly regulated in bladder cancer clusters and UROMOL classes of NMIBC, and bladder cancer clusters and consensus classes of MIBC. Enrichment magnitude in the shared gene sets as compared with the entire genome was measured by the odds ratio statistic. P values were corrected for multiple testing with the false discovery rate (FDR) method. Significant enrichment was considered for odds ratio >= 1.44, at least three regulated genes per term, and pFDR < 0.05. Results for significantly enriched GO terms are listed. The table is available as a supplementary Excel file.

|  |
| --- |
|  |

Table 65: Marker genes of bladder cancer clusters of NMIBC, UROMOL classes of NMIBC, bladder cancer clusters of MIBC, and consensus classes of MIBC. The marker genes were identified by false discovery rate (FDR) corrected one-way ANOVA with eta-square effect size statistic, and receiver-operating characteristic. Markers of the molecular subsets were identified by significant differences in expression with ANOVA pFDR < 0.05 and eta-square >= 0.06, and area under the ROC curve (AUC) >= 0.714. The optimal cutoffs of log2 gene expression levels for identification of the molecular subset found with the Youden criterion, sensitivity, specificity, and AUC are listed for the marker genes shared by at least three cohorts. The table is available as a supplementary Excel file.

|  |
| --- |
|  |

Table 66: Assignment of cancer samples in the TCGA BLCA cohort to pan-carcinoma luminal A (LumA), lumunal B (LumB), and basal PAM50 molecular subsets by Zhao et al. The table is available as a supplementary Excel file.

|  |
| --- |
|  |

Table 67: Differential expression of PAM50 genes in bladder cancer clusters and PAM50 molecular subsets of the TCGA BLCA data set as compared with the cohort average. The differential gene expression in the molecular subsets as compared with the cohort average was investigated by one-way ANOVA with eta-square effect size statistic and one-sample post-hoc T test. P values were corrected for multiple testing with the false discovery rate (FDR) method. Significant regulation was considered for ANOVA pFDR < 0.05, eta-square >= 0.06, and T test pFDR < 0.05. Log2 fold-change (FC) estimates with 95% confidence intervals (95%CI), and FDR-adjusted p values in one-way ANOVA and post-hoc tests are listed. The table is available as a supplementary Excel file.

|  |
| --- |
|  |

Table 68: Summary of the RNA-seq data performed on the three representative cell line clusters treated with neratinib, afatinib, trametinib and erdafitinib. The complete statistical results from the blocked gene set enrichment analysis are summarized. The table is available as a supplementary Excel file.

|  |
| --- |
|  |

# Supplementary Figures


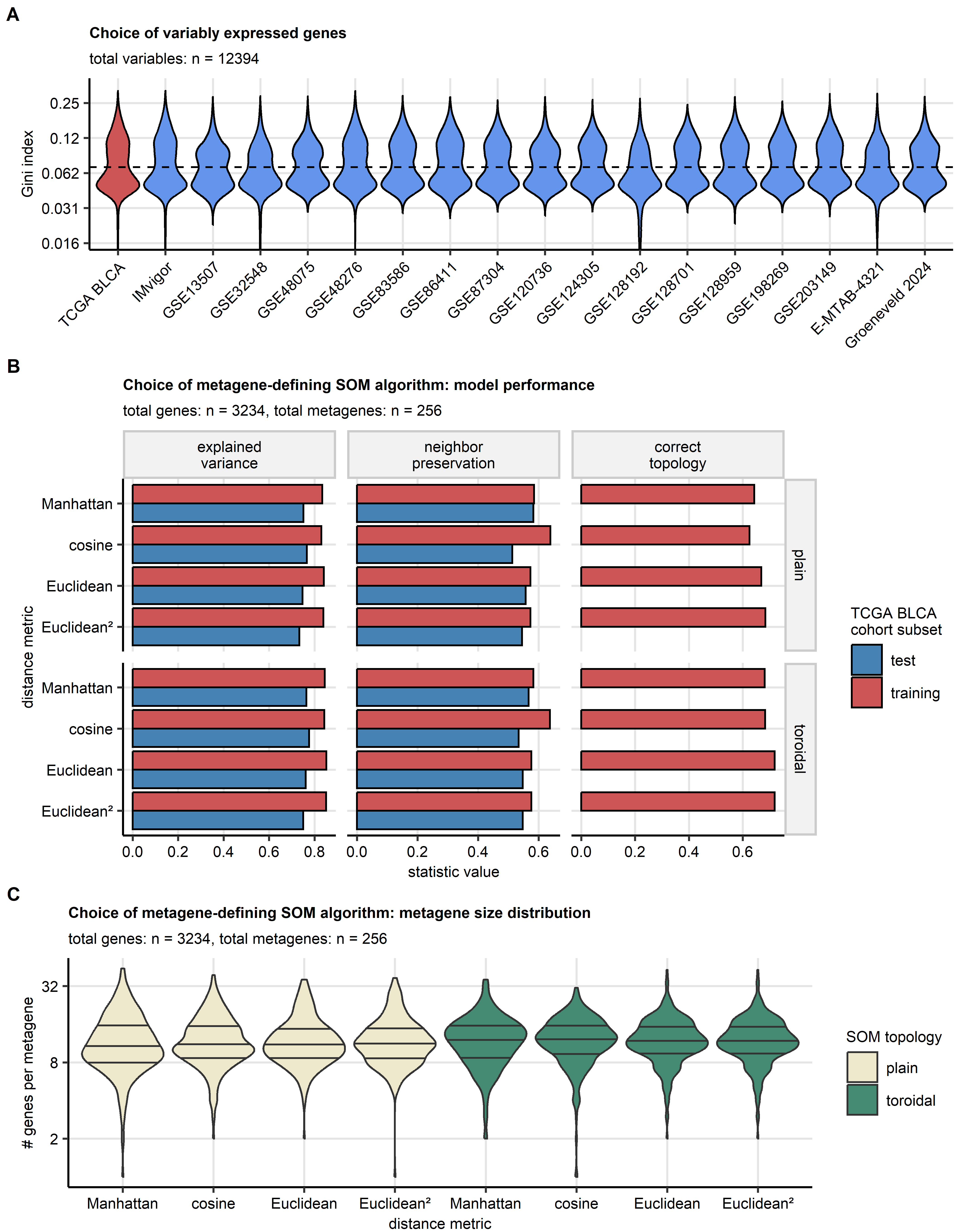


**Supplementary Figure S1. Selection of genes and self-organizing map algorithm used for definition of metagenes in the TCGA BLCA training cohort.**

*A total of 12394 transcripts were measured in all investigated transcriptomic cohorts. Their* $log_{2}$*-transformed levels were subjected to batch correction with the ComBat algorithm, and 3234 genes with sufficient expression variability used for definition of metagenes were selected with a cutoff of Gini index of G = 0.07.* *Self-organizing map (SOM) algorithm was used to define metagenes, i.e. micro-clusters of tightly co-expressed transcripts. The data used by SOM were ComBat-processed* $log_{2}$*-transformed expression levels of genes passing the Gini index cutoff as the selection criterion. Several 16* $\times$ *16 unit hexagonal SOM objects with different gene - node distance metrics (Euclidean, Manhattan, cosine, and squared Euclidean) and topology types (plain or toroidal) were developed in a training subset of the TCGA BLCA cohort consisting of randomly selected three-fourths of the samples (n = 305 specimens). Subsequently, the gene - SOM node assignment was predicted for the test subset of the TCGA BLCA cohort (n = 102).* *Explained clustering variance (ratio of the total between metagene sum of squares to the total sum of squares), neighborhood preservation (mean fraction of the five nearest gene neighbors in the expression data set assigned to the same metagene), and fraction of metagenes with the correct SOM topology (mean fraction of neighbor nodes prior to training preserved in the final SOM) were used to assess quality of representation of the genuine gene expression data by the metagene structure, and to select the final metagene-refining SOM algorithm.*

*(A) Selection of variably expressed genes used for definition of metagenes. Distributions of Gini indexes for the ComBat-adjusted* $log_{2}$ *expression levels in the transcriptomic cohorts are presented in violin plots. The dashed line represents the Gini index cutoff. The total number of genes measured in all transcriptomic cohorts is displayed in the plot caption.*

*(B) Choice of the SOM algorithm for development of metagenes. Evaluation of performance of SOM algorithms in the training and test subsets of the TCGA BLCA cohort. Note the good reproducibility of the squared Euclidean distance toroidal SOM selected as the final metagene-defining procedure in terms of explained clustering variance and neighborhood preservation in the training and test subsets of the TCGA BLCA cohort. The squared Euclidean distance toroidal SOM was also characterized by the highest rate of nodes with correct topology. The total number of variably expressed genes used for the SOM construction and the number of metagenes are displayed in the plot caption.*

*(C) Choice of the SOM algorithm for development of metagenes. Distribution of sizes of metagenes defined by various SOM algorithms presented in violin plots. Horizontal lines in the violins represent median metagene sizes with interquartile ranges. The total number of variably expressed genes used for the SOM construction and the number of metagenes are displayed in the plot caption.*


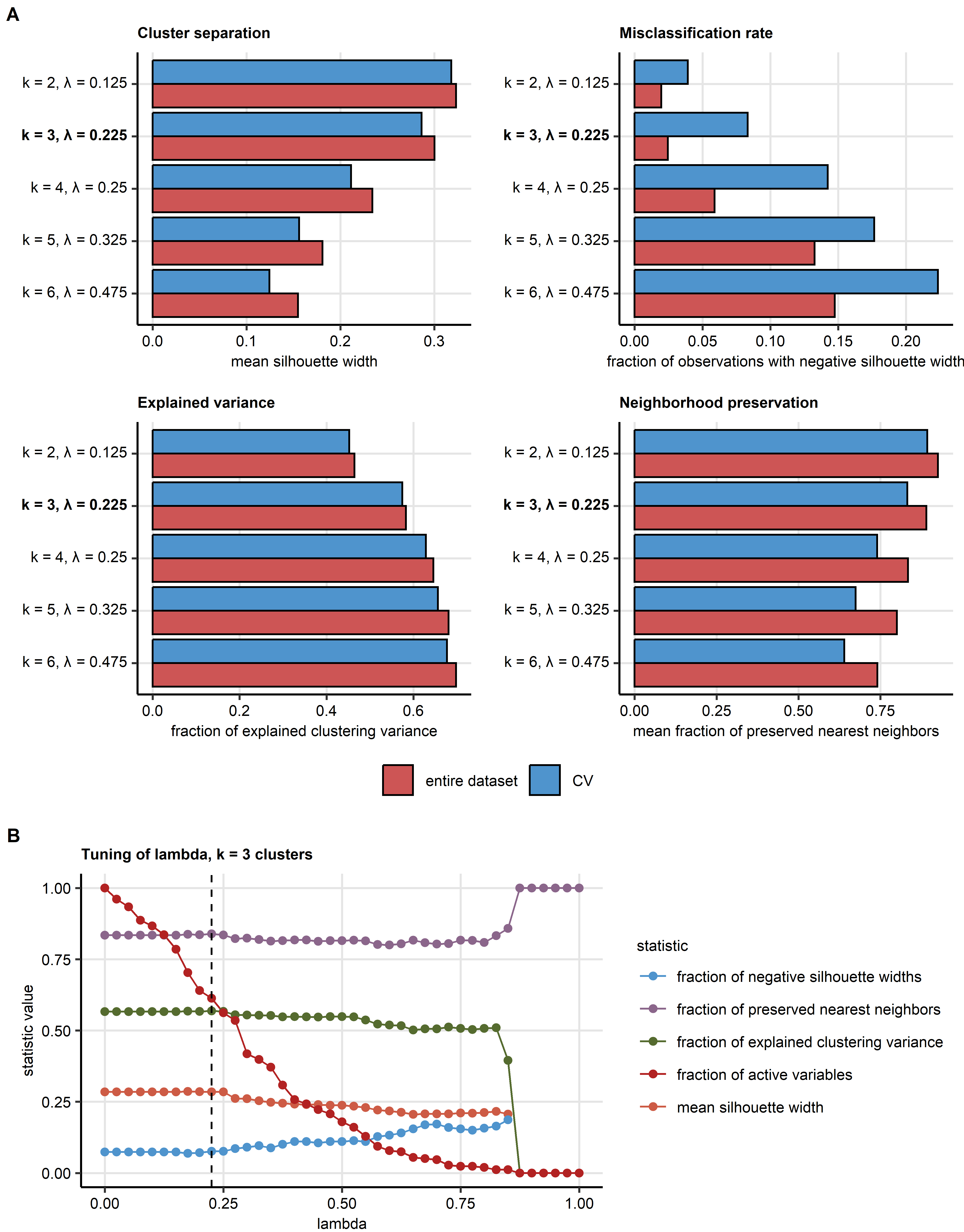


**Supplementary Figure S2. Tuning of the regularized KMEANS algorithm for definition of bladder cancer clusters.**

*Cancer samples in the TCGA BLCA cohort (n = 407) were subjected to unsupervised clustering in respect to ssGSEA (single sample gene set enrichment analysis) scores of the SOM-defined metagenes with hard-threshold regularized KMEANS algorithm with varying cluster numbers k. For each cluster number scenario, choice of the shrinkage hyper-parameter* $\lambda$ *was done by cross-validated tuning. Quality of the clustering scenarios was assessed in the entire data set and 5-fold cross-validation (CV) by fraction of explained clustering variance (ratio of the total between cluster sum of squares to the total sum of squares) as a measure of explanatory performance or information content, neighborhood preservation (mean fraction of the five nearest neighbors in the data set placed in the same cluster), mean silhouette width as a measure of cluster separation, and fraction of observations with negative silhouette widths indicative of misclassification.*

*(A) Choice of number of bladder cancer clusters. Statistics of evaluation of the clustering scenarios with varying numbers of clusters k. Note a good trade-off between explanatory performance, cluster separation and rate of misclassification for the k = 3 cluster solution (highlighted with bold font). The k = 3 cluster algorithm was used for definition of bladder cancer clusters.*

*(B) Choice of the optimal* $\lambda$ *value for a three cluster scenario. The optimal value of* $\lambda$ *= 0.225 was selected by the peaks of explained clustering variance, mean silhouette width and neighborhood preservation, and is represented by the vertical dashed line.*


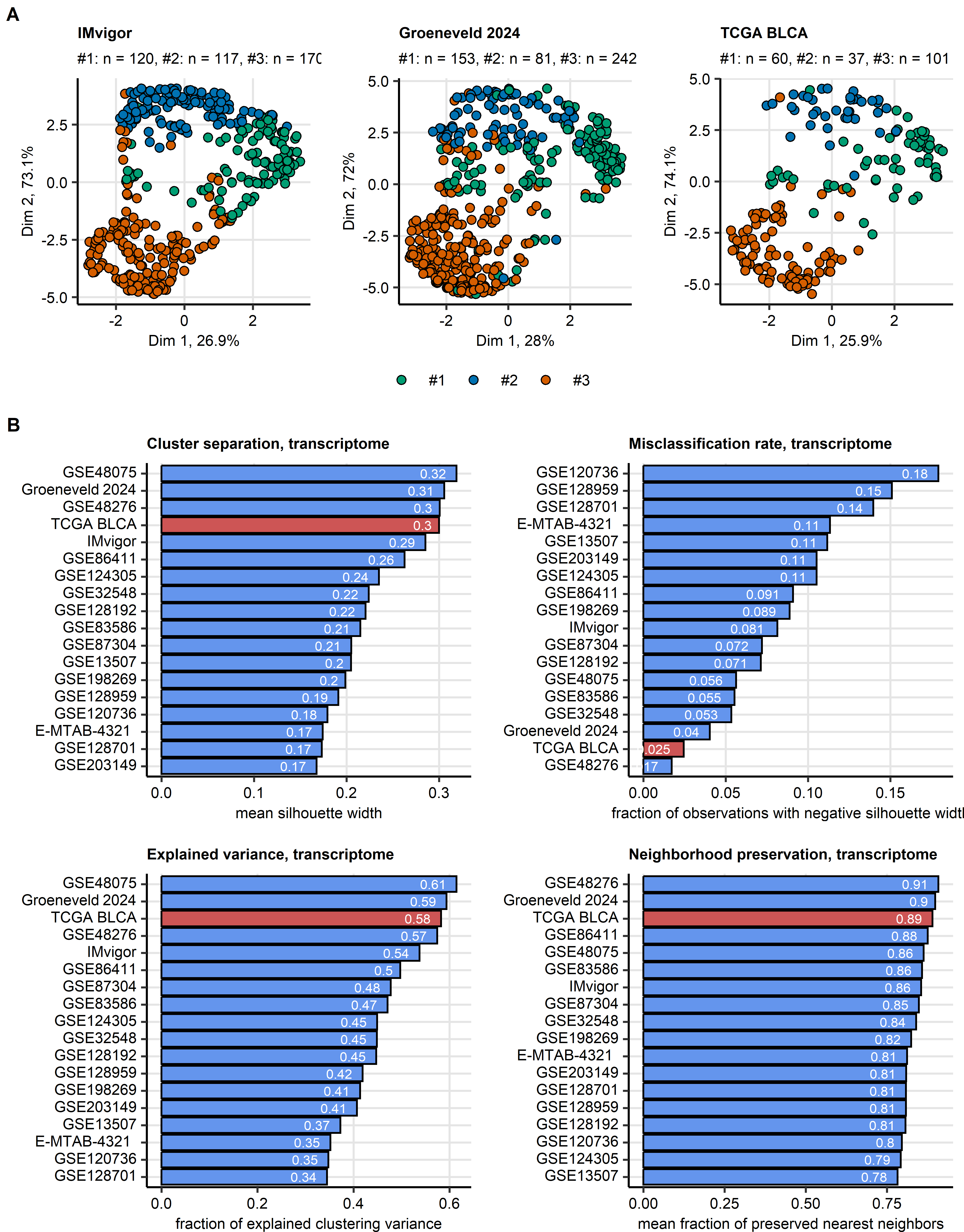


**Supplementary Figure S3. Assignment of transcriptomic urothelial cancer samples to bladder cancer clusters. Evaluation of clustering of the transcriptome data.**

*Three clusters of bladder cancer samples were defined in the TCGA BLCA training cohort of bulk cancer transcriptomes in respect to the metagene ssGSEA (single sample gene set enrichment analysis) scores by the regularized hard-threshold KMEANS algorithm. ssGSEA scores of the metagenes were computed for 17 validation collectives of bulk cancer transcriptomes (TCGA BLCA: n = 407, IMvigor: n = 221, GSE13507: n = 188, GSE32548: n = 131, GSE48075: n = 142, GSE48276: n = 116, GSE83586: n = 307, GSE86411: n = 132, GSE87304: n = 305, GSE120736: n = 145, GSE124305: n = 133, GSE128192: n = 112, GSE128701: n = 136, GSE128959: n = 192, GSE198269: n = 394, GSE203149: n = 171, E-MTAB-4321: n = 476, and Groeneveld 2024: n = 198).* *Assignment of bulk cancer transcriptomes in the validation cohorts to bladder cancer clusters in respect to the ssGSEA metagene scores was done with an inverse distance-weighted k-nearest neighbor classifier.* *The metagene development, metagene clustering, and transcriptome sample assignment process is documented in Supplementary Tables S5 - S9.*

*(A) Visualization of bladder cancer cluster by dimensionality reduction of the metagene data set. Cluster assignment in the TCGA BLCA training cohort (muscle invasive bladder cancers [MIBC]), E-MTAB 4321 collective (non-muscle invasive bladder cancers [NMIBC]), and the Groeneveld 2024 cohort (NMIBC and MIBC) was visualized in two - dimensional UMAP (uniform manifold approximation and projection) embeddings of the metagene ssGSEA scores. Each point represents a single cancer sample; cluster assignment is color coded. Numbers of observations are displayed in the plot captions.*

*(B) Evaluation of performance and technical validation of the bladder cancer cluster classification scheme. Quality of the clustering structures in the TCGA BLCA training cohort (red bars) and the transcriptomic validation collectives (blue bars) was assessed by fraction of explained clustering variance (ratio of the total between cluster sum of squares to the total sum of squares) as a measure of explanatory performance, neighborhood preservation (mean fraction of the nearest five neighbors placed in the same cluster), mean silhouette width as a readout of cluster separation, and fraction of observations with negative silhouette width indicative of misclassification.*


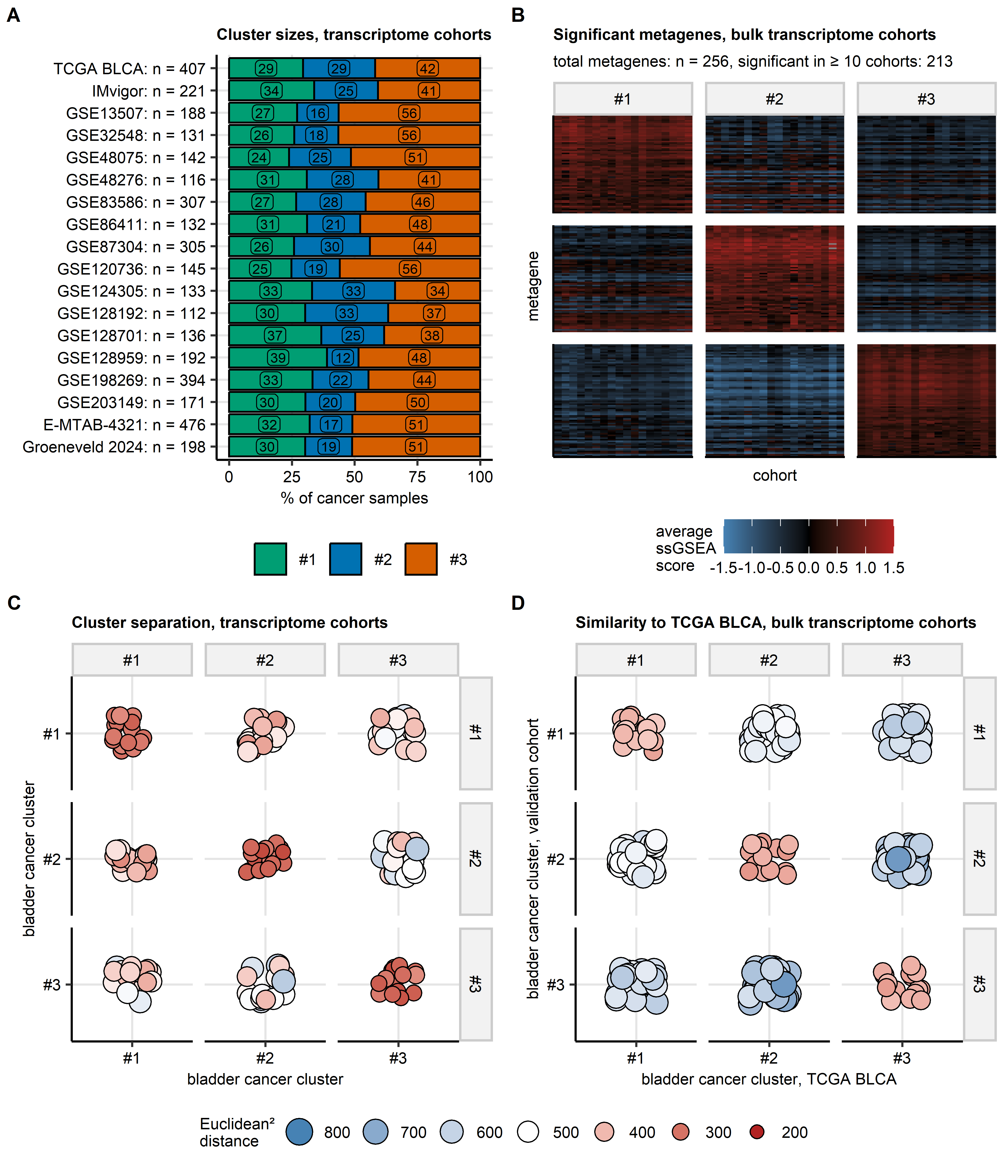


**Supplementary Figure S4. Size distribution, similarity, and separation of bladder cancer clusters in data sets of bulk cancer transcriptomes.**

*Bladder cancer clusters of urothelial cancers classified in respect to metagene single sample gene enrichment analysis (ssGSEA) scores were defined in the TCGA BLCA training cohort of bulk cancer transcriptomes and predicted for 17 independent transcriptomic bulk cancer collectives by a k-nearest neighbor procedure (Supplementary Tables S5 - S9).*

*(A) Sizes of bladder cancer clusters. Distribution of sizes of bladder cancer clusters expressed as percentages of all samples in the TCGA BLCA cohort and the validation transcriptomic collectives. Total numbers of samples in the cohorts are indicated in the Y axis.*

*(B) Expression of the cluster-defining metagenes in bladder cancer clusters. ssGSEA scores of the cluster-defining metagenes were compared between bladder cancer clusters by false discovery rate (FDR) corrected Kruskal-Wallis test and* $\eta^{2}$ *effect size statistic. Differentially regulated metagenes were identified by the pFDR < 0.05 and* $\eta^{2}\geq$ *0.06 cutoffs indicative of significant, moderate-to-large differences between the clusters. Cluster averages of metagene ssGSEA scores for differentially regulated metagenes shared by at least 10 cohorts are presented in a heat map. The total number of metagenes and the number of metagenes found to be differentially regulated in at least 10 cohorts are displayed in the plot caption. Full testing results are presented in Supplementary Table S10.*

*(C) Separation of bladder cancer clusters in respect to the metagene ssGSEA scores was assessed by pairwise squared Euclidean distances between observations. The distances averaged by cluster are presented in a bubble plot. Each point represents a single cohort; point size and color code for the average distance.*

*(D) Similarity of bladder cancer clusters of bulk cancer transcriptome data. Similarity of bladder cancer clusters in the TCGA BLCA training cohort and the corresponding clusters in the validation transcriptomic cohorts was assessed by pairwise squared Euclidean cross-distances. The distances averaged by cluster are presented in a bubble plot. Each point represents a single cohort; point size and color code for the average distance.*


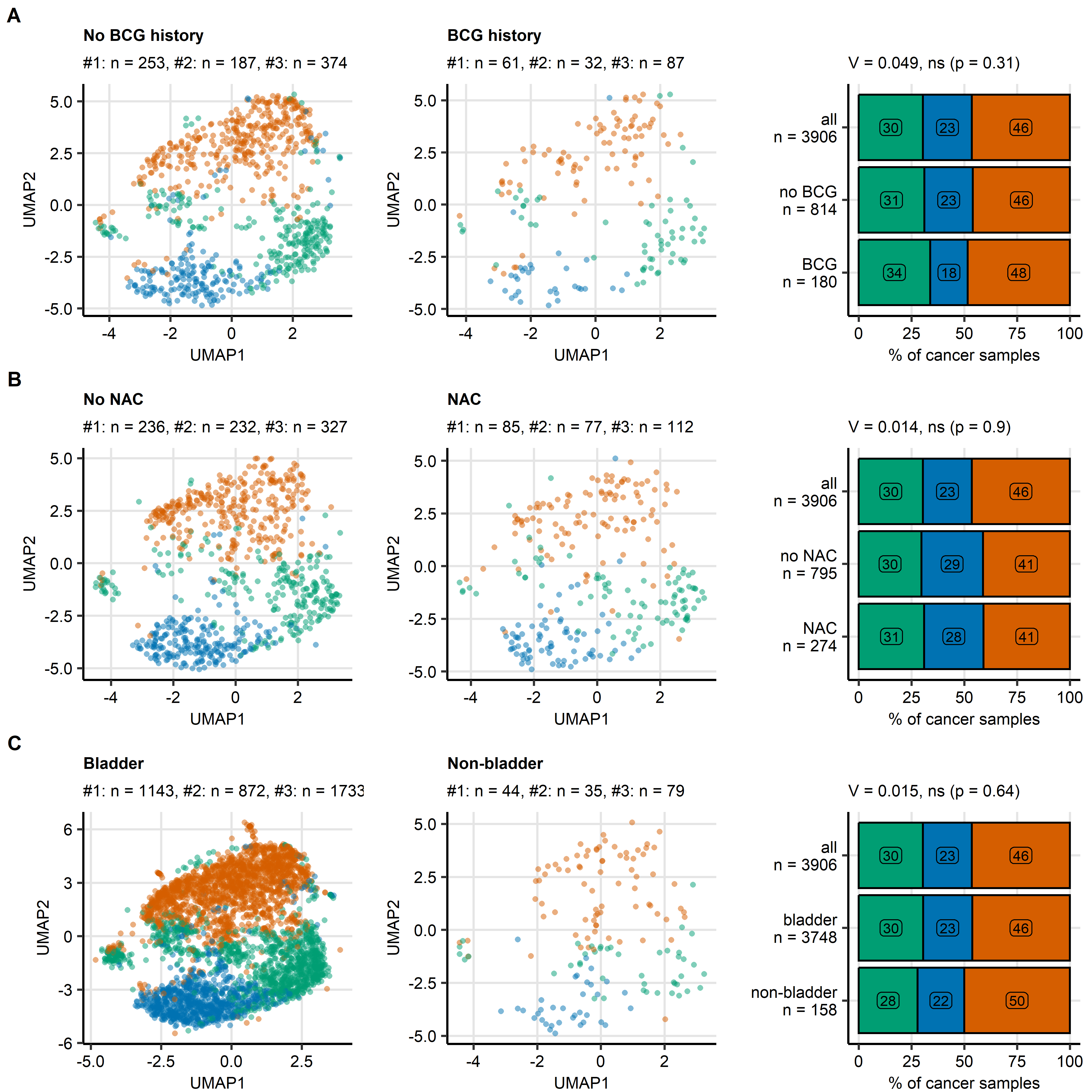


**Supplementary Figure S5. Bladder cancer clusters, BCG treatments, neoadjuvant chemotherapy, and bladder/non-bladder anatomical location.**

*Separation and size distribution of bladder cancer clusters was evaluated in pooled collectives of cancers with and without history of treatment with BCG (Bacillus Calmette-Guerin, A), with and without neoadjuvant systemic chemotherapy (NAC, B), and cancers located in and beyond the bladder (C).*

*Left: visualization of bladder cancer clusters via dimensionality reduction of cluster-defining metagenes. UMAP (uniform manifold approximation and projection) embeddings of scores of cluster-defining metagenes (256 metagenes) were computed and displayed in scatter plots. Each point represents a single cancer sample. Point color codes for cluster assignment. Numbers of samples in bladder cancer clusters are indicated in the plot captions.*

*Right: Sizes of bladder cancer clusters. Differences of sizes of bladder cancer clusters were assessed by* $\chi^{2}$ *test with Cramer’s V effect size statistic. Percentages of samples assigned to bladder cancer clusters are depicted in stack plots. Effect sizes and p values are displayed in the plot captions. Numbers of samples are indicated in the Y axes.*

*Data sources in (A), no BCG: TCGA BLCA, IMvigor, and E-MTAB-4321 cohorts; BCG history: TCGA BLCA, IMvigor, and E-MTAB-4321 cohorts.*

*Data sources in (B), no NAC: TCGA BLCA, IMvigor, GSE48276, and GSE87304 cohorts; NAC history: TCGA BLCA, IMvigor, GSE48276, and GSE124305 cohorts.*

*Data sources in (B), bladder: TCGA BLCA, IMvigor, GSE13507, GSE32548, GSE48075, GSE48276, GSE83586, GSE86411, GSE87304, GSE120736, GSE124305, GSE128192, GSE128701, GSE128959, GSE198269, GSE203149, E-MTAB-4321, and Groeneveld 2024 cohorts; non-bladder: IMvigor and GSE198269 cohorts.*


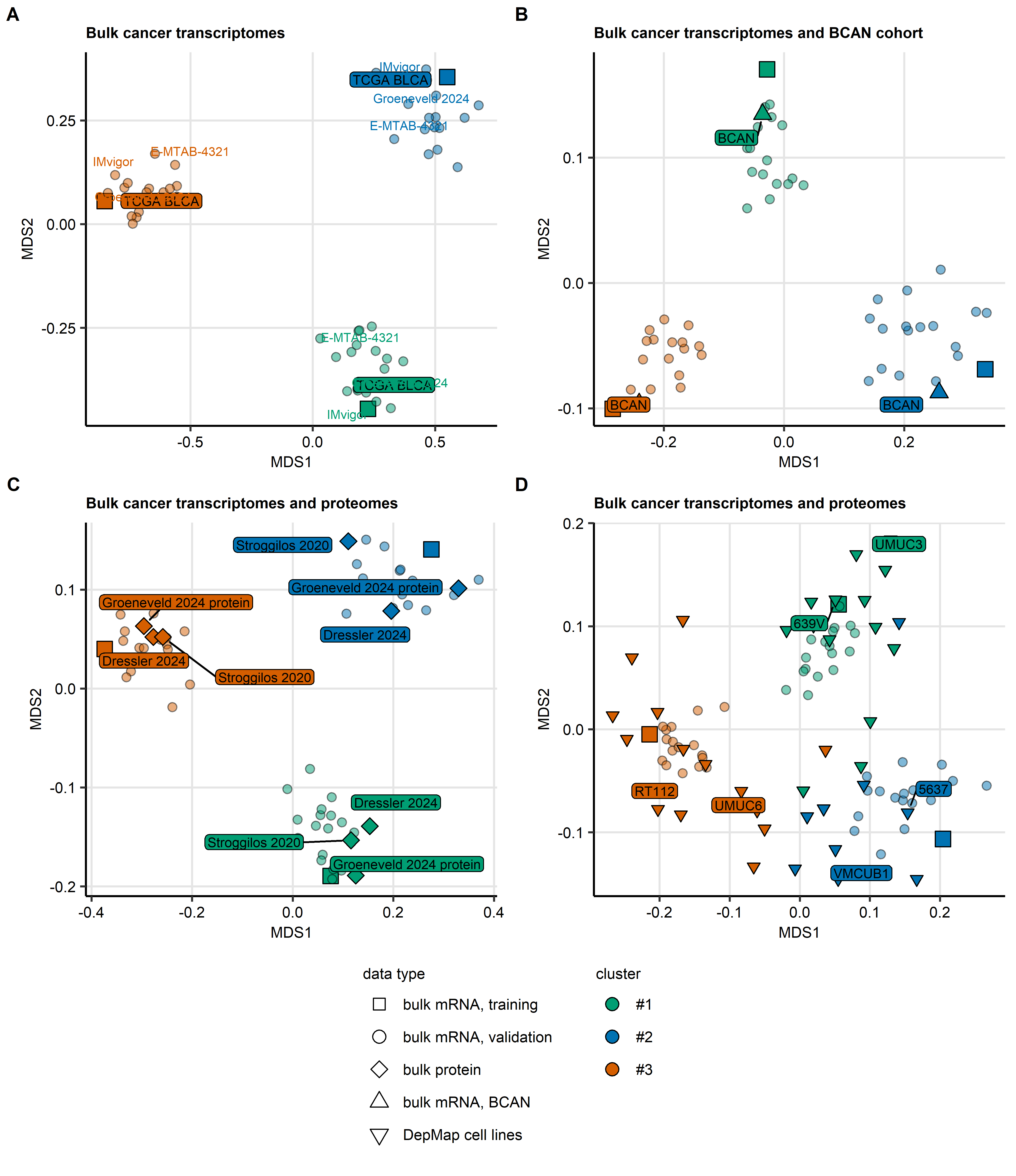


**Supplementary Figure S6. Average correlations with centroids of bladder cancer clusters and similarity of the clusters in bulk cancer transcriptome, bulk cancer proteome and DepMap urothelial cancer cell line data.**

*Samples in 19 transcriptomic bulk cancer cohorts (TCGA BLCA, IMvigor, GSE13507, GSE32548, GSE48075, GSE48276, GSE83586, GSE86411, GSE87304, GSE120736, GSE124305, GSE128192, GSE128701, GSE128959, GSE198269, GSE203149, E-MTAB-4321, Groeneveld 2024, and BCAN), cancer specimens in Groeneveld 2024, Dressler 2024 and Stroggilos 2020 bulk cancer proteomic collectives, and DepMap urothelial cancer cell lines were assigned to bladder cancer clusters by clustering of metagene scores, k-nearest neighbor and Elastic Net classifiers (Supplementary Tables S8, S14, and S51).*

*Similarity of bladder cancer clusters in the TCGA BLCA training cohort and remaining data sets was assessed by Spearman’s correlations with centroids of the clusters in the TCGA BLCA cohort. For the bulk cancer transcriptomes (A; cohorts: TCGA BLCA, IMvigor, GSE13507, GSE32548, GSE48075, GSE48276, GSE83586, GSE86411, GSE87304, GSE120736, GSE124305, GSE128192, GSE128701, GSE128959, GSE198269, GSE203149, E-MTAB-4321, and Groeneveld 2024), the correlations were computed in the data sets of metagene scores (Supplementary Table S6). For BCAN bulk cancer transcriptome cohort (B), the bulk cancer proteomes (C; Groeneveld 2024, Dressler 2024, Stroggilos 2020 cohort), and DepMap urothelial cell lines (D), the correlations were calculated in data sets of ComBat-processed* $log_{2}$ *protein and gene expression levels used by the Elastic Net classifiers listed in Supplementary Table S11.* *Two-dimensional multi-dimensional scaling (MDS) embeddings of average* $\rho$ *coefficients of correlation with the centroids in the investigated data sets are presented in scatter plots. Point color codes for bladder cancer cluster, point shape codes for data type. In (C) symbols representing the proteomic cohorts are labeled with cohort names. In (D), symbols representing cell lines used in in vitro experiments are labeled with cell line names.*


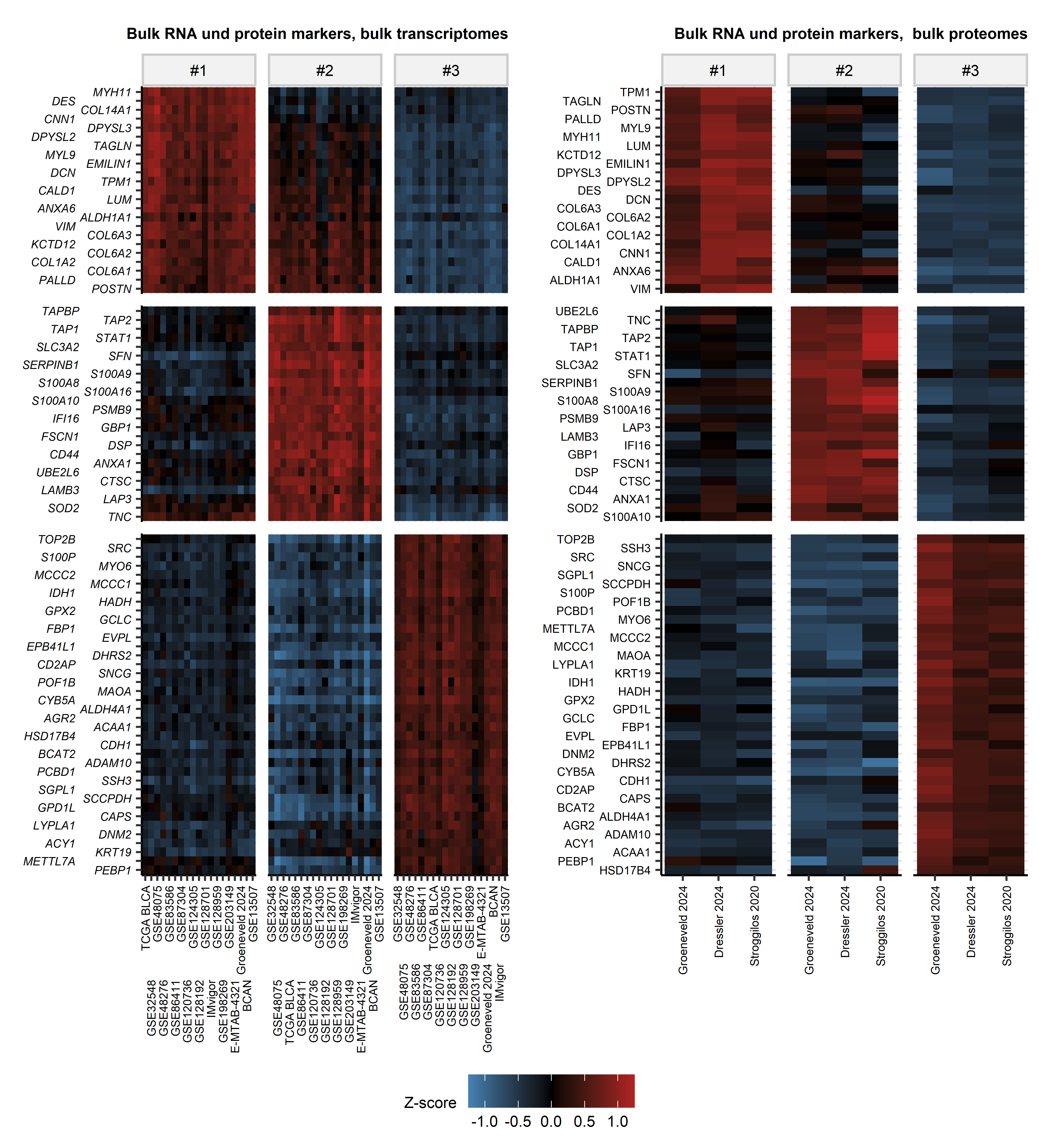


**Supplementary Figure S7. Expression of shared markers of bladder clusters of bulk cancer transcriptomes and proteomes.**

*Differences in* $log_{2}$ *mRNA and protein levels between bladder cancer clusters of 19 bulk cancer transcriptome collectives (TCGA BLCA, IMvigor, GSE13507, GSE32548, GSE48075, GSE48276, GSE83586, GSE86411, GSE87304, GSE120736, GSE124305, GSE128192, GSE128701, GSE128959, GSE198269, GSE203149, E-MTAB-4321, Groeneveld 2024, and BCAN) and three bulk cancer proteome cohorts (Groeneveld 2024, Dressler 2024, Stroggilos 2020) were assessed by false discovery rate (FDR) corrected one-way ANOVA with* $\eta^{2}$ *effect size statistic. The differentially expressed genes and proteins (pFDR < 0.05,* $\eta^{2}\geq$ *0.06) were subjected to receiver-operating characteristic (ROC) analysis. Gene and protein markers of the clusters were considered for area under the ROC curve (AUC)* $\geq$ *0.714.* *Cluster-wise mean Z-scores of* $log_{2}$ *expression levels of 85 cluster markers shared by at least 10 transcriptomic cohorts and two proteomic collectives are presented in heat maps. Each heat map tile represents a mean Z-score in a bladder cancer cluster of one cohort. Cohort names are indicated in the X axes.*


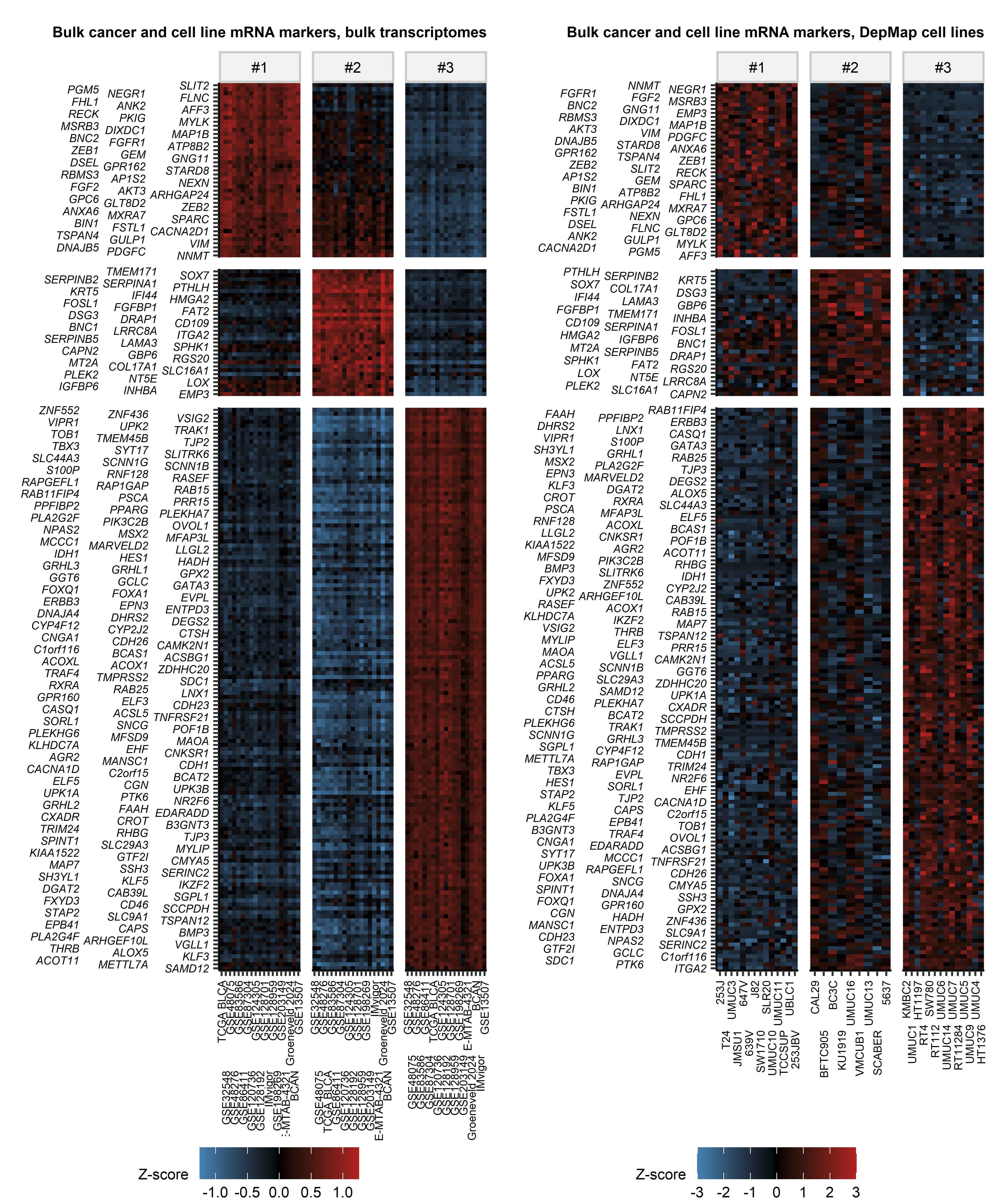


**Supplementary Figure S8. Expression of shared markers of bladder clusters of bulk cancer transcriptomes and urothelial cancer cell lines.**

*Differences in* $log_{2}$ *mRNA gene expression between bladder cancer clusters of 19 collectives (TCGA BLCA, IMvigor, GSE13507, GSE32548, GSE48075, GSE48276, GSE83586, GSE86411, GSE87304, GSE120736, GSE124305, GSE128192, GSE128701, GSE128959, GSE198269, GSE203149, E-MTAB-4321, Groeneveld 2024, and BCAN) of bulk cancer transcriptomes and DepMap urothelial cell lines were assessed by false discovery rate (FDR) corrected one-way ANOVA with* $\eta^{2}$ *effect size statistic. The differentially expressed genes (pFDR < 0.05,* $\eta^{2}\geq$ *0.06) were subjected to receiver-operating characteristic (ROC) analysis and were considered as bladder cluster markers for area under the ROC curve (AUC)* $\geq$ *0.714.* *Cluster-wise mean Z-scores of* $log_{2}$ *expression levels of 216 cluster markers shared by at least 10 transcriptomic cohorts and the DepMap cell line data set are presented in heat maps. For the bulk cancer transcriptomes, each heat map tile represents a mean Z-score in a bladder cancer cluster of one cohort; cohort names are indicated in the X axis. For the DepMap cell lines, each heat map tile represents a Z-score value in one cell line; cell line names are displayed in the X axis.*


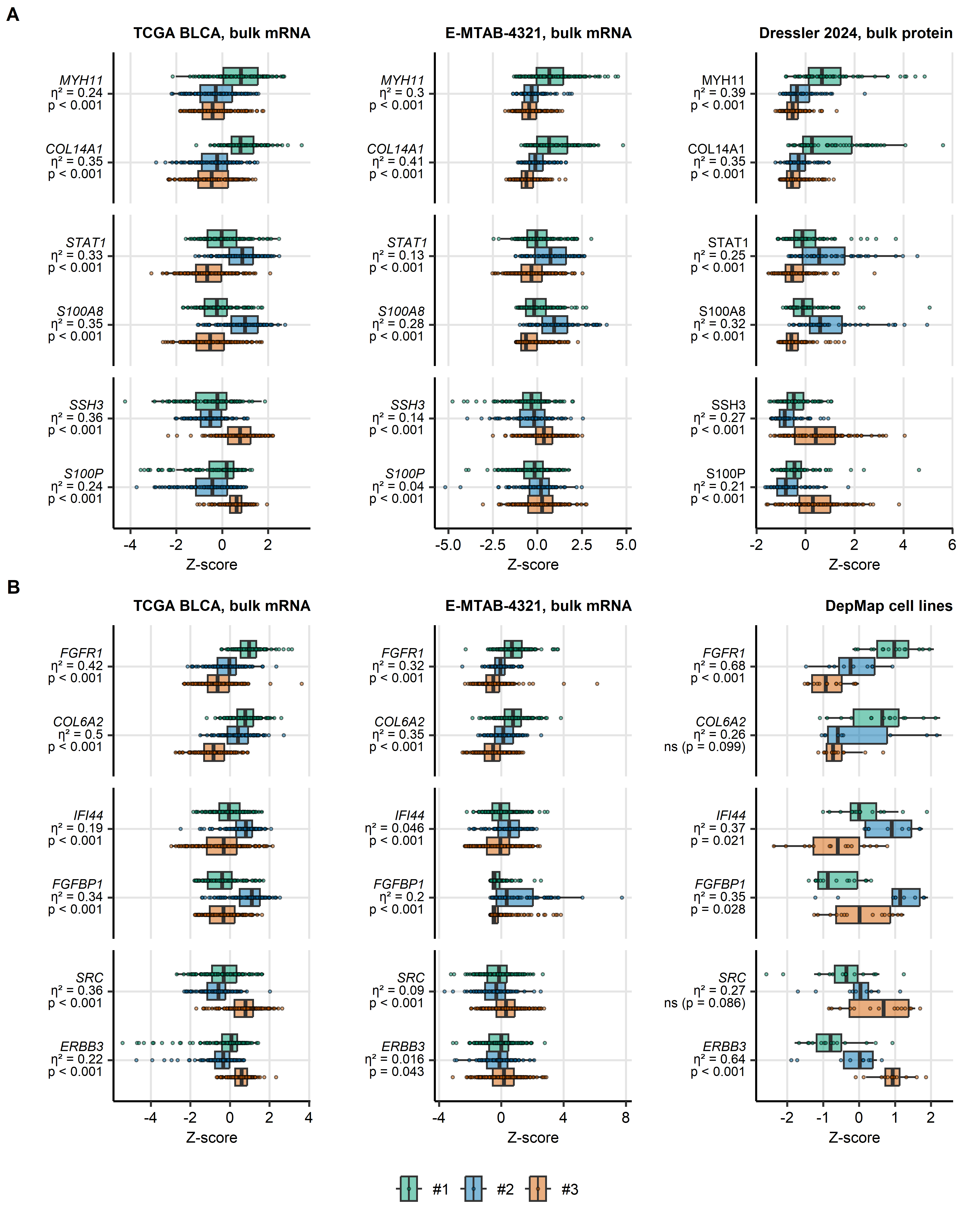


**Supplementary Figure S9. Bladder cancer clusters: selected cluster markers shared by bulk cancer transcriptomic and proteomic collectives, and DepMap urothelial cancer cell lines.**

*Samples in 19 transcriptomic bulk cancer cohorts (TCGA BLCA, IMvigor, GSE13507, GSE32548, GSE48075, GSE48276, GSE83586, GSE86411, GSE87304, GSE120736, GSE124305, GSE128192, GSE128701, GSE128959, GSE198269, GSE203149, E-MTAB-4321, Groeneveld 2024, and BCAN), cancer specimens in Groeneveld 2024, Dressler 2024 and Stroggilos 2020 bulk cancer proteomic collectives, and DepMap urothelial cancer cell lines were assigned to bladder cancer clusters by clustering of metagene scores, k-nearest neighbor and Elastic Net classifiers (Supplementary Tables S8, S14, and S51).*

*(A, B) Expression of representative markers of bladder cancer clusters at mRNA and protein level. Differences in* $log_{2}$ *expression between bladder cancer clusters for representative genes and proteins identified as shared markers of bladder cancer clusters in bulk cancer transcriptome and proteome data sets (A), and shared markers of bladder cancer clusters in bulk cancer transcriptome and DepMap cell lines (B). Median* $log_{2}$ *expression values with interquartile ranges are presented as boxes with whiskers spanning over 150% of the interquartile ranges. Single cancer samples and cell lines are depicted as points. Effect size* $\eta^{2}$ *statistics and false discovery (FDR) corrected p values obtained by one-way ANOVA are displayed in the Y axes. Results for the TCGA BLCA (muscle invasive cancers, transcriptome; #1: n = 120, #2: n = 117, #3: n = 170), E-MTAB-4321 (non-muscle invasive cancers, transcriptome; #1: n = 153, #2: n = 81, #3: n = 242) and Dressler 2024 (non-muscle invasive and invasive cancers, proteome; #1: n = 69, #2: n = 50, #3: n = 123) bulk cancer sample collectives, and DepMap urothelial cell lines are shown.*


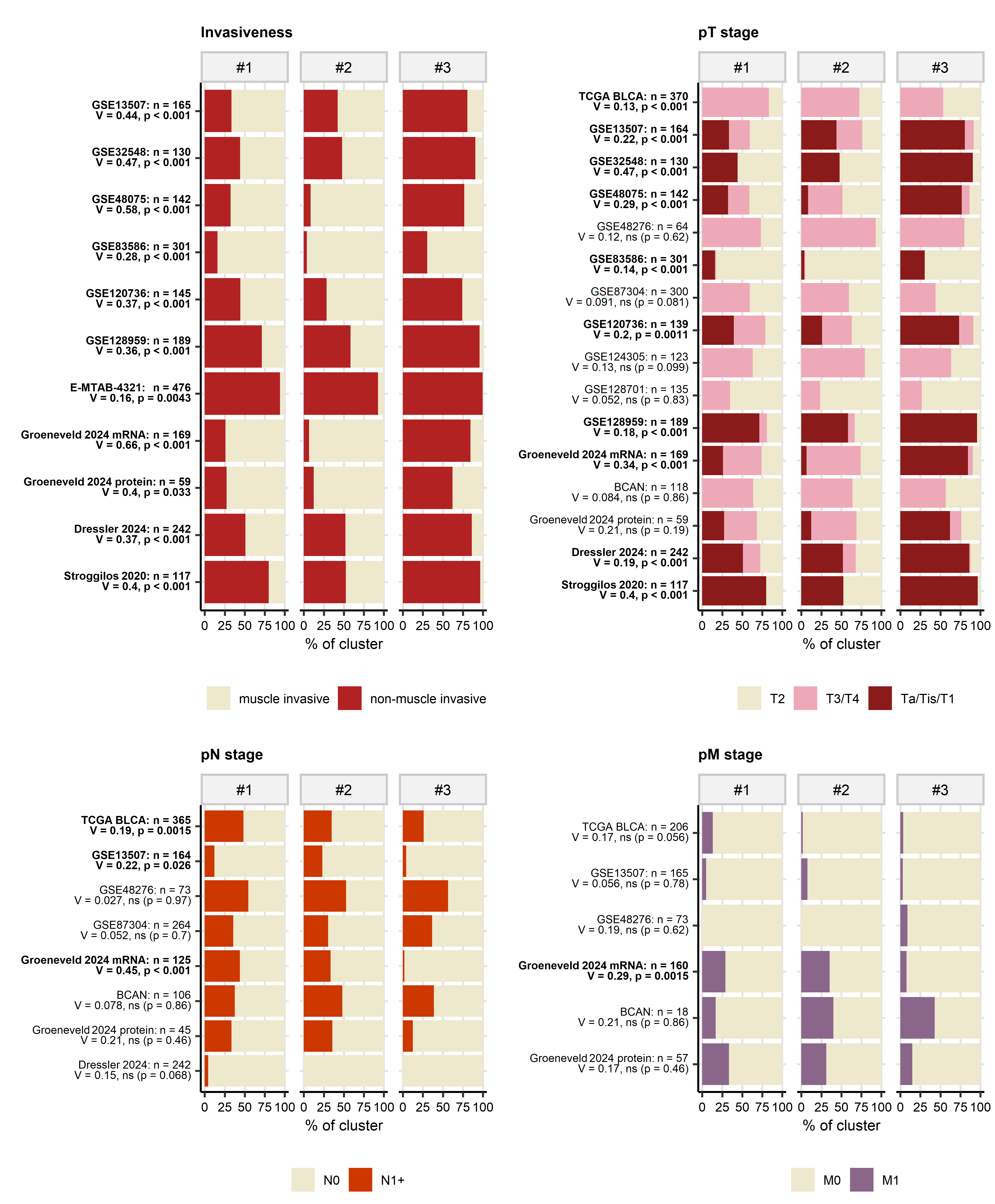


**Supplementary Figure S10. Cancer invasiveness, and pathological tumor, lymph node and metastasis stages in bladder cancer clusters.**

*Differences of rates of cancer invasiveness, and distribution of pathological tumor (pT), node (pN) and metastasis (pM) stage between bladder cancer clusters were investigated by* $\chi^{2}$ *test with Cramer’s V effect size statistic. P values were corrected for multiple testing with the false discovery rate method.* *Frequencies of invasiveness and pathological stages were expressed as percentages of observations in particular bladder cancer clusters and depicted in stack plots. Significant differences between bladder cancer clusters are highlighted by bold labels.* *Full descriptive statistics and testing results for the stating in bladder cancer clusters are presented in Supplementary Table S19.*


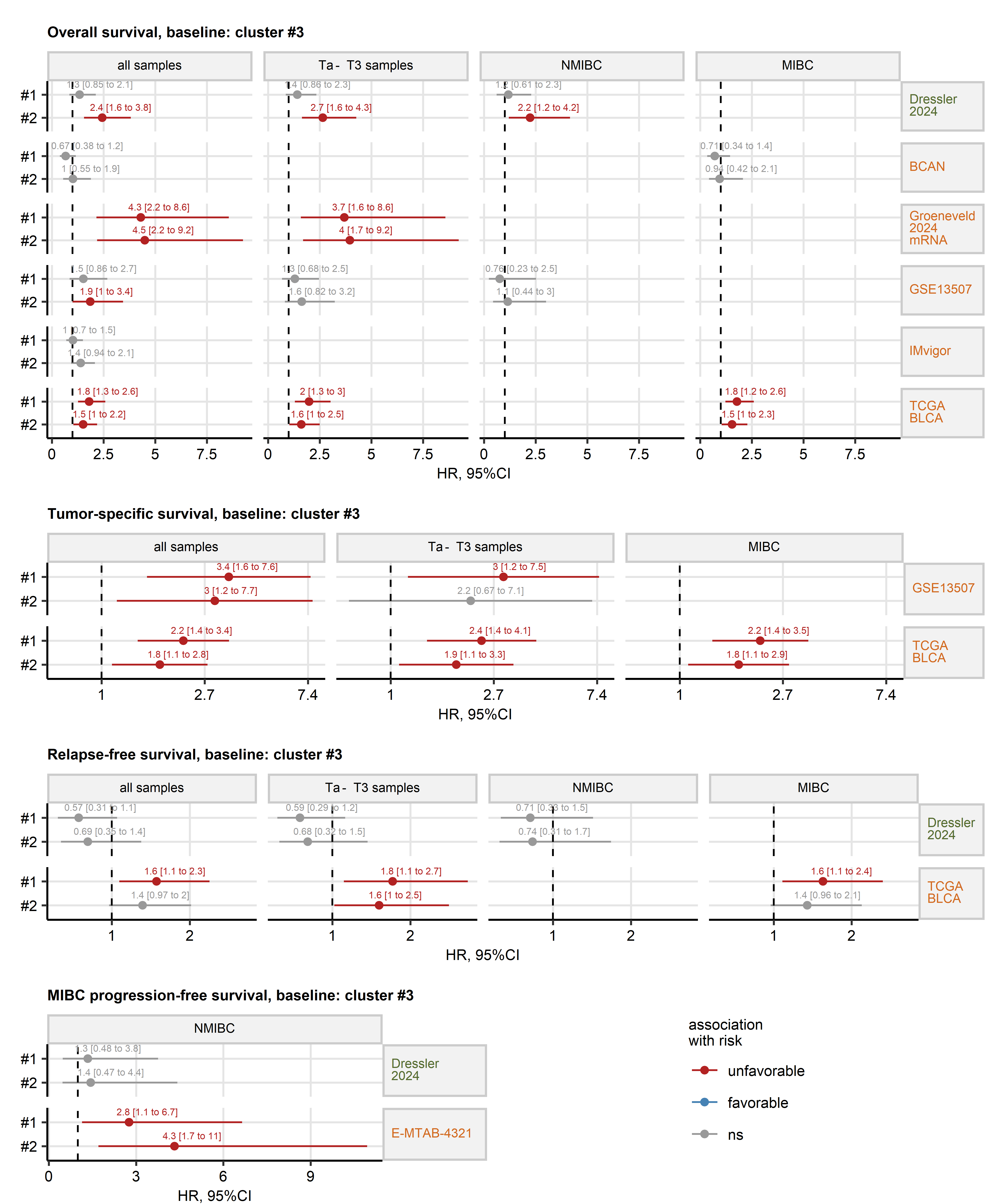
**Supplementary Figure S11. Modeling of overall, tumor-specific, relapse-free, and MIBC progression-free survival in bladder cancer clusters #1 and #2 as compared with cluster #3.**

*Univariable Cox proportional hazard models of overall, tumor-specific, relapse-free, and MIBC progression-free survival with bladder cluster assignment as the sole explanatory factor were constructed for all patients, patients with Ta - T3 tumors, non-muscle invasive bladder cancer (NMIBC) and muscle invasive bladder cancer patients in the TCGA BLCA, IMvigor, GSE13507, Groeneveld 2024, BCAN, Dressler 2024, and E-MTAB-4321 cohorts. Patient subsets with less than 80 cases with complete survival observations and less than 5 events were excluded from the analysis. Cluster #3 served as the model’s baseline.* *Coefficients of the Cox proportional hazard models are expressed as hazard ratios (HR) and presented with their 95% confidence intervals in Forest plots (points and whiskers). Point and whisker color codes for significance and association of the cluster assignment with risk. The points are labeled with their HR estimates. Cohort names are indicated in plot facets; cohort names in green code for bulk proteome data, cohort names in orange represent bulk cancer transcriptome data.* *Numbers of patients and events, and results of Kaplan-Meier analyses overall, tumor-specific, relapse-free, and progression-free survival in bladder cancer clusters are presented in Supplementary Table S20.*


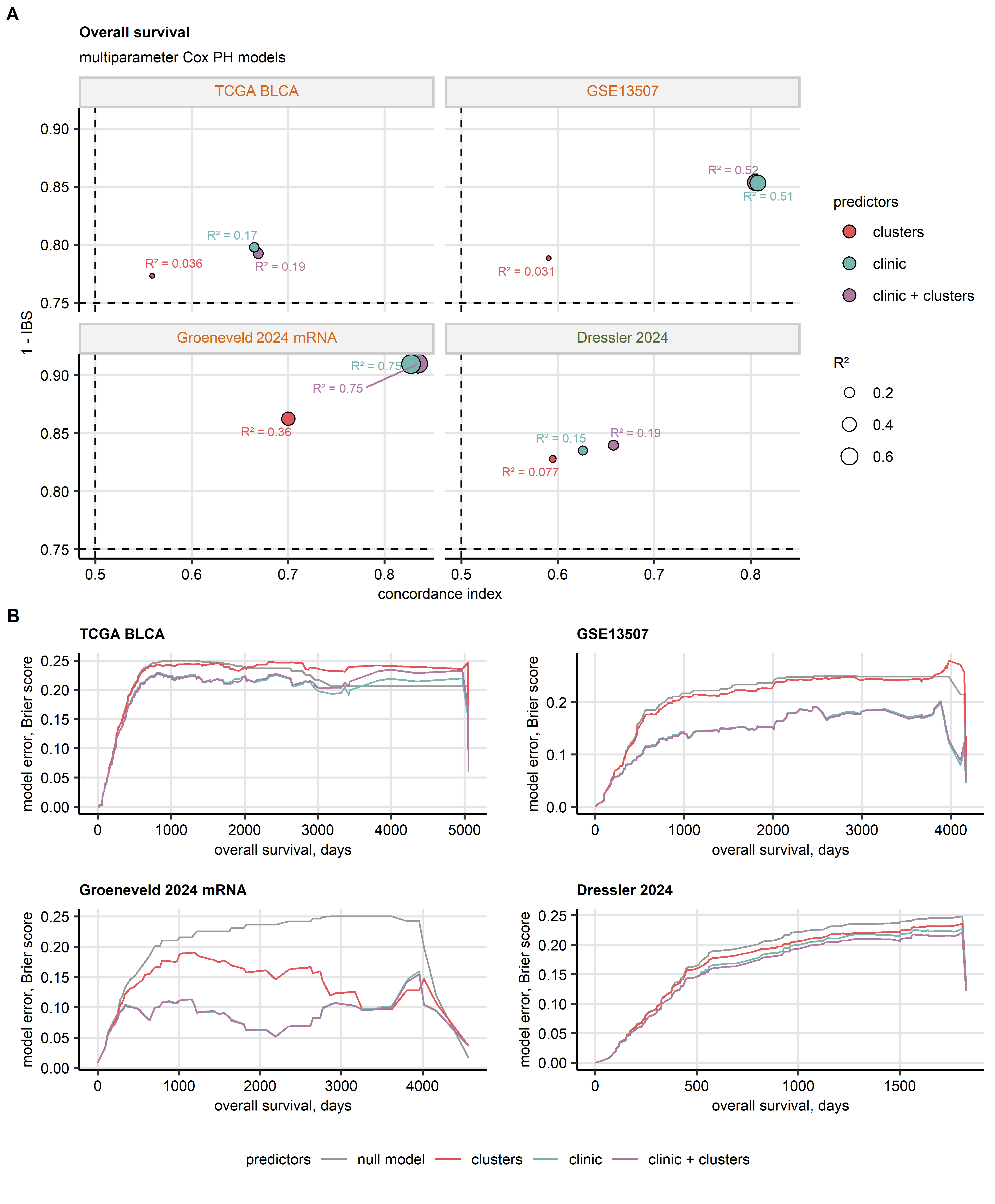


**Supplementary Figure S12. Modeling of overall survival with bladder cancer clusters and clinical predictors: model performance.**

*Uni- and multi-parameter Cox proportional hazard models of overall survival with bladder cancer clusters, clinical predictors, and bladder cancer clusters along with clinical predictors were constructed in the*, *TCGA BLCA (observations: n = 345, events: 152; clinical predictors: age, sex, pathological tumor stage, pathological node stage),* *GSE13507 (observations: n = 163, events: 67; clinical predictors: age, sex, pathological tumor stage, pathological node stage),* *Groeneveld 2024 mRNA (observations: n = 114, events: 43; clinical predictors: age, sex, pathological tumor stage, pathological node stage), and* *Dressler 2024 proteome cohort (observations: n = 242, events: 109; clinical predictors: pathological tumor stage).*

*(A) Performance of the Cox proportional hazard models. Accuracy of the predicted risk and actual was quantified by Harrell’s concordance index, model confidence was metered by integrated Brier score (IBS; low values are expected for a highly confident model), explained survival variance was measured by* $R^{2}$ *statistic. The performance statistics are presented in dot plots; point color codes for the model’s predictor set, point sizes corresponds to* $R^{2}$ *values.*

*(B) Errors of the models expressed as Brier scores (squared differences between the predicted death risk and 0/1-coded death status) during the follow-up.*


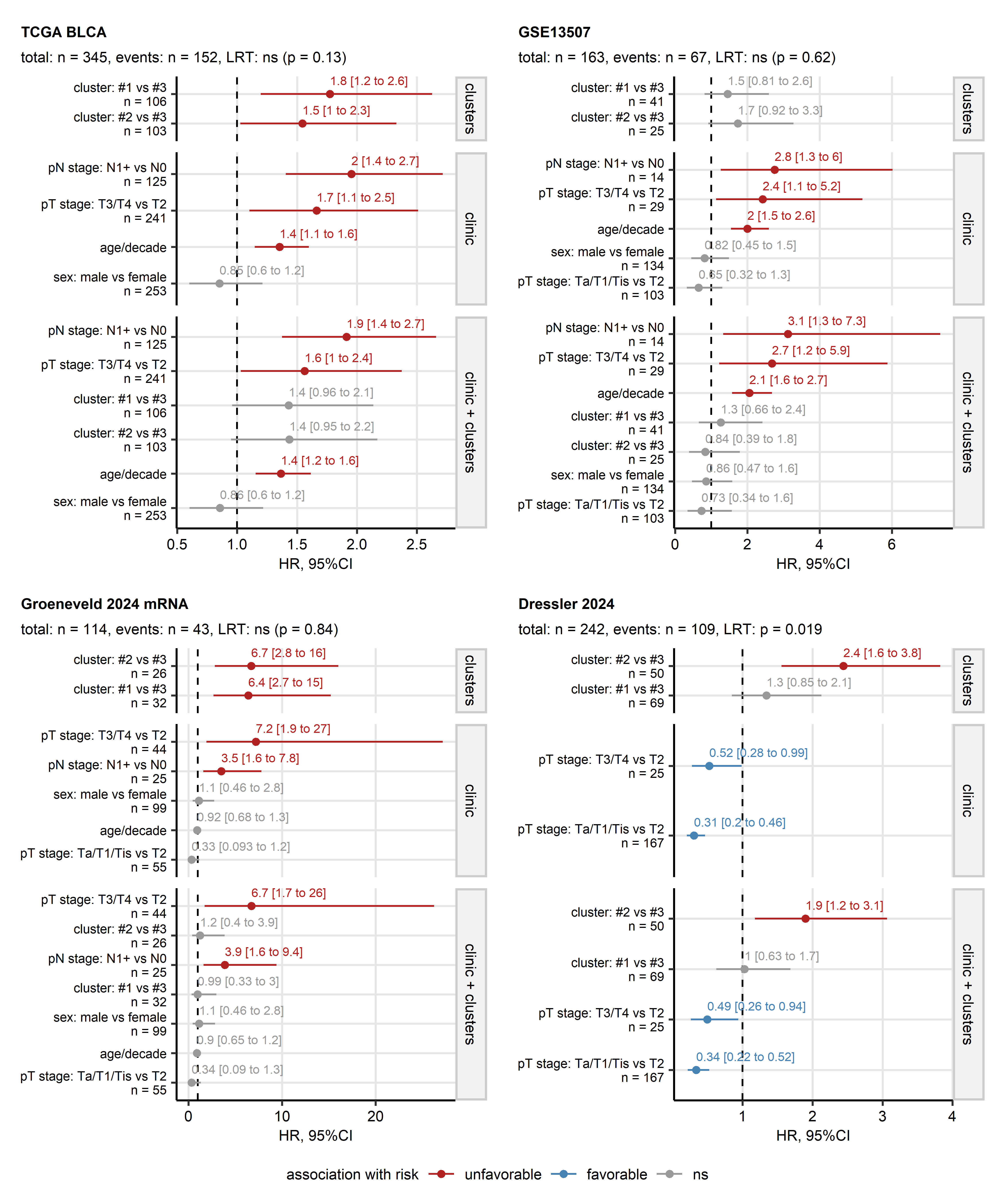


**Supplementary Figure S13. Modeling of overall survival with bladder cancer clusters and clinical predictors: model inference.**

*Uni- and multi-parameter Cox proportional hazard models of overall survival with bladder cancer clusters, clinical predictors, and bladder cancer clusters along with clinical predictors were constructed in the*, *TCGA BLCA (clinical predictors: age, sex, pathological tumor stage, pathological node stage),* *GSE13507 (clinical predictors: age, sex, pathological tumor stage, pathological node stage),* *Groeneveld 2024 mRNA (clinical predictors: age, sex, pathological tumor stage, pathological node stage), and* *Dressler 2024 proteome cohort (clinical predictors: pathological tumor stage).* *Significance of the bladder cancer term in the models adjusted for clinical predictors was assessed by likelihood-ratio test (LRT).*

*Risk estimates for groups of patients defined by bladder cancer clusters and clinical predictors expressed as hazard ratios (HR) with 95% confidence intervals (95%CI) are shown in Forest plots. Symbol colors codes for significance and association with death risk. The symbols are labeled with HR estimates with lower and upper bounds of 95%CI. Total numbers of observations and deaths, and p values in the LRT tests are displayed in the plot captions. Modeling baselines and numbers of cases in the risk groups are indicated in the Y axes.*


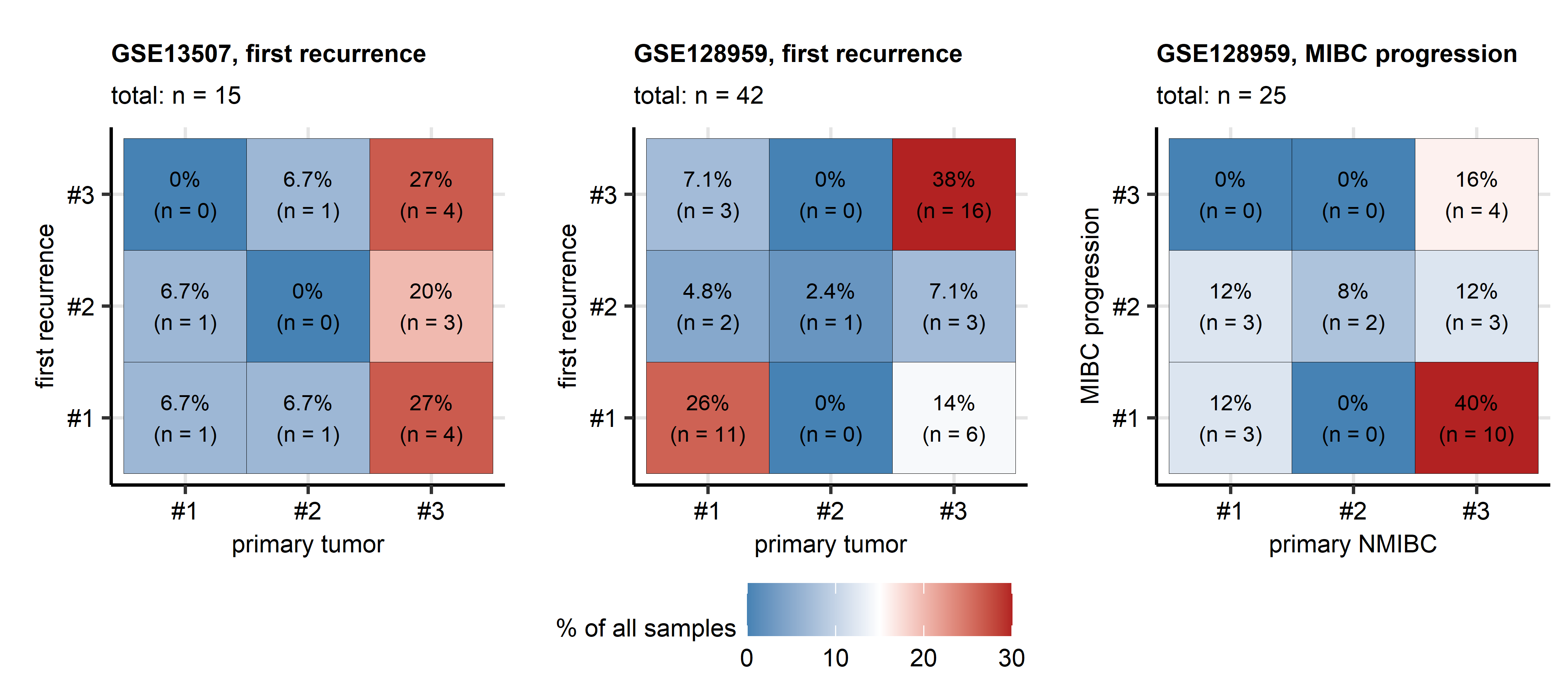


**Supplementary Figure S14. Evolution of bladder cancer clusters during recurrence and NMIBC - MIBC progression of urothelial malignancies.**

*Bulk mRNA sequencing results for patient-matched primary and recurrent cancer, and primary NMIBC (non-muscle invasive bladder cancer) and MIBC (muscle invasive bladder cancer) progression specimens were available for the GSE13507 and GSE128959 cohorts.* *Changes of bladder cluster assignment between primary cancers and first recurrence specimens, and between primary NMIBC and MIBC progression samples were visualized as heat maps of transition matrices.* *Total numbers of samples are displayed in the plot captions.*


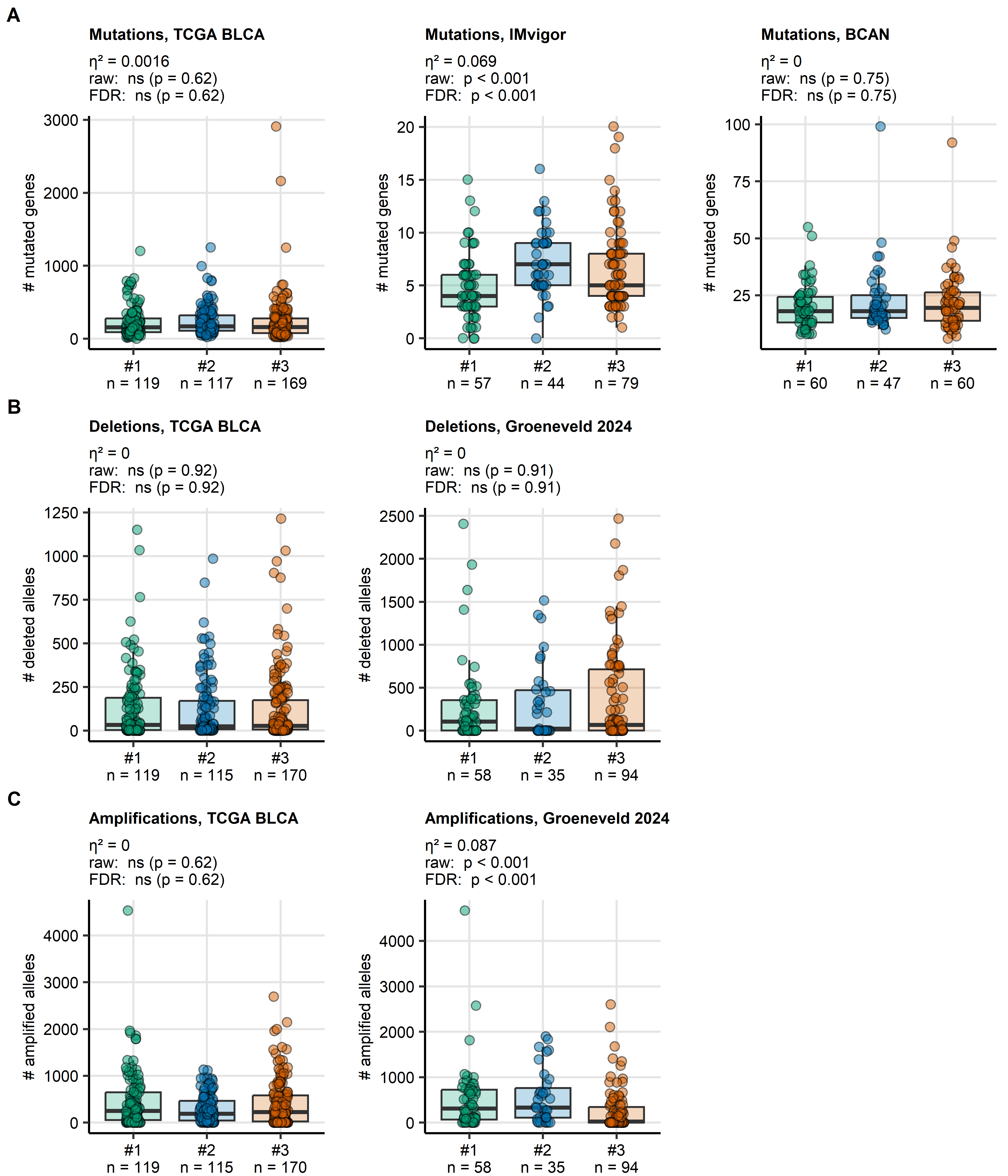


**Supplementary Figure S15. Numbers of mutated, deleted, and amplified genes in bladder cancer clusters.**

*Differences between bladder cancer clusters in numbers of genes with somatic mutations, amplifications and deletions (at least one gene copy) were assessed by Kruskal-Wallis test with* $\eta^{2}$ *effect size statistic. P values were corrected for multiple testing with the false discovery rate (FDR) method. Median alteration numbers with interquartile ranges are depicted as boxes with whiskers spanning over 150% of the interquartile ranges. Single cancer samples are visualized as points. Effect sizes, raw and FDR-corrected effect sizes are displayed in the plot captions. Numbers of cancer samples in bladder cancer clusters are indicated in the X axes. Descriptive statistics and full testing results are presented in Supplementary Table S21.*


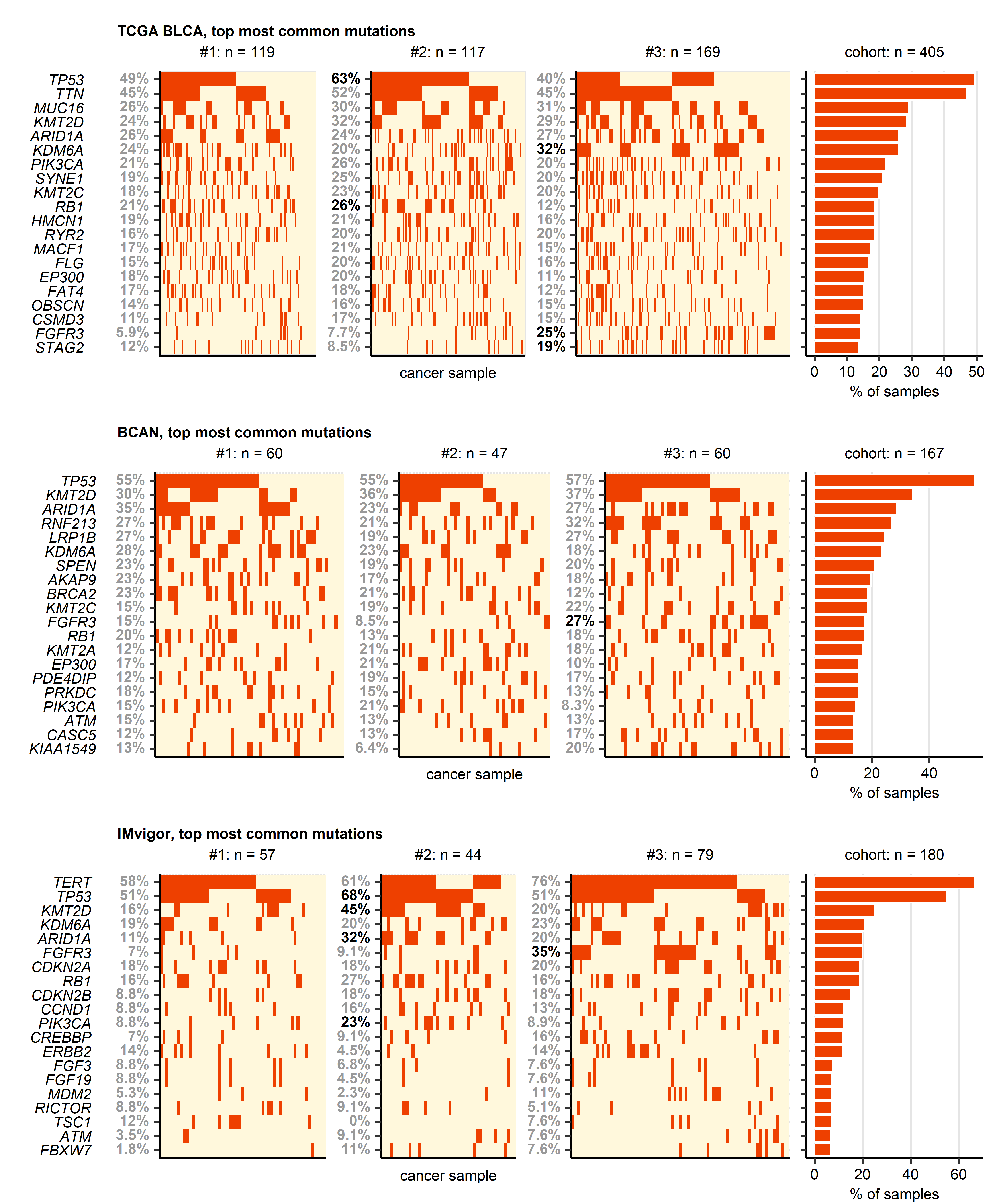


**Supplementary Figure S16. Overall rates of somatic mutations in the most frequently mutated genes in the TCGA BLCA, IMvigor, and BCAN cohorts.**

*Enrichment of somatic mutations in bladder cancer clusters was investigated by permutation testing (perTest tool, perich package). Enrichment in a cluster was measured by enrichment score (ES), i.e. ratio of the observed mutation frequency to the mean expected mutation frequency adjusted for random overall mutation rate obtained in n = 10000 algorithm iterations. Significant enrichment was assumed for ES >= 1.2 and raw p < 0.05. Because the p values depend on the iteration number, no multiple testing correction was applied.* *Somatic mutations in top 20 genes with the highest mutation rates in the TCGA BLCA, BCAN, and IMvigor cohorts are visualized in oncoplots. Each plot tile represents a single cancer sample. Color tile codes for presence of at least one somatic mutation in a gene. Percentages of samples with mutations in particular bladder cancer clusters are indicated in the Y axes. Significant enrichment in a bladder cancer cluster is highlighted with bold font. The bar plots display percentages of samples with mutations in a given gene in the entire cohort.* *Full results of mutation analysis results by permutation testing are summarized in Supplementary Table S22.*


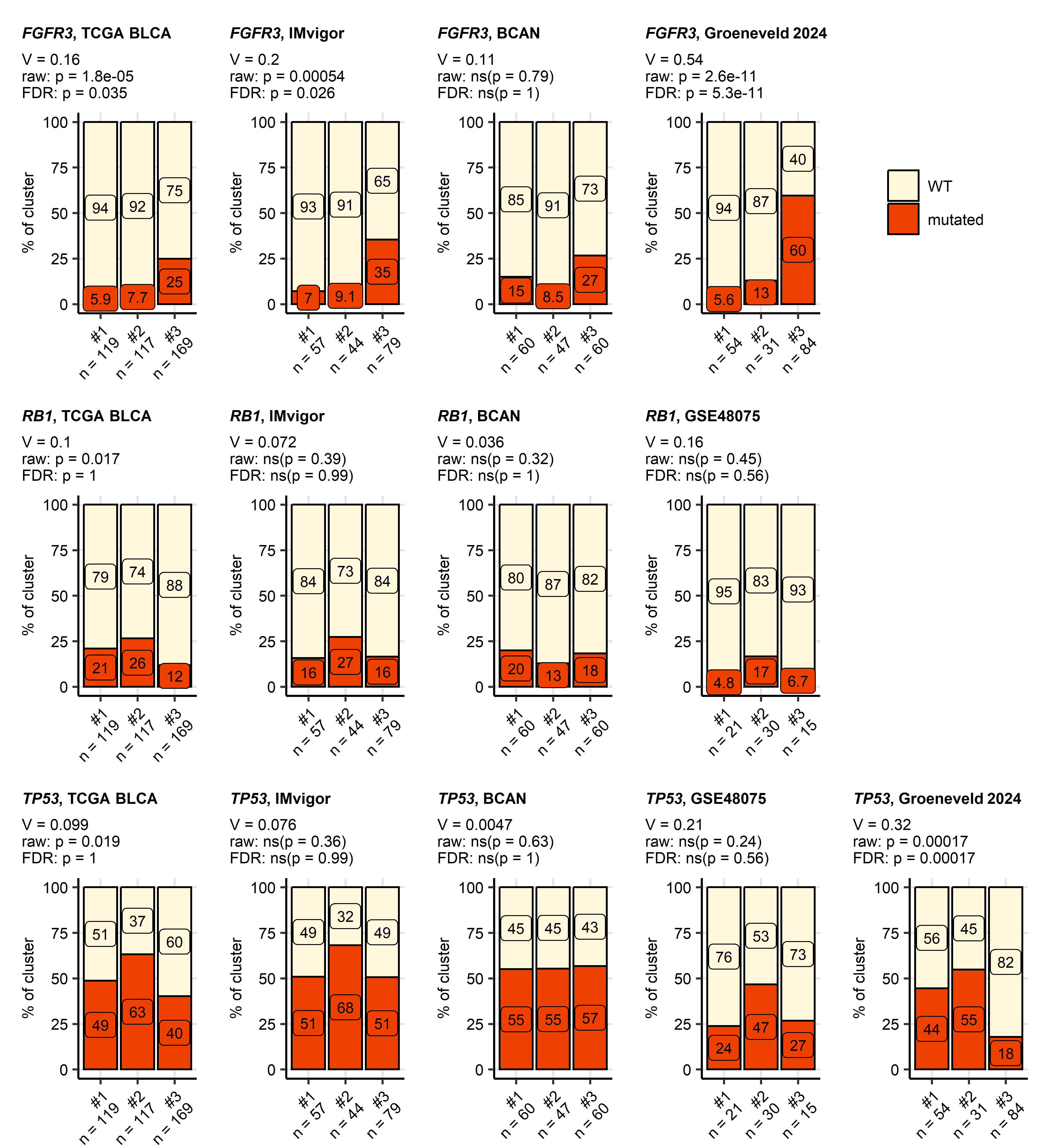


**Supplementary Figure S17. Differences in somatic mutation rates of *FGFR3*, *RB1*, and *TP53* genes between bladder cancer clusters.**

*Differences in frequency of somatic mutations in FGFR3, RB1, and TP53 genes between bladder cancer clusters in the TCGA BLCA, IMvigor, BCAN, GSE48075, and Groeneveld 2024 cohorts were assessed by* $\chi^{2}$ *test with Cramer’s V effect size statistic. P values were adjusted for multiple testing with the false discovery rate method (FDR). Percentages of samples with mutations in the genes of interest in bladder cancer clusters are presented in stack plots. Effect sizes, raw unadjusted p values, and FDR-adjusted p values are displayed in the plot captions. Numbers of specimens in bladder cancer clusters are indicated in the X axes.*


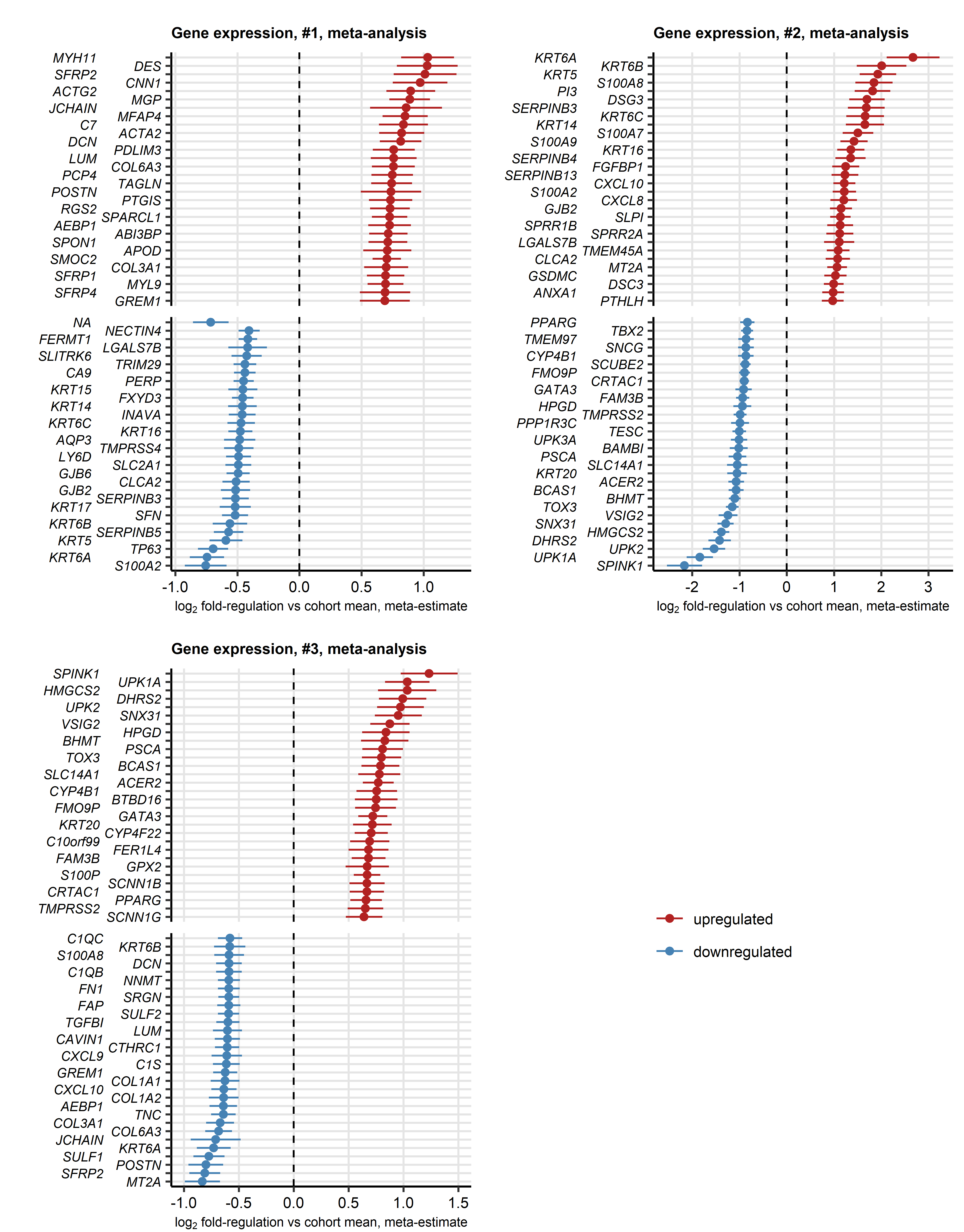


**Supplementary Figure S18. Top differentially regulated genes in bladder cancer clusters.**

$log_{2}$*-transformed gene expression levels (no ComBat adjustment) were compared between bladder cancer clusters of transcriptomic bulk cancer cohorts by one-way ANOVA with* $\eta^{2}$ *effect size statistic. Differences between mean* $log_{2}$ *expression level in the clusters and the respective cohort means were evaluated by one-sample T test. P values were corrected for multiple comparisons with the false discovery rate (FDR) method. Significantly differentially regulated genes were defined by ANOVA pFDR < 0.05,* $\eta^{2}\geq$ *0.06, and T test pFDR < 0.05.* *For genes found to be significantly regulated in at least 10 out of 19 analyzed cohorts (TCGA BLCA, IMvigor, GSE13507, GSE32548, GSE48075, GSE48276, GSE83586, GSE86411, GSE87304, GSE120736, GSE124305, GSE128192, GSE128701, GSE128959, GSE198269, GSE203149, E-MTAB-4321, Groeneveld 2024, and BCAN) meta-estimates of* $log_{2}$ *fold-regulation estimates in particular bladder cancer clusters as compared with cohort means were computed by the DerSimonian-Lair inverse variance algorithm.* *These meta estimates for the top strongest up- and downregulated genes are presented with 95% confidence intervals in Forest plots.* *Full results of differential gene expression analysis and meta-analysis for bulk cancer transcriptome cohorts are summarized in Supplementary Table S23 and S24.*


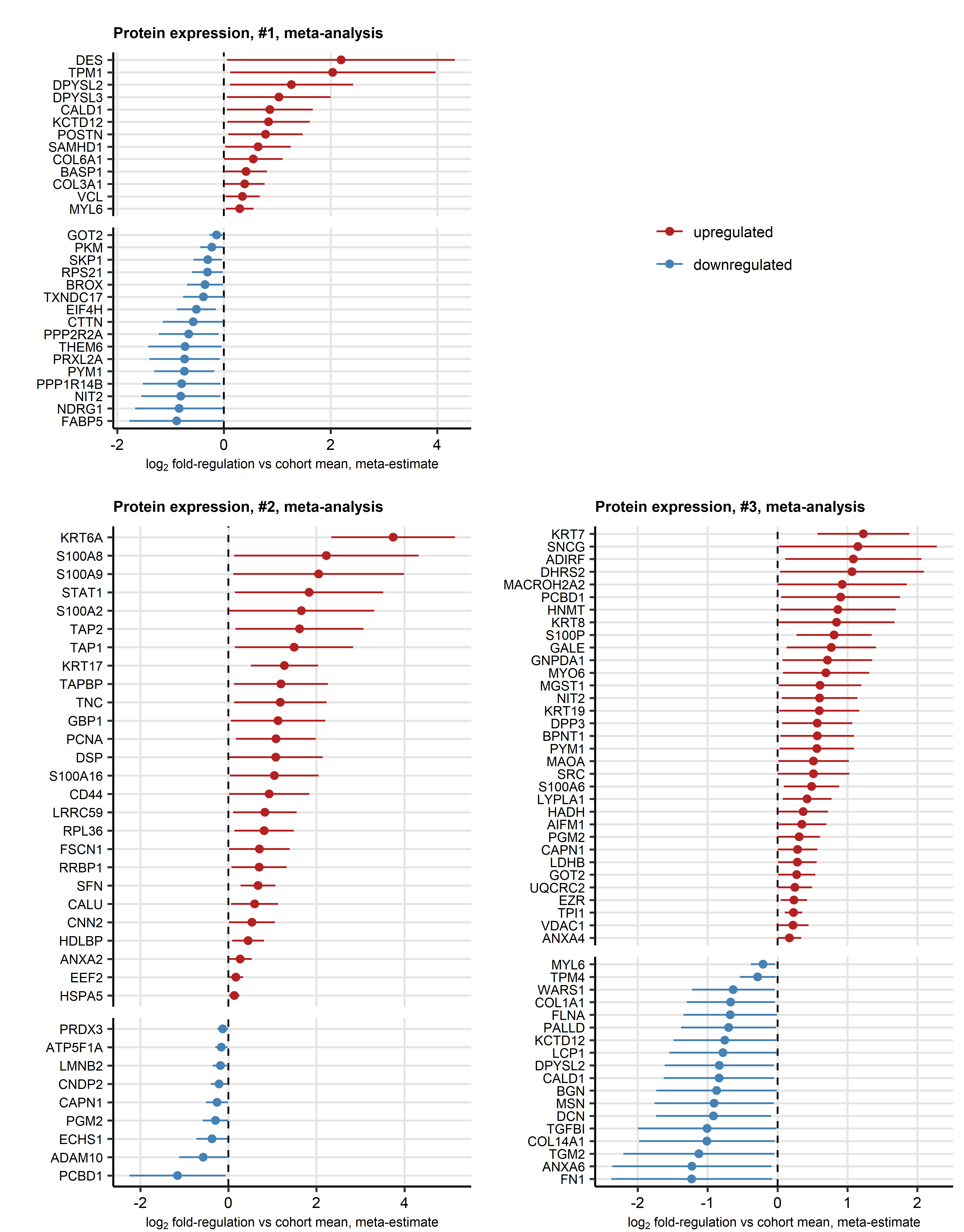


**Supplementary Figure S19. Top differentially regulated proteins in bladder cancer clusters.**

$log_{2}$*-transformed protein expression levels (no ComBat adjustment) in the Groeneveld 2024, Dressler 2024, and Stroggilos 2020 proteomic cohorts were compared between bladder cancer clusters by one-way ANOVA with* $\eta^{2}$ *effect size statistic. Differences between mean* $log_{2}$ *expression level in the clusters and the respective cohort means were evaluated by one-sample T test. P values were corrected for multiple comparisons with the false discovery rate (FDR) method. Significantly differentially regulated proteins were defined by ANOVA pFDR < 0.05,* $\eta^{2}\geq$ *0.06, and T test pFDR < 0.05.* *For proteins found to be significantly regulated in at least two proteomic cohorts, meta-estimates of* $log_{2}$ *fold-regulation estimates in particular bladder cancer clusters as compared with cohort means were computed by the DerSimonian-Lair inverse variance algorithm.* *These meta estimates for the top strongest up- and downregulated proteins are presented with 95% confidence intervals in Forest plots.* *Full results of differential protein expression analysis and meta-analysis for bulk cancer proteome cohorts are summarized in Supplementary Table S25 and S26.*


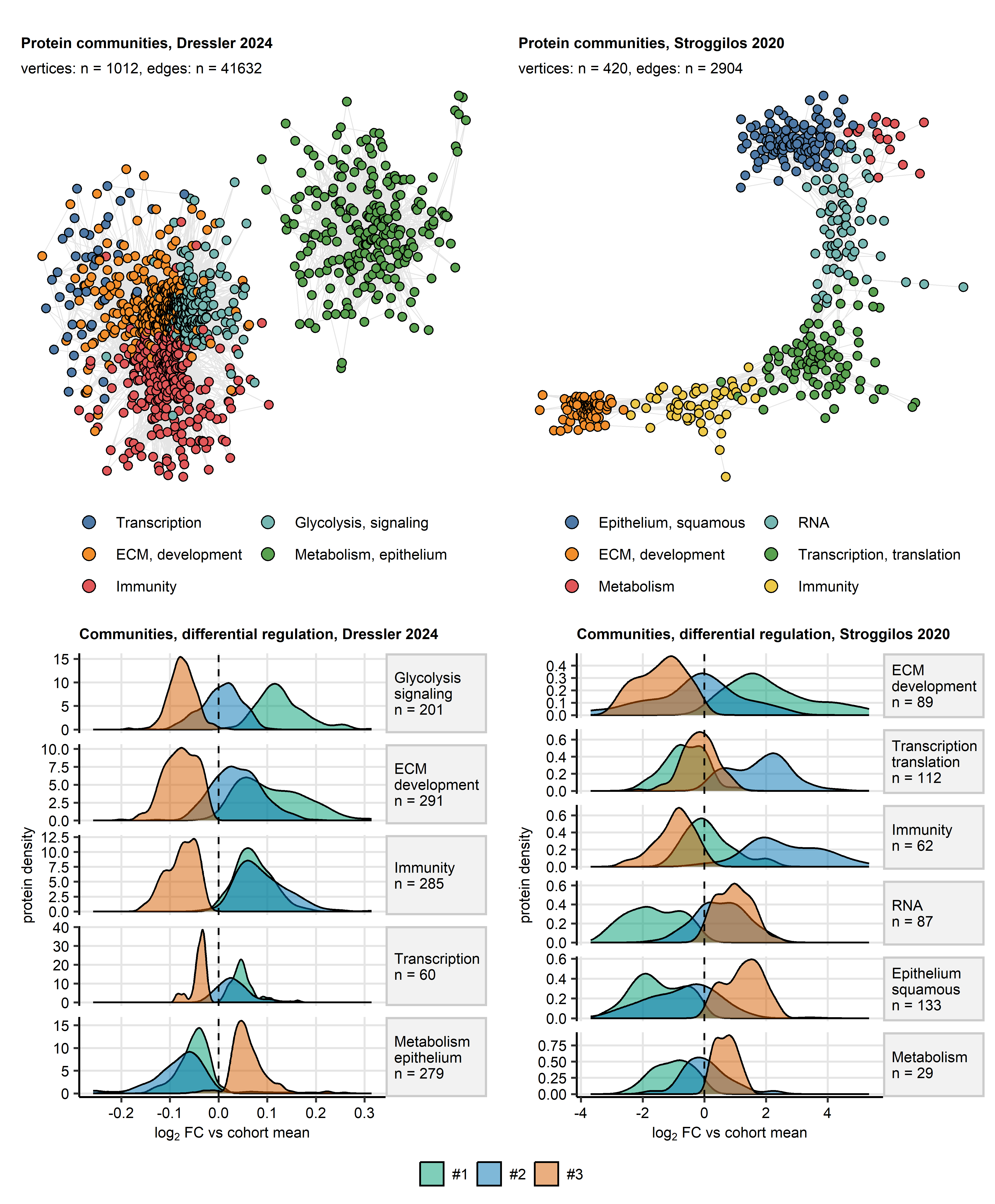


**Supplementary Figure S20. Co-regulation networks of differentially expressed proteins in bladder cancer clusters of bulk cancer proteomes.**

*Co-regulation networks of proteins differentially expressed in bladder cancer clusters as compared with the cohort means in the bulk cancer proteome cohorts (Groeneveld 2024: n = 63, Dressler 2024: n = 242, and Stroggilos 2020: n = 117).* $log_{2}$*-transformed protein expression levels (no ComBat adjustment) were compared between bladder cancer clusters by one-way ANOVA with* $\eta^{2}$ *effect size statistic. Differences between mean* $log_{2}$ *expression levels in the clusters and the respective cohort means were evaluated by one-sample T test. P values were corrected for multiple comparisons with the false discovery rate (FDR) method. Significantly differentially regulated genes and proteins were defined by ANOVA pFDR < 0.05,* $\eta^{2}\geq$ *0.06, and T test pFDR < 0.05.* *Co-expression patterns of the differentially regulated proteins were investigated by network analysis. The networks’ edges were defined by pairwise correlations of protein levels with Spearman’s* $\rho\geq$ *0.5 and weighted by the corresponding* $\rho$ *values. Isolated vertices, i.e. proteins without correlation partners were removed.* *Communities of co-regulated proteins were identified by the Leiden algorithm and named after their key biological processes revealed by biological process gene ontology enrichment (Supplementary Tables S29 and S30).*

*The co-expression networks were visualized as force-directed graphs with the Fruchterman–Reingold algorithm for the Dressler 2024 and Stroggilos 2020 collectives. Single genes are represented by points, correlations between genes with* $\rho\geq$ *0.5 are depicted as lines. Point color codes for the community assignment.* *Density of estimates of* $log_{2}$ *fold changes of expression in the clusters as compared with the cohort mean for members of protein communities are presented in density plots; numbers of proteins in the communities are indicated in the plot facets.*


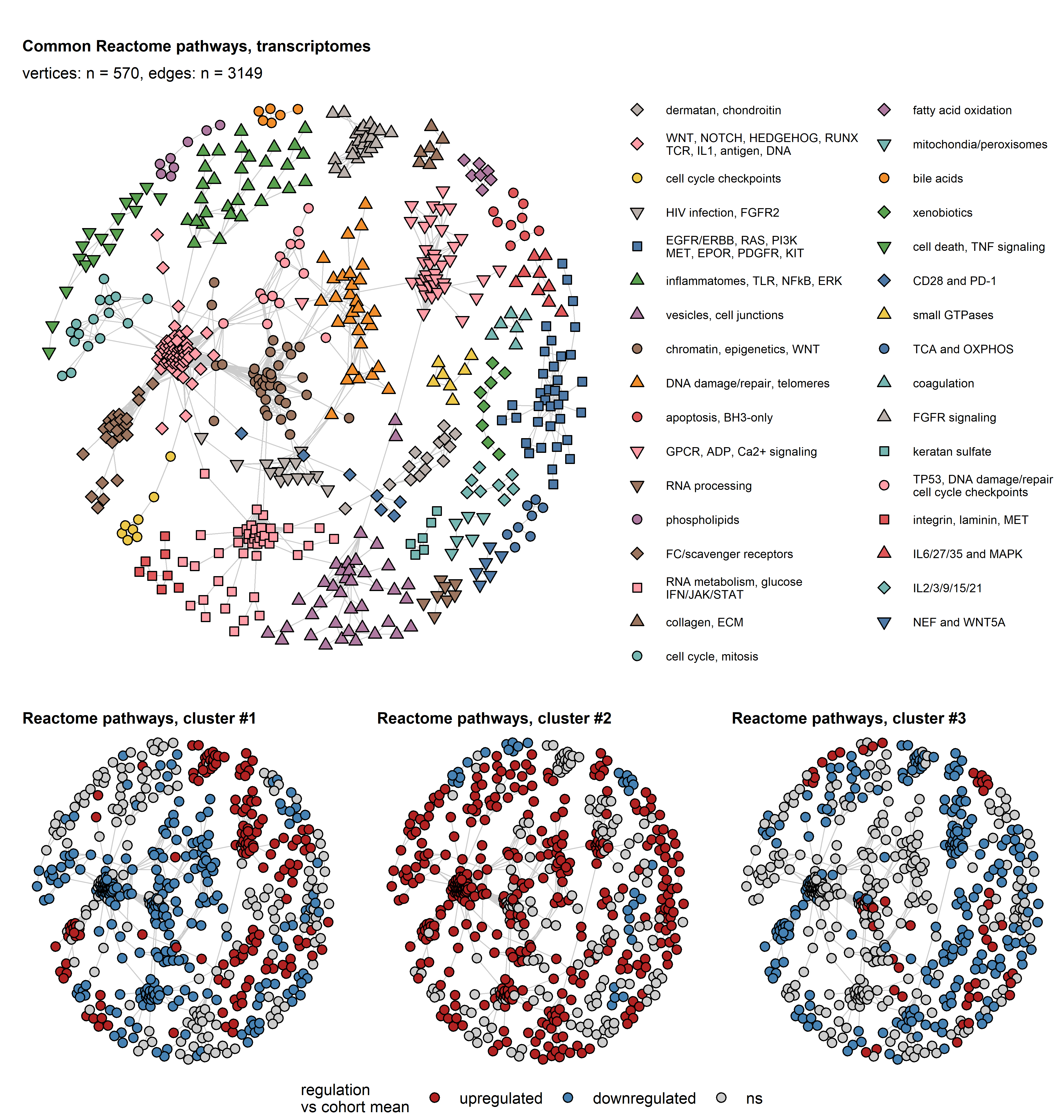


**Supplementary Figure S21. Gene set variation analysis of Reactome Pathways in bladder cancer clusters of bulk cancer transcriptomes.**

*Single sample gene set enrichment analysis (ssGSEA) scores of Reactome Pathway gene signatures were compared between bladder cancer clusters in transcriptomic bulk cancer cohorts by one-way ANOVA with* $\eta^{2}$ *effect size statistic. Differences between mean ssGSEA scores in the clusters and the respective cohort means were evaluated by one-sample T test. P values were corrected for multiple comparisons with the false discovery rate (FDR) method. Significantly differentially regulated Reactome Pathway signatures were defined by ANOVA pFDR < 0.05,* $\eta^{2}\geq$ *0.14, and T test pFDR < 0.05 (Supplementary Table S31).* *Reactome Pathway gene signatures found to be significantly regulated in at least 10 out of 19 analyzed cohorts (TCGA BLCA, IMvigor, GSE13507, GSE32548, GSE48075, GSE48276, GSE83586, GSE86411, GSE87304, GSE120736, GSE124305, GSE128192, GSE128701, GSE128959, GSE198269, GSE203149, E-MTAB-4321, Groeneveld 2024, and BCAN) were subjected to a network analysis.* *For these common differentially regulated signatures, pairwise Jaccard similarity coefficients J were computed in respect to the overlapping signature members. Networks were constructed for signatures associated with each other with with J* $\geq$ *0.3.* *Communities of overlapping signatures were identified by the Leiden algorithm and named after their key biological processes.* *Network structures were visualized as force-directed graphs with the Fruchterman–Reingold algorithm. Single signatures are represented by points, associations between signatures with J* $\geq$ *0.3 are depicted as edges. Point colors and shapes code for community assignment, or regulation status in a particular bladder cancer cluster as compared with the cohort average shared by at least 10 cohorts. Numbers of vertices (Reactome Pathways) and edges (associations between the Reactome Pathways) are displayed in the caption of the upper network plot. Assignment of the signatures to communities is summarized in Supplementary Table S32.*

*Ag: antigen; TCR: T cell receptor; ECM: extracellular matrix; TCA: tri-carboxylic acid cycle/Krebs cycle; OXPHOS: oxidative phosphorylation.*


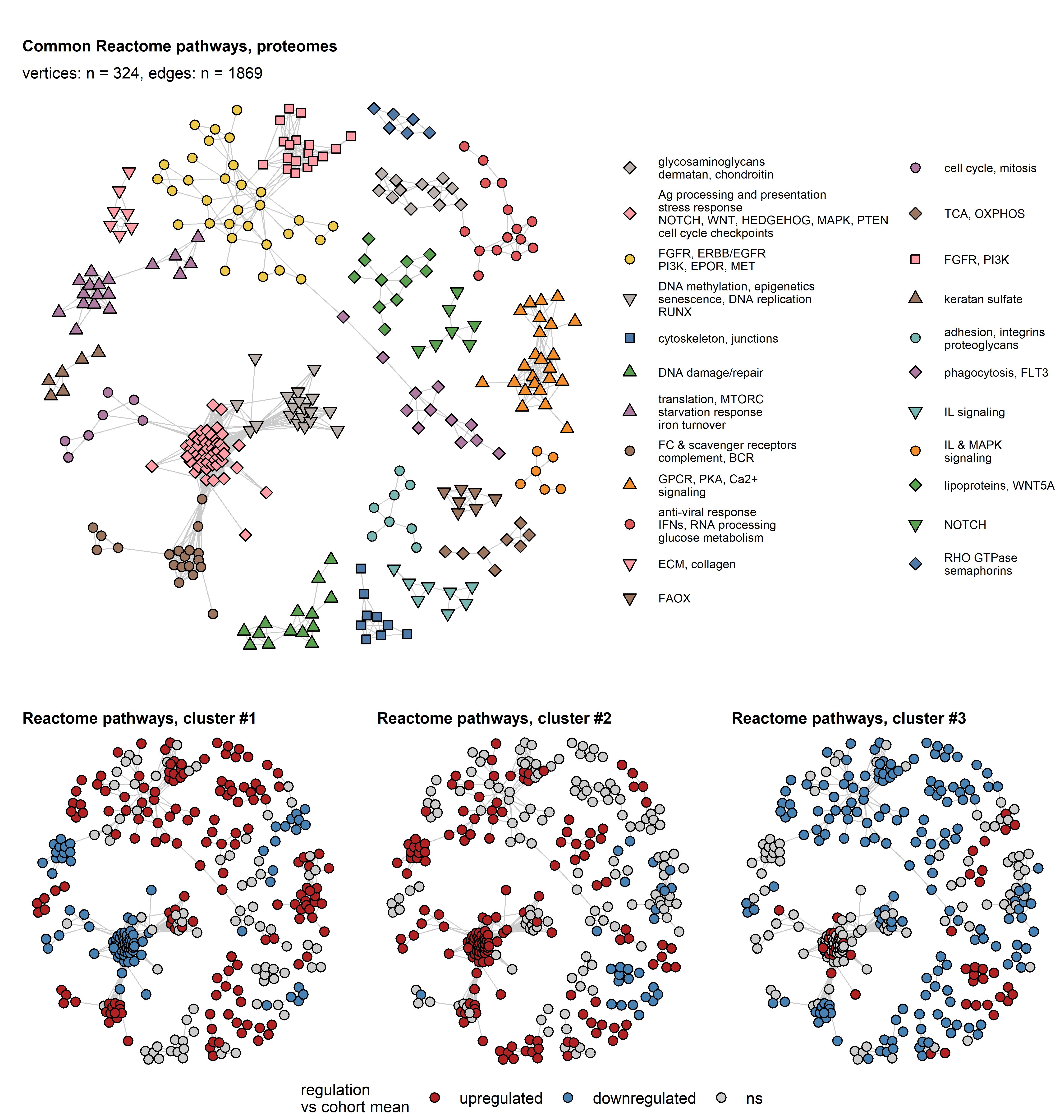


**Supplementary Figure S22. Protein set variation analysis of Reactome Pathways in bladder cancer clusters of bulk cancer proteomes.**

*Single sample gene set enrichment analysis (ssGSEA) scores of Reactome Pathway protein signatures were compared between bladder cancer clusters in proteomic bulk cancer cohorts by one-way ANOVA with* $\eta^{2}$ *effect size statistic. Differences between mean ssGSEA scores in the clusters and the respective cohort means were evaluated by one-sample T test. P values were corrected for multiple comparisons with the false discovery rate (FDR) method. Significantly differentially regulated Reactome Pathway signatures were defined by ANOVA pFDR < 0.05,* $\eta^{2}\geq$ *0.14, and T test pFDR < 0.05 (Supplementary Table S33).* *Reactome Pathway protein signatures found to be significantly regulated in at least 2 of the Groeneveld 2024, Dressler 2024, and Stroggilos 2020 cohorts were subjected to a network analysis.* *For these common differentially regulated signatures, pairwise Jaccard similarity coefficients J were computed in respect to the overlapping signature members. Networks were constructed for signatures associated with each other with with J* $\geq$ *0.3.* *Communities of overlapping signatures were identified by the Leiden algorithm and named after their key biological processes.* *Network structures were visualized as force-directed graphs with the Fruchterman–Reingold algorithm. Single signatures are represented by points, associations between signatures with J* $\geq$ *0.3 are depicted as edges. Point colors and shapes code for community assignment, or regulation status in a particular bladder cancer cluster as compared with the cohort average shared by at least 10 cohorts. Numbers of vertices (Reactome Pathways) and edges (associations between the Reactome Pathways) are displayed in the caption of the upper network plot. Assignment of the signatures to communities is summarized in Supplementary Table S34.*

*Ag: antigen; BCR: B cell receptor; GPCR: G protein-coupled receptors; IFNs: interferons; ECM: extracellular matrix; FAOX: fatty acid oxidation; TCA: tri-carboxylic acid cycle/Krebs cycle; OXPHOS: oxidative phosphorylation; IL: interleukin.*


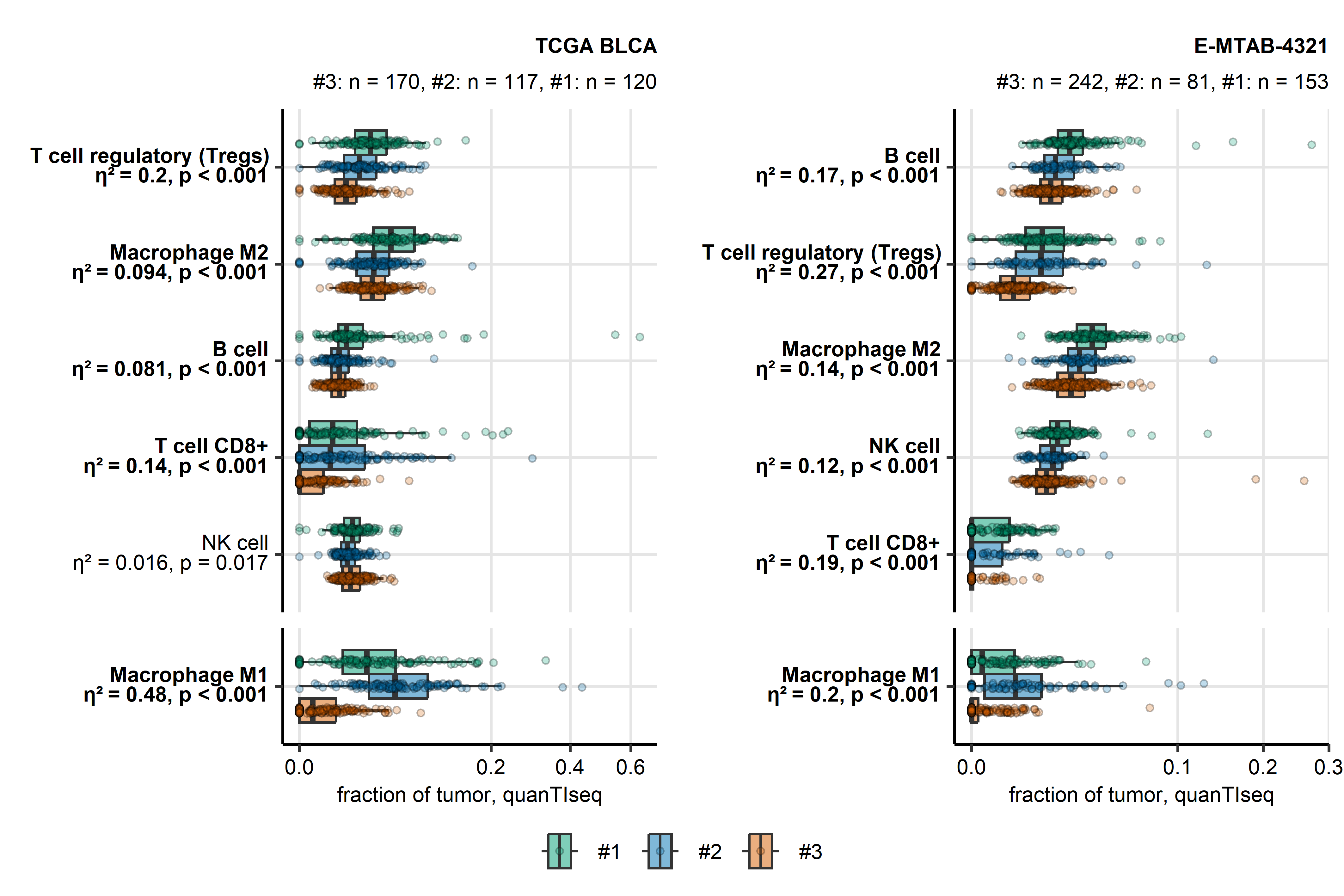


**Supplementary Figure S23. Fractions of non-malignant tumor-infiltrating cells in bladder cancer clusters predicted by QuanTIseq immunedeconvolution**

*Fractions of non-malignant cells in bulk cancer samples were estimated by QuanTIseq immunedeconvolution of bulk cancer transcriptomes. The predicted infiltrating cell fractions were compared between bladder cancer clusters by Kruskal-Wallis test with* $\eta^{2}$ *effect size statistic. P values were corrected for multiple testing with the false discovery rate (FDR). Biologically relevant differences were considered for pFDR < 0.05 and* $\eta^{2}\geq$ *0.06, which translates to significant effects of moderate-to-strong size (Supplementary Table S35).* *Fractions of cell populations found to differ between bladder cancer clusters in at least 10 out of 18 analyzed cohorts (TCGA BLCA, IMvigor, GSE13507, GSE32548, GSE48075, GSE48276, GSE83586, GSE86411, GSE87304, GSE120736, GSE124305, GSE128192, GSE128701, GSE128959, GSE198269, GSE203149, E-MTAB-4321, and Groeneveld 2024) are shown for the TCGA cohort representative for muscle invasive bladder cancer and the E-MTAB 4321 collective representative of non-muscle invasive bladder cancers. Median infiltration levels with interquartile ranges are presented as boxes with whiskers spanning over 150% of the interquartile ranges. Single cancer samples are visualized as points. Numbers of cancer samples in bladder cancer clusters are displayed in the plot captions. Effect sizes and p values are indicated in the Y axis. Biologically relevant differences are highlighted with bold font.*


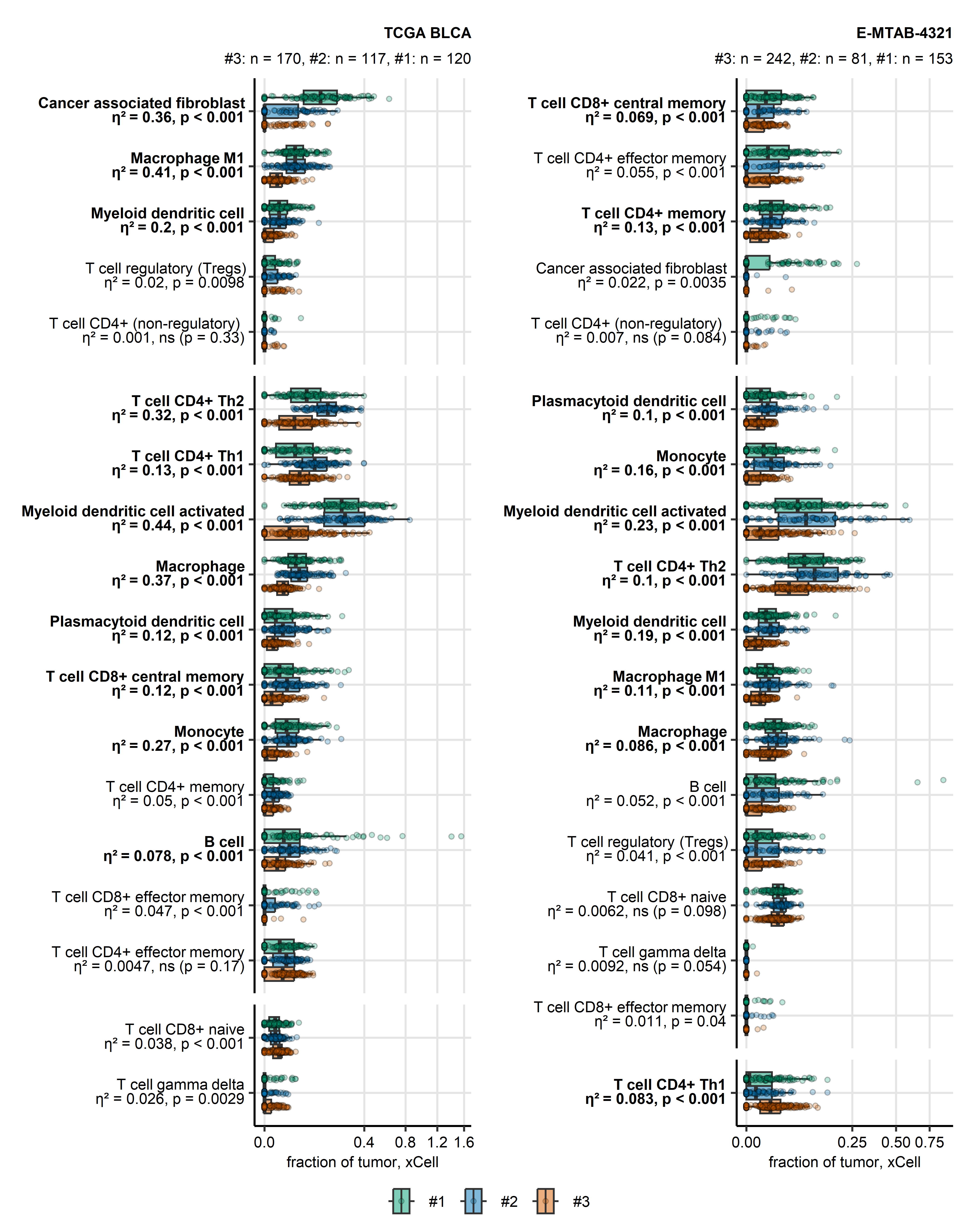


**Supplementary Figure S24. Fractions of non-malignant tumor-infiltrating cells in bladder cancer clusters predicted by xCell immunedeconvolution**

*Fractions of non-malignant cells in bulk cancer samples were estimated by xCell immunedeconvolution of bulk cancer transcriptomes. The predicted infiltrating cell fractions were compared between bladder cancer clusters by Kruskal-Wallis test with* $\eta^{2}$ *effect size statistic. P values were corrected for multiple testing with the false discovery rate (FDR). Biologically relevant differences were considered for pFDR < 0.05 and* $\eta^{2}\geq$ *0.06, which translates to significant effects of moderate-to-strong size (Supplementary Table S35).* *Fractions of cell populations found to differ between bladder cancer clusters in at least 10 out of 18 analyzed cohorts (TCGA BLCA, IMvigor, GSE13507, GSE32548, GSE48075, GSE48276, GSE83586, GSE86411, GSE87304, GSE120736, GSE124305, GSE128192, GSE128701, GSE128959, GSE198269, GSE203149, E-MTAB-4321, and Groeneveld 2024) are shown for the TCGA cohort representative for muscle invasive bladder cancer and the E-MTAB 4321 collective representative of non-muscle invasive bladder cancers. Median infiltration levels with interquartile ranges are presented as boxes with whiskers spanning over 150% of the interquartile ranges. Single cancer samples are visualized as points. Numbers of cancer samples in bladder cancer clusters are displayed in the plot captions. Effect sizes and p values are indicated in the Y axis. Biologically relevant differences are highlighted with bold font.*


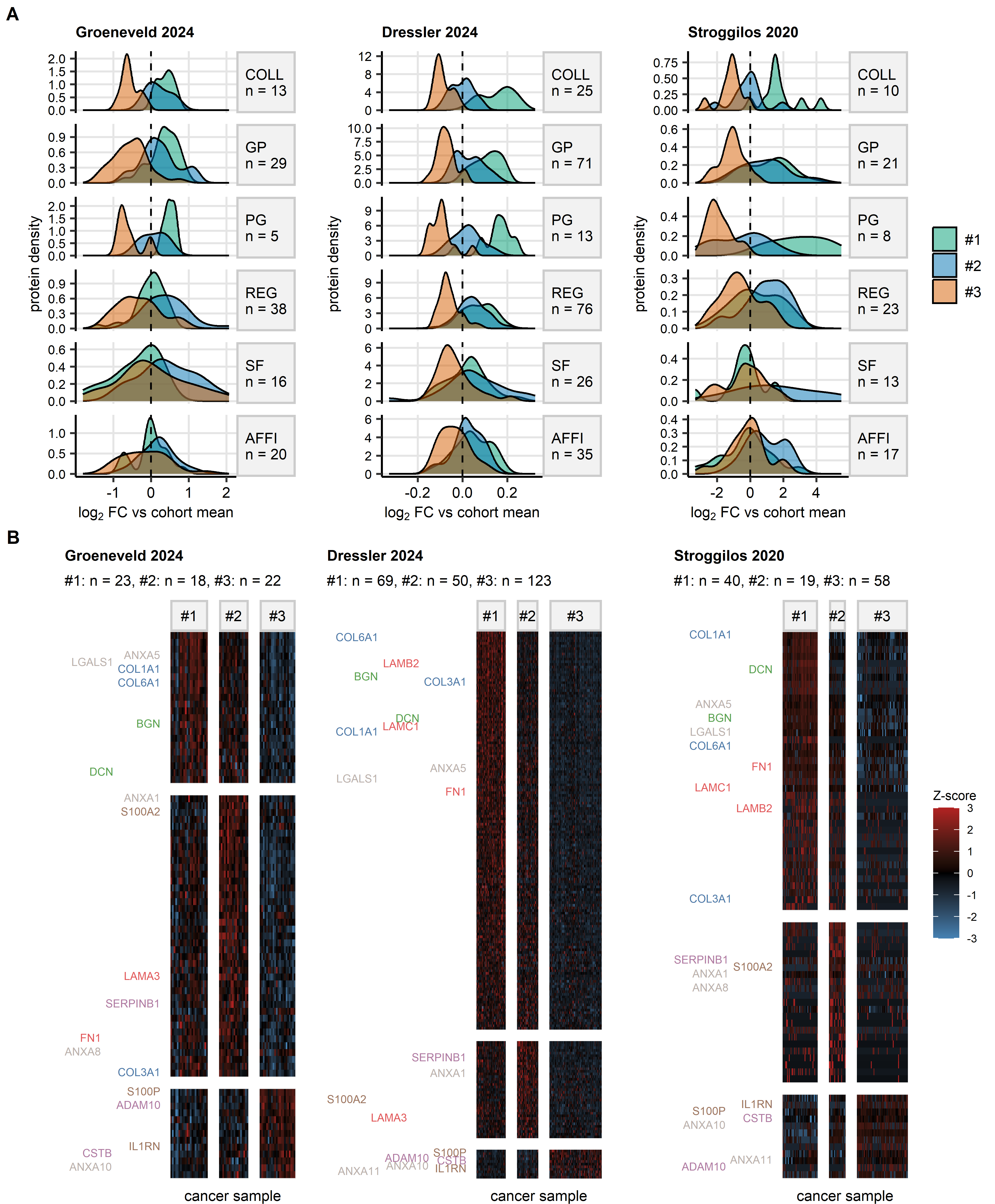


**Supplementary Figure S25. Differential regulation of matrisome proteins in bladder cancer clusters of bulk cancer proteomes.**

*Differential expression of matrisome proteins in bladder cancer clusters was investigated in the Groeneveld 2024, Dressler 2024, and Stroggilos 2020 cohorts by one-way ANOVA with one-sample post-hoc test (Supplementary Table S37).*

*(A) Density plots of* $log_{2}$ *fold change estimates of differential protein expression in bladder cancer clusters as compared with the cohort means for significantly regulated collagens (COLL), glycoproteins (GP), proteoglycans (PG), regulatory proteins (REG), secretory factors (SF), and affiliated proteins (AFFI). Numbers of differentially regulated proteins in the matrisome categories are indicated in the plot facets.*

*(B) Z-scores of* $log_{2}$ *expression levels for significantly differentially regulated matrisome proteins presented in heat maps. Selected representative members of the collagen (blue), glycoproteins (red), proteoglycans (green), regulatory proteins (purple), secretory factor (brown), and affiliated proteins (gray) are labeled with their protein symbols. Each heat map tile represents a single bulk proteome sample. Numbers of samples in bladder cancer clusters are displayed in the plot captions.*


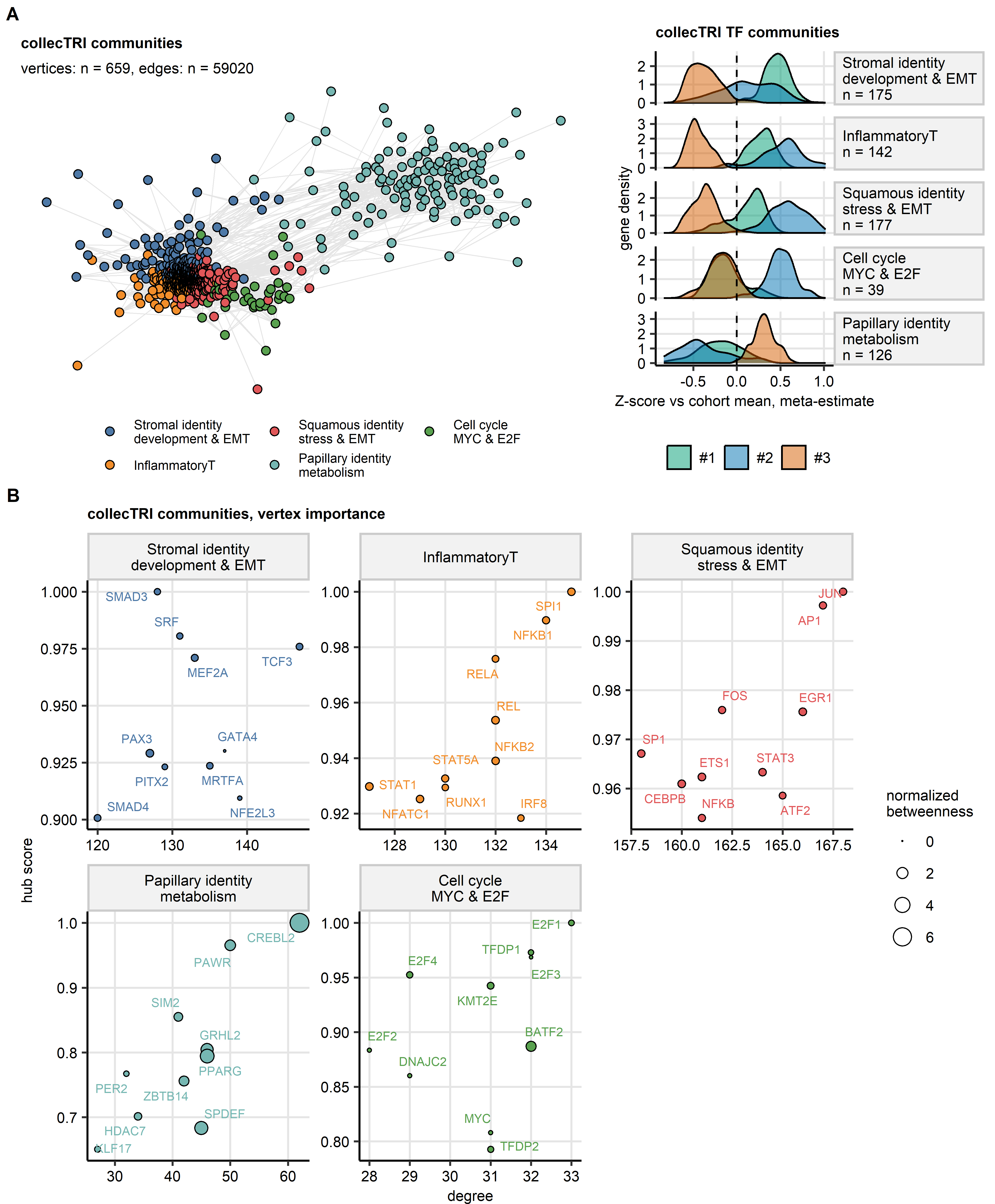


**Supplementary Figure S26. Identification of the key transcription factors for bladder cluster identity by a network analysis of collecTRI regulons.**

*Activity of collecTRI transcriptional regulons gauged by linear modeling (LM) scores was predicted for bulk cancer transcriptomes by decoupleR univariable linear modeling algorithm (n = 18 cohorts, TCGA BLCA, IMvigor, GSE13507, GSE32548, GSE48075, GSE48276, GSE83586, GSE86411, GSE87304, GSE120736, GSE124305, GSE128192, GSE128701, GSE128959, GSE198269, GSE203149, E-MTAB-4321, and Groeneveld 2024).* *LM scores were compared between bladder cancer clusters by one-way ANOVA with* $\eta^{2}$ *effect size statistic. Differences between mean LM scores in the clusters and the respective cohort means were evaluated by one-sample T test. P values were corrected for multiple comparisons with the FDR method. Significantly differentially regulated regulons and signaling pathways were defined by ANOVA pFDR < 0.05,* $\eta^{2}\geq$ *0.06, and T test pFDR < 0.05 (Supplementary Table S38).* *For regulons found to be differentially modulated between the clusters in at least 10 cohorts, meta-estimates of differences in LM scores between the clusters and cohort means were computed with the DerSimonian-Lair method (Supplementary Table S39).*

*Regulons found to be differentially modulated between the clusters in at least 10 cohorts were subjected to a network analysis in a pooled collective of normalized LM scores for all available bulk cancer transcriptome samples. The network edges were defined for pairwise correlations of LM scores with Spearman’s* $\rho\geq$ *0.3 and weighted by the* $\rho$ *values. Isolated vertices, i.e. regulons without correlation partners, were removed. Communities of co-activated regulons were identified by the Leiden algorithm and named after their biological characteristics (Supplementary Table S40).*

*(A) Visualization of the communities of co-activated regulons. The co-regulation network was plotted as a force-directed graph with the Fruchterman-Reingold algorithm (left). Regulons are visualized as point; pairwise correlations between LM scores with* $\rho\geq$ *0.3, i.e. network edges, are depicted as lines. Point color codes for the community assignment. Meta-estimates of differences of LM scores between the clusters and cohort mean for the community member regulons are displayed in a density plot (right). Numbers of regulons in the communities are indicated in the plot captions.*

*(B) Vertex importance statistics for the most essential regulons (top 10 largest hub score) in the communities. The vertex importance metrics, degree (number of correlation partners with* $\rho\geq$ *0.3), hub score (eigenvector of the correlation matrix; measure of the overall correlation strength) and betweenness (normalized number of the shortest paths between each pair of vertices of the community that pass through the vertex), are presented in scatter plots.*


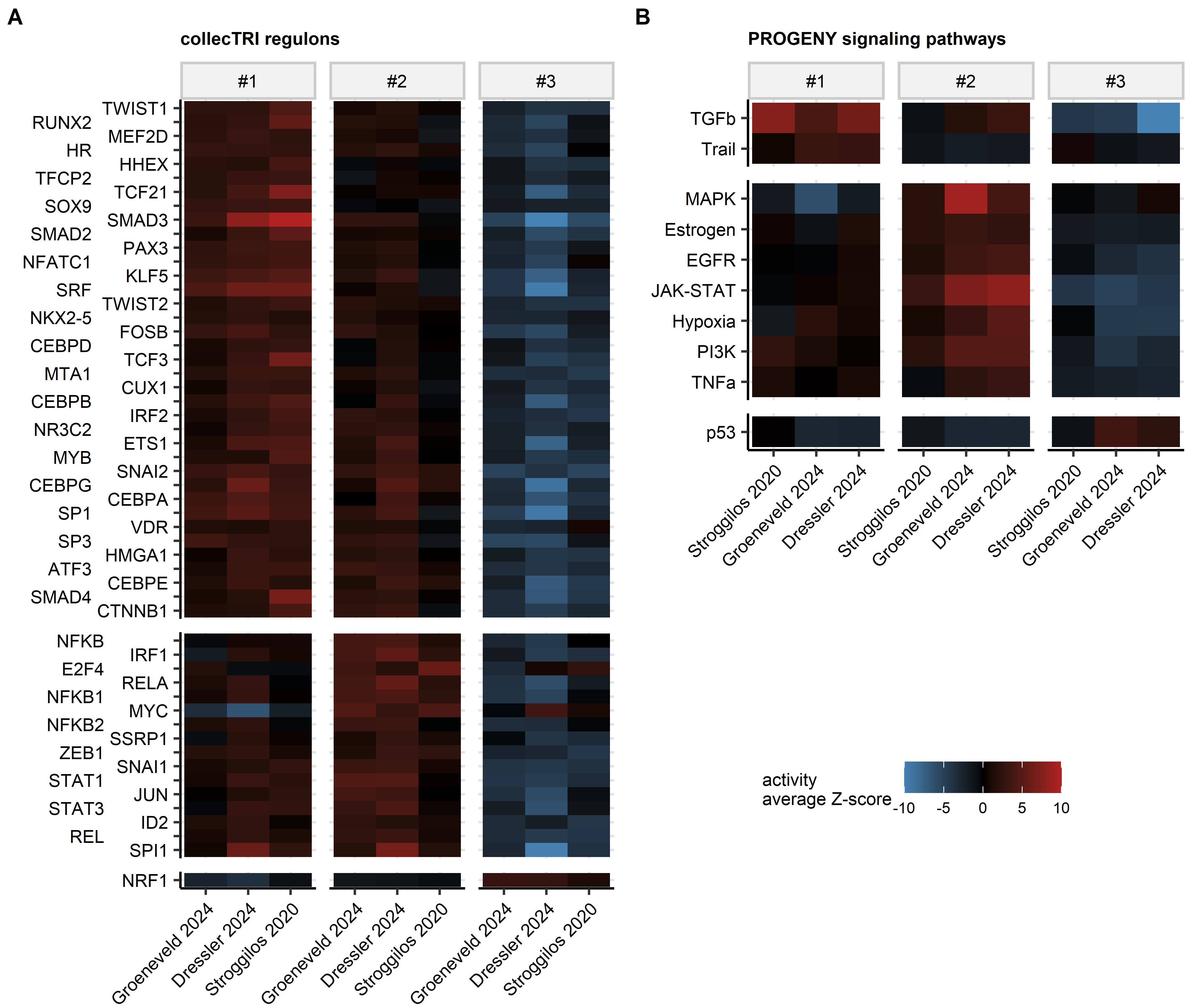


**Supplementary Figure S27. Activity of collecTRI regulons and PROGENy signaling pathways in bladder cancer clusters of bulk cancer proteomes.**

*Activity of transcriptional regulons and signaling pathways in bladder cancer clusters of bulk proteome cohorts was assessed with, respectively, univariable and multivariable linear modeling algorithms of decoupleR package, and collecTRI and PROGENy knowledge models.* *The modeling algorithms were fed with T statistics of differential protein expression in the clusters as compared with the cluster means obtained by one-sample T test for all available proteins.* *Activity of the collecTRI regulons and PROGENy signaling pathways in the clusters as compared with the cohort means was gauged by linear modeling (LM) score. P values of significant activation or inhibition were corrected for multiple testing with the false discovery rate method (Supplementary Tables S42 and S43).*

*Average normalized LM scores (Z-scores) of significant regulons (A) and signaling pathways (B) shared by at least two of the Groeneveld 2024, Dressler 2024, and Stroggilos 2020 cohorts are presented in heat maps. Each tile represents a single cluster of a cohort.*


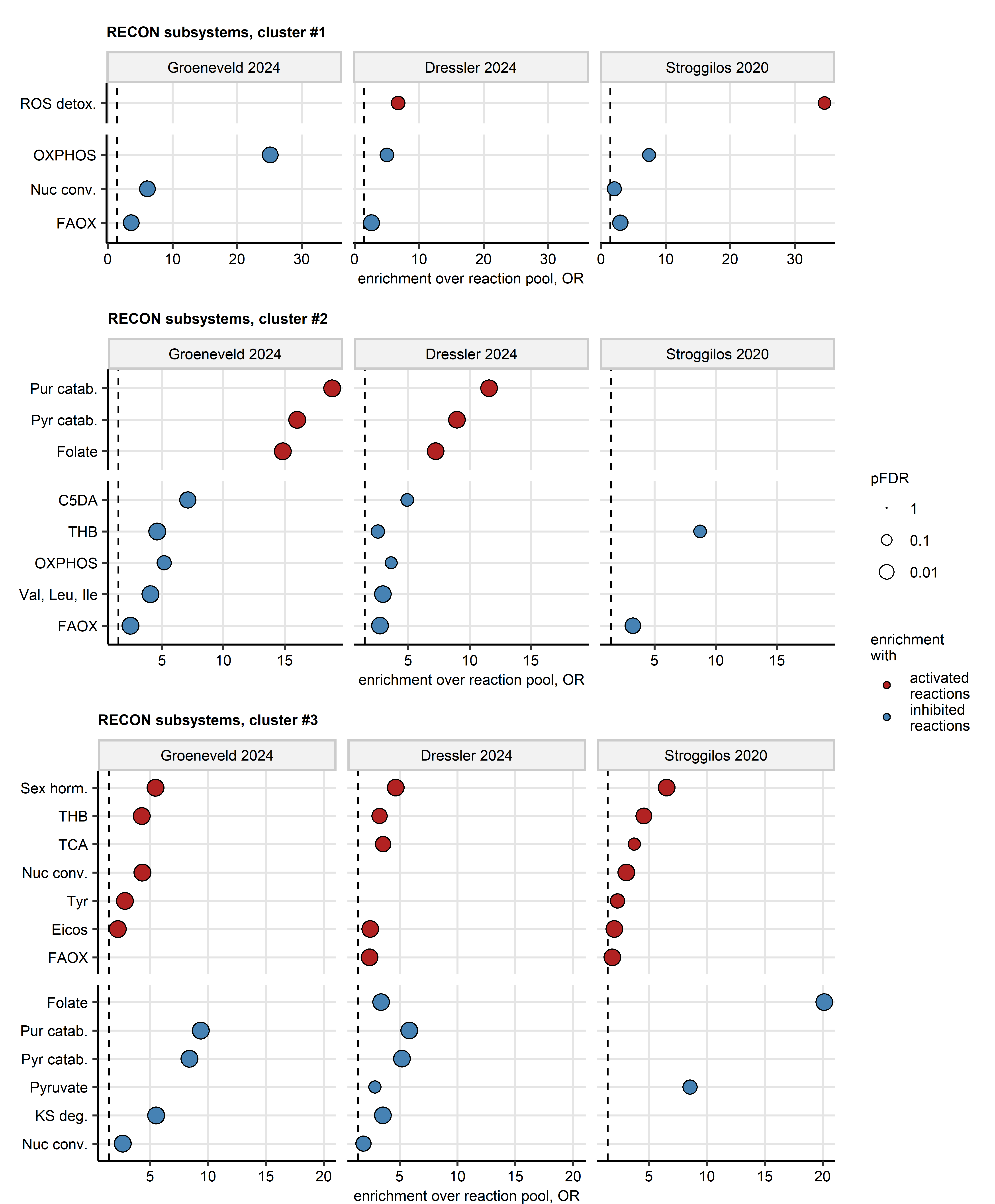


**Supplementary Figure S28. Enrichment analysis for activity of RECON metabolic subsystems in bladder cancer clusters of bulk cancer proteome cohorts**

*Activity of RECON metabolic reactions in the Groeneveld 2024, Dressler 2024, and Stroggilos 2020 proteomic cohorts was modeled by Monte Carlo simulations with tools of BiGGR and biggrExtra packages fed with* $log_{2}$ *fold-regulation estimates and standard errors of differential expression for all measured proteins (Supplementary Table S46). Enrichment of RECON metabolic subsystems with significantly activated and inhibited reactions was assessed by comparison with 10000 random draws from the entire reaction pool. Enrichment p values were corrected for multiple testing with the FDR method. Odds ratio (OR) served as a metric of enrichment magnitude. Significant enrichment with activated or inhibited reactions was considered for pFDR < 0.05 and OR* $\geq$ *1.44 (Supplementary Table S47). For the significant subsystems shared by at least two cohorts, OR values are presented in dot plots. Each point represents a single cohort. Point color codes for reaction activity status and significance, point size codes for FDR-corrected p value.*

*ROS detox.: detoxification of reactive oxygen species; OXPHOS: oxidative phosphorylation; Nuc conv.: nucleotide conversion; FAOX: fatty acid oxidation; Pur catab.: purine catabolism; Pyr catab.: pyrimidine catabolism; C5DA: C5-branched dibasic acids; THB: tetra-hydro biopterin; Val, Leu, Ile: valine, leucine, and isoleucine metabolism; Sex horm.: sex hormone metabolism; TCA: tri-carboxylic acid cycle/Krebs cycle; Tyr: tyrosine metabolism; Eicos: eicosanoid metabolism; KS deg.: keratan sulfate degradation.*


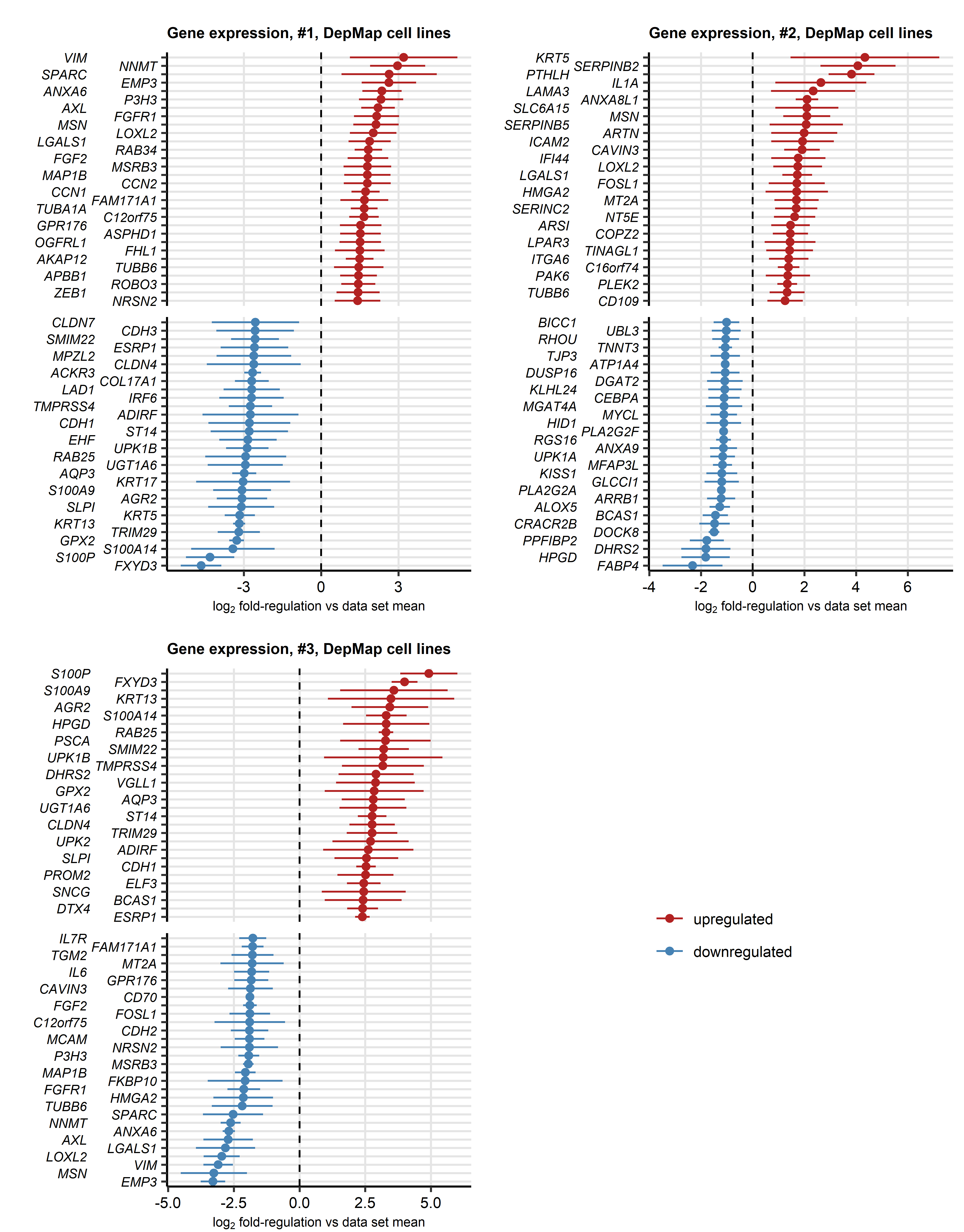


**Supplementary Figure S29. Top differentially regulated genes in bladder cancer clusters of urothelial cancer cell lines.**

$log_{2}$*-transformed gene expression levels were compared between bladder cancer clusters of DepMap urothelial cancer cell lines (cluster #1: n = 13, #2: n = 7, #3: n = 13 cell lines) by one-way ANOVA with* $\eta^{2}$ *effect size statistic. Differences between mean* $log_{2}$ *expression levels in the clusters and the respective data set means were evaluated by one-sample T test. P values were corrected for multiple comparisons with the false discovery rate (FDR) method. Significantly differentially regulated genes were defined by ANOVA pFDR < 0.05,* $\eta^{2}\geq$ *0.06, and T test pFDR < 0.05.* $log_{2}$ *fold change estimates of differential expression in the cluster as compared with the data set means the top strongest up- and downregulated genes are presented with 95% confidence intervals in Forest plots.* *Full results of differential gene expression analysis for the DepMap urothelial cancer cell lines are summarized in Supplementary Table S52.*


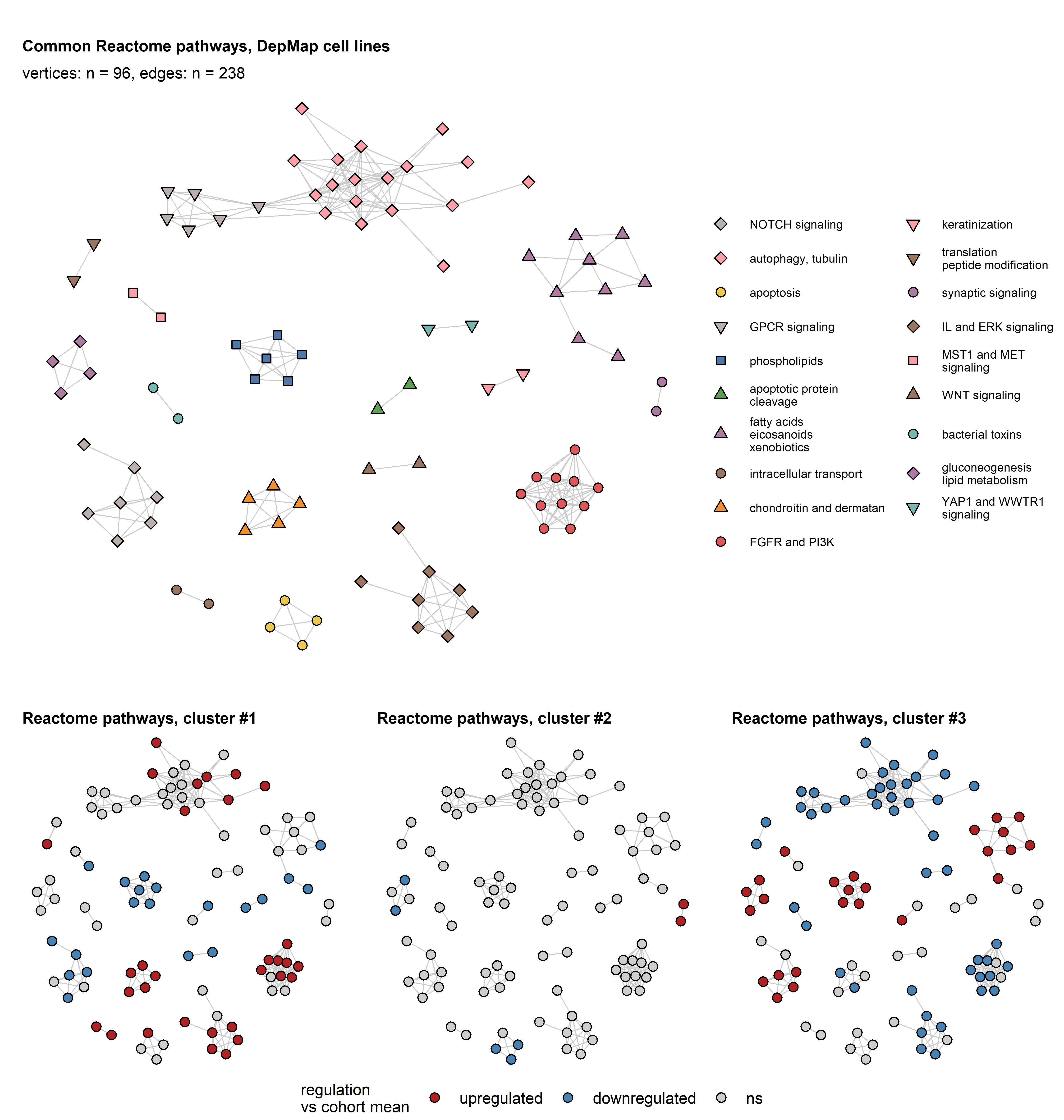


**Supplementary Figure S30. Genes set variation analysis of Reactome Pathways in bladder cancer clusters of DepMap urothelial cancer cell lines.**

*Single sample gene set enrichment analysis (ssGSEA) scores of Reactome Pathway protein signatures were compared between bladder cancer clusters of DepMap urothelial cancer cell lines (cluster #1: n = 13, #2: n = 7, #3: n = 13 cell lines) by one-way ANOVA with* $\eta^{2}$ *effect size statistic. Differences between mean ssGSEA scores in the clusters and the respective data set means were evaluated by one-sample T test. P values were corrected for multiple comparisons with the false discovery rate (FDR) method. Significantly differentially regulated Reactome Pathway signatures were defined by ANOVA pFDR < 0.05,* $\eta^{2}\geq$ *0.14, and T test pFDR < 0.05 (Supplementary Table S55).* *Reactome Pathway protein signatures found to be significantly regulated in at least 2 out of 3 analyzed cohorts were subjected to a network analysis.* *For these common differentially regulated signatures, pairwise Jaccard similarity coefficients J were computed in respect to the overlapping signature members. Networks were constructed for signatures associated with each other with with J* $\geq$ *0.3.* *Communities of overlapping signatures were identified by the Leiden algorithm and named after their key biological processes.* *Network structures were visualized as force-directed graphs with the Fruchterman–Reingold algorithm. Single signatures are represented by points, associations between signatures with J* $\geq$ *0.3 are depicted as edges. Point colors and shapes code for community assignment, or regulation status in a particular bladder cancer cluster as compared with the cohort average shared by at least 10 cohorts. Numbers of vertices (Reactome Pathways) and edges (associations between the Reactome Pathways) are displayed in the caption of the upper network plot. Assignment of the signatures to communities is summarized in Supplementary Table 56.*

*GPCR: G protein-coupled receptors; IL: interleukin.*


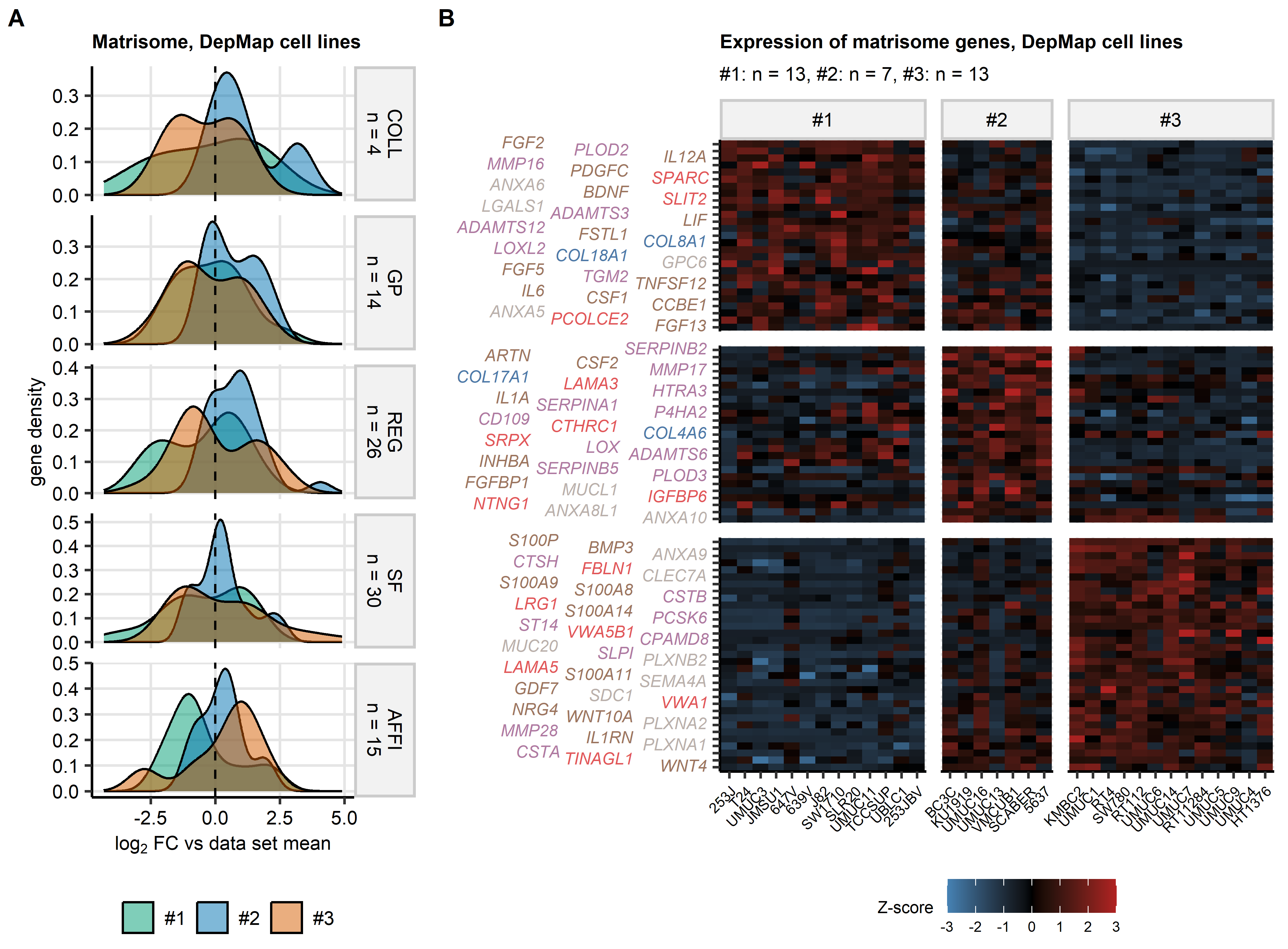


**Supplementary Figure S31. Differential regulation of matrisome genes in bladder cancer clusters of DepMap urothelial cancer cell lines.**

*Differential expression of matrisome genes in bladder cancer clusters of DepMap urothelial cancer cell lines (cluster #1: n = 13, #2: n = 7, #3: n = 13 cell lines) was investigated by one-way ANOVA with one-sample post-hoc test (Supplementary Table S57).*

*(A) Density plots of* $log_{2}$ *fold change estimates of differential genes expression in bladder cancer clusters as compared with the data set means for significantly regulated genes of collagens (COLL), glycoproteins (GP), proteoglycans (PG), regulatory proteins (REG), secretory factors (SF), and affiliated proteins (AFFI). Numbers of differentially regulated genes in the matrisome categories are indicated in the plot facets.*

*(B) Z-scores of* $log_{2}$ *expression levels for significantly differentially regulated matrisome genes presented in heat maps (collagens: blue, glycoproteins: red, proteoglycans: green, regulatory proteins: purple, secretory factors: brown, and affiliated proteins: gray). Each heat map tile represents a single cell line. Numbers of cell lines in bladder cancer clusters are displayed in the plot captions.*


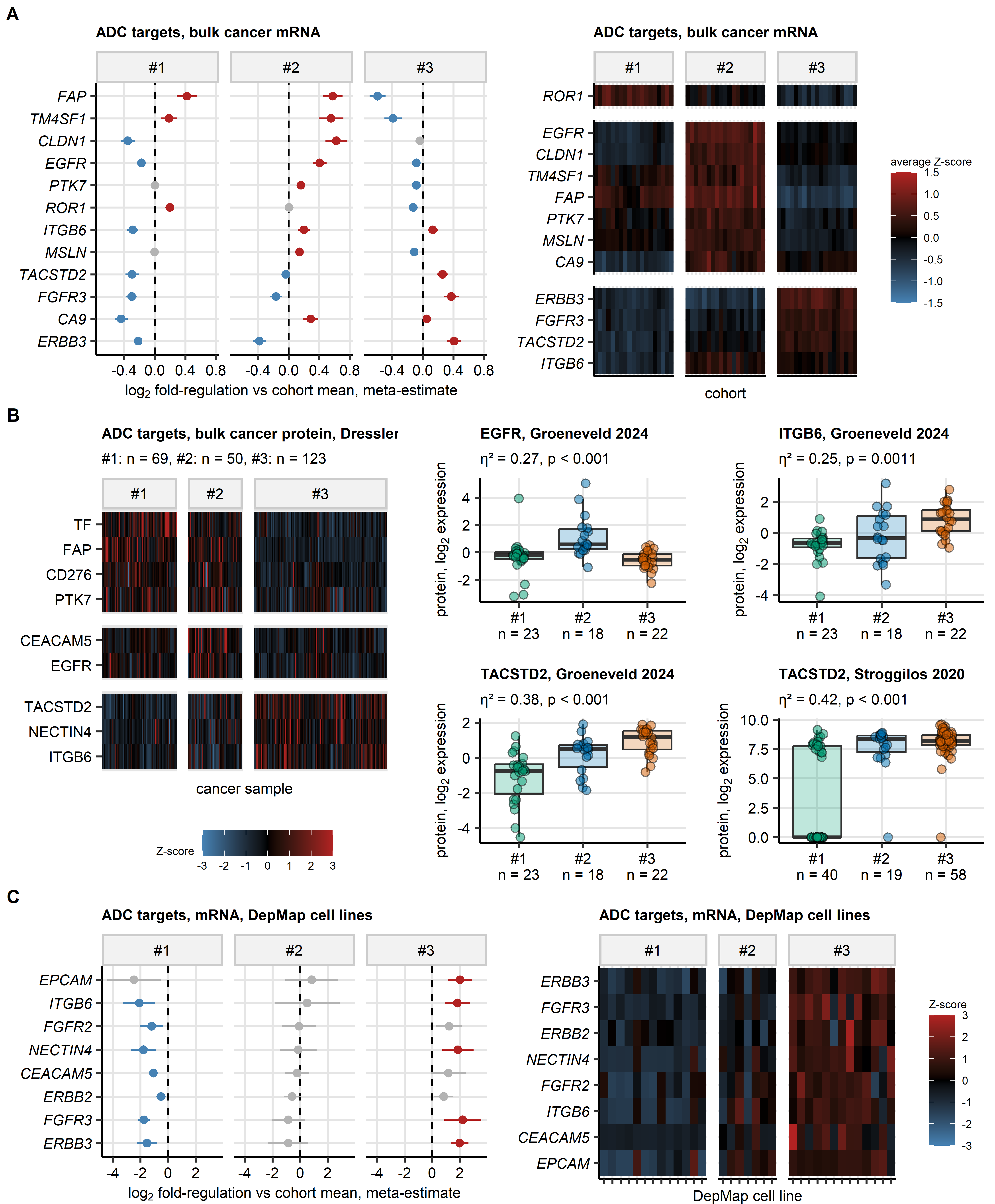


**Supplementary Figure S32. Expression of genes and proteins coding for targets of approved and experimental antibody-drug conjugates (ADC) in bladder cancer clusters.**

*Differences in expression levels of genes and proteins between bladder cancer clusters of bulk cancer transcriptomes (TCGA BLCA, IMvigor, GSE13507, GSE32548, GSE48075, GSE48276, GSE83586, GSE86411, GSE87304, GSE120736, GSE124305, GSE128192, GSE128701, GSE128959, GSE198269, GSE203149, E-MTAB-4321, Groeneveld 2024, and BCAN cohorts), bulk cancer proteomes (Groeneveld 2024, Dressler 2024, and Stroggilos 2020 cohorts), and DepMap urothelial cancer cell lines and the cohort means were investigated by one-way ANOVA with one-sample post-hoc T test as presented in Supplementary Figures S18, S19, and S29.* *Meta-estimates of differential expression in bladder cancer clusters of bulk cancer transcriptomes and cohort means were computed for genes found to be significantly regulated in at least ten bulk cancer transcriptome cohorts with the DerSimonian-Lair algorithm.*

*(A) Significant differences in expression of genes coding for ADC targets between bladder cancer clusters of bulk cancer transcriptomes shared by at least ten out of 19 cohorts. Left: meta-estimates of* $log_{2}$ *fold-differences in expression levels between the clusters and cohort means with 95% confidence intervals are presented in a Forest plot (red: significant upregulation, blue: significant downregulation, gray: non-significant). Right: mean Z-scores of* $log_{2}$ *expression levels are presented in a summary heat map; the heat map tiles represent cohorts and clusters; tile color corresponds to average Z score in the cluster and cohort.*

*(B) Significant differences in expression levels of proteins coding for ADC targets between bladder cancer clusters of bulk cancer proteomes. Left: Z-scores of* $log_{2}$ *expression levels of significant proteins in the Dressler 2024 cohort are shown in a heat map; numbers of samples in the clusters are displayed in the plot caption. Right:* $log_{2}$ *expression levels of significantly regulated proteins in the Groeneveld 2024 and Stroggilos 2020 cohort; medians with interquartile ranges are depicted as boxes, whiskers span over 150% of the interquartile ranges, single samples are visualized as points.* $\eta^{2}$ *effect size statistics and FDR-corrected p values of one-way ANOVA are displayed in the plot captions, numbers of samples are indicated in the X axes.*

*(C) Significant differences in expression of genes coding for ADC targets between bladder cancer clusters of DepMap urothelial cancer cell lines (#1: n = 13, #2: n = 7, #3: n = 13 cell lines). Left: estimates of* $log_{2}$ *differences in expression levels between the clusters and the cell collective mean with 95% confidence intervals are presented in Forest plots (red: significant upregulation, blue: significant downregulation, gray: non-significant). Right: Z-scores of* $log_{2}$ *expression levels of significant genes are shown in a heat map.*


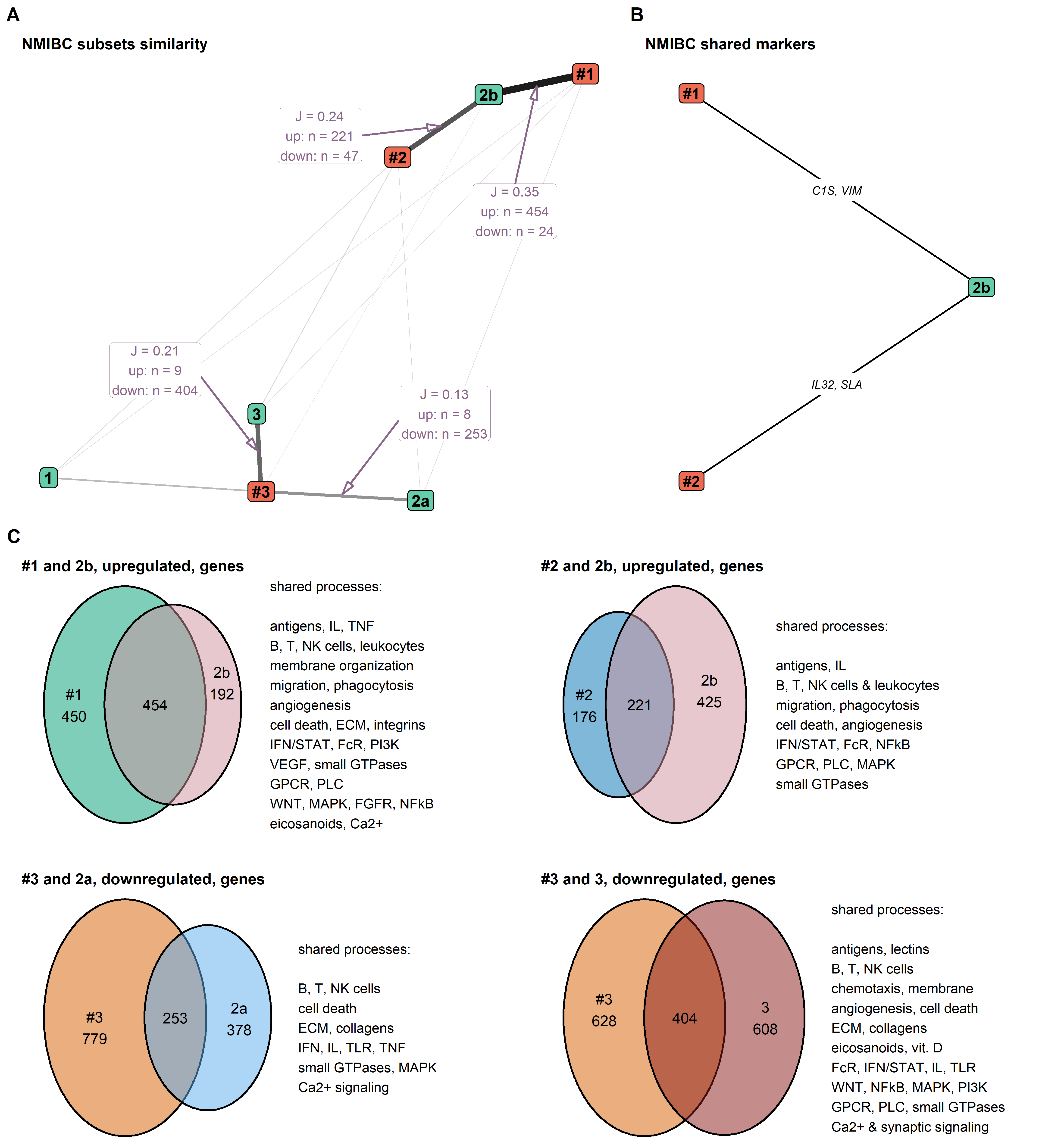


**Supplementary Figure S33. Comparison of differentially regulated transcriptomes of bladder cancer clusters and UROMOL molecular classes in non-muscle invasive bladder cancer (NMIBC).**

*Differences in* $log_{2}$*-transformed gene expression levels in bladder cancer clusters as compared with the cohort average and in UROMOL classes as compared with the cohort average were investigated in bulk NMIBC samples in the GSE13507, GSE32548, GSE86411, GSE128959, and E-MTAB-4321 cohorts.* *Statistical significance was assessed by one-way ANOVA with* $\eta^{2}$ *effect size metric and post-hoc one-sample T test, a strategy presented in Supplementary Figure S18. P values were adjusted for multiple testing with the false discovery rate (FDR) method. Significant differences were considered for pFDR(ANOVA) < 0.05,* $\eta^{2}\geq$ *0.06, and pFDR(T test) < 0.05. Subsequently, common differentially regulated genes were defined as genes significantly regulated in at least three cohorts.* *Common markers of bladder cancer clusters or UROMOL classes were identified among genes with differential expression between the subsets (pFDR(ANOVA) < 0.05,* $\eta^{2}\geq$ *0.06) by receiver-operating characteristics (ROC) with area under the ROC curve AUC >= 0.714 in at least three cohorts.*

*(A) Pairwise similarities of bladder cancer clusters and UROMOL classes of NMIBC in respect to the common differentially regulated genes measured by Jaccard’s similarity coefficients J. The similarities are visualized as a weighted graph with Fruchterman-Reingold method. Width and color of edges corresponds to the J value. Values of J, numbers of significantly up- and downregulated genes in at least three cohorts and shared between the bladder cancer clusters and UROMOL classes are indicated in the edge labels.*

*(B) Marker genes identified in at least three cohorts and shared between bladder cancer clusters #1 and #2 and UROMOL class 2b.*

*(C) Quantification and biological function of differentially regulated genes identified in at least three cohorts and shared between bladder cancer clusters and UROMOL classes. Numbers of concordantly and discordantly regulated genes in bladder cancer cluster #1 and UROMOL class 2b (upregulated genes), in bladder cancer cluster #2 and UROMOL class 2b (upregulated genes), and bladder cancer cluster #3 and UROMOL classes 2a and 3 (downregulated genes) are presented in Euler plots. Biological functions of the differentially regulated genes shared by the bladder cancer clusters and UROMOL classes were investigated by biological process gene ontology (GO) enrichment analyses. Summaries of significantly enriched GO terms (at least three shared differentially regulated genes per term, odds ratio* $\geq$ *1.44, pFDR < 0.05) are displayed next to the Euler plots.*


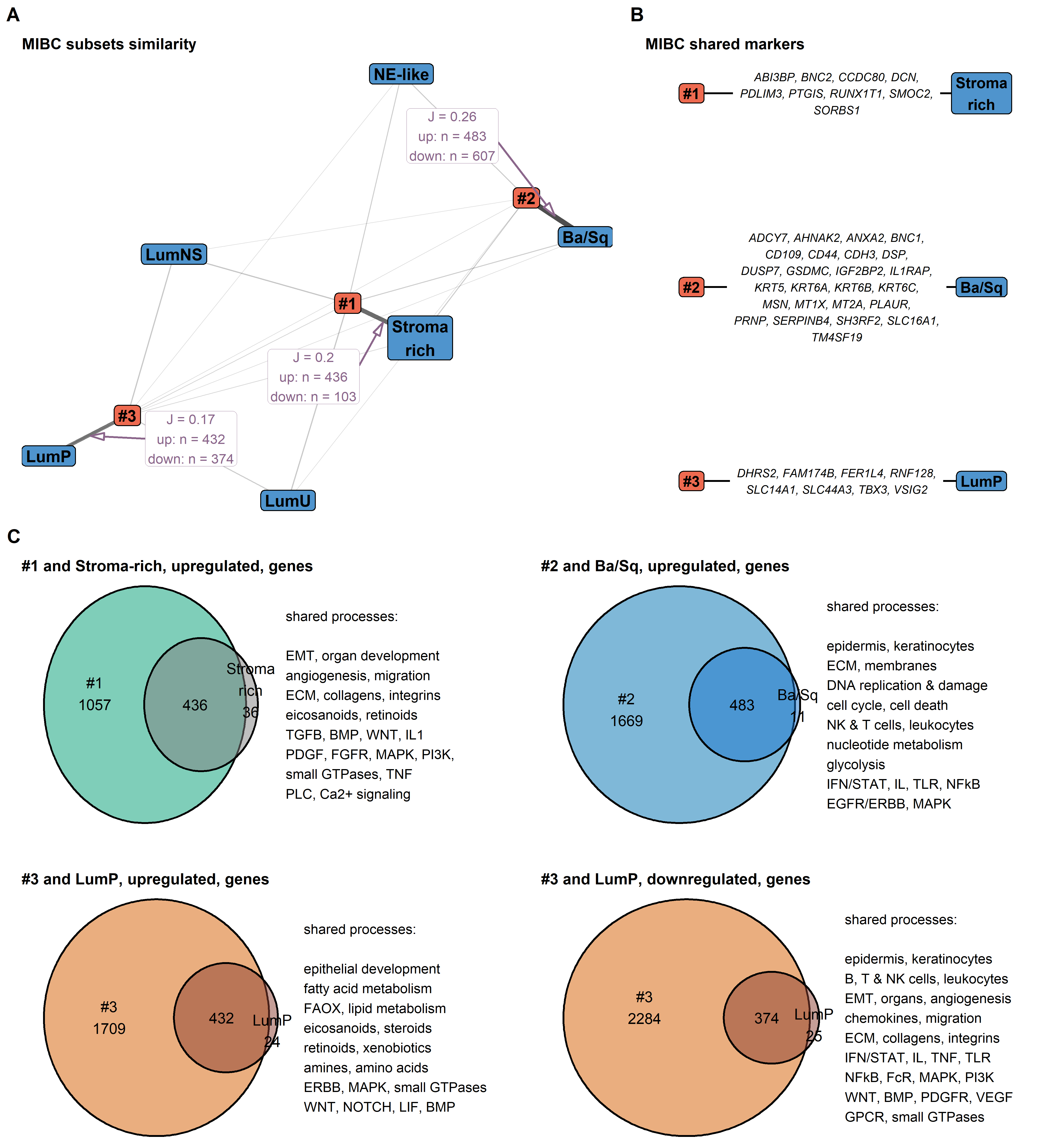


**Supplementary Figure S34. Comparison of differentially regulated transcriptomes of bladder cancer clusters and consensus molecular classes in muscle invasive bladder cancer (MIBC).**

*Differences in* $log_{2}$*-transformed gene expression levels in bladder cancer clusters as compared with the cohort average and in consensus classes as compared with the cohort average were investigated in bulk MIBC samples in the TCGA BLCA, IMvigor, GSE83586, GSE87304, GSE128192, and GSE203149 cohorts.* *Statistical significance was assessed by one-way ANOVA with* $\eta^{2}$ *effect size metric and post-hoc one-sample T test, a strategy presented in Supplementary Figure S18. P values were adjusted for multiple testing with the false discovery rate (FDR) method. Significant differences were considered for pFDR(ANOVA) < 0.05,* $\eta^{2}\geq$ *0.06, and pFDR(T test) < 0.05. Subsequently, common differentially regulated genes were defined as genes significantly regulated in at least three cohorts.* *Common markers of bladder cancer clusters or consensus classes were identified among genes with differential expression between the subsets (pFDR(ANOVA) < 0.05,* $\eta^{2}\geq$ *0.06) by receiver-operating characteristics (ROC) with area under the ROC curve AUC >= 0.714 in at least three cohorts.*

*(A) Pairwise similarities of bladder cancer clusters and consensus classes of MIBC in respect to the common differentially regulated genes measured by Jaccard’s similarity coefficients J. The similarities are visualized as a weighted graph with Fruchterman-Reingold method. Width and color of edges corresponds to the J value. Values of J, numbers of significantly up- and downregulated genes in at least three cohorts and shared between the bladder cancer clusters and consensus classes are indicated in the edge labels.*

*(B) Marker genes identified in at least three cohorts and shared between bladder cancer clusters #1, #2 and #3, and stroma-rich, basal/squamous (Ba/Sq) and luminal-papillary (LumP) consensus classes.*

*(C) Quantification and biological function of differentially regulated genes identified in at least three cohorts and shared between bladder cancer clusters and consensus classes of MIBC. Numbers of concordantly and discordantly regulated genes in bladder cancer cluster #1 and stroma-rich consensus class (upregulated genes), bladder cancer cluster #2 and Ba/Sq consensus class, and bladder cancer cluster #3 and LumP consensus class (up- and downregulated genes) are presented in Euler plots. Biological functions of the differentially regulated genes shared by the bladder cancer clusters and consensus classes were investigated by biological process gene ontology (GO) enrichment analyses. Summaries of significantly enriched GO terms (at least three shared differentially regulated genes per term, odds ratio* $\geq$ *1.44, pFDR < 0.05) are displayed next to the Euler plots.*


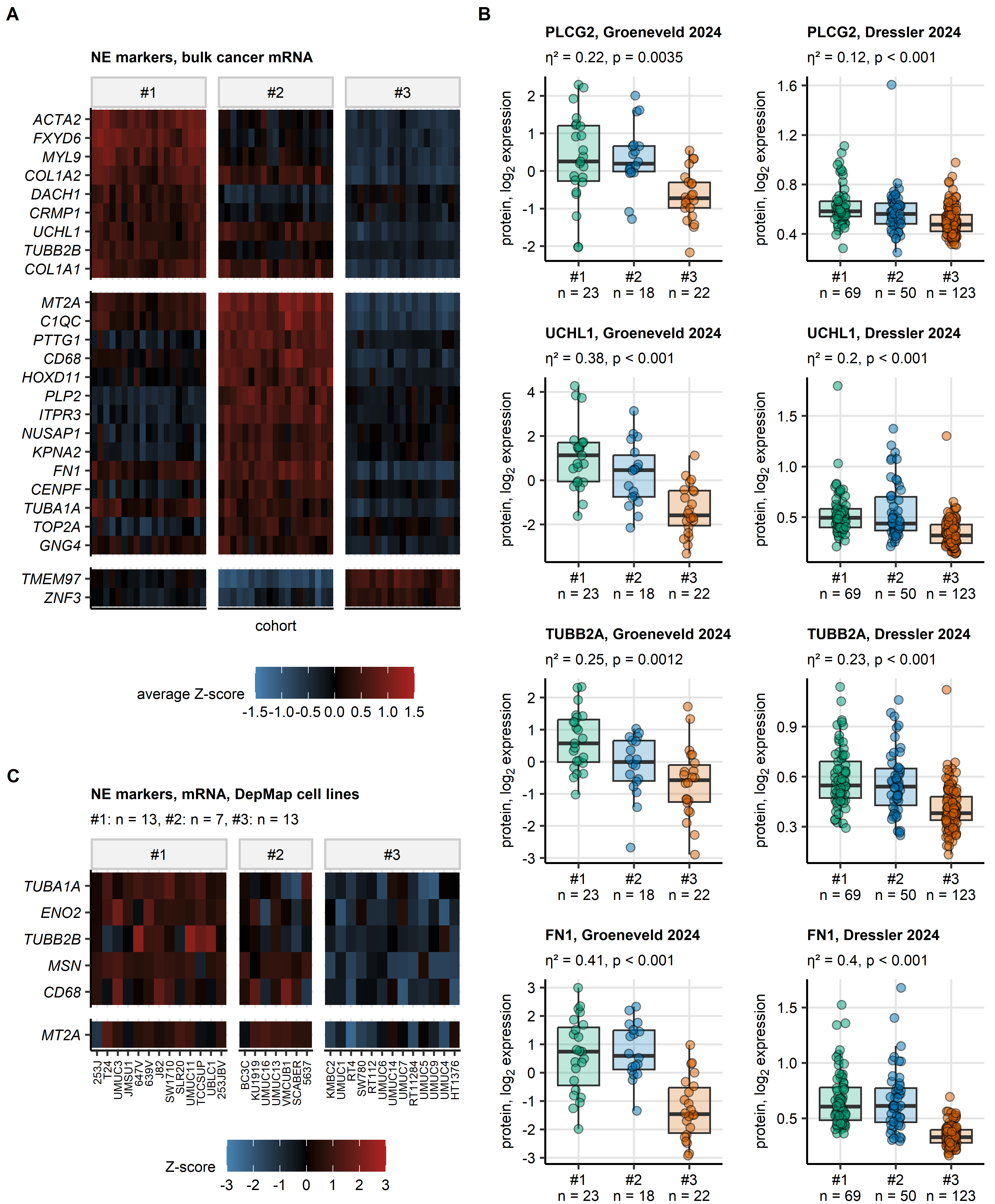


**Supplementary Figure S35. Gene and protein expression of markers of neuroendocrine differentiation in bladder cancer clusters.**

*Differences in expression levels of genes and proteins between bladder cancer clusters of bulk cancer transcriptomes (TCGA BLCA, IMvigor, GSE13507, GSE32548, GSE48075, GSE48276, GSE83586, GSE86411, GSE87304, GSE120736, GSE124305, GSE128192, GSE128701, GSE128959, GSE198269, GSE203149, E-MTAB-4321, Groeneveld 2024, and BCAN cohorts), bulk cancer proteomes (Groeneveld 2024, Dressler 2024, and Stroggilos 2020 cohorts), and DepMap urothelial cancer cell lines and the cohort means were investigated by one-way ANOVA with one-sample post-hoc T test as presented in Supplementary Figures S18, S19, and S29.* *Meta-estimates of differential expression in bladder cancer clusters of bulk cancer transcriptomes and cohort means were computed for genes found to be significantly regulated in at least ten bulk cancer transcriptome cohorts with the DerSimonian-Lair algorithm.*

*(A) Significant differences in expression of genes coding for markers of neuroendocrine differentiation between bladder cancer clusters of bulk cancer transcriptomes shared by at least ten out of 19 cohorts. Mean Z-scores of* $log_{2}$ *expression levels are presented in a summary heat map; the heat map tiles represent cohorts and clusters; tile color corresponds to average Z score in the cluster and cohort.*

*(B) Significant differences in expression levels of PLCG2, UCHL1, TUBB2A, and FN1 neuroendocrine marker proteins between bladder cancer clusters of bulk cancer proteomes in the Groeneveld 2024 and Dressler 2024 cohort. Medians with interquartile ranges are depicted as boxes, whiskers span over 150% of the interquartile ranges, single samples are visualized as points.* $\eta^{2}$ *effect size statistics and FDR-corrected p values of one-way ANOVA are displayed in the plot captions, numbers of samples are indicated in the X axes.*

*(C) Significant differences in expression of genes coding for neuroendocrine markers between bladder cancer clusters of DepMap urothelial cancer cell lines. Z-scores of* $log_{2}$ *expression levels of significant genes are shown in a heat map, numbers of cell lines in the clusters are indicated in the plot caption.*


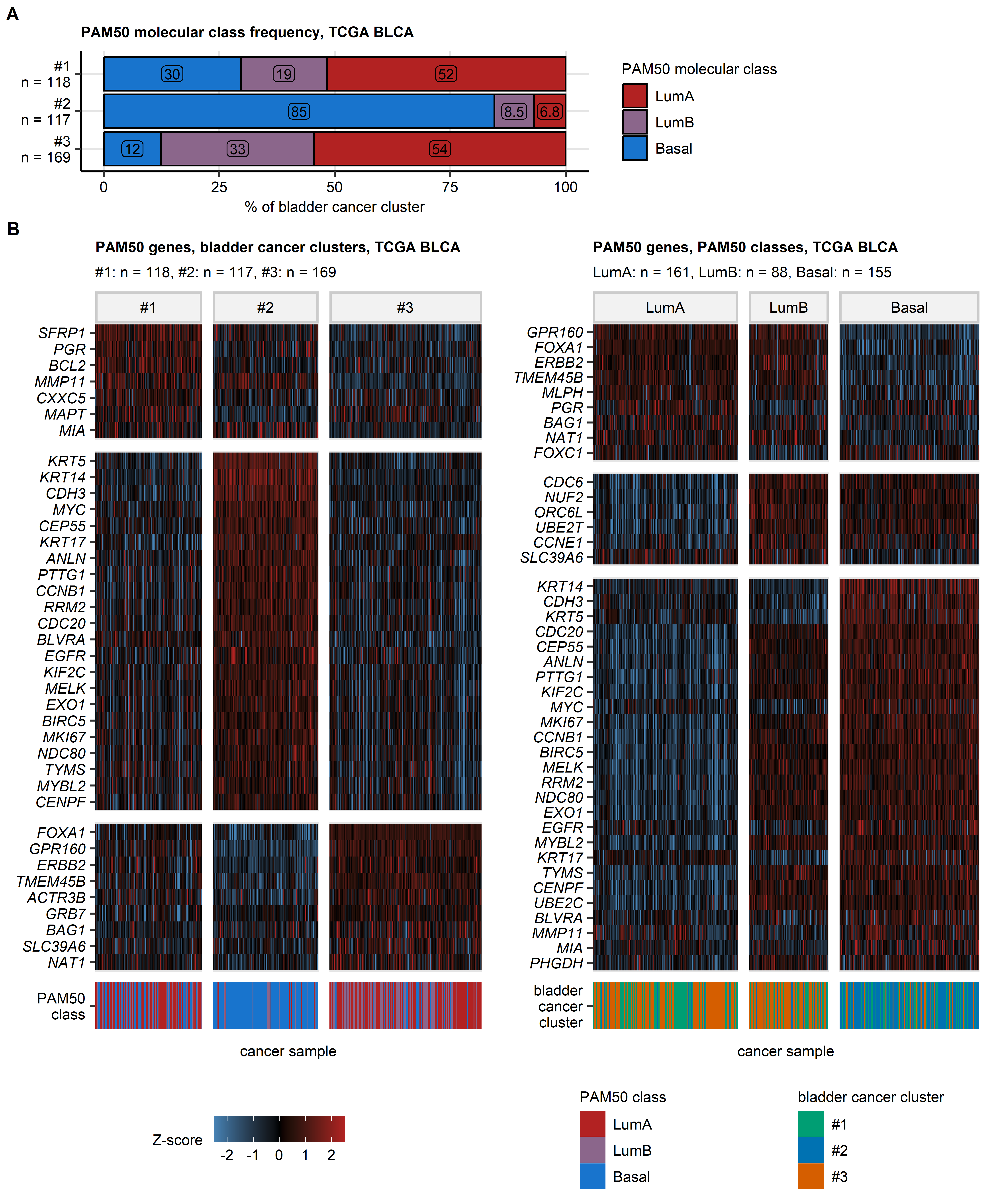


**Supplementary Figure S36. PAM50 molecular classes and bladder cancer clusters in the TCGA BLCA cohort.**

*Samples in the TCGA BLCA cohort were assigned to luminal A (LumA), luminal B (LumB), and basal molecular clusters defined by expression of PAM50 genes by Zhao et colleagues.*

*(A) Comparison of percentages of PAM50 molecular subsets in bladder cancer clusters. Percentages of samples in bladder cancer clusters are presented in a stack plot. Numbers of samples in bladder cancer clusters are indicated in the Y axis.*

*(B) Differential expression of PAM50 genes in bladder cancer clusters and PAM50 molecular subsets of the TCGA BLCA cohort. Differences in* $log_{2}$ *expression of PAM50 transcripts in bladder cancer clusters and PAM50 molecular subsets of the TCGA BLCA cohort as compared with the cohort average was investigated by false discovery rate (FDR) adjusted one-way ANOVA with* $\eta^{2}$ *effect size statistic and one-sample post-hoc T test. Differential regulation was considered for pFDR(ANOVA) < 0.05,* $\eta^{2}\geq$ *0.06, and pFDR(T test) < 0.05. Expression Z-scores of significantly differentially regulated PAM50 genes in bladder cancer clusters and PAM50 molecular classes are shown in heat maps. Numbers of samples in the molecular subsets are displayed in the plot captions.*

# References

1. Wickham H, Averick M, Bryan J, Chang W, McGowan L, François R, Grolemund G, Hayes A, Henry L, Hester J, et al. Welcome to the Tidyverse. *Journal of Open Source Software* (2019) 4:1686. doi: [10.21105/joss.01686](https://doi.org/10.21105/joss.01686)

2. Henry L, Wickham Hadley. rlang: Functions for Base Types and Core R and ’Tidyverse’ Features. (2022) <https://cran.r-project.org/web/packages/rlang/index.html>

3. Gagolewski M, Tartanus B. Package ’stringi’. (2021) [https://cran.r-project.org/web/packages/stringi/index.html http://cran.ism.ac.jp/web/packages/stringi/stringi.pdf](https://cran.r-project.org/web/packages/stringi/index.html%20http://cran.ism.ac.jp/web/packages/stringi/stringi.pdf)

4. Gohel D. flextable: Functions for Tabular Reporting. (2022) <https://cran.r-project.org/web/packages/flextable/index.html>

5. Wickham Hadley. *ggplot2: Elegant Graphics for Data Analysis*. 1st ed. New York: Springer-Verlag (2016). <https://ggplot2.tidyverse.org>

6. Csardi G, Nepusz T. The igraph software package for complex network research. *InterJournal* (2006) Complex Sy:1695. <https://igraph.org>

7. Briatte F, Bojanowski M, Canouil M, Charlop-Powers Z, Fisher JC, Johnson K, Rinker T. ggnetwork: Geometries to Plot Networks with ’ggplot2’. (2021) <https://cran.r-project.org/package=ggnetwork>

8. Slowikowski K, Shep A, Hughes S, Dang TK, Lukauskas S, Irisson J-O, Kamvar ZN, Ryan T, Dervieux C, Yutani H, et al. ggrepel: Automatically Position Non-Overlapping Text Labels with ’ggplot2’. (2023) <https://cran.r-project.org/web/packages/ggrepel/index.html>

9. Wilke CO, Wiernik BM. ggtext: Improved Text Rendering Support for ’ggplot2’. (2022) <https://cran.r-project.org/package=ggtext>

10. Arnold JB. ggthemes: Extra Themes, Scales and Geoms for ’ggplot2’. (2024) doi: [10.32614/CRAN.PACKAGE.GGTHEMES](https://doi.org/10.32614/CRAN.PACKAGE.GGTHEMES)

11. Wilke CO. *Fundamentals of Data Visualization: A Primer on Making Informative and Compelling Figures*. 1st ed. Sebastopol: O’Reilly Media (2019).

12. Allaire J, Xie Y, McPherson J, Luraschi J, Ushey K, Atkins A, Wickham H, Cheng J. rmarkdown: Dynamic Documents for R. (2022) <https://cran.r-project.org/web/packages/rmarkdown/index.html>

13. Xie Y. *Bookdown: Authoring books and technical documents with R Markdown*. (2016). doi: [10.1201/9781315204963](https://doi.org/10.1201/9781315204963)

14. Sean D, Meltzer PS. GEOquery: a bridge between the Gene Expression Omnibus (GEO) and BioConductor. *Bioinformatics (Oxford, England)* (2007) 23:1846–1847. doi: [10.1093/BIOINFORMATICS/BTM254](https://doi.org/10.1093/BIOINFORMATICS/BTM254)

15. Robertson AG, Kim J, Al-Ahmadie H, Bellmunt J, Guo G, Cherniack AD, Hinoue T, Laird PW, Hoadley KA, Akbani R, et al. Comprehensive Molecular Characterization of Muscle-Invasive Bladder Cancer. *Cell* (2017) 171:540–556.e25. doi: [10.1016/J.CELL.2017.09.007](https://doi.org/10.1016/J.CELL.2017.09.007)

16. Liu J, Lichtenberg T, Hoadley KA, Poisson LM, Lazar AJ, Cherniack AD, Kovatich AJ, Benz CC, Levine DA, Lee AV, et al. An Integrated TCGA Pan-Cancer Clinical Data Resource to Drive High-Quality Survival Outcome Analytics. *Cell* (2018) 173:400–416.e11. doi: [10.1016/J.CELL.2018.02.052](https://doi.org/10.1016/J.CELL.2018.02.052)

17. Moiso E. Manual curation of TCGA treatment data and identification of potential markers of therapy response. *medRxiv* (2021)2021.04.30.21251941. doi: [10.1101/2021.04.30.21251941](https://doi.org/10.1101/2021.04.30.21251941)

18. Zhao SG, Chen WS, Das R, Chang SL, Tomlins SA, Chou J, Quigley DA, Dang HX, Barnard TJ, Mahal BA, et al. Clinical and genomic implications of luminal and basal subtypes across carcinomas. *Clinical Cancer Research* (2019) 25:2450–2457. doi: [10.1158/1078-0432.CCR-18-3121](https://doi.org/10.1158/1078-0432.CCR-18-3121)

19. Balar AV, Galsky MD, Rosenberg JE, Powles T, Petrylak DP, Bellmunt J, Loriot Y, Necchi A, Hoffman-Censits J, Perez-Gracia JL, et al. Atezolizumab as first-line therapy in cisplatin-ineligible patients with locally advanced and metastatic urothelial carcinoma: a single-arm, multicentre, phase 2 trial. *Lancet (London, England)* (2017) 389:67. doi: [10.1016/S0140-6736(16)32455-2](https://doi.org/10.1016/S0140-6736(16)32455-2)

20. Mariathasan S, Turley SJ, Nickles D, Castiglioni A, Yuen K, Wang Y, Kadel EE, Koeppen H, Astarita JL, Cubas R, et al. TGF$\beta$ attenuates tumour response to PD-L1 blockade by contributing to exclusion of T cells. *Nature 2018 554:7693* (2018) 554:544–548. doi: [10.1038/nature25501](https://doi.org/10.1038/nature25501)

21. Kim WJ, Kim EJ, Kim SK, Kim YJ, Ha YS, Jeong PP, Kim MJ, Yun SJ, Lee KM, Moon SK, et al. Predictive value of progression-related gene classifier in primary non-muscle invasive bladder cancer. *Molecular cancer* (2010) 9: doi: [10.1186/1476-4598-9-3](https://doi.org/10.1186/1476-4598-9-3)

22. Lindgren D, Sjödahl G, Lauss M, Staaf J, Chebil G, Lövgren K, Gudjonsson S, Liedberg F, Patschan O, Månsson W, et al. Integrated genomic and gene expression profiling identifies two major genomic circuits in urothelial carcinoma. *PloS one* (2012) 7: doi: [10.1371/JOURNAL.PONE.0038863](https://doi.org/10.1371/JOURNAL.PONE.0038863)

23. Choi W, Porten S, Kim S, Willis D, Plimack ER, Hoffman-Censits J, Roth B, Cheng T, Tran M, Lee IL, et al. Identification of distinct basal and luminal subtypes of muscle-invasive bladder cancer with different sensitivities to frontline chemotherapy. *Cancer cell* (2014) 25:152–165. doi: [10.1016/J.CCR.2014.01.009](https://doi.org/10.1016/J.CCR.2014.01.009)

24. Sjödahl G, Eriksson P, Liedberg F, Höglund M. Molecular classification of urothelial carcinoma: global mRNA classification versus tumour-cell phenotype classification. *The Journal of pathology* (2017) 242:113–125. doi: [10.1002/PATH.4886](https://doi.org/10.1002/PATH.4886)

25. Guo CC, Bondaruk J, Yao H, Wang Z, Zhang L, Lee S, Lee JG, Cogdell D, Zhang M, Yang G, et al. Assessment of Luminal and Basal Phenotypes in Bladder Cancer. *Scientific reports* (2020) 10: doi: [10.1038/S41598-020-66747-7](https://doi.org/10.1038/S41598-020-66747-7)

26. Seiler R, Ashab HAD, Erho N, Rhijn BWG van, Winters B, Douglas J, Van Kessel KE, Fransen van de Putte EE, Sommerlad M, Wang NQ, et al. Impact of Molecular Subtypes in Muscle-invasive Bladder Cancer on Predicting Response and Survival after Neoadjuvant Chemotherapy. *European urology* (2017) 72:544–554. doi: [10.1016/J.EURURO.2017.03.030](https://doi.org/10.1016/J.EURURO.2017.03.030)

27. Song BN, Kim SK, Mun JY, Choi YD, Leem SH, Chu IS. Identification of an immunotherapy-responsive molecular subtype of bladder cancer. *EBioMedicine* (2019) 50:238–245. doi: [10.1016/J.EBIOM.2019.10.058](https://doi.org/10.1016/J.EBIOM.2019.10.058)

28. Seiler R, Gibb EA, Wang NQ, Oo HZ, Lam HM, Kessel KE van, Voskuilen CS, Winters B, Erho N, Takhar MM, et al. Divergent Biological Response to Neoadjuvant Chemotherapy in Muscle-invasive Bladder Cancer. *Clinical cancer research : an official journal of the American Association for Cancer Research* (2019) 25:5082–5093. doi: [10.1158/1078-0432.CCR-18-1106](https://doi.org/10.1158/1078-0432.CCR-18-1106)

29. Guo CC, Majewski T, Zhang L, Yao H, Bondaruk J, Wang Y, Zhang S, Wang Z, Lee JG, Lee S, et al. Dysregulation of EMT Drives the Progression to Clinically Aggressive Sarcomatoid Bladder Cancer. *Cell reports* (2019) 27:1781–1793.e4. doi: [10.1016/J.CELREP.2019.04.048](https://doi.org/10.1016/J.CELREP.2019.04.048)

30. Efstathiou JA, Mouw KW, Gibb EA, Liu Y, Wu CL, Drumm MR, Costa JB da, Plessis M du, Wang NQ, Davicioni E, et al. Impact of Immune and Stromal Infiltration on Outcomes Following Bladder-Sparing Trimodality Therapy for Muscle-Invasive Bladder Cancer. *European urology* (2019) 76:59–68. doi: [10.1016/J.EURURO.2019.01.011](https://doi.org/10.1016/J.EURURO.2019.01.011)

31. Sjödahl G, Eriksson P, Patschan O, Marzouka NAD, Jakobsson L, Bernardo C, Lövgren K, Chebil G, Zwarthoff E, Liedberg F, et al. Molecular changes during progression from nonmuscle invasive to advanced urothelial carcinoma. *International journal of cancer* (2020) 146:2636–2647. doi: [10.1002/IJC.32737](https://doi.org/10.1002/IJC.32737)

32. Heijden MS van der, Powles T, Petrylak D, Wit R de, Necchi A, Sternberg CN, Matsubara N, Nishiyama H, Castellano D, Hussain SA, et al. Predictive biomarkers for survival benefit with ramucirumab in urothelial cancer in the RANGE trial. *Nature communications* (2022) 13: doi: [10.1038/S41467-022-29441-Y](https://doi.org/10.1038/S41467-022-29441-Y)

33. Smith T, Lane B, More, E, Valentine H, Lunj S, Irlam-Jones J, Reeves K, Hoskin P, Choudhury A, West C. GSE203149: Gene expression data from muscle-invasive bladder cancer samples. (2022) <https://www.ncbi.nlm.nih.gov/geo/query/acc.cgi?acc=GSE203149> [Accessed July 1, 2025]

34. Hedegaard J, Lamy P, Nordentoft I, Algaba F, Høyer S, Ulhøi BP, Vang S, Reinert T, Hermann GG, Mogensen K, et al. Comprehensive Transcriptional Analysis of Early-Stage Urothelial Carcinoma. *Cancer Cell* (2016) 30:27–42. doi: [10.1016/j.ccell.2016.05.004](https://doi.org/10.1016/j.ccell.2016.05.004)

35. Groeneveld CS, Sanchez-Quiles V, Dufour F, Shi M, Dingli F, Nicolle R, Chapeaublanc E, Poullet P, Jeffery D, Krucker C, et al. Proteogenomic Characterization of Bladder Cancer Reveals Sensitivity to Apoptosis Induced by Tumor Necrosis Factor–related Apoptosis-inducing Ligand in FGFR3-mutated Tumors. *European Urology* (2024) 85:483–494. doi: [10.1016/J.EURURO.2023.05.037](https://doi.org/10.1016/J.EURURO.2023.05.037)

36. Damrauer JS, Beckabir W, Klomp J, Zhou M, Plimack ER, Galsky MD, Grivas P, Hahn NM, O’Donnell PH, Iyer G, et al. Collaborative study from the Bladder Cancer Advocacy Network for the genomic analysis of metastatic urothelial cancer. *Nature communications* (2022) 13: doi: [10.1038/S41467-022-33980-9](https://doi.org/10.1038/S41467-022-33980-9)

37. Hastie T, Tibshirani R, Narasimhan B, Chu G. impute: Imputation for microarray data. (2023) <https://bioconductor.org/packages/release/bioc/html/impute.html>

38. Leek JT, Johnson WE, Parker HS, Jaffe AE, Storey JD. The sva package for removing batch effects and other unwanted variation in high-throughput experiments. *Bioinformatics* (2012) 28:882. doi: [10.1093/BIOINFORMATICS/BTS034](https://doi.org/10.1093/BIOINFORMATICS/BTS034)

39. Kamoun A, Reyniès A de, Allory Y, Sjödahl G, Robertson AG, Seiler R, Hoadley KA, Groeneveld CS, Al-Ahmadie H, Choi W, et al. A Consensus Molecular Classification of Muscle-invasive Bladder Cancer. *European urology* (2020) 77:420. doi: [10.1016/J.EURURO.2019.09.006](https://doi.org/10.1016/J.EURURO.2019.09.006)

40. Sturm G, Finotello F, List M. Immunedeconv: An R Package for Unified Access to Computational Methods for Estimating Immune Cell Fractions from Bulk RNA-Sequencing Data. *Methods in molecular biology (Clifton, NJ)* (2020) 2120:223–232. doi: [10.1007/978-1-0716-0327-7_16](https://doi.org/10.1007/978-1-0716-0327-7_16)

41. Finotello F, Mayer C, Plattner C, Laschober G, Rieder Di, Hackl H, Krogsdam A, Loncova Z, Posch W, Wilflingseder D, et al. Molecular and pharmacological modulators of the tumor immune contexture revealed by deconvolution of RNA-seq data. *Genome Medicine* (2019) 11:34. doi: [10.1186/s13073-019-0638-6](https://doi.org/10.1186/s13073-019-0638-6)

42. Aran D, Hu Z, Butte AJ. xCell: Digitally portraying the tissue cellular heterogeneity landscape. *Genome Biology* (2017) 18:220. doi: [10.1186/s13059-017-1349-1](https://doi.org/10.1186/s13059-017-1349-1)

43. Ramaker RC, Lasseigne BN, Hardigan AA, Palacio L, Gunther DS, Myers RM, Cooper SJ. RNA sequencing-based cell proliferation analysis across 19 cancers identifies a subset of proliferation-informative cancers with a common survival signature. *Oncotarget* (2017) 8:38668. doi: [10.18632/ONCOTARGET.16961](https://doi.org/10.18632/ONCOTARGET.16961)

44. Li X, Luo S, Fu W, Huang M, Huang X, Kang S, Zhang J, Wang Q, Song C. Discovery of a proliferation essential gene signature and actin-like 6A as potential biomarkers for predicting prognosis and neoadjuvant chemotherapy response in triple-positive breast cancer. *Cancer* (2024) 130:1435–1448. doi: [10.1002/CNCR.35228](https://doi.org/10.1002/CNCR.35228)

45. Altorki NK, Bhinder B, Borczuk AC, Elemento O, Mittal V, McGraw TE. A signature of enhanced proliferation associated with response and survival to anti-PD-L1 therapy in early-stage non-small cell lung cancer. *Cell Reports Medicine* (2024) 5:101438. doi: [10.1016/J.XCRM.2024.101438](https://doi.org/10.1016/J.XCRM.2024.101438)

46. Locard-Paulet M, Palasca O, Jensen LJ. Identifying the genes impacted by cell proliferation in proteomics and transcriptomics studies. *PLOS Computational Biology* (2022) 18:e1010604. doi: [10.1371/JOURNAL.PCBI.1010604](https://doi.org/10.1371/JOURNAL.PCBI.1010604)

47. Pang KL, Li P, Yao XR, Xiao WT, Ren X, He JY. Deciphering a proliferation-essential gene signature based on CRISPR-Cas9 screening to predict prognosis and characterize the immune microenvironment in HNSCC. *BMC Cancer* (2025) 25:1–20. doi: [10.1186/S12885-025-14181-1/FIGURES/14](https://doi.org/10.1186/S12885-025-14181-1/FIGURES/14)

48. Venet D, Dumont JE, Detours V. Most random gene expression signatures are significantly associated with breast cancer outcome. *PLoS Computational Biology* (2011) 7: doi: [10.1371/journal.pcbi.1002240](https://doi.org/10.1371/journal.pcbi.1002240)

49. Ingebriktsen LM, Finne K, Akslen LA, Wik E. A novel age-related gene expression signature associates with proliferation and disease progression in breast cancer. *British Journal of Cancer 2022 127:10* (2022) 127:1865–1875. doi: [10.1038/s41416-022-01953-w](https://doi.org/10.1038/s41416-022-01953-w)

50. Hänzelmann S, Castelo R, Guinney J. GSVA: Gene set variation analysis for microarray and RNA-Seq data. *BMC Bioinformatics* (2013) 14:7. doi: [10.1186/1471-2105-14-7](https://doi.org/10.1186/1471-2105-14-7)

51. Benjamin D, Sato T, Cibulskis K, Getz G, Stewart C, Lichtenstein L. Calling Somatic SNVs and Indels with Mutect2. *bioRxiv* (2019)861054. doi: [10.1101/861054](https://doi.org/10.1101/861054)

52. Yang W, Soares J, Greninger P, Edelman EJ, Lightfoot H, Forbes S, Bindal N, Beare D, Smith JA, Thompson IR, et al. Genomics of Drug Sensitivity in Cancer (GDSC): a resource for therapeutic biomarker discovery in cancer cells. *Nucleic Acids Research* (2013) 41:D955–D961. doi: [10.1093/NAR/GKS1111](https://doi.org/10.1093/NAR/GKS1111)

53. Seashore-Ludlow B, Rees MG, Cheah JH, Coko M, Price EV, Coletti ME, Jones V, Bodycombe NE, Soule CK, Gould J, et al. Harnessing Connectivity in a Large-Scale Small-Molecule Sensitivity Dataset. *Cancer discovery* (2015) 5:1210–1223. doi: [10.1158/2159-8290.CD-15-0235](https://doi.org/10.1158/2159-8290.CD-15-0235)

54. Corsello SM, Nagari RT, Spangler RD, Rossen J, Kocak M, Bryan JG, Humeidi R, Peck D, Wu X, Tang AA, et al. Discovering the anticancer potential of non-oncology drugs by systematic viability profiling. *Nature Cancer 2020 1:2* (2020) 1:235–248. doi: [10.1038/s43018-019-0018-6](https://doi.org/10.1038/s43018-019-0018-6)

55. DepMap. Current DepMap Release data, including CRISPR Screens, PRISM Drug Screens, Copy Number, Mutation, Expression, and Fusions. *Figshare+* (2024) Broad: doi: [10.25452/figshare.plus.25880521.v1](https://doi.org/10.25452/figshare.plus.25880521.v1)

56. Geeleher P, Cox N, Stephanie Huang R. pRRophetic: An R Package for Prediction of Clinical Chemotherapeutic Response from Tumor Gene Expression Levels. *PLOS ONE* (2014) 9:e107468. doi: [10.1371/JOURNAL.PONE.0107468](https://doi.org/10.1371/JOURNAL.PONE.0107468)

57. Friedman J, Hastie T, Tibshirani R. Regularization paths for generalized linear models via coordinate descent. *Journal of Statistical Software* (2010) 33:1–22. doi: [10.18637/jss.v033.i01](https://doi.org/10.18637/jss.v033.i01)

58. Törzsök P, Santer FR, Kunz Y, Creij NCH van, Tymoszuk P, Klinglmair G, Culig Z, Pichler R. Biological and therapeutic implications of sex hormone-related gene clustering in testicular cancer. *Basic and clinical andrology* (2025) 35: doi: [10.1186/S12610-025-00254-5](https://doi.org/10.1186/S12610-025-00254-5)

59. Müller-Dott S, Tsirvouli E, Vazquez M, Ramirez Flores RO, Badia-i-Mompel P, Fallegger R, Türei D, Lægreid A, Saez-Rodriguez J. Expanding the coverage of regulons from high-confidence prior knowledge for accurate estimation of transcription factor activities. *Nucleic acids research* (2023) 51:10934–10949. doi: [10.1093/NAR/GKAD841](https://doi.org/10.1093/NAR/GKAD841)

60. Schubert M, Klinger B, Klünemann M, Sieber A, Uhlitz F, Sauer S, Garnett MJ, Blüthgen N, Saez-Rodriguez J. Perturbation-response genes reveal signaling footprints in cancer gene expression. *Nature Communications 2017 9:1* (2018) 9:1–11. doi: [10.1038/s41467-017-02391-6](https://doi.org/10.1038/s41467-017-02391-6)

61. Badia-I-Mompel P, Vélez Santiago J, Braunger J, Geiss C, Dimitrov D, Müller-Dott S, Taus P, Dugourd A, Holland CH, Ramirez Flores RO, et al. decoupleR: ensemble of computational methods to infer biological activities from omics data. *Bioinformatics Advances* (2022) 2: doi: [10.1093/BIOADV/VBAC016](https://doi.org/10.1093/BIOADV/VBAC016)

62. Yao Z, Xu N, Shang G, Wang H, Tao H, Wang Y, Qin Z, Tan S, Feng J, Zhu J, et al. Proteogenomics of different urothelial bladder cancer stages reveals distinct molecular features for papillary cancer and carcinoma in situ. *Nature communications* (2023) 14: doi: [10.1038/S41467-023-41139-3](https://doi.org/10.1038/S41467-023-41139-3)

63. Dressler FF, Diedrichs F, Sabtan D, Hinrichs S, Krisp C, Gemoll T, Hennig M, Mackedanz P, Schlotfeldt M, Voß H, et al. Proteomic analysis of the urothelial cancer landscape. *Nature Communications* (2024) 15: doi: [10.1038/S41467-024-48096-5](https://doi.org/10.1038/S41467-024-48096-5)

64. Stroggilos R, Mokou M, Latosinska A, Makridakis M, Lygirou V, Mavrogeorgis E, Drekolias D, Frantzi M, Mullen W, Fragkoulis C, et al. Proteome-based classification of Nonmuscle Invasive Bladder Cancer. *International journal of cancer* (2020) 146:281–294. doi: [10.1002/IJC.32556](https://doi.org/10.1002/IJC.32556)

65. Dempster JM, Pacini C, Pantel S, Behan FM, Green T, Krill-Burger J, Beaver CM, Younger ST, Zhivich V, Najgebauer H, et al. Agreement between two large pan-cancer CRISPR-Cas9 gene dependency data sets. *Nature Communications* (2019) 10:5817. doi: [10.1038/S41467-019-13805-Y](https://doi.org/10.1038/S41467-019-13805-Y)

66. Dempster JM, Boyle I, Vazquez F, Root DE, Boehm JS, Hahn WC, Tsherniak A, McFarland JM. Chronos: a cell population dynamics model of CRISPR experiments that improves inference of gene fitness effects. *Genome Biology* (2021) 22:1–23. doi: [10.1186/S13059-021-02540-7/FIGURES/6](https://doi.org/10.1186/S13059-021-02540-7/FIGURES/6)

67. Chen B, Zheng X, Wu J, Chen G, Yu J, Xu Y, Wu WKK, Tse GMK, To KF, Kang W. Antibody–drug conjugates in cancer therapy: current landscape, challenges, and future directions. *Molecular Cancer 2025 24:1* (2025) 24:279–. doi: [10.1186/S12943-025-02489-2](https://doi.org/10.1186/S12943-025-02489-2)

68. Wang R, Hu B, Pan Z, Mo C, Zhao X, Liu G, Hou P, Cui Q, Xu Z, Wang W, et al. Antibody-Drug Conjugates (ADCs): current and future biopharmaceuticals. *Journal of hematology & oncology* (2025) 18: doi: [10.1186/S13045-025-01704-3](https://doi.org/10.1186/S13045-025-01704-3)

69. Da Costa JB, Gibb EA, Bivalacqua TJ, Liu Y, Zarni Oo H, Miyamoto DT, Alshalalfa M, Davicioni E, Wright J, Dall’Era MA, et al. Molecular characterization of neuroendocrinelike bladder cancer. *Clinical Cancer Research* (2019) 25:3908–3920. doi: [10.1158/1078-0432.CCR-18-3558/74172/AM/MOLECULAR-CHARACTERIZATION-OF-NEUROENDOCRINE-LIKE](https://doi.org/10.1158/1078-0432.CCR-18-3558/74172/AM/MOLECULAR-CHARACTERIZATION-OF-NEUROENDOCRINE-LIKE)

70. Akbulut D, Al-Ahmadie H. Neuroendocrine tumours of the urinary bladder: recent advances. *Histopathology* (2026) 88:108–121. doi: [10.1111/HIS.70044](https://doi.org/10.1111/HIS.70044)

71. Zhang T, Zhang T, Zhang X, Qian L, Hu C, Li J. Molecular and immune characteristics of neuroendocrine bladder carcinoma — Implications for diagnosis, prognosis, and therapy: A review. *Biomolecules and Biomedicine* (2025) 26:711–719. doi: [10.17305/bb.2025.13151](https://doi.org/10.17305/bb.2025.13151)

72. Balanis NG, Sheu KM, Esedebe FN, Patel SJ, Smith BA, Park JW, Alhani S, Gomperts BN, Huang J, Witte ON, et al. Pan-cancer convergence to a small cell neuroendocrine phenotype that shares susceptibilities with hematological malignancies. *Cancer cell* (2019) 36:17. doi: [10.1016/J.CCELL.2019.06.005](https://doi.org/10.1016/J.CCELL.2019.06.005)

73. Therneau TM, Grambsch PM. *Modeling Survival Data: Extending the Cox Model*. 1st ed. New York: Springer Verlag (2000).

74. Efron B. Better bootstrap confidence intervals. *Journal of the American Statistical Association* (1987) 82:171–185. doi: [10.1080/01621459.1987.10478410](https://doi.org/10.1080/01621459.1987.10478410)

75. Kassambara A, Kosinski M, Biecek P. survminer: Drawing Survival Curves using ’ggplot2’. (2016) <https://cran.r-project.org/package=survminer>

76. Benjamini Y, Hochberg Y. Controlling the False Discovery Rate: A Practical and Powerful Approach to Multiple Testing. *Journal of the Royal Statistical Society: Series B (Methodological)* (1995) 57:289–300. doi: [10.1111/j.2517-6161.1995.tb02031.x](https://doi.org/10.1111/j.2517-6161.1995.tb02031.x)

77. Balduzzi S, Rücker G, Schwarzer G. How to perform a meta-analysis with R: A practical tutorial. *Evidence-Based Mental Health* (2019) 22:153–160. doi: [10.1136/ebmental-2019-300117](https://doi.org/10.1136/ebmental-2019-300117)

78. Funder DC, Ozer DJ. Evaluating Effect Size in Psychological Research: Sense and Nonsense. *Advances in Methods and Practices in Psychological Science* (2019) 2:156–168. doi: [10.1177/2515245919847202](https://doi.org/10.1177/2515245919847202)

79. Cohen J. Statistical Power Analysis for the Behavioral Sciences. *Statistical Power Analysis for the Behavioral Sciences* (2013) doi: [10.4324/9780203771587](https://doi.org/10.4324/9780203771587)

80. Field AP. Discovering statistics using IBM SPSS Statistics: and sex and drugs and rock ‘n’ roll, 4th edition. *Choice Reviews Online* (2013) 50:xviii, 908, xxxvi. <http://www.uk.sagepub.com/field4e/default.htm>

81. Cohen J. A Coefficient of Agreement for Nominal Scales. *Educational and Psychological Measurement* (1960) 20:37–46. doi: [10.1177/001316446002000104](https://doi.org/10.1177/001316446002000104)

82. McHugh ML. Interrater reliability: the kappa statistic. *Biochemia Medica* (2012) 22:276. doi: [10.11613/bm.2012.031](https://doi.org/10.11613/bm.2012.031)

83. Rice ME, Harris GT. Comparing effect sizes in follow-up studies: ROC area, Cohen’s d, and r. *Law and Human Behavior* (2005) 29:615–620. doi: [10.1007/S10979-005-6832-7](https://doi.org/10.1007/S10979-005-6832-7)

84. Vesanto J, Alhoniemi E. Clustering of the self-organizing map. *IEEE Transactions on Neural Networks* (2000) 11:586–600. doi: [10.1109/72.846731](https://doi.org/10.1109/72.846731)

85. Kohonen T. *Self-Organizing Maps*. Berlin, Heidelberg: Springer Berlin Heidelberg (1995). doi: [10.1007/978-3-642-97610-0](https://doi.org/10.1007/978-3-642-97610-0)

86. Wehrens R, Kruisselbrink J. Flexible self-organizing maps in kohonen 3.0. *Journal of Statistical Software* (2018) 87:1–18. doi: [10.18637/jss.v087.i07](https://doi.org/10.18637/jss.v087.i07)

87. Venna J, Kaski S. Neighborhood preservation in nonlinear projection methods: An experimental study. *Lecture Notes in Computer Science (including subseries Lecture Notes in Artificial Intelligence and Lecture Notes in Bioinformatics)* (2001) 2130:485–491. doi: [10.1007/3-540-44668-0_68](https://doi.org/10.1007/3-540-44668-0_68)

88. Young MD, Wakefield MJ, Smyth GK, Oshlack A. Gene ontology analysis for RNA-seq: accounting for selection bias. *Genome Biology* (2010) 11:R14. doi: [10.1186/gb-2010-11-2-r14](https://doi.org/10.1186/gb-2010-11-2-r14)

89. Raymaekers J, Zamar RH. Regularized K-means Through Hard-Thresholding. *Journal of Machine Learning Research* (2022) 23:1–48. <http://jmlr.org/papers/v23/21-0052.html>

90. Rousseeuw PJ. Silhouettes: A graphical aid to the interpretation and validation of cluster analysis. *Journal of Computational and Applied Mathematics* (1987) 20:53–65. doi: [10.1016/0377-0427(87)90125-7](https://doi.org/10.1016/0377-0427(87)90125-7)

91. Lange T, Roth V, Braun ML, Buhmann JM. Stability-based validation of clustering solutions. *Neural Computation* (2004) 16:1299–1323. doi: [10.1162/089976604773717621](https://doi.org/10.1162/089976604773717621)

92. Leng M, Wang J, Cheng J, Zhou H, Chen X. Adaptive semi-supervised clustering algorithm with label propagation. *Journal of Software Engineering* (2014) 8:14–22. doi: [10.3923/jse.2014.14.22](https://doi.org/10.3923/jse.2014.14.22)

93. Zou H, Hastie T. Regularization and variable selection via the elastic net. *Journal of the Royal Statistical Society Series B: Statistical Methodology* (2005) 67:301–320. doi: [10.1111/j.1467-9868.2005.00503.x](https://doi.org/10.1111/j.1467-9868.2005.00503.x)

94. Brier GW. VERIFICATION OF FORECASTS EXPRESSED IN TERMS OF PROBABILITY. *Monthly Weather Review* (1950) 78:1–3. doi: [10.1175/1520-0493(1950)078<0001:vofeit>2.0.co;2](https://doi.org/10.1175/1520-0493(1950)078%3c0001:vofeit%3e2.0.co;2)

95. Kuhn M. Building predictive models in R using the caret package. *Journal of Statistical Software* (2008) 28:1–26. doi: [10.18637/jss.v028.i05](https://doi.org/10.18637/jss.v028.i05)

96. Grambsch PM, Therneau TM. Proportional Hazards Tests and Diagnostics Based on Weighted Residuals. *Biometrika* (1994) 81:515. doi: [10.2307/2337123](https://doi.org/10.2307/2337123)

97. Harrell FE, Lee KL, Mark DB. Multivariable prognostic models: Issues in developing models, evaluating assumptions and adequacy, and measuring and reducing errors. *Statistics in Medicine* (1996) 15:361–387. doi: [10.1002/(SICI)1097-0258(19960229)15:4<361::AID-SIM168>3.0.CO;2-4](https://doi.org/10.1002/(SICI)1097-0258(19960229)15:4%3c361::AID-SIM168%3e3.0.CO;2-4)

98. Graf E, Schmoor C, Sauerbrei W, Schumacher M. Assessment and comparison of prognostic classification schemes for survival data. *Statistics in Medicine* (1999) 18:2529–2545. doi: [10.1002/(sici)1097-0258(19990915/30)18:17/18<2529::aid-sim274>3.0.co;2-5](https://doi.org/10.1002/(sici)1097-0258(19990915/30)18:17/18%3c2529::aid-sim274%3e3.0.co;2-5)

99. Royston P, Group C. Explained variation for survival models. *Stata Journal* (2006) 6:83–96. doi: [10.1177/1536867X0600600105](https://doi.org/10.1177/1536867X0600600105)

100. Brunson JC. ggalluvial: Layered Grammar for Alluvial Plots. *Journal of Open Source Software* (2020) 5:2017. doi: [10.21105/JOSS.02017](https://doi.org/10.21105/JOSS.02017)

101. Pichler R, Creij NCH van, Mertens LS, Giudice F del, Koll F, Soria F, Subiela JD, Plage H, Tymoszuk P, Mayr R, et al. FGFR1/3 Signaling as an Achilles’ Heel of Phenotypic Diversity in Urothelial Carcinoma. *European urology oncology* (2025) doi: [10.1016/J.EUO.2025.07.005](https://doi.org/10.1016/J.EUO.2025.07.005)

102. Zhang B, Horvath S. A general framework for weighted gene co-expression network analysis. *Statistical Applications in Genetics and Molecular Biology* (2005) 4: doi: [10.2202/1544-6115.1128/MACHINEREADABLECITATION/RIS](https://doi.org/10.2202/1544-6115.1128/MACHINEREADABLECITATION/RIS)

103. Traag VA, Waltman L, Eck NJ van. From Louvain to Leiden: guaranteeing well-connected communities. *Scientific Reports 2019 9:1* (2019) 9:1–12. doi: [10.1038/s41598-019-41695-z](https://doi.org/10.1038/s41598-019-41695-z)

104. Naba A, Clauser KR, Hoersch S, Liu H, Carr SA, Hynes RO. The matrisome: In silico definition and in vivo characterization by proteomics of normal and tumor extracellular matrices. *Molecular and Cellular Proteomics* (2012) 11:M111.014647. doi: [10.1074/mcp.M111.014647](https://doi.org/10.1074/mcp.M111.014647)

105. Petrov PB, Considine JM, Izzi V, Naba A. Matrisome AnalyzeR – a suite of tools to annotate and quantify ECM molecules in big datasets across organisms. *Journal of Cell Science* (2023) 136: doi: [10.1242/JCS.261255/325836/AM/MATRISOME-ANALYZER-A-SUITE-OF-TOOLS-TO-ANNOTATE](https://doi.org/10.1242/JCS.261255/325836/AM/MATRISOME-ANALYZER-A-SUITE-OF-TOOLS-TO-ANNOTATE)

106. Thiele I, Swainston N, Fleming RMT, Hoppe A, Sahoo S, Aurich MK, Haraldsdottir H, Mo ML, Rolfsson O, Stobbe MD, et al. A community-driven global reconstruction of human metabolism. *Nature Biotechnology 2013 31:5* (2013) 31:419–425. doi: [10.1038/nbt.2488](https://doi.org/10.1038/nbt.2488)

107. Heidegger I, Frantzi M, Salcher S, Tymoszuk P, Martowicz A, Gomez-Gomez E, Blanca A, Lendinez Cano G, Latosinska A, Mischak H, et al. Prediction of Clinically Significant Prostate Cancer by a Specific Collagen-related Transcriptome, Proteome, and Urinome Signature. *European urology oncology* (2024) doi: [10.1016/J.EUO.2024.05.014](https://doi.org/10.1016/J.EUO.2024.05.014)

108. Pichler R, Siska PJ, Tymoszuk P, Martowicz A, Untergasser G, Mayr R, Weber F, Seeber A, Kocher F, Barth DA, et al. A chemokine network of T cell exhaustion and metabolic reprogramming in renal cell carcinoma. *Frontiers in Immunology* (2023) 14:1208. doi: [10.3389/FIMMU.2023.1095195/BIBTEX](https://doi.org/10.3389/FIMMU.2023.1095195/BIBTEX)

109. Gavai AK, Supandi F, Hettling H, Murrell P, Leunissen JAM, Van Beek JHGM. Using Bioconductor Package BiGGR for Metabolic Flux Estimation Based on Gene Expression Changes in Brain. *PLOS ONE* (2015) 10:e0119016. doi: [10.1371/JOURNAL.PONE.0119016](https://doi.org/10.1371/JOURNAL.PONE.0119016)

110. King ZA, Lu J, Dräger A, Miller P, Federowicz S, Lerman JA, Ebrahim A, Palsson BO, Lewis NE. BiGG Models: A platform for integrating, standardizing and sharing genome-scale models. *Nucleic Acids Research* (2016) 44:D515–D522. doi: [10.1093/NAR/GKV1049](https://doi.org/10.1093/NAR/GKV1049)

111. Canty A, Ripley B, Brazzale A. boot: Bootstrap Functions. (2024) doi: [10.32614/CRAN.PACKAGE.BOOT](https://doi.org/10.32614/CRAN.PACKAGE.BOOT)

112. Lindskrog SV, Prip F, Lamy P, Taber A, Groeneveld CS, Birkenkamp-Demtröder K, Jensen JB, Strandgaard T, Nordentoft I, Christensen E, et al. An integrated multi-omics analysis identifies prognostic molecular subtypes of non-muscle-invasive bladder cancer. *Nature Communications 2021 12:1* (2021) 12:1–18. doi: [10.1038/s41467-021-22465-w](https://doi.org/10.1038/s41467-021-22465-w)

113. Larsson J. Area-Proportional Euler and Venn Diagrams with Ellipses. (2025) doi: [10.32614/CRAN.PACKAGE.EULERR](https://doi.org/10.32614/CRAN.PACKAGE.EULERR)

114. Parker JS, Mullins M, Cheung MCU, Leung S, Voduc D, Vickery T, Davies S, Fauron C, He X, Hu Z, et al. Supervised risk predictor of breast cancer based on intrinsic subtypes. *Journal of Clinical Oncology* (2009) 27:1160–1167. doi: [10.1200/JCO.2008.18.1370](https://doi.org/10.1200/JCO.2008.18.1370)
